# Forensic Proteomics for the Discovery of New *post mortem* Interval Biomarkers: A Preliminary Study

**DOI:** 10.3390/ijms241914627

**Published:** 2023-09-27

**Authors:** Alessandro Marrone, Daniele La Russa, Laura Barberio, Maria Stella Murfuni, Marco Gaspari, Daniela Pellegrino

**Affiliations:** 1 Department of Biology, Ecology and Earth Sciences, University of Calabria, 87036 Rende, Italy; alessandro.marrone@unical.it (A.M.); daniele.larussa@unical.it (D.L.R.); laura.barberio@unical.it (L.B.); 2 Department of Experimental and Clinical Medicine, Magna Graecia University, 88100 Catanzaro, Italy; murfuni@unicz.it (M.S.M.); gaspari@unicz.it (M.G.)

**Keywords:** *post mortem* interval (PMI), proteomics, mass spectrometry, biomarkers, forensic

## Abstract

Estimating the time since death (*post mortem* interval, PMI) represents one of the most important tasks in daily forensic casework. For decades, forensic scientists have investigated changes in *post mortem* body composition, focusing on different physical, chemical, or biological aspects, to discover a reliable method for estimating PMI; nevertheless, all of these attempts remain unsuccessful considering the currently available methodical spectrum characterized by great inaccuracies and limitations. However, recent promising approaches focus on the *post mortem* decomposition of biomolecules. In particular, significant advances have been made in research on the *post mortem* degradation of proteins. In the present study, we investigated early *post mortem* changes (during the first 24 h) in the proteome profile of the pig skeletal muscle looking for new PMI specific biomarkers. By mass spectrometry (MS)-based proteomics, we were able to identify a total of nine potential PMI biomarkers, whose quantity changed constantly and progressively over time, directly or inversely proportional to the advancement of *post mortem* hours. Our preliminary study underlines the importance of the proteomic approach in the search for a reliable method for PMI determination and highlights the need to characterize a large number of reliable marker proteins useful in forensic practice for PMI estimation.

## 1. Introduction

*Post mortem* interval (PMI) estimation is undoubtedly the most important factor in forensic science and the accuracy of this determination has always been a real challenge. Knowing the time since death helps reveal the circumstances of death and thus, in criminal cases, provides decisive evidence in court for the resolution of a crime [1]. Currently in criminal investigations, the PMI is estimated through the comparison of various parameters, in the first hours/few days of death using the classic triad *livor*-*rigor*-*algor mortis* [2] and in the following times through the analysis of the transformative phenomena of the corpse also assisted by forensic entomology [3]. However, these methodologies have precise limitations that depend mainly on the time interval from death but also on environmental factors (temperature, humidity, ventilation, etc.) and intrinsic characteristics of the corpse (body mass, age, muscle development, etc.). Therefore, additional approaches are required to complement the conventionally applied methodologies and to provide a reliable set of tools to improve PMI estimation. Recently, scientific research has focused on *post mortem* alterations in biomolecules and this field holds particular promise in forensic practice since the inclusion of biochemical methods in a multidisciplinary approach can also help eliminate examiner bias, which imposes always a risk factor within the techniques currently available [4]. In this regard, several studies have investigated the *post mortem* alterations of RNA [5], DNA [6], or proteins [7]. In particular, the use of technologies such as proteomics are opening up effective scenarios in the identification of new biomarkers in forensic science as they allow for the analysis, identification, and efficient and reproducible characterization of peptides and proteins from different biological matrices. Thanks to this innovative approach, the analysis of time-dependent nucleic acids (DNA and RNA), widely investigated in the forensic field, is replaced by the analysis of proteins. Indeed, protein markers are resistant, and not very susceptible to environmental conditions, with reduced sample contamination problems compared to DNA, while providing highly reliable data. Moreover, protein degradation after death is slower and more reproducible than the degradation of RNAs. In the last twenty years, indeed, a sum of evidence has demonstrated that specific proteins have been found to exhibit degradation patterns that are predictable and significantly correlated with PMI [7,8]. Therefore, the study of the chrono-degradation of proteins represents a very promising tool in forensic PMI estimation [7,8].

As regards the target tissues of studies on protein degradation, skeletal muscle tissue is receiving a lot of attention, indeed, several authors are describing muscle as an ideal sample to analyse time-dependent protein degradation [4,9]. This choice of skeletal muscle as a candidate tissue for PMI estimation is mainly due to two factors: it is the most abundant tissue in the human body [10] and it is easily accessible while being relatively well protected by the skin from environmental influences [11]. In addition, *post mortem* changes in this tissue occur more slowly compared to internal organs and nervous tissue [12], but still faster than in cartilage and bone [13]. The *post mortem* degradation of skeletal muscle has also been extensively investigated in the context of meat quality, in terms of tenderness and storage [14]. Several studies, using standardized animal models, have analysed the *post mortem* changes of structural muscle proteins thanks to biochemical techniques such as Western blotting or zymography and have revealed interesting results such as the loss of the native protein band or the appearance of specific degradation products, significantly correlated with PMI [4,9]. It is no coincidence that the proteins showing the highest level of evidence to date are muscle proteins. Among these proteins are, for example, several cytoskeletal proteins as titin, nebulin, desmin, troponin, vinculin, actin, calpain, alpha-tubulin, alpha-actinin, but also sarco-endoplasmic reticulum calcium ATPase (SERCA1) and glyceraldehyde 3-phosphate dehydrogenase (GAPDH). All these proteins have different rates of degradation, but each of these gives rise to the related breakdown products of lower molecular weight, whose appearance or disappearance may occur in specific *post mortem* ranges [7]. However, although several research groups have been able to identify such changes, only very few appear to be practicable or accurate enough to hold true for daily forensic work. For this reason, it is strongly recommended to characterize additional marker proteins, which exhibit predictable degradation patterns, in order to provide a widely applicable method for PMI estimation. Mass spectrometry-based proteomics is a powerful new tool that enables the large-scale study of all proteins in an organism or system [15]. It can provide a holistic view of the physiological and biochemical states of certain samples through the efficient and reproducible identification and quantification of a large number of peptides and proteins with high accuracy and sensitivity [16]. Mass spectrometry-based proteomics is an extremely successful and rapidly growing research tool in the biological sciences and since proteomics methods are potentially applicable whenever looking for information about the proteins contained in a sample, the forensic field is no exception. In recent years, the applicability of proteomics to important forensic questions has been increasingly recognized, however, the application of mass spectrometry for PMI biomarker discovery is still in the early stages of development, as evidenced by the few recent studies in the literature [9]. In the present study, we investigate early *post mortem* changes (during the first 24 h) in the proteome profile of the pig (*Sus scrofa domesticus*) skeletal muscle, commonly used as a human analogue in decomposition studies, to look for new PMI specific biomarkers. This preliminary study aims to implement a protein-based methodology for forensic PMI estimation, and although we identified new protein markers, the limited number of samples did not allow us to verify the identified proteins using quantitative methods for set up mathematical models to predict PMI.

## 2. Results

**Figure 1:**
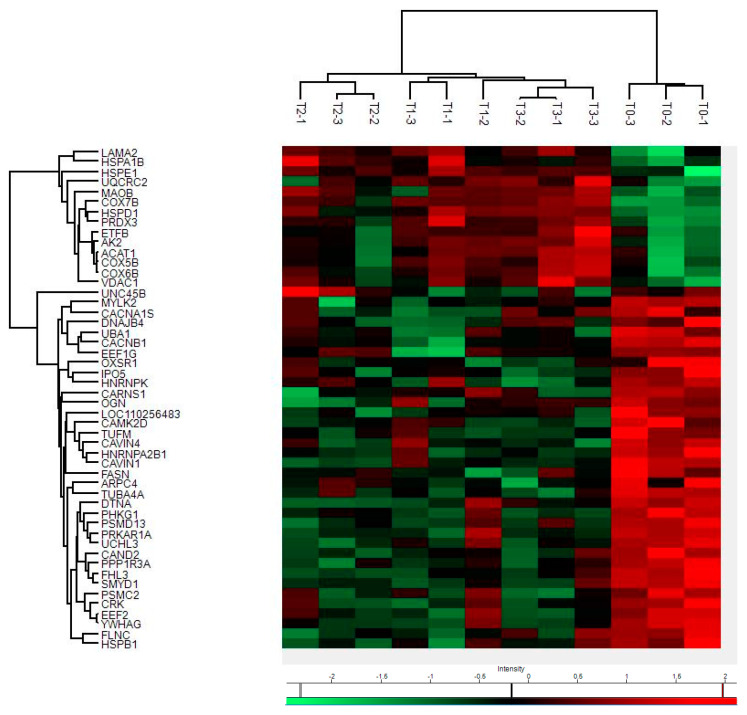
Hierarchical clustering and Heat Map of protein intensities at various hpm. Progressively brighter shades of red indicate greater abundance, while progressively brighter shades of green indicate lower abundance.

**Figure 2:**
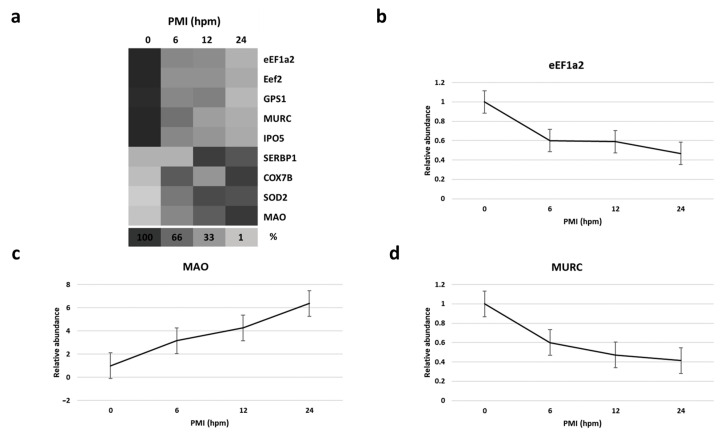
(**a**) Heat map depicting the percentage amount of each potential identified biomarker as a function of hours *post mortem* (hpm).Progressively brighter shades of the grayscale indicate lower abundance.(**b**–**d**), respectively, show the relative abundance of eEF1A2, MAO, and MURC at various hpm. Data represented as mean ± SD.

By mass spectrometry (MS)-based proteomics, we investigated early the *post mortem* changes [time intervals 0 (T0), 6 (T1), 12 (T2), and 24 (T3) h *post mortem* or hpm] in the proteome profile of pig skeletal muscle (three samples per time-group) looking for new PMI specific biomarkers. We identified and quantified a total of 657 proteins from *Sus scrofa* (domestic pig) in the considered *post mortem* interval. According to protein intensities, a Hierarchical clustering was performed (Figure 1). Observing the heat map (Figure 1), the difference between the samples collected at T0 and all the others is evident. The evidence that samples T1 and T3 cluster with each other, independently of T2, suggests that the algorithm does not detect a progressive trend from T1 to T3. This outcome suggests that in the considered time window, the proteome changes quite sharply in the first 6 hpm (from T0 to T1), after which the variations occur more slowly. According to the protein intensity, protein changes have been classified into four groups. We identified down-regulated proteins, up-regulated proteins, and then proteins which showed an up-regulation after down-regulation and *viceversa*. Although some proteins showed nonlinear patterns, we focused our attention on those proteins whose quantity changed constantly and progressively over time (from each time point to the subsequent), directly or inversely proportional to the advancement of *post mortem* hours. We identified five proteins (eEF1A2, eEF2, GPS1, MURC, and IPO5) whose intensity quantitatively decreased with increasing *post mortem* hours and 4 (SERBP1, COX7B, SOD2, and MAO) which increased, for a total of nine identified potential markers (Figure 2a).

## 3. Discussion

In forensic science, the temporal reconstruction of the events represents a key point for the correct interpretation of the crime scene as it allows for the revealing of the dynamics of the criminal event. The determination of PMI can be decisive in reconstructing homicide cases and represents a continuous challenge in forensic research. In recent years, research to calculate the time elapsed since the death of a victim has been oriented towards the search for markers which are specificand reliable, but primarily free from exogenous and/or environmental interference. These new markers include the bloodstains aging [17] and the main biomolecules, DNA, RNA, and proteins, and these last ones seem to be particularly suitable for the estimation of the PMI [4]. Indeed, recent evidence has shown that the post-mortem expression and degradation of some proteins proceeds in a predictable and time-dependent manner [7]. In order to implement a protein-based methodology for forensic PMI estimation, the identification of additional protein markers in addition to the currently known is necessary [9]. In this preliminary research, we investigated early *post mortem* changes (during the first 24 h) in the proteome profile of the pig skeletal muscle, looking for new PMI specific biomarkers. By mass spectrometry (MS)-based proteomics, we were able to identify a total of nine potential PMI biomarkers, whose quantity changed constantly and progressively over time, directly or inversely proportional to the advancement of *post mortem* hours: five proteins (eEF1A2, eEF2, GPS1, MURC, and IPO5) whose intensity quantitatively decreases with increasing *post mortem* hours and four (SERBP1, COX7B, SOD2 and MAOB) which increase.

eEF2—Eukaryotic translation elongation factor 2—is involved in the translocation of eukaryotic protein synthesis. In the active form it is bound to GTP whose hydrolysis allows for the release of energy which can be coupled to the translocation [18]. GPS1—G protein pathway suppressor 1—is a component of COP9, a multifunctional protein complex required for proteasome-dependent protein degradation [19]. IPO5—Importin 5—belongs to the family of importins which mediate the transport of some proteins towards the interior of the nucleus through the recognition of specific sequences [20]. MURC—Muscle-restricted coiled-coil protein—stands out among other candidate biomarkers for PMI because it shows a continuous decrease in all considered intervals. This protein positively regulates skeletal muscle myogenesis (an event that characterizes the differentiation of a myogenic precursor in a skeletal muscle fiber) by increasing the expression of myogenin and activating ERK, a kinase involved in the cell differentiation pathway [21]. Within skeletal muscle, MURC is localized in the sarcomere, in particular there is an accumulation at the level of the Z disk. eEF1A2—Eukaryotic translation elongation factor 1 alpha 2—is an isoform of eEF1A and allows for the recruitment of aminoacyl-tRNA in eukaryotic protein synthesis. This protein deserves particular attention as it is already known in the literature, having been proposed by Choi and coworker [22] as a candidate biomarker for PMI estimation in murine organisms (mice and rats). The fact that this protein has recorded interesting trends in relation to PMI in different studies, which investigated different species, bodes well for the applicability of this biomarker also in humans.

Most studies analysing protein degradation in the early stages of PMI have highlighted a pattern of protein degradation or decrease. This phenomenon, which begins immediately after death, can be traced back to those enzymatic (proteolytic enzymes) and non-enzymatic processes (changes in pH values and temperature) which jointly act to degrade proteins into smaller fragments [23,24]. This fragmentation phenomenon has been confirmed in forensic studies and is also consistently detected by several studies from other scientific fields, including meat science [9,14]. Moreover, these studies also indicate that the protein variation (degradation and/or decrease) is protein specific. Decomposition rates vary between proteins, and also within the same tissue; therefore, individual proteins exhibit different susceptibility to *post mortem* proteolysis. The phenomenon of protein decomposition is very complex and influenced by various factors that act synergistically in determining the ways and times of cleavage of native proteins into more or less small fragments. Among these factors, they are of great importance differences in amino acid sequence, post-translational modification, all in turn influencing spatial structure, molecule function, and accounting for variability in protease cleavage sites [25,26].

However, although most of the proteins were found to decrease *postmortem*, we, as well as other authors, have found that certain proteins show an increase after death, often followed by a subsequent decrease. We have highlighted four proteins that behave like this: SERBP1, COX7B, SOD2, and MAOB. SERBP1—SERPINE1 mRNA binding protein 1—is a messenger RNA binding protein that has a primary role in the regulation of mRNA stability and is involved in post-transcriptional mechanisms [27]. In general, RNA-binding proteins (RBPs) function as master regulators of gene expression in both physiological and pathological conditions. For example, alterations in RBP expression and function are often observed in cancer and influence the critical pathways implicated in tumour initiation and growth [28,29,30]. COX7B—Cytochrome C oxidase subunit 7B—is a nuclear-encoded subunit of cytochrome C oxidase (COX), the terminal enzyme complex of the eukaryotic oxidative phosphorylation in the mitochondria [31]. SOD2—Superoxide dismutase 2—also known as manganese-dependent superoxide dismutase (MnSOD), is an enzyme which transforms toxic superoxide, a byproduct of the mitochondrial oxidative phosphorylation, into hydrogen peroxide and diatomic oxygen. This function allows SOD2 to clear mitochondrial reactive oxygen species (ROS) and, as a result, confer protection against cell death [32]. Therefore, this protein plays an antiapoptotic role against oxidative stress, ionizing radiation, and inflammatory cytokines [33]. MAO—Monoamine oxidase—is an enzyme, located in the outer mitochondrial membrane, belonging to the class of oxidoreductases which catalyses the oxidative deamination of amines present within the body, both endogenous and exogenous [34]. Monoamine oxidase seems to be particularly promising because it shows an important progressive increase in relation to the increase in PMI.

The cause of the counterintuitive *post mortem* increase in the level of some proteins is often defined as unknown, although there are several possible explanations for this phenomenon. Since mass spectrometry data are normalized on protein median, the apparent increase in levels of some proteins might simply reflect an increase in the relative abundance, and not the absolute abundance. Proteins less subjected to degradation will increase their relative abundance in post-mortem samples. Alternatively, in very early *post mortem* phases, it could result from continued protein synthesis, as evidenced also by Sanoudou and coworkers, who reported a high transcriptional and possibly also translational activity during the first hours *post mortem* in skeletal muscle [35]. Another interesting study explored the thanatotranscriptome up to 48 h *post mortem* in two model organisms: mouse and zebrafish, highlighting that in addition to the already mentioned genes involved in the onset and development of cancer, several upregulated genes were implicated in pathways related to apoptosis, inflammation, and different types of stress, such as oxidative stress [36].

After death, oxygen is not delivered by the circulatory system to *postmortem* muscle, therefore skeletal muscle metabolism changes from aerobic to anaerobic [37]. Anyway, anoxic *postmortem* muscle is biochemically active and some metabolic pathways do not stop instantly, while others are even started. Clearly, in adverse conditions, cells activate a wide array of mechanisms to re-establish homeostasis and to repair stress-induced molecular damage [38]. In particular, in the dying muscle, different routes/pathways of cell death catabolism (apoptosis, autophagy) may occur, having great influence on the process of converting muscle into meat [39]. In addition, post-mortem aging is a process of oxidative stress that inevitably generates reactive oxygen species (ROS) [40]. This oxygen deprivation condition, associated with increases in free radical activity, as happens with hypoxia or ischemia, inevitably involves the mitochondria [39]. Mitochondria are in fact the first and also one of the main organelles affected by post-mortem changes, therefore, they are decisive in the subsequent cellular responses influencing the pathway to cell demise [39]. Together with the endoplasmic reticulum, mitochondria constitute the major storage compartment for intracellular Ca^2+^ ions. Ca^2+^ fluxes not only regulate the bioenergetic and anabolic metabolism but also impact on the activation of cell death pathways and the response of cells to a wide number of perturbations, including oxidative stress [41,42]. These *post mortem* conditions might activate some oxidation related protein synthesis, while deactivating other proteins and metabolic pathways. For example, the two-fold increase in the mitochondrial enzymes COX7B, SOD2, and MAOB leads us to assume that oxidative phenomena are triggered after death. Similarly, such *post mortem* conditions may also influence other proteins that do not tend to show continuous increase or decrease. In distinct cases, the transient increase in a marker could be due to the loss of inhibition during life. The inhibitory units of enzymes can dissociate when membrane potentials break down, as exemplified by calpain activation caused by *post mortem* Ca^2+^ increased levels.

This study has potential limitations. First of all, the results obtained with an animal model are not directly transferable to humans. The use of pigs as a prototype species represents the best choice for forensic studies, as the pig anatomy and physiology present a high resemblance to humans, but comparing two species will be subject to uncertainties. Furthermore, in our preliminary study we evaluated only one specific state (death by bleeding of organisms with high BMI in an environment with non-extreme and relatively constant temperatures), thus numerous other experiments are necessary to validate the obtained results and the applicability to real forensic casework.

## 4. Materials and Methods

### 4.1. Animals and Sampling

Three male (castrated) breeding pigs (*Sus scrofa domesticus*), weighing about 120 kg (120.66 ± 7.02, mean ± SD), were used for this study. Animal samples were obtained from a commercial slaughterhouse. Muscle tissue was sampled from the belly of the musculus biceps femoris of the left hind limbs at pre-defined *post mortem* time intervals (0, 6, 12, and 24 hpm) during the maturation of the whole animal at a temperature between 12 and 18 °C. For sampling, an incision was made through the skin and the underlying fascia using a surgical sterile scalpel, and a piece of muscle tissue, with a size of approximately 2 × 2 × 2 cm (~2 g) was excised in a depth of 2 cm. To avoid artificial desiccation and contamination effects, the minimum distance between sampling sites (for each new time point) was 5 cm. The samples collected each time were sectioned to smaller pieces of approximately 100 mg, snap frozen in liquid nitrogen, and stored at −80 °C until further processing. Frozen tissues were homogenized manually, using a prechilled mortar and pestle, in ice-cold RIPA buffer containing a protease inhibitor cocktail (Sigma-Aldrich, Milan, Italy). After centrifugation for 20 min at 14,000× *g* at 4 °C, supernatants were collected and stored at −20 °C until further use. Protein concentration was determined by spectrophotometry using the Bradford method (Sigma, St Louis, MO, USA).

### 4.2. Proteomic Analysis

All chemicals used in the experiments described were purchased from Sigma-Aldrich (St. Louis, MO, USA) unless otherwise specified.

FASP digestion—Supernatant 100 μL (50 µg) were dissolved in SDS 1%, 50 mM DTT, and 50 mM Tris HCl pH 8.0 and heated at 95 °C for 10 min. Protein samples were added to the filter unit (Microcon, Centrifugal Filters, Merck Millipore Ltd., Burlington, MA, USA) and centrifuged at 14,000× *g* for 15 min. Subsequently, two washes were performed with urea buffer (8M urea, 100 mM Tris buffer, pH 8.0). Cysteine alkylation was performed with 50 mM iodoacetamide (IAA in 8M urea buffer) for 20 min. Before the addition of the trypsin enzyme, two washes with urea buffer and two washes with 50 mM TEAB were performed. Then, 500 ng of trypsin were added to each sample for digestion at 37 °C overnight. The following day 140 µL of H2O were added and the samples were centrifuged at 14,000× *g* for 20 min to collect 180–200 µL of digest.

Peptides purification—Peptides (5 µg) were purified by strong cation exchange (SCX) StageTips method, aiming at removing eventual detergent residues. The stationary phase was 1 mm^3^ of EmporeTM-3M SCX resin (Merck Millipore). The digested samples were acidified by adding 80% acetonitrile-0.5% formic acid (Solution W2). The StageTip was conditioned by adding 20% acetonitrile-0.5% formic acid (Solution W1,) and 50 µL of W2. Peptides were loaded by slowly letting the fluid pass through one plug using a benchtop centrifuge. After 2 washes with 50 µL of Solution W2 and 50 µL of Solution W1, respectively, peptides were eluted by adding 10 µL of 500 mM ammonium acetate-20% acetonitrile.

LC-MSMS analysis—LFQ (label free quantification) analysis was performed by an LC-MS/MS system consisting of an Easy nLC-1000 chromatographic instrument coupled to a Q-Exactive-Orbitrap mass spectrometer (both from Thermo Scientific, Bremen, Germany). All the LC-MS/MS analyses were carried out at 230μL/min flowrate and peptides were eluted by using two mobile phases: A (0.1% FA in 2% ACN) and B (20% water, 80% acetonitrile, and 0.1% formic acid). LC-MS/MS analysis was performed in data-dependent acquisition (DDA). The full scan m/z range was 350–1800, followed by MS/MS scans on the 12 most intense precursor ions. DDA analysis was performed with resolution for full MS scan of 70,000 and of 35,000 for MS/MS scan; the isolation window was 1.6 *m*/*z*. The maximum injection time was set to 50 ms for full MS scans and to 120 ms for MS/MS scans.

### 4.3. Data Analysis

Raw MS data (available in supplementary material) were analysed through MaxQuant software (2.0.1.0 version) using the Andromeda search engine. The MS/MS spectra were searched against a *Sus Scrofa* proteome database (49,972 sequences downloaded on 28 October 2021).For LF samples the following settings were used: fixed modifications: carbamidomethyl (C); variable modification: oxidized methionine (M) and acetyl (N-terminus). Unique peptides were used for protein identification. Forthe statistical analysis of MaxQuant output, the Perseus software (1.6.1.3 version) was used as follows: the LFQ intensity of proteins from the MaxQuant analysis were imported and contaminants, reverse identification, and proteins only identified by site were excluded from further data analysis. Data were transformed in logarithmic scale (log2). Proteins quantified in less than two replicates belonging to at least one group were discarded. The remaining missing LFQ values were imputed from a normal distribution (width, 0.3; down shift, 1.8 SD). Finally, for the whole data sets, two-sample *t*-test was used to assess statistical significance of protein abundances (*p* value < 0.01, S0 = 0.2).

## 5. Conclusions

Protein-based analysis for time since death estimation has emerged as a promising tool in recent years, although the spectrum of target proteins that are known to undergo predictable changes in early and mid-*post mortem* is still quite limited. Because of complex *post mortem* conditions and the influence of several factor, PMI estimation based on the behaviour of one or a few proteins is not pragmatic. In this study, we exploited a mass spectrometry-based proteomics approach to profile *post mortem* proteome alterations in skeletal muscle tissue and to characterize additional marker proteins for future use in PMI determination. In conclusion, despite the previously indicated limitations and the preliminary nature of our study, in this work we identified a total of nine potential markers, some of which had a decreasing trend and others an increasing one. The overall intersection of the data obtained from the variations in several markers, both in terms of increase and decrease, could be fundamental to estimate the PMI with increasingly accuracy and precision. This proteomic approach is highly functional for the characterization of reliable marker proteins useful in forensic practice. To obtain an improvement in the accuracy and overall precision in the PMI estimation it is necessary to characterize several markers even on different species, prolonged time intervals, or variable environmental conditions. In particular, investigations of the human *post mortem* proteome could contribute crucially to the characterization of important additional biomarkers.

## Data Availability

We shared raw data in supplementary material.

## Supplementary Materials

The following supporting information can be downloaded at https://www.mdpi.com/article/10.3390/ijms241914627/s1.
