# Supplementary material for: Forensic Proteomics for the Discovery of New post mortem Interval Biomarkers: A Preliminary Study
Source: Int J Mol Sci. 2023 Sep 27;24(19):14627. doi: 10.3390/ijms241914627 (PMC10572818; doi:10.3390/ijms241914627)
Supplement: Supplementary file 1 [file ijms-24-14627-s001.zip › ijms-2613466-supplementary.pdf]

LFQ intensity T0-1 LFQ intensity T0-2 LFQ intensity T0-3 LFQ intensity T1-1 LFQ intensity T1-2 LFQ intensity T1-3 LFQ intensity T2-1 LFQ intensity T2-2 LFQ intensity T2-3 LFQ intensity T3-1 LFQ intensity T3-2 LFQ intensity T3-3 C: Only identified by site C: Reverse C: Potential contaminant N: Peptides N: Razor + unique peptides N: Unique peptides N: Sequence coverage [%] N: Unique + razor sequence coverage [%] N: Unique sequence coverage [%] N: Mol. weight [kDa] N: Q-value N: Score N: Intensity N: MS/MS count T: Protein IDs T: Majority protein IDs T: id T: Fasta headers

22765000 44677000 18004000 0 9505100 0 0 0 0 0 0 0 6 6 6 20,9 20,9 20,9 44,836 0 11,551 176630000 12 A0A024BTL2;F1RG75 A0A024BTL2 0 tr|A0A024BTL2|A0A024BTL2_PIG Phosphorylase kinase OS=Sus scrofa OX=9823 GN=PHKG1 PE=1 SV=1

0 0 0 0 0 0 0 0 0 0 0 0 1 1 1 9,1 9,1 9,1 10,41 0 10,805 774040000 13 A0A075B7J0;A0A075B7H9;A0A075B7I5;A0A075B7I9 A0A075B7J0;A0A075B7H9;A0A075B7I5;A0A075B7I9 1 tr|A0A075B7J0|A0A075B7J0_PIG Ig-like domain-containing protein OS=Sus scrofa OX=9823 PE=4 SV=7;tr|A0A075B7H9|A0A075B7H9_PIG Ig-like domain-containing protein OS=Sus scrofa OX=9823 PE=4 SV=7;tr|A0A075B7I5|A0A075B7I5_PIG Ig-like domain-containing protein OS=

0 0 0 0 0 0 0 0 0 0 0 0 2 2 2 23,2 23,2 23,2 10,318 0 7,2136 93531000 6 A0A075B7I6;A0A287BMT4 A0A075B7I6;A0A287BMT4 2 tr|A0A075B7I6|A0A075B7I6_PIG Ig-like domain-containing protein OS=Sus scrofa OX=9823 PE=4 SV=3;tr|A0A287BMT4|A0A287BMT4_PIG Ig-like domain-containing protein OS=Sus scrofa OX=9823 PE=4 SV=2

348070000 147170000 304230000 373470000 89819000 677050000 400040000 115850000 311560000 444460000 167740000 348770000 4 4 4 51,4 51,4 51,4 11,003 0 83,947 6225900000 195 P01846;A0A286ZNH4;A0A287ARX1;A0A0A0MY58;A0A287A1M4;A0A287BP38 P01846;A0A286ZNH4;A0A287ARX1;A0A0A0MY58;A0A287A1M4;A0A287BP38 3 sp|P01846|LAC_PIG Ig lambda chain C region OS=Sus scrofa OX=9823 PE=1 SV=1;tr|A0A286ZNH4|A0A286ZNH4_PIG Uncharacterized protein OS=Sus scrofa OX=9823 PE=4 SV=1;tr|A0A287ARX1|A0A287ARX1_PIG Uncharacterized protein OS=Sus scrofa OX=9823 PE=4 SV=1;tr|A0A0A0MY

0 0 0 0 0 0 0 0 0 0 0 0 1 1 1 5 5 5 28,847 0 3,5148 66607000 7 A0A286ZUP1;A0A0D5BWD2 A0A286ZUP1;A0A0D5BWD2 4 tr|A0A286ZUP1|A0A286ZUP1_PIG Complement component 1 Q subcomponent-binding protein, mitochondrial OS=Sus scrofa OX=9823 GN=C1QBP PE=3 SV=1;tr|A0A0D5BWD2|A0A0D5BWD2_PIG Complement component 1 Q subcomponent-binding protein, mitochondrial OS=Sus scrofa OX=98

11751000 14220000 25185000 0 5898400 15799000 0 0 0 7735500 6870800 7324700 3 3 3 9,7 9,7 9,7 33,986 0 6,0309 244970000 11 A0A0H5ANC0 A0A0H5ANC0 5 tr|A0A0H5ANC0|A0A0H5ANC0_PIG Mimecan OS=Sus scrofa OX=9823 GN=OGN PE=1 SV=1

0 0 0 7669400 0 5038200 0 0 6020800 6271500 0 0 6 6 6 1,7 1,7 1,7 448,22 0 8,8843 106630000 10 A0A5G2QCX2;A0A286ZHV7;F1SU03;A0A5G2RG98;A0A287ATP0;A0A287AF47;A0A5G2QET5 A0A5G2QCX2;A0A286ZHV7;F1SU03;A0A5G2RG98;A0A287ATP0;A0A287AF47 7 tr|A0A5G2QCX2|A0A5G2QCX2_PIG Heparan sulfate proteoglycan 2 OS=Sus scrofa OX=9823 GN=HSPG2 PE=1 SV=1;tr|A0A286ZHV7|A0A286ZHV7_PIG Heparan sulfate proteoglycan 2 OS=Sus scrofa OX=9823 GN=HSPG2 PE=1 SV=1;tr|F1SU03|F1SU03_PIG Heparan sulfate proteoglycan 2 OS

0 0 5935200 0 0 0 0 0 0 0 0 8869400 2 2 2 17 17 17 17,306 0 3,4912 34869000 6 F1SK49;A0A286ZI26 F1SK49;A0A286ZI26 8 tr|F1SK49|F1SK49_PIG Plasminogen receptor with a C-terminal lysine OS=Sus scrofa OX=9823 GN=PLGRKT PE=1 SV=1;tr|A0A286ZI26|A0A286ZI26_PIG Plasminogen receptor with a C-terminal lysine OS=Sus scrofa OX=9823 GN=PLGRKT PE=1 SV=1

19174000 8293000 11885000 0 0 0 0 0 0 0 0 0 5 5 5 14 14 14 35,958 0 11,91 115710000 13 A0A287AEJ3;A0A481CJY0;A0A287AW32;A0A286ZI52;A0A286ZYY2;M3UZ37;A0A287BLE9;F1SA35;A0A5G2QCR1;F6Q9I4;A0A5G2QED5;A0A286ZPE3 A0A287AEJ3;A0A481CJY0;A0A287AW32;A0A286ZI52;A0A286ZYY2;M3UZ37 9 tr|A0A287AEJ3|A0A287AEJ3_PIG Heterogeneous nuclear ribonucleoproteins A2/B1 OS=Sus scrofa OX=9823 GN=HNRNPA2B1 PE=1 SV=2;tr|A0A481CJY0|A0A481CJY0_PIG Heterogeneous nuclear ribonucleoproteins A2/B1 OS=Sus scrofa OX=9823 GN=HNRNPA2B1 PE=1 SV=1;tr|A0A287AW32|

811530000 748630000 566860000 1422000000 631380000 503240000 1519900000 580060000 552160000 1129200000 665060000 339960000 18 18 16 48,3 48,3 44,8 43,328 0 195,6 60113000000 471 A0A286ZI55;A0A287BDV9;F1SHA2 A0A286ZI55;A0A287BDV9;F1SHA2 10 tr|A0A286ZI55|A0A286ZI55_PIG Glycerol-3-phosphate dehydrogenase [NAD(+)] OS=Sus scrofa OX=9823 GN=GPD1 PE=1 SV=2;tr|A0A287BDV9|A0A287BDV9_PIG Glycerol-3-phosphate dehydrogenase [NAD(+)] OS=Sus scrofa OX=9823 GN=GPD1 PE=1 SV=2;tr|F1SHA2|F1SHA2_PIG Glycerol-

12194000 14097000 14873000 27140000 10913000 17657000 20278000 5911400 17203000 23171000 9703100 22316000 7 7 7 9 9 9 79,944 0 14,885 551610000 44 A0A286ZI83;A0A286ZPA0;F1S1G8;A0A5G2RD43;A0A5G2RI05;F1S1G7 A0A286ZI83;A0A286ZPA0;F1S1G8;A0A5G2RD43;A0A5G2RI05 11 tr|A0A286ZI83|A0A286ZI83_PIG Amine oxidase OS=Sus scrofa OX=9823 GN=LOC100520329 PE=1 SV=2;tr|A0A286ZPA0|A0A286ZPA0_PIG Amine oxidase OS=Sus scrofa OX=9823 GN=LOC100520329 PE=1 SV=1;tr|F1S1G8|F1S1G8_PIG Amine oxidase OS=Sus scrofa OX=9823 GN=LOC110256000 P

112870000 13325000 209990000 129340000 91246000 172180000 83296000 49450000 60396000 58685000 81146000 213240000 10 10 10 39 39 39 21,646 0 33,252 5663700000 139 B3VCE8;A0A287BG25;A0A286ZI93;A0A287AMI5 B3VCE8;A0A287BG25;A0A286ZI93;A0A287AMI5 12 tr|B3VCE8|B3VCE8_PIG Troponin I OS=Sus scrofa OX=9823 GN=TNNI1 PE=1 SV=1;tr|A0A287BG25|A0A287BG25_PIG Troponin I1,

slow skeletal type OS=Sus scrofa OX=9823 GN=TNNI1 PE=1 SV=1;tr|A0A286ZI93|A0A286ZI93_PIG Troponin I1, slow skeletal type OS=Sus scrofa OX=982
40265000 15526000 20989000 22697000 45506000 11263000 40341000 63767000 29890000 42493000 40539000 27649000 10 10 10 23,4 23,4 23,4 47,875 0 33,426 1357500000 59 A0A286ZIE2 A0A286ZIE2 14 tr|A0A286ZIE2|A0A286ZIE2_PIG Uncharacterized protein OS=Sus scrofa OX=9823 PE=4 SV=1
192050000 270670000 725350000 166160000 657220000 747190000 135860000 278490000 627500000 188790000 670960000 1206500000 14 14 14 41,9 41,9 41,9 33,026 0 49,293 23562000000 247

A0A286ZIE8;F1RZQ6;I3LDJ6;A0A5G2R3N5;F2Z565;A0A287B804;A0A287AXS7;A0A5G2RDZ2;A0A287AVQ0;A0A5G2QRU7;Q6QRN9;A0A5G2R3W2;F1RRA6

A0A286ZIE8;F1RZQ6;I3LDJ6;A0A5G2R3N5;F2Z565 15 tr|A0A286ZIE8|A0A286ZIE8_PIG ADP/ATP translocase OS=Sus scrofa OX=9823 GN=SLC25A4 PE=1 SV=1;tr|F1RZQ6|F1RZQ6_PIG ADP/ATP translocase OS=Sus scrofa OX=9823 GN=SLC25A4 PE=1 SV=2;tr|I3LDJ6|I3LDJ6_PIG ADP/ATP translocase OS=Sus scrofa OX=9823 GN=SLC25A4 PE=1 S
30050000 29067000 20875000 42225000 0 30155000 30747000 37547000 26469000 34266000 19144000 25782000 4 4 4 20,3 20,3 20,3 38,781 0 49,614 607670000 51

A0A286ZIM7;A0A480ZBJ4;A0A5K1U2D0;A0A5G2QBC1;A0A287ACT9;A0A287BLL7

A0A286ZIM7;A0A480ZBJ4;A0A5K1U2D0;A0A5G2QBC1;A0A287ACT9 16

tr|A0A286ZIM7|A0A286ZIM7_PIG Protein NDRG2 OS=Sus scrofa OX=9823 GN=NDRG2 PE=1 SV=2;tr|A0A480ZBJ4|A0A480ZBJ4_PIG Protein NDRG2 OS=Sus scrofa OX=9823 GN=NDRG2 PE=1 SV=1;tr|A0A5K1U2D0|A0A5K1U2D0_PIG Protein NDRG2 OS=Sus scrofa OX=9823 GN=NDRG2 PE=1 SV=1;tr|A
77036000 53668000 36139000 56762000 47883000 40466000 74475000 27679000 35322000 64373000 28157000 0 2 2 2 8,4 8,4 8,4 30,609 0 10,181 650860000 16 A0A286ZIN0;A0A480M1B4;A0A287AAI4;F1RVC9

A0A286ZIN0;A0A480M1B4;A0A287AAI4;F1RVC9 17 tr|A0A286ZIN0|A0A286ZIN0_PIG Heterogeneous nuclear ribonucleoprotein D OS=Sus scrofa OX=9823 GN=HNRNPD PE=1 SV=2;tr|A0A480M1B4|A0A480M1B4_PIG Heterogeneous nuclear ribonucleoprotein D OS=Sus scrofa OX=9823 GN=HNRNPD PE=1 SV=1;tr|A0A287AAI4|A0A287AAI4_PIG H
0 0 0 0 0 0 0 0 0 0 0 0 1 1 1 5,4 5,4 5,4 25,131 0,0021906 2,2966 24957000 2 P0C596;F1S2G2;A0A286ZIS3

P0C596;F1S2G2;A0A286ZIS3 18 sp|P0C596|DUS29_PIG Dual specificity phosphatase 29 OS=Sus scrofa OX=9823 GN=DUSP29 PE=3 SV=1;tr|F1S2G2|F1S2G2_PIG Protein-serine/threonine phosphatase OS=Sus scrofa OX=9823 GN=DUSP29 PE=3 SV=2;tr|A0A286ZIS3|A0A286ZIS3_PIG Protein-serine/threonine phosphat
0 0 0 0 0 0 0 0 0 0 0 0 1 1 1 5,9 5,9 5,9 22,898 0,0011521 2,7436 12242000 1

A0A5G2R0F7;A0A5G2QKB6;A0A287BH49;A0A286ZIU2;A0A5G2Q7W2

A0A5G2R0F7;A0A5G2QKB6;A0A287BH49;A0A286ZIU2;A0A5G2Q7W2 19

tr|A0A5G2R0F7|A0A5G2R0F7_PIG Chloride intracellular channel protein OS=Sus scrofa OX=9823 GN=CLIC5 PE=1 SV=1;tr|A0A5G2QKB6|A0A5G2QKB6_PIG Chloride intracellular

channel protein OS=Sus scrofa OX=9823 GN=CLIC5 PE=1 SV=1;tr|A0A287BH49|A0A287BH49_PIG Chloride
65630000 0 0 0 0 0 71423000 0 0 49828000 0 0 2 2 2
6 6 6 30,814 0,0086455 1,3944 565020000 3 A0A286ZJ42;F1SAL3
A0A286ZJ42;F1SAL3 20 tr|A0A286ZJ42|A0A286ZJ42_PIG Sodium voltage-gated channel beta subunit 4 OS=Sus scrofa OX=9823 GN=SCN4B PE=1 SV=2;tr|F1SAL3|F1SAL3_PIG Sodium voltage-gated channel beta subunit 4 OS=Sus scrofa OX=9823 GN=SCN4B PE=1 SV=2
0 0 0 0 0 0 0 0 0 0 0 0 2 2 2 6,5 6,5 6,5
43,152 0 4,2975 58393000 9
A0A286ZZV5;F1RRS8;A0A5G2QNM5;A0A5G2RNE3;A0A287AAQ4;A0A287B830;A0A286ZXV0;A0A286ZJ75;A0A5G2RN10
A0A286ZZV5;F1RRS8;A0A5G2QNM5;A0A5G2RNE3;A0A287AAQ4;A0A287B830;A0A286ZXV0;A0A286ZJ75;A0A5G2RN10 21 tr|A0A286ZZV5|A0A286ZZV5_PIG Microtubule-associated protein OS=Sus scrofa OX=9823 GN=MAPT PE=1 SV=2;tr|F1RRS8|F1RRS8_PIG Microtubule-associated protein OS=Sus scrofa OX=9823 GN=MAPT PE=1 SV=4;tr|A0A5G2QNM5|A0A5G2QNM5_PIG Microtubule-associated protein OS=S
0 0 0 0 0 0 0 0 0 0 0 0 1 1 1 6,4 6,4 6,4
20,124 0,0031847 2,0478 9382000 1
A0A5G2QF65;A0A287BA60;A0A286ZJ82;F1RHM8
A0A5G2QF65;A0A287BA60;A0A286ZJ82;F1RHM8 22 tr|A0A5G2QF65|A0A5G2QF65_PIG RAN binding protein 1 OS=Sus scrofa OX=9823 GN=RANBP1 PE=1 SV=1;tr|A0A287BA60|A0A287BA60_PIG RAN binding protein 1 OS=Sus scrofa OX=9823 GN=RANBP1 PE=1 SV=1;tr|A0A286ZJ82|A0A286ZJ82_PIG RAN binding protein 1 OS=Sus scrofa OX=98
22562000 34696000 25160000 14786000 30884000 19118000 20014000 22463000 24025000 16288000
23871000 15216000 11 11 11 31,2 31,2 31,2 41,29 0 44,964
1141900000 76
A0A286ZJC3;F6Q8Z7;A0A287A8V1;F6Q625;A6M928;A0A5G2QX96;A0A5G2QMP7;A0A5G2R7R3;A0A286ZUD0;A0A5G2QFK1;A6M931;A0A5S8KY97
A0A286ZJC3;F6Q8Z7;A0A287A8V1;F6Q625;A6M928 23
tr|A0A286ZJC3|A0A286ZJC3_PIG RNA helicase OS=Sus scrofa OX=9823 GN=EIF4A2 PE=1 SV=1;tr|F6Q8Z7|F6Q8Z7_PIG RNA helicase OS=Sus scrofa OX=9823 GN=EIF4A2 PE=1 SV=2;tr|A0A287A8V1|A0A287A8V1_PIG RNA helicase OS=Sus scrofa OX=9823 GN=EIF4A1 PE=1 SV=1;tr|F6Q625|F6
19684000 14923000 9185900 29804000 8034300 13488000 24790000 12806000 21186000 30093000
12560000 9761600 16 16 12 26,7 26,7 20,3 67,758 0 30,977
1182700000 94
A0A287BHA1;F1SV14;A0A287AQS9;A0A287AB04;A0A286ZNJ8;A0A286ZYV1;A0A287AN48;A0A286ZJJ2;A0A5G2QE36;P26044;F1SB42
A0A287BHA1;F1SV14;A0A287AQS9;A0A287AB04;A0A286ZNJ8;A0A286ZYV1;A0A287AN48;A0A286ZJJ2;A0A5G2QE36;P26044 24 tr|A0A287BHA1|A0A287BHA1_PIG Radixin OS=Sus scrofa OX=9823 GN=RDX PE=1 SV=2;tr|F1SV14|F1SV14_PIG Radixin OS=Sus scrofa OX=9823

GN=RDX PE=1 SV=4;tr|A0A287AQS9|A0A287AQS9_PIG Radixin OS=Sus scrofa OX=9823 GN=RDX PE=1 SV=2;tr|A0A287AB04|A0A287AB04_PIG Radixin

0 0 0 0 13563000 12857000 0 0 7268800 0 0 16188000 4 4 4 14,5 14,5 14,5 34,425 0 5,8486 123340000 8 A0A5G2QW89;A0A286ZJJ4;A0A287ANK3;A0A5G2QQW0;F1SU78 A0A5G2QW89;A0A286ZJJ4;A0A287ANK3;A0A5G2QQW0;F1SU78 25 tr|A0A5G2QW89|A0A5G2QW89_PIG Calcium uniporter protein OS=Sus scrofa OX=9823 GN=MCU PE=1 SV=1;tr|A0A286ZJJ4|A0A286ZJJ4_PIG Calcium uniporter protein OS=Sus scrofa OX=9823 GN=MCU PE=1 SV=1;tr|A0A287ANK3|A0A287ANK3_PIG Calcium uniporter protein OS=Sus scrofa

280130000 62877000 208800000 243270000 82223000 502780000 289210000 64940000 196280000 238510000 92472000 255610000 4 4 4 11,5 11,5 11,5 38,847 0 11,333 5963500000 76 A0A287ATT2;A0A287B9G9;A0A287A2V7;A0A286ZJL9;A0A287B9X9 A0A287ATT2;A0A287B9G9;A0A287A2V7;A0A286ZJL9;A0A287B9X9 26 tr|A0A287ATT2|A0A287ATT2_PIG Uncharacterized protein OS=Sus scrofa OX=9823 PE=4 SV=2;tr|A0A287B9G9|A0A287B9G9_PIG Uncharacterized protein OS=Sus scrofa OX=9823 PE=4 SV=2;tr|A0A287A2V7|A0A287A2V7_PIG Uncharacterized protein OS=Sus scrofa OX=9823 PE=4 SV=2;t

0 0 0 0 0 0 0 0 0 0 0 0 3 3 3 27,3 27,3 27,3 10,069 0,0031513 1,9721 16760000 3 A0A286ZJN9 A0A286ZJN9 27 tr|A0A286ZJN9|A0A286ZJN9_PIG Acyl-CoA binding domain containing 7 OS=Sus scrofa OX=9823 GN=ACBD7 PE=4 SV=1

133560000 73414000 34754000 25906000 35329000 0 108090000 50979000 37318000 105840000 36105000 34951000 7 7 7 32,5 32,5 32,5 16,832 0 16,552 1368700000 28 A0A286ZUV8;A0A286ZJP7;A0A286ZK63;A0A287B356;A0A286ZPS5;A0A287B4A3 A0A286ZUV8;A0A286ZJP7;A0A286ZK63;A0A287B356;A0A286ZPS5;A0A287B4A3 28 tr|A0A286ZUV8|A0A286ZUV8_PIG Eukaryotic translation initiation factor 5A OS=Sus scrofa OX=9823 GN=EIF5A PE=1 SV=1;tr|A0A286ZJP7|A0A286ZJP7_PIG eIF-5a domain-containing protein OS=Sus scrofa OX=9823 GN=EIF5A PE=1 SV=1;tr|A0A286ZK63|A0A286ZK63_PIG eIF-5a dom

0 0 0 0 0 0 0 0 0 0 0 0 3 1 1 13,6 7,9 7,9 20,554 0,0011274 2,5245 67199000 2 A0A286ZJQ9;Q6RVA9 A0A286ZJQ9;Q6RVA9 29 tr|A0A286ZJQ9|A0A286ZJQ9_PIG Caveolin OS=Sus scrofa OX=9823 GN=CAV1 PE=3 SV=1;sp|Q6RVA9|CAV1_PIG Caveolin-1 OS=Sus scrofa OX=9823 GN=CAV1 PE=2 SV=1

0 0 0 0 0 0 0 0 0 0 0 0 1 1 1 5,5 5,5 5,5 20,546 0,0030364 1,7521 5750300 1 A0A286ZJU5 A0A286ZJU5 30 tr|A0A286ZJU5|A0A286ZJU5_PIG Malignant T-cell-amplified sequence OS=Sus scrofa OX=9823 GN=LOC100738684 PE=1 SV=1

385010000 303560000 219520000 383010000 311130000 267970000 387010000 302910000 360030000 437660000 355740000 193710000 42 42 42 52,7 52,7 52,7 96,758 0 323,31 55627000000 1031

A0A5G2QZI7;A0A286ZK06;F1RVL5;A0A287BSB6;A0A287A887

A0A5G2QZI7;A0A286ZK06;F1RVL5;A0A287BSB6;A0A287A887 31

tr|A0A5G2QZI7|A0A5G2QZI7_PIG Uncharacterized protein OS=Sus scrofa OX=9823 GN=MYOM2 PE=1 SV=1;tr|A0A286ZK06|A0A286ZK06_PIG Uncharacterized protein OS=Sus scrofa OX=9823 GN=MYOM2 PE=1 SV=1;tr|F1RVL5|F1RVL5_PIG Uncharacterized protein OS=Sus scrofa OX=9823 G

4202700 0 4230100 4871800 0 4194600 0 0 3943400 8452400 0 4442100

4 4 4 14,3 14,3 14,3 32,631 0 10,354 77749000 9

F1RXD4;A0A287AMF3;A0A286ZK35;A0A5G2QK40

F1RXD4;A0A287AMF3;A0A286ZK35;A0A5G2QK40 32 tr|F1RXD4|F1RXD4_PIG Regulator of microtubule dynamics 1 OS=Sus scrofa OX=9823 GN=RMDN1 PE=1 SV=2;tr|A0A287AMF3|A0A287AMF3_PIG Regulator of microtubule dynamics 1 OS=Sus scrofa OX=9823 GN=RMDN1 PE=1 SV=1;tr|A0A286ZK35|A0A286ZK35_PIG Regulator of microtubul

0 0 0 0 0 0 0 0 0 0 0 0 5 5 5 9,2 9,2 9,2

69,79 0 12,508 111470000 5

A0A287B895;A0A287BEB5;A0A286ZK64;A0A5G2R212;F1S8Q4;A0A287AZ56

A0A287B895;A0A287BEB5;A0A286ZK64;A0A5G2R212;F1S8Q4;A0A287AZ56 33

tr|A0A287B895|A0A287B895_PIG Atypical kinase COQ8A, mitochondrial OS=Sus scrofa OX=9823 GN=COQ8A PE=1 SV=2;tr|A0A287BEB5|A0A287BEB5_PIG Atypical kinase COQ8A, mitochondrial OS=Sus scrofa OX=9823 GN=COQ8A PE=1 SV=2;tr|A0A286ZK64|A0A286ZK64_PIG Atypical kina

13268000 23509000 18313000 0 19228000 5753800 9226900 15326000 13568000 0 14639000

10609000 13 5 4 22 9 7,1 76,327 0 32,339 267010000 17

A0A287BK26;A0A287A9T4;F1RQU2;A0A286ZR68;A0A287BDM6;A0A286ZKC5

A0A287BK26;A0A287A9T4;F1RQU2;A0A286ZR68;A0A287BDM6;A0A286ZKC5 34

tr|A0A287BK26|A0A287BK26_PIG HATPase_c domain-containing protein OS=Sus scrofa OX=9823 GN=HSP90AB1 PE=1 SV=1;tr|A0A287A9T4|A0A287A9T4_PIG HATPase_c domain-containing protein OS=Sus scrofa OX=9823 GN=HSP90AB1 PE=1 SV=1;tr|F1RQU2|F1RQU2_PIG HATPase_c domain-

0 0 0 0 0 0 0 0 0 0 0 0 2 1 1 3,6 2,2 2,2

64,299 0,0011429 2,6758 48887000 13

A0A5G2QU76;A0A286ZKD4;F1SF87;A0A5G2QVV8;A0A5G2QUC7;A0A287B665;A0A287ALB9;A0A5G2QD37

A0A5G2QU76;A0A286ZKD4;F1SF87;A0A5G2QVV8;A0A5G2QUC7;A0A287B665;A0A287ALB9;A0A5G2QD37 35 tr|A0A5G2QU76|A0A5G2QU76_PIG Solute carrier family 25 member 13 OS=Sus scrofa OX=9823 GN=SLC25A13 PE=1 SV=1;tr|A0A286ZKD4|A0A286ZKD4_PIG Solute carrier family 25 member 13 OS=Sus scrofa OX=9823 GN=SLC25A13 PE=1 SV=2;tr|F1SF87|F1SF87_PIG Solute carrier fami

2743900 14274000 5361100 0 3883800 3397500 0 0 8455200 0 4122100 0

4 4 4 17,1 17,1 17,1 32,956 0 12,824 100160000 17 F1SS86;A0A286ZKE7

F1SS86;A0A286ZKE7 36 tr|F1SS86|F1SS86_PIG Dehydrogenase/reductase 7C OS=Sus scrofa OX=9823 GN=DHRS7C PE=1 SV=3;tr|A0A286ZKE7|A0A286ZKE7_PIG Dehydrogenase/reductase 7C OS=Sus scrofa OX=9823 GN=DHRS7C PE=1 SV=1

0 0 0 6741800 0 0 13325000 0 0 11734000 0 0 2 2 2
12,9 12,9 12,9 22,283 0 9,9626 50473000 6 A0A286ZKG9 A0A286ZKG9 37
tr|A0A286ZKG9|A0A286ZKG9_PIG Peptidyl-prolyl cis-trans isomerase OS=Sus scrofa OX=9823 GN=PPIB PE=1 SV=1
197960000 1118400000 317040000 198890000 661950000 288110000 157020000
882700000 723010000 141070000 683610000 191980000 28 28
28 42,8 42,8 42,8 85,314 0 323,31 40862000000 809
A0A286ZKJ2;A0A286ZIJ9;A0A287B8N1;A0A287BCP5;A0A286ZQ47;A0A286ZU66;I3LCA1;Q2HYU2;A0A287B7N8;I3LFQ5;A0A287AGA4;A0A5G2QVT8;A0A286ZZL7;A0A5G2RD49;A0A287AB45;I3LK68;F1RX49
A0A286ZKJ2;A0A286ZIJ9;A0A287B8N1;A0A287BCP5;A0A286ZQ47;A0A286ZU66;I3LCA1;Q2HYU2;A0A287B7N8 39 tr|A0A286ZKJ2|A0A286ZKJ2_PIG ATP-dependent 6-phosphofructokinase OS=Sus scrofa OX=9823 GN=PFKM PE=1 SV=1;tr|A0A286ZIJ9|A0A286ZIJ9_PIG ATP-dependent 6-phosphofructokinase OS=Sus scrofa OX=9823 GN=PFKM PE=1 SV=1;tr|A0A287B8N1|A0A287B8N1_PIG ATP-dependent 6-p
0 0 9620900 6798100 8348900 6951800 5584300 0 0 6356800 5488200 8499900
4 4 4 9 9 9 59,475 0 12,593 127590000 16
A0A287BQR7;I3LJE2;A0A287BD54;A0A286ZKK0;F1RJT3;A0A5K1VI86;A0A287A9G1;A0A287BBD8;I3LEA3 A0A287BQR7;I3LJE2;A0A287BD54;A0A286ZKK0;F1RJT3 40
tr|A0A287BQR7|A0A287BQR7_PIG Dihydropyrimidinase-related protein 2 OS=Sus scrofa OX=9823 GN=DPYSL2 PE=1 SV=2;tr|I3LJE2|I3LJE2_PIG Dihydropyrimidinase-related protein 2 OS=Sus scrofa OX=9823 GN=DPYSL2 PE=1 SV=2;tr|A0A287BD54|A0A287BD54_PIG Dihydropyrimidina
0 0 0 0 0 0 0 0 0 0 0 0 5 2 2 12,6 4,7 4,7
54,686 0 4,1425 9038200 2
F6QB46;A0A5G2QSW9;A0A287BCC5;F6QB57;A0A286ZVC8;A0A287B7M7;A0A286ZKT1;A0A287BS43;A0A5K1UJ76;A0A5G2RLI5;A0A287AGQ9;A0A287BEM2;A0A286ZL03;A0A5G2QQK7;F1SSH3;A0A5G2R2W2;A0A286ZTV0;A0A5G2QK95;A0A5G2QVX7;A0A5G2QCQ9
F6QB46;A0A5G2QSW9;A0A287BCC5;F6QB57;A0A286ZVC8;A0A287B7M7;A0A286ZKT1;A0A287BS43;A0A5K1UJ76;A0A5G2RLI5;A0A287AGQ9;A0A287BEM2;A0A286ZL03;A0A5G2QQK7;F1SSH3;A0A5G2R2W2;A0A286ZTV0;A0A5G2QK95;A0A5G2QVX7;A0A5G2QCQ9 41
tr|F6QB46|F6QB46_PIG Calcium/calmodulin-dependent protein kinase OS=Sus scrofa OX=9823 GN=CAMK2G PE=1 SV=2;tr|A0A5G2QSW9|A0A5G2QSW9_PIG Calcium/calmodulin-dependent protein kinase OS=Sus scrofa OX=9823 GN=CAMK2G PE=1 SV=1;tr|A0A287BCC5|A0A287BCC5_PIG Calci
0 0 0 0 0 0 0 0 0 0 0 0 1 1 1 0,4 0,4 0,4
249,32 0,005005 1,6542 483490000 14 I3LPY4;A0A286ZKT7;A0A286ZSF3
I3LPY4;A0A286ZKT7;A0A286ZSF3 42 tr|I3LPY4|I3LPY4_PIG Uncharacterized protein OS=Sus scrofa OX=9823 GN=BRWD1 PE=4 SV=2;tr|A0A286ZKT7|A0A286ZKT7_PIG Uncharacterized protein OS=Sus scrofa OX=9823 GN=BRWD1 PE=4 SV=1;tr|A0A286ZSF3|A0A286ZSF3_PIG Uncharacterized protein OS=Sus scrofa OX=9823 G
0 0 0 0 0 0 0 0 0 0 0 0 2 2 2 8,1 8,1 8,1
41,152 0 4,2281 14452000 5

A0A287BNN6;A0A5G2QZ15;A0A5G2QRX4;F1SA44;A0A286ZKW0;A0A287BE36;A0A287B952;A0A287AFJ9;A0A5G2RHB2

A0A287BNN6;A0A5G2QZ15;A0A5G2QRX4;F1SA44;A0A286ZKW0;A0A287BE36;A0A287B952;A0A287AFJ9;A0A5G2RHB2 43 tr|A0A287BNN6|A0A287BNN6_PIG Molybdopterin molybdenumtransferase OS=Sus scrofa OX=9823 GN=GPHN PE=1 SV=2;tr|A0A5G2QZ15|A0A5G2QZ15_PIG Molybdopterin molybdenumtransferase OS=Sus scrofa OX=9823 GN=GPHN PE=1 SV=1;tr|A0A5G2QRX4|A0A5G2QRX4_PIG Molybdopterin mol
13847000 10243000 5265300 14066000 9603400 5556700 16228000 7350300 3730000 15604000
7597200 0 13 13 13 18,5 18,5 18,5 81,115 0 25,747 464050000
49 A0A286ZKW5;A0A286ZSD6;A0A287ANK5;A0A287ASK5

A0A286ZKW5;A0A286ZSD6;A0A287ANK5 44 tr|A0A286ZKW5|A0A286ZKW5_PIG Glycerol-3-phosphate dehydrogenase OS=Sus scrofa OX=9823 GN=GPD2 PE=1 SV=1;tr|A0A286ZSD6|A0A286ZSD6_PIG Glycerol-3-phosphate dehydrogenase OS=Sus scrofa OX=9823 GN=GPD2 PE=1 SV=2;tr|A0A287ANK5|A0A287ANK5_PIG Glycerol-3-phosphat
0 0 0 0 0 17292000 0 0 0 3945000 0 0 5 5 5 21,9
21,9 21,9 23,769 0 12,187 71700000 5

A0A286ZKY6;P81558;A0A287AYH7;A0A5G2QCT5

A0A286ZKY6;P81558;A0A287AYH7;A0A5G2QCT5 45 tr|A0A286ZKY6|A0A286ZKY6_PIG Myelin basic protein OS=Sus scrofa OX=9823 GN=MBP PE=1 SV=2;sp|P81558|MBP_PIG Myelin basic protein OS=Sus scrofa OX=9823 GN=MBP PE=1 SV=1;tr|A0A287AYH7|A0A287AYH7_PIG Myelin basic protein OS=Sus scrofa OX=9823 GN=MBP PE=1 SV=1;
0 0 0 0 0 0 0 0 0 0 0 0 2 2 2 14,9 14,9 14,9
20,92 0 6,4161 39808000 4 A0A286ZL26;F2Z546 A0A286ZL26;F2Z546 46

tr|A0A286ZL26|A0A286ZL26_PIG Ribosomal protein L19 OS=Sus scrofa OX=9823 GN=RPL19 PE=1 SV=1;tr|F2Z546|F2Z546_PIG 60S ribosomal protein L19 OS=Sus scrofa OX=9823 GN=RPL19 PE=1 SV=4
0 0 0 0 0 0 0 0 0 0 0 0 3 3 3 10 10 10
33,088 0 4,6551 9729900 4

A0A5G2RFU4;A0A5G2QIH9;A0A287A2F6;A0A287AJ82;F1RF45;A0A286ZL34;A0A5G2R052;A0A286ZQD2

A0A5G2RFU4;A0A5G2QIH9;A0A287A2F6;A0A287AJ82;F1RF45;A0A286ZL34;A0A5G2R052;A0A286ZQD2 47 tr|A0A5G2RFU4|A0A5G2RFU4_PIG Homer scaffold protein 1 OS=Sus scrofa OX=9823 GN=HOMER1 PE=1 SV=1;tr|A0A5G2QIH9|A0A5G2QIH9_PIG Homer scaffold protein 1 OS=Sus scrofa OX=9823 GN=HOMER1 PE=1 SV=1;tr|A0A287A2F6|A0A287A2F6_PIG Homer scaffold protein 1 OS=Sus scr
0 0 0 0 0 0 0 0 0 0 0 0 1 1 1 8,3 8,3 8,3
17,695 0 4,6855 0 1 A0A286ZL51 A0A286ZL51 48

tr|A0A286ZL51|A0A286ZL51_PIG Ribosomal protein L23a OS=Sus scrofa OX=9823 GN=RPL23A PE=1 SV=2
11815000 7693300 6525800 13358000 8073000 6121300 13619000 5978800 4709900 13405000
8810200 7009700 7 7 7 14,9 14,9 14,9 55,456 0 16,17
556990000 42

A0A287ADK3;A0A286ZL79;A0A287A399;A0A287AWM0;A0A5G2RFA2;A0A5G2QZJ5;F1RKM

1;A0A5G2R3L2;A0A5G2Q7X5;A0A5G2RFH5

A0A287ADK3;A0A286ZL79;A0A287A399;A0A287AWM0;A0A5G2RFA2;A0A5G2QZJ5;F1RKM1 49 tr|A0A287ADK3|A0A287ADK3_PIG Aldehyde dehydrogenase (NAD(+)) OS=Sus scrofa OX=9823 GN=ALDH7A1 PE=1 SV=1;tr|A0A286ZL79|A0A286ZL79_PIG Aldehyde dehydrogenase (NAD(+)) OS=Sus scrofa OX=9823 GN=ALDH7A1 PE=1 SV=2;tr|A0A287A399|A0A287A399_PIG Aldehyde dehydrogen

4952800 7157500 6376300 0 4005300 0 0 0 0 0 0 2990000 4

4 4 4,3 4,3 4,3 93,822 0 3,9232 75405000 9

I3LEW5;A0A286ZLH1;A0A287AN02;I3LQT3 I3LEW5;A0A286ZLH1;A0A287AN02;I3LQT3

50 tr|I3LEW5|I3LEW5_PIG 26S proteasome non-ATPase regulatory subunit 2 OS=Sus scrofa OX=9823 GN=PSMD2 PE=1 SV=2;tr|A0A286ZLH1|A0A286ZLH1_PIG 26S proteasome non-ATPase regulatory subunit 2 OS=Sus scrofa OX=9823 GN=PSMD2 PE=1 SV=1;tr|A0A287AN02|A0A287AN02_PIG 2

8045200 0 4329000 9189600 5861200 8399700 6520800 0 6337000 9434000 0 7587900

5 5 5 12,6 12,6 12,6 67,07 0 10,939 149130000 21

A0A5G2QYN3;A0A5G2R260;A0A286ZLL9 A0A5G2QYN3;A0A5G2R260;A0A286ZLL9

51 tr|A0A5G2QYN3|A0A5G2QYN3_PIG Synaptopodin OS=Sus scrofa OX=9823 GN=SYNPO PE=4 SV=1;tr|A0A5G2R260|A0A5G2R260_PIG Synaptopodin OS=Sus scrofa OX=9823 GN=SYNPO PE=4 SV=1;tr|A0A286ZLL9|A0A286ZLL9_PIG Synaptopodin OS=Sus scrofa OX=9823 GN=SYNPO PE=4 SV=2

0 0 0 0 0 0 0 0 0 0 0 0 1 1 1 3,9 3,9 3,9

43,226 0,0058997 1,5167 369680000 2

A0A5G2QZ43;I3LMJ8;A0A286ZLR1;I3LMM7;I3LC00;A0A5G2QFJ8;A0A480Q3H5;F1S432;A0A5G2RDC6

A0A5G2QZ43;I3LMJ8;A0A286ZLR1;I3LMM7;I3LC00;A0A5G2QFJ8;A0A480Q3H5;F1S432;A0A5G2RDC6 52 tr|A0A5G2QZ43|A0A5G2QZ43_PIG RNA helicase OS=Sus scrofa OX=9823 GN=DDX19B PE=1 SV=1;tr|I3LMJ8|I3LMJ8_PIG RNA helicase OS=Sus scrofa OX=9823 GN=DDX19B PE=1 SV=3;tr|A0A286ZLR1|A0A286ZLR1_PIG Uncharacterized protein OS=Sus scrofa OX=9823 GN=DDX19B PE=1 SV=1;t

0 0 0 0 0 0 0 0 0 0 0 0 2 2 2 1,3 1,3 1,3

181,7 0 3,1097 18758000 3

A0A287BLM4;A0A287BL81;A0A286ZLV2;A0A286ZMC0;A0A287BPF4;A0A286ZPQ1;A0A5G2R8G7;A0A287B163;A0A286ZVG7;I3LUR7

A0A287BLM4;A0A287BL81;A0A286ZLV2;A0A286ZMC0;A0A287BPF4;A0A286ZPQ1;A0A5G2R8G7;A0A287B163;A0A286ZVG7;I3LUR7 53 tr|A0A287BLM4|A0A287BLM4_PIG Collagen type VI alpha 3 chain OS=Sus scrofa OX=9823 GN=COL6A3 PE=1 SV=1;tr|A0A287BL81|A0A287BL81_PIG Collagen type VI alpha 3 chain OS=Sus scrofa OX=9823 GN=COL6A3 PE=1 SV=1;tr|A0A286ZLV2|A0A286ZLV2_PIG Collagen type VI alpha

0 3480700 0 0 0 0 5237000 0 0 0 0 0 2 2 2 13,9

13,9 13,9 20,312 0 12,804 23828000 5

A0A286ZLX7;A0A287AIF3;A0A287A9S3;A0A287AG25;F1RX99;A0A481BLE6;A0A5G2QTW6

A0A286ZLX7;A0A287AIF3;A0A287A9S3;A0A287AG25;F1RX99;A0A481BLE6;A0A5G2QTW6

54 tr|A0A286ZLX7|A0A286ZLX7_PIG Tumor protein D52 OS=Sus scrofa OX=9823

GN=TPD52 PE=1 SV=2;tr|A0A287AIF3|A0A287AIF3_PIG Tumor protein D52 OS=Sus scrofa OX=9823 GN=TPD52 PE=1 SV=1;tr|A0A287A9S3|A0A287A9S3_PIG Tumor protein D52 OS=Sus scrofa OX=9823 GN=TPD52 PE

95499000 55257000 36500000 36238000 0 0 53647000 22710000 25058000 46113000 21864000 20699000 4 4 4 19 19 19 22,104 0 30,594 976120000 28 I3LEV7;A0A286ZM34;A0A5G2QTP0 I3LEV7;A0A286ZM34 55 tr|I3LEV7|I3LEV7_PIG Uncharacterized protein OS=Sus scrofa OX=9823 GN=SH3BGR PE=1 SV=1;tr|A0A286ZM34|A0A286ZM34_PIG Uncharacterized protein OS=Sus scrofa OX=9823 GN=SH3BGR PE=1 SV=1

14663000 16176000 7181000 5869200 6229500 6234800 5922800 6348900 6520700 13025000 4520100 9712200 7 7 7 19,7 19,7 19,7 42,89 0 32,271 290160000 17 A0A287A702;A0A286ZM82;F1SMN1;A0A5G2QG35 A0A287A702;A0A286ZM82;F1SMN1 56 tr|A0A287A702|A0A287A702_PIG Calumenin OS=Sus scrofa OX=9823 GN=CALU PE=1 SV=1;tr|A0A286ZM82|A0A286ZM82_PIG Calumenin OS=Sus scrofa OX=9823 GN=CALU PE=1 SV=2;tr|F1SMN1|F1SMN1_PIG Calumenin OS=Sus scrofa OX=9823 GN=CALU PE=1 SV=4

8156800 0 9551400 9788400 0 10559000 9519200 0 6737500 12352000 0 7526200 8 6 5 20,9 15,1 11,5 43,902 0 8,071 245250000 18 A0A287AGW0;A0A287B2Z5;A0A286ZTU7;A0A287AJE3;A0A286ZM84;A0A286ZYZ5;A0A286ZVN0 A0A287AGW0;A0A287B2Z5;A0A286ZTU7;A0A287AJE3;A0A286ZM84;A0A286ZYZ5 57 tr|A0A287AGW0|A0A287AGW0_PIG SERPIN domain-containing protein OS=Sus scrofa OX=9823 GN=LOC106504547 PE=1 SV=1;tr|A0A287B2Z5|A0A287B2Z5_PIG SERPIN domain-containing protein OS=Sus scrofa OX=9823 GN=LOC106504547 PE=1 SV=1;tr|A0A286ZTU7|A0A286ZTU7_PIG SERPIN

0 0 0 0 0 0 0 0 0 0 0 0 1 1 1 2,1 2,1 2,1 63,972 0,0030738 1,8191 23537000 1 A0A286ZMD2 A0A286ZMD2 58 tr|A0A286ZMD2|A0A286ZMD2_PIG Atlastin GTPase 2 OS=Sus scrofa OX=9823 GN=ATL2 PE=1 SV=1

18985000 14045000 15562000 0 4931500 0 6580800 5815000 4791400 6040200 5073100 5029600 4 4 4 10,4 10,4 10,4 65,418 0 22,235 209920000 17 A0A287B2Z1;A0A286ZMD6;A0A287B082;A0A287A190;A0A287ANW2;F1RWK8;A0A5G2Q732;A0A286ZP52;A0A5G2R3P3;A0A287AL78;A0A287BIE3;A0A5G2R637;A0A287BSZ0;A0A287A6B3;A0A287AJJ7;F1RWA1;A0A287B2G2;A0A287AHB4;A0A287AHZ5;A0A5G2Q8J4;A0A287ANL1 A0A287B2Z1;A0A286ZMD6;A0A287B082;A0A287A190;A0A287ANW2;F1RWK8 59 tr|A0A287B2Z1|A0A287B2Z1_PIG Calcium channel voltage-dependent subunit beta 1 OS=Sus scrofa OX=9823 GN=CACNB1 PE=3 SV=1;tr|A0A286ZMD6|A0A286ZMD6_PIG Calcium channel voltage-dependent subunit beta 1 OS=Sus scrofa OX=9823 GN=CACNB1 PE=3 SV=2;tr|A0A287B082|A0

0 0 0 0 0 0 0 0 0 0 0 0 2 1 1 4,4 2,3 2,3 60,496 0,003148 1,9666 21306000 1 A0A286ZMH6;A0A5G2R092;A0A5G2RA07;A0A287BRK6 A0A286ZMH6;A0A5G2R092;A0A5G2RA07;A0A287BRK6 60 tr|A0A286ZMH6|A0A286ZMH6_PIG Uncharacterized protein OS=Sus scrofa OX=9823

GN=LOC110259328 PE=1 SV=1;tr|A0A5G2R092|A0A5G2R092_PIG Uncharacterized protein OS=Sus scrofa OX=9823 GN=LOC110259328 PE=3 SV=1;tr|A0A5G2RA07|A0A5G2RA07_PIG Uncharacterized protein O
0 0 0 0 0 0 0 0 0 0 0 0 2 2 2 6,6 6,6 6,6
35,699 0,0011655 2,8366 11377000 4 A0A286ZMQ7 A0A286ZMQ7 61
tr|A0A286ZMQ7|A0A286ZMQ7_PIG RAB12, member RAS oncogene family OS=Sus scrofa OX=9823 GN=RAB12 PE=4 SV=2
0 0 0 0 0 0 0 0 0 0 0 0 1 1 1 4,2 4,2 4,2
26,708 0 4,7149 125770000 6 A0A5G2R857;A0A286ZMT0;F1SB33
A0A5G2R857;A0A286ZMT0;F1SB33 62 tr|A0A5G2R857|A0A5G2R857_PIG MARVEL domain-containing protein OS=Sus scrofa OX=9823 GN=SYPL1 PE=1 SV=1;tr|A0A286ZMT0|A0A286ZMT0_PIG MARVEL domain-containing protein OS=Sus scrofa OX=9823 GN=SYPL1 PE=1 SV=1;tr|F1SB33|F1SB33_PIG MARVEL domain-containing prot
0 0 0 0 0 0 0 0 0 0 0 0 2 1 1 7,8 4,4 4,4
33,8 0,0031185 1,9072 12401000 1
A0A5G2QVL8;F1SFQ4;A0A5G2R731;A0A287BBM1;A0A5G2Q7Y5;A0A287BHL0;A0A286ZMU5;A0A480Q0J7;I3LRF8;A0A5G2QGE1;A0A5G2R625;A0A5G2QU32;A0A5G2REH8;A0A5G2QDP8
A0A5G2QVL8;F1SFQ4;A0A5G2R731;A0A287BBM1;A0A5G2Q7Y5;A0A287BHL0;A0A286ZMU5;A0A480Q0J7;I3LRF8;A0A5G2QGE1;A0A5G2R625;A0A5G2QU32;A0A5G2REH8;A0A5G2QDP8
63 tr|A0A5G2QVL8|A0A5G2QVL8_PIG Uncharacterized protein OS=Sus scrofa OX=9823 GN=PCBP2 PE=1 SV=1;tr|F1SFQ4|F1SFQ4_PIG Uncharacterized protein OS=Sus scrofa OX=9823 GN=PCBP2 PE=1 SV=3;tr|A0A5G2R731|A0A5G2R731_PIG Uncharacterized protein OS=Sus scrofa OX=9823 G
0 0 6171200 0 0 0 0 0 0 0 0 5493800 4 4 4 6,5
6,5 6,5 76,589 0 5,1206 41700000 7 A0A286ZMU8;A0A5G2R2M6;I3LFR2
A0A286ZMU8;A0A5G2R2M6;I3LFR2 64 tr|A0A286ZMU8|A0A286ZMU8_PIG Cullin 5 OS=Sus scrofa OX=9823 GN=CUL5 PE=1 SV=2;tr|A0A5G2R2M6|A0A5G2R2M6_PIG Cullin 5 OS=Sus scrofa OX=9823 GN=CUL5 PE=1 SV=1;tr|I3LFR2|I3LFR2_PIG Cullin-5 OS=Sus scrofa OX=9823 GN=CUL5 PE=1 SV=1
1200600000 3857200000 2098600000 508210000 3116600000 2025500000 503130000
3244000000 2813600000 463960000 2968100000 1225200000 52 52
47 53,1 53,1 47,9 96,874 0 323,31 293330000000 2221
A0A286ZMZ9;A0A5G2QU59;F1RQQ8;A0A287B6I2;A0A5G2RE07;A0A287AL55;F1RQQ7;A0A5G2QWP3;A0A287AT94;A0A5G2QW02;A0A5G2QYQ7
A0A286ZMZ9;A0A5G2QU59;F1RQQ8;A0A287B6I2;A0A5G2RE07 65
tr|A0A286ZMZ9|A0A286ZMZ9_PIG Alpha-1,4 glucan phosphorylase OS=Sus scrofa OX=9823 GN=PYGM PE=1 SV=1;tr|A0A5G2QU59|A0A5G2QU59_PIG Alpha-1,4 glucan phosphorylase OS=Sus scrofa OX=9823 GN=PYGM PE=1 SV=1;tr|F1RQQ8|F1RQQ8_PIG Alpha-1,4 glucan phosphorylase OS=S
0 0 0 0 0 0 0 0 0 0 0 0 2 2 2 2,5 2,5 2,5
98,743 0,0030896 1,8588 8067200 2
A0A5G2QEW1;A0A287BGD6;P79263;A0A286ZN24;F1SH92;A0A5G2R7M3;A0A5G2QN24;A0A286ZT99;A0A5G2QU18

A0A5G2QEW1;A0A287BGD6;P79263;A0A286ZN24;F1SH92;A0A5G2R7M3;A0A5G2QN24;A0A286ZT99;A0A5G2QU18 66 tr|A0A5G2QEW1|A0A5G2QEW1_PIG Inter-alpha-trypsin inhibitor heavy chain H4 OS=Sus scrofa OX=9823 GN=ITIH4 PE=1 SV=1;tr|A0A287BGD6|A0A287BGD6_PIG Inter-alpha-trypsin inhibitor heavy chain H4 OS=Sus scrofa OX=9823 GN=ITIH4 PE=1 SV=2;sp|P79263|ITIH4_PIG Inter-

0 0 0 0 0 0 0 0 0 0 0 0 1 1 1 3 3 3

33,62 0 2,9495 2636200000 2 A0A286ZN44;F1SJ42;A0A5G2QSG3;A0A5G2QC04

A0A286ZN44;F1SJ42;A0A5G2QSG3;A0A5G2QC04 67 tr|A0A286ZN44|A0A286ZN44_PIG Olfactory receptor OS=Sus scrofa OX=9823 GN=LOC100523356 PE=3 SV=2;tr|F1SJ42|F1SJ42_PIG Olfactory receptor OS=Sus scrofa OX=9823 PE=3 SV=3;tr|A0A5G2QSG3|A0A5G2QSG3_PIG Olfactory receptor OS=Sus scrofa OX=9823 GN=LOC100523167 PE

15550000 15387000 10426000 19334000 14267000 17830000 23600000 11492000 14415000 14157000 0

10341000 3 3 3 16,6 16,6 16,6 21,62 0 4,7759 303400000 17

A0A5G2R3I7;A0A286ZN52 A0A5G2R3I7;A0A286ZN52 68

tr|A0A5G2R3I7|A0A5G2R3I7_PIG Proteasome subunit beta OS=Sus scrofa OX=9823 GN=PSMB3 PE=1 SV=1;tr|A0A286ZN52|A0A286ZN52_PIG Proteasome subunit beta OS=Sus scrofa OX=9823 GN=PSMB3 PE=1 SV=1

0 0 0 0 0 0 0 0 0 0 0 0 1 1 1 4 4 4

37,681 0 4,7256 0 1

A0A5G2QA32;A0A286ZN59;A0A287B7C9;F1RHU6;A0A286ZP03;A0A287BTE0

A0A5G2QA32;A0A286ZN59;A0A287B7C9;F1RHU6;A0A286ZP03;A0A287BTE0 69

tr|A0A5G2QA32|A0A5G2QA32_PIG Protein arginine methyltransferase 1 OS=Sus scrofa OX=9823 GN=PRMT1 PE=1 SV=1;tr|A0A286ZN59|A0A286ZN59_PIG Protein arginine methyltransferase 1 OS=Sus scrofa OX=9823 GN=PRMT1 PE=1 SV=2;tr|A0A287B7C9|A0A287B7C9_PIG Protein argin

9988700 0 0 11305000 0 0 10878000 0 0 9779800 0 0 8 8

8 15,1 15,1 15,1 73,831 0 15,654 235140000 23

A0A288CFT7;A0A286ZQD7;P12675;A0A286ZNA7;A0A287A526;F6PU32

A0A288CFT7;A0A286ZQD7;P12675;A0A286ZNA7;A0A287A526;F6PU32 70

tr|A0A288CFT7|A0A288CFT7_PIG Calpain inhibitor OS=Sus scrofa OX=9823 GN=CAST PE=3 SV=2;tr|A0A286ZQD7|A0A286ZQD7_PIG Calpain inhibitor OS=Sus scrofa OX=9823 GN=CAST PE=3 SV=2;sp|P12675|ICAL_PIG Calpastatin OS=Sus scrofa OX=9823 GN=CAST PE=1 SV=1;tr|A0A286ZN

54774000 27226000 59445000 77122000 32152000 83357000 78695000 17572000 38038000 64554000

28475000 64010000 7 7 5 27,7 27,7 21 25,049 0 16,791

2797600000 67 A0A286ZND5;F1S3U9;A0A5K1VJP7 A0A286ZND5;F1S3U9 71

tr|A0A286ZND5|A0A286ZND5_PIG Peroxiredoxin-1 OS=Sus scrofa OX=9823 GN=PRDX1 PE=1 SV=1;tr|F1S3U9|F1S3U9_PIG Peroxiredoxin-1 OS=Sus scrofa OX=9823 GN=PRDX1 PE=1 SV=3

0 9499200 28458000 0 9719300 9794300 0 8374000 11177000 7420600 9577200 12250000

3 3 3 8,3 8,3 8,3 39,366 0 5,2387 250860000 6

A0A287AGH7;A0A287ACC3;A0A287A4E7;P36887;P05383;F1SD45;A0A286ZUD6;A0A286ZNG3;A0A287AGL2;A0A287BQL5

A0A287AGH7;A0A287ACC3;A0A287A4E7;P36887;P05383;F1SD45;A0A286ZUD6;A0A286ZNG

3;A0A287AGL2;A0A287BQL5 72 tr|A0A287AGH7|A0A287AGH7_PIG cAMP-dependent protein kinase OS=Sus scrofa OX=9823 GN=PRKACB PE=3 SV=1;tr|A0A287ACC3|A0A287ACC3_PIG cAMP-dependent protein kinase OS=Sus scrofa OX=9823 GN=PRKACA PE=3 SV=2;tr|A0A287A4E7|A0A287A4E7_PIG cAMP-dependent protein ki
15449000 10945000 36349000 23642000 24041000 30282000 16638000 0 34527000 42291000 23582000 76400000 6 6 6 28,4 28,4 28,4 30,201 0 16,954 937570000 54

A0A286ZNN4 A0A286ZNN4 73 tr|A0A286ZNN4|A0A286ZNN4_PIG Complex I-30kD OS=Sus scrofa OX=9823 GN=NDUFS3 PE=1 SV=1
0 0 0 0 0 0 0 0 0 0 0 0 1 1 1 4,9 4,9 4,9 26,296 0,0078125 1,4857 4700300 3 A0A286ZNV0;I3LUD1 A0A286ZNV0;I3LUD1

74 tr|A0A286ZNV0|A0A286ZNV0_PIG Superoxide dismutase [Cu-Zn] OS=Sus scrofa OX=9823 GN=SOD3 PE=1 SV=1;tr|I3LUD1|I3LUD1_PIG Superoxide dismutase [Cu-Zn] OS=Sus scrofa OX=9823 GN=SOD3 PE=1 SV=2
366310000 320480000 293510000 114210000 275720000 236840000 229010000 136630000 226580000 156420000 146080000 154460000 14 14 6 26 26 11,5 53,636 0 46,706 14823000000 196

A0A5G2QNU4;A0A286ZNV2;A0A5K1VC16;A0A5G2QGC2;A0A5G2QBA4;A0A5G2QN81;F1STM4;A0A5G2RBZ6;A0A5G2RFX0;A0A5G2QCL5;A0A286ZIH3;A0A5G2RHB8;A0A5G2Q9Y7

A0A5G2QNU4;A0A286ZNV2;A0A5K1VC16;A0A5G2QGC2;A0A5G2QBA4;A0A5G2QN81;F1STM4;A0A5G2RBZ6;A0A5G2RFX0;A0A5G2QCL5 75 tr|A0A5G2QNU4|A0A5G2QNU4_PIG Elongation factor 1-alpha OS=Sus scrofa OX=9823 GN=EEF1A2 PE=3 SV=1;tr|A0A286ZNV2|A0A286ZNV2_PIG Elongation factor 1-alpha 2 OS=Sus scrofa OX=9823 GN=EEF1A2 PE=3 SV=1;tr|A0A5K1VC16|A0A5K1VC16_PIG Elongation factor 1-alpha OS=Su
0 0 0 0 0 0 0 0 0 0 0 0 2 2 2 3 3 3 73,277 0,004985 1,6213 26460000 3

A0A5G2QYF7;A0A286ZXZ1;A0A5G2RDH1;A0A5G2RAQ4;A0A5G2QRI6;A0A286ZNW1

A0A5G2QYF7;A0A286ZXZ1;A0A5G2RDH1;A0A5G2RAQ4;A0A5G2QRI6;A0A286ZNW1 76

tr|A0A5G2QYF7|A0A5G2QYF7_PIG EMAP like 2 OS=Sus scrofa OX=9823 GN=EML2 PE=1 SV=1;tr|A0A286ZXZ1|A0A286ZXZ1_PIG EMAP like 2 OS=Sus scrofa OX=9823 GN=EML2 PE=1 SV=2;tr|A0A5G2RDH1|A0A5G2RDH1_PIG EMAP like 2 OS=Sus scrofa OX=9823 GN=EML2 PE=1 SV=1;tr|A0A5G2RAQ4
2975100000 8113800000 2765200000 3723100000 5077400000 2961200000 2989000000 7895000000 5026700000 2062500000 5752000000 2172600000 19 19 18 45,2 45,2 41,6 36,618 0 198,33 290470000000 2144

P00339;A0A287AFC1;A0A287B2H7;A0A5S6GD60;A0A286ZNX3;A0A286ZXT7;A0A5G2QUB8;A0A5G2QSJ4;A0A5G2QLD7;Q9TSX5;F1SFX0

P00339;A0A287AFC1;A0A287B2H7;A0A5S6GD60;A0A286ZNX3;A0A286ZXT7 77

sp|P00339|LDHA_PIG L-lactate dehydrogenase A chain OS=Sus scrofa OX=9823 GN=LDHA PE=1 SV=3;tr|A0A287AFC1|A0A287AFC1_PIG L-lactate dehydrogenase OS=Sus scrofa OX=9823 GN=LDHA PE=3 SV=2;tr|A0A287B2H7|A0A287B2H7_PIG L-lactate dehydrogenase OS=Sus scrofa OX=98
14126000 15461000 21214000 0 4007400 3943800 0 0 7374200 0 0 5041100 6 6 3 17,8 17,8 8,9 48,895 0 9,2133 214610000 20

F2Z5S8;A0A286ZNY1;A0A5G2R655;F1SR80;A0A5G2QX54;A0A287BMB7;A0A287B5H5

F2Z5S8;A0A286ZNY1;A0A5G2R655;F1SR80;A0A5G2QX54;A0A287BMB7;A0A287B5H5 78

tr|F2Z5S8|F2Z5S8_PIG Tubulin alpha chain OS=Sus scrofa OX=9823 GN=TUBA4A PE=1 SV=2;tr|A0A286ZNY1|A0A286ZNY1_PIG Tubulin alpha chain OS=Sus scrofa OX=9823 GN=TUBA4A PE=1 SV=1;tr|A0A5G2R655|A0A5G2R655_PIG Tubulin alpha 4a OS=Sus scrofa OX=9823 GN=TUBA4A PE=1

0 0 0 0 0 0 0 0 4656900 4113200 0 0 2 2 2 9,2

9,2 9,2 35,082 0 4,303 32755000 4

A0A5G2QTV8;F1SN52;A0A5G2REV7;A0A287BBF3;A0A286ZP38;A0A287B9G2;A0A5G2QUD2;A0A5G2QLF4

A0A5G2QTV8;F1SN52;A0A5G2REV7;A0A287BBF3;A0A286ZP38;A0A287B9G2;A0A5G2QUD2;A0A5G2QLF4 79 tr|A0A5G2QTV8|A0A5G2QTV8_PIG 3-hydroxyisobutyryl-CoA hydrolase, mitochondrial OS=Sus scrofa OX=9823 GN=HIBCH PE=1 SV=1;tr|F1SN52|F1SN52_PIG 3-hydroxyisobutyryl-CoA hydrolase, mitochondrial OS=Sus scrofa OX=9823 GN=HIBCH PE=1 SV=4;tr|A0A5G2REV7|A0A5G2REV7_P

16784000 6709800 8010500 13397000 6591600 8552700 13018000 0 11012000 15336000 6822800

8197900 4 4 2 22,2 22,2 11,8 22,931 0 10,805 215700000 32

A0A5G2RMX2;A0A480T5S7;A0A286ZP69;A0A5G2RMS7;F1SPG0;A0A5G2R382;A0A287BQX8

A0A5G2RMX2;A0A480T5S7;A0A286ZP69;A0A5G2RMS7;F1SPG0;A0A5G2R382;A0A287BQX8

80 tr|A0A5G2RMX2|A0A5G2RMX2_PIG RAB7A, member RAS oncogene family OS=Sus scrofa OX=9823 GN=RAB7A PE=1 SV=1;tr|A0A480T5S7|A0A480T5S7_PIG RAB7A, member RAS oncogene family OS=Sus scrofa OX=9823 GN=RAB7A PE=1 SV=1;tr|A0A286ZP69|A0A286ZP69_PIG RAB7A, member RAS o

0 0 0 0 0 0 0 0 0 0 0 0 1 1 1 1,1 1,1 1,1

105,62 0,0086124 1,3787 4122100 2 A0A287AVR0;A0A286ZP85

A0A287AVR0;A0A286ZP85 81 tr|A0A287AVR0|A0A287AVR0_PIG Eukaryotic translation initiation factor 3 subunit C OS=Sus scrofa OX=9823 GN=LOC110260088 PE=3 SV=1;tr|A0A286ZP85|A0A286ZP85_PIG Eukaryotic translation initiation factor 3 subunit C OS=Sus scrofa OX=9823 GN=LOC110260088 PE=3 S

0 0 0 0 0 0 8411400 0 0 8383100 0 0 2 2 2 8,4

8,4 8,4 42,654 0 14,53 44351000 5

A0A286ZRK0;A0A286ZPG2;A0A287BPY7;A0A5G2R1G6;A0A287B444;A0A286ZSN6

A0A286ZRK0;A0A286ZPG2;A0A287BPY7;A0A5G2R1G6;A0A287B444;A0A286ZSN6 82

tr|A0A286ZRK0|A0A286ZRK0_PIG Uncharacterized protein OS=Sus scrofa OX=9823 GN=LOC100622780 PE=1 SV=2;tr|A0A286ZPG2|A0A286ZPG2_PIG Uncharacterized protein OS=Sus scrofa OX=9823 GN=TMPO PE=3 SV=2;tr|A0A287BPY7|A0A287BPY7_PIG Uncharacterized protein OS=Sus sc

0 0 0 0 0 0 0 0 0 0 0 0 1 1 1 9,3 9,3 9,3

13,635 0,0030519 1,7888 250690000 9

A0A5G2QNJ5;A0A287ABE6;A0A5G2R1D9;A0A286ZPH4;Q06AA9;F1S105;A0A288CFV1

A0A5G2QNJ5;A0A287ABE6;A0A5G2R1D9;A0A286ZPH4;Q06AA9;F1S105;A0A288CFV1 83

tr|A0A5G2QNJ5|A0A5G2QNJ5_PIG Ubiquitin-conjugating enzyme E2 D2 OS=Sus scrofa OX=9823 GN=UBE2D2 PE=3 SV=1;tr|A0A287ABE6|A0A287ABE6_PIG UBC core domain-containing

protein OS=Sus scrofa OX=9823 PE=4 SV=1;tr|A0A5G2R1D9|A0A5G2R1D9_PIG Ubiquitin-conjugating enz
0 0 0 0 0 0 0 0 0 0 0 0 1 1 1 2,8 2,8 2,8
55,794 0,0030334 1,7517 85955000 5 A0A286ZPP7;A0A286ZWZ8
A0A286ZPP7;A0A286ZWZ884 tr|A0A286ZPP7|A0A286ZPP7_PIG RAR related orphan receptor C OS=Sus scrofa OX=9823 GN=RORC PE=3 SV=2;tr|A0A286ZWZ8|A0A286ZWZ8_PIG RAR related orphan receptor C OS=Sus scrofa OX=9823 GN=RORC PE=3 SV=1
27457000 18276000 36009000 51591000 23753000 31180000 32366000 18348000 43263000 57196000
29229000 71177000 7 7 7 32,9 32,9 32,9 27,406 0 55,564
1170000000 94 A0A287BG40;A0A286ZPT6;F1SM98 A0A287BG40;A0A286ZPT6;F1SM98
85 tr|A0A287BG40|A0A287BG40_PIG NADH dehydrogenase [ubiquinone] flavoprotein 2, mitochondrial OS=Sus scrofa OX=9823 GN=NDUFV2 PE=1 SV=1;tr|A0A286ZPT6|A0A286ZPT6_PIG NADH dehydrogenase [ubiquinone] flavoprotein 2, mitochondrial OS=Sus scrofa OX=9823 GN=NDUFV2
17253000 0 57923000 25893000 34361000 66986000 18606000 10079000 19618000 23285000 19667000
70707000 40 19 7 21,5 11,6 4,5 220,85 0 137,06 1165400000 72
A0A286ZPX6;P79293;A0A286ZS93;A0A286ZPQ9;A0A287AGU3
A0A286ZPX6;P79293;A0A286ZS93;A0A286ZPQ9;A0A287AGU3 86
tr|A0A286ZPX6|A0A286ZPX6_PIG Uncharacterized protein OS=Sus scrofa OX=9823 GN=LOC100736765 PE=1 SV=2;sp|P79293|MYH7_PIG Myosin-7 OS=Sus scrofa OX=9823 GN=MYH7 PE=1 SV=2;tr|A0A286ZS93|A0A286ZS93_PIG Uncharacterized protein OS=Sus scrofa OX=9823 GN=LOC100736
25381000 0 0 27044000 0 13392000 0 0 0 13787000 0 0 17 4
4 40,5 6 6 129,69 0 4,8599 192840000 12 A0A286ZPY1 A0A286ZPY1 87
tr|A0A286ZPY1|A0A286ZPY1_PIG AHNAK nucleoprotein OS=Sus scrofa OX=9823 GN=AHNAK PE=1 SV=2
0 2152100 3636600 1755300 1977800 1984400 0 0 2528100 0 0 2024300
2 2 2 12,5 12,5 12,5 17,814 0,0049603 1,5945 32976000 3
A0A5G2REK8;B5APU9;A0A5G2QG21;A0A286ZPY9;A0A5G2QPW1;A0A5G2QAE1;A0A5K1VDV6;A0A286ZXR9
A0A5G2REK8;B5APU9;A0A5G2QG21;A0A286ZPY9;A0A5G2QPW1;A0A5G2QAE1;A0A5K1VDV6;A0A286ZXR9 88 tr|A0A5G2REK8|A0A5G2REK8_PIG Uncharacterized protein OS=Sus scrofa OX=9823 GN=ARPC4 PE=1 SV=1;tr|B5APU9|B5APU9_PIG Actin-related protein 2/3 complex subunit 4 OS=Sus scrofa OX=9823 GN=ARPC4 PE=1 SV=1;tr|A0A5G2QG21|A0A5G2QG21_PIG Actin-related protein 2/3 c
5093800 21159000 39911000 18382000 45089000 44348000 10911000 24376000 40570000 13610000
45321000 52339000 16 16 15 26,2 26,2 25 72,422 0 63,801
2776100000 137
A0A5G2QBW2;A0A5G2QQF8;F1S086;A0A286ZQ07;A0A5G2QY62;A0A5G2QQP2;I3L8T2;I3L614;A0A286ZRZ5
A0A5G2QBW2;A0A5G2QQF8;F1S086;A0A286ZQ07;A0A5G2QY62;A0A5G2QQP2;I3L8T2;I3L614 89 tr|A0A5G2QBW2|A0A5G2QBW2_PIG Solute carrier family 25 member 12 OS=Sus scrofa OX=9823 GN=SLC25A12 PE=1 SV=1;tr|A0A5G2QQF8|A0A5G2QQF8_PIG Solute carrier family 25

member 12 OS=Sus scrofa OX=9823 GN=SLC25A12 PE=1 SV=1;tr|F1S086|F1S086_PIG Calcium-binding mit
21788000 26264000 0 11402000 20969000 0 0 21691000 0 0 16050000 0
3 3 3 12,7 12,7 12,7 29,939 0 3,9371 332110000 7 A0A286ZQ31
A0A286ZQ31 90 tr|A0A286ZQ31|A0A286ZQ31_PIG Phosphoglycerate mutase OS=Sus scrofa OX=9823 GN=BPGM PE=3 SV=1
0 0 0 0 0 0 0 0 0 0 0 0 15 1 1 13,2 2,2 2,2
104,7 0 33,317 1638700000 6 F1RGR0;A0A286ZVJ3;O77696;A0A286ZQ54
F1RGR0;A0A286ZVJ3;O77696;A0A286ZQ54 91 tr|F1RGR0|F1RGR0_PIG Calcium-transporting ATPase OS=Sus scrofa OX=9823 GN=ATP2A3 PE=3 SV=4;tr|A0A286ZVJ3|A0A286ZVJ3_PIG Calcium-transporting ATPase OS=Sus scrofa OX=9823 GN=ATP2A3 PE=3 SV=1;sp|O77696|AT2A3_PIG Sarcoplasmic/endoplasmic reticulum calcium ATP
752980000 998730000 440960000 424050000 948080000 352840000 909640000
757210000 414650000 577490000 619010000 293150000 14 14
14 73,2 73,2 73,2 21,639 0 114,99 36439000000 451
P00571;A0A286ZQ79;A0A5S6G079;A0A287AJJ9;F1S9R3;A0A5G2RN95
P00571;A0A286ZQ79;A0A5S6G079 92 sp|P00571|KAD1_PIG Adenylate kinase isoenzyme 1 OS=Sus scrofa OX=9823 GN=AK1 PE=1 SV=1;tr|A0A286ZQ79|A0A286ZQ79_PIG Adenylate kinase isoenzyme 1 OS=Sus scrofa OX=9823 GN=AK1 PE=3 SV=2;tr|A0A5S6G079|A0A5S6G079_PIG Adenylate kinase isoenzyme 1 OS=Sus scrofa
0 0 0 0 0 0 0 0 0 0 0 0 1 1 1 9,8 9,8 9,8
14,461 0 4,3086 0 1
Q29375;A0A287BIE2;A0A287BM53;A0A5G2QBT0;A0A286ZQA5;A0A5G2R293
Q29375;A0A287BIE2;A0A287BM53;A0A5G2QBT0;A0A286ZQA5;A0A5G2R293 93
sp|Q29375|RL7A_PIG 60S ribosomal protein L7a (Fragment) OS=Sus scrofa OX=9823 GN=RPL7A PE=2 SV=1;tr|A0A287BIE2|A0A287BIE2_PIG Ribosomal_L7Ae domain-containing protein OS=Sus scrofa OX=9823 PE=4 SV=2;tr|A0A287BM53|A0A287BM53_PIG 60S ribosomal protein L7a OS
0 0 0 0 0 0 0 0 0 0 0 0 1 1 1 4 4 4
22,196 0,0078278 1,4927 78443000 1
A0A287BH85;A0A5G2QS99;I3LL89;A0A5G2Q9I7;A0A480EFR8;A0A286ZQM7
A0A287BH85;A0A5G2QS99;I3LL89;A0A5G2Q9I7;A0A480EFR8;A0A286ZQM7 94
tr|A0A287BH85|A0A287BH85_PIG Synaptoporin OS=Sus scrofa OX=9823 GN=SYNPR PE=1 SV=2;tr|A0A5G2QS99|A0A5G2QS99_PIG Synaptoporin OS=Sus scrofa OX=9823 GN=SYNPR PE=1 SV=1;tr|I3LL89|I3LL89_PIG Synaptoporin OS=Sus scrofa OX=9823 GN=SYNPR PE=1 SV=2;tr|A0A5G2Q9I7|A
0 0 0 0 0 0 0 0 0 0 0 0 1 1 1 0,7 0,7 0,7
122,76 0,009542 1,363 14145000 1
A0A287B9L4;I3LTV3;A0A287A1U4;A0A481BP96;A0A286ZQP1
A0A287B9L4;I3LTV3;A0A287A1U4;A0A481BP96;A0A286ZQP1 95
tr|A0A287B9L4|A0A287B9L4_PIG Fibronectin type III domain containing 3A OS=Sus scrofa OX=9823 GN=FNDC3A PE=1 SV=2;tr|I3LTV3|I3LTV3_PIG Fibronectin type III domain containing 3A OS=Sus scrofa OX=9823 GN=FNDC3A PE=1 SV=2;tr|A0A287A1U4|A0A287A1U4_PIG Fibronect

37804000 29405000 51564000 54691000 45472000 39979000 52934000 30721000 34721000 59403000
35818000 57734000 10 10 10 29,9 29,9 29,9 37,406 0 53,753
2430800000 86 A0A286ZQY6;P56471 A0A286ZQY6 96
tr|A0A286ZQY6|A0A286ZQY6_PIG Isocitrate dehydrogenase [NAD] subunit, mitochondrial OS=Sus scrofa OX=9823 GN=IDH3A PE=1 SV=2
0 0 0 0 0 0 0 0 0 0 0 0 1 1 1 2,9 2,9 2,9
46,08 0,0022371 2,4717 4879100 1
A0A287A9Z2;F1SHX9;A0A5G2QW07;A0A286ZQZ4
A0A287A9Z2;F1SHX9;A0A5G2QW07;A0A286ZQZ4 97
tr|A0A287A9Z2|A0A287A9Z2_PIG L-2-hydroxyglutarate dehydrogenase OS=Sus scrofa OX=9823 GN=L2HGDH PE=1 SV=1;tr|F1SHX9|F1SHX9_PIG L-2-hydroxyglutarate dehydrogenase OS=Sus scrofa OX=9823 GN=L2HGDH PE=1 SV=2;tr|A0A5G2QW07|A0A5G2QW07_PIG L-2-hydroxyglutarate de
0 0 0 0 0 0 0 0 0 0 0 0 2 2 2 11,4 11,4 11,4
14,55 0,0022173 2,3854 177810000 8
F2Z5K9;A0A286ZRB3;Q71LE2;F1RVA0;A0A286ZTM7;A0A287BKF7;A0A287B5T7
F2Z5K9;A0A286ZRB3;Q71LE2;F1RVA0;A0A286ZTM7;A0A287BKF7;A0A287B5T7 98
tr|F2Z5K9|F2Z5K9_PIG Histone domain-containing protein OS=Sus scrofa OX=9823 PE=3 SV=3;tr|A0A286ZRB3|A0A286ZRB3_PIG Histone domain-containing protein OS=Sus scrofa OX=9823 PE=1 SV=1;sp|Q71LE2|H33_PIG Histone H3.3 OS=Sus scrofa OX=9823 GN=H3-3A PE=2 SV=3;tr
15549000 13940000 21478000 19030000 20304000 25966000 17225000 15675000 20299000 19567000
20659000 32329000 10 10 10 17,7 17,7 17,7 70,902 0 28,691
1430100000 100
A0A286ZRC3;A0A287AVT7;A0A5G2QG61;A0A287A2X3;F1RR45;A0A5G2R4L7;A0A287AMX9
A0A286ZRC3;A0A287AVT7;A0A5G2QG61;A0A287A2X3;F1RR45;A0A5G2R4L7;A0A287AMX9
99 tr|A0A286ZRC3|A0A286ZRC3_PIG Carnitine O-acetyltransferase OS=Sus scrofa OX=9823 GN=CRAT PE=1 SV=1;tr|A0A287AVT7|A0A287AVT7_PIG Carnitine O-acetyltransferase OS=Sus scrofa OX=9823 GN=CRAT PE=1 SV=1;tr|A0A5G2QG61|A0A5G2QG61_PIG Carnitine O-acetyltransferase
8742300 10612000 16625000 10282000 12858000 25523000 7508900 7894900 16106000 11895000
13510000 22483000 20 20 9 24,9 24,9 9,6 103,62 0 65,034
1312400000 100
A0A287AVW1;A0A286ZRG2;D2WKD8;A0A287BCY9;A0A5S6G1Z7;A0A286ZRK2;A0A5G2R1K3;A0A5K1U342;A0A5G2R034;A0A5G2QW96;A0A5G2QJY8;I3L8L8;A0A5G2Q9U8;I3LN55;F1RM59;P19156;A0A5G2QYH2;A0A5G2R1K0
A0A287AVW1;A0A286ZRG2;D2WKD8;A0A287BCY9;A0A5S6G1Z7;A0A286ZRK2;A0A5G2R1K3;A0A5K1U342;A0A5G2R034;A0A5G2QW96 100 tr|A0A287AVW1|A0A287AVW1_PIG Sodium/potassium-transporting ATPase subunit alpha OS=Sus scrofa OX=9823 GN=LOC100157711 PE=3 SV=1;tr|A0A286ZRG2|A0A286ZRG2_PIG Sodium/potassium-transporting ATPase subunit alpha OS=Sus scrofa OX=9823 GN=LOC100157711 PE=3 SV=2;
0 11083000 10612000 0 0 9907100 0 0 0 0 0 0 3 3 2
7,2 7,2 5,6 61,465 0 5,8191 108800000 10

A0A286ZRG9;F1RQM4;A0A5G2RAJ8;Q29558;A0A5G2QSJ2;A0A5G2R7A1;F1RPN9;A0A5G2QMD3;A0A5G2QB59;A0A286ZNS2 A0A286ZRG9;F1RQM4;A0A5G2RAJ8;Q29558 101

tr|A0A286ZRG9|A0A286ZRG9_PIG Malic enzyme OS=Sus scrofa OX=9823 GN=ME1 PE=1 SV=1;tr|F1RQM4|F1RQM4_PIG Malic enzyme OS=Sus scrofa OX=9823 GN=ME1 PE=1 SV=4;tr|A0A5G2RAJ8|A0A5G2RAJ8_PIG Malic enzyme OS=Sus scrofa OX=9823 GN=ME1 PE=1 SV=1;sp|Q29558|MAOX_PIG NA

21239000 14831000 37329000 12144000 25036000 33132000 11271000 14276000 22210000 32025000 27778000 49151000 4 4 4 19,5 19,5 19,5 25,43 0 18,55 848130000 42 A0A5G2RD05;F1SNF6;A0A286ZRH7;A0A287AC97;A0A286ZUS4

A0A5G2RD05;F1SNF6;A0A286ZRH7;A0A287AC97;A0A286ZUS4 102

tr|A0A5G2RD05|A0A5G2RD05_PIG Uncharacterized protein OS=Sus scrofa OX=9823 GN=CHCHD3 PE=1 SV=1;tr|F1SNF6|F1SNF6_PIG MICOS complex subunit MIC19 isoform 2 OS=Sus scrofa OX=9823 GN=CHCHD3 PE=1 SV=2;tr|A0A286ZRH7|A0A286ZRH7_PIG Uncharacterized protein OS=Sus

252090000 240880000 151600000 135150000 166840000 147430000 253150000 157350000 154670000 266010000 143950000 144880000 18 18 18 42,5 42,5 42,5 49,774 0 269,75 11892000000 339

F1RXZ6;A0A287B1S9;A0A287B1H6;A0A287BAF8;A0A286ZRI3;A0A287AW68;A0A287AVP0;A0A287AMG7;A0A286ZWH1;A0A287B568;F1SH84;A0A287AX64;I3L7Y9;A0A5G2RHA8;A0A5G2R5I4;I3L8X6

F1RXZ6;A0A287B1S9;A0A287B1H6;A0A287BAF8;A0A286ZRI3;A0A287AW68;A0A287AVP0;A0A287AMG7;A0A286ZWH1;A0A287B568 103 tr|F1RXZ6|F1RXZ6_PIG Bridging integrator 1 OS=Sus scrofa OX=9823 GN=BIN1 PE=1 SV=3;tr|A0A287B1S9|A0A287B1S9_PIG Bridging integrator 1 OS=Sus scrofa OX=9823 GN=BIN1 PE=1 SV=1;tr|A0A287B1H6|A0A287B1H6_PIG Bridging integrator 1 OS=Sus scrofa OX=9823 GN=BIN1 P

30214000 58823000 40168000 27306000 100060000 0 70622000 79185000 38672000 48477000 46518000 0 5 5 5 41,5 41,5 41,5 14,694 0 15,626 895810000 41 Q49I35;A0A286ZRI5;A0A286ZWB2;A0A5G2QD67;A0A5G2Q797

Q49I35;A0A286ZRI5;A0A286ZWB2;A0A5G2QD67;A0A5G2Q797 104 sp|Q49I35|LEG1_PIG Galectin-1 OS=Sus scrofa OX=9823 GN=LGALS1 PE=2 SV=3;tr|A0A286ZRI5|A0A286ZRI5_PIG Galectin OS=Sus scrofa OX=9823 GN=LGALS1 PE=1 SV=1;tr|A0A286ZWB2|A0A286ZWB2_PIG Galectin OS=Sus scrofa OX=9823 GN=LGALS1 PE=1 SV=2;tr|A0A5G2QD67|A0A5G2QD67

0 0 0 0 0 0 0 0 0 0 0 0 1 1 1 2,6 2,6 2,6 52,129 0,0031983 2,0861 0 1

A0A5G2QV42;A0A5G2R1N1;A0A286ZSJ9;A0A286ZRQ3;A0A287A100;A0A5G2QAR5;A0A5G2R934;A0A5G2QQD1;E7EI18;F1SMQ4

A0A5G2QV42;A0A5G2R1N1;A0A286ZSJ9;A0A286ZRQ3;A0A287A100;A0A5G2QAR5;A0A5G2R934;A0A5G2QQD1;E7EI18;F1SMQ4 105 tr|A0A5G2QV42|A0A5G2QV42_PIG Adenosylhomocysteinase like 1 OS=Sus scrofa OX=9823 GN=AHCYL1 PE=1 SV=1;tr|A0A5G2R1N1|A0A5G2R1N1_PIG Adenosylhomocysteinase like 1 OS=Sus scrofa OX=9823 GN=AHCYL1 PE=1 SV=1;tr|A0A286ZSJ9|A0A286ZSJ9_PIG Adenosylhomocysteinase li

0 0 0 0 0 0 0 0 0 0 0 0 1 1 1 2,9 2,9 2,9 46,524 0 2,9179 5149000 3 A0A286ZRU9 A0A286ZRU9 106

tr|A0A286ZRU9|A0A286ZRU9_PIG Collagen-binding protein OS=Sus scrofa OX=9823 GN=SERPINH1 PE=1 SV=1

116930000 96419000 84509000 140180000 111740000 77728000 154580000 97794000

54593000 90655000 116550000 77789000 8 8 8 24,7 24,7 24,7 36,262

0 46,073 3307800000 100

A0A480XNH5;A0A287BDZ2;A0A286ZRX4;A0A5G2QVC5;A0A5G2QZZ8

A0A480XNH5;A0A287BDZ2;A0A286ZRX4;A0A5G2QVC5;A0A5G2QZZ8 107

tr|A0A480XNH5|A0A480XNH5_PIG Mono-ADP ribosylhydrolase 1 OS=Sus scrofa OX=9823 GN=MACROD1 PE=1 SV=1;tr|A0A287BDZ2|A0A287BDZ2_PIG Mono-ADP ribosylhydrolase 1 OS=Sus scrofa OX=9823 GN=MACROD1 PE=1 SV=2;tr|A0A286ZRX4|A0A286ZRX4_PIG Mono-ADP ribosylhydrolase 1

102560000 82132000 84511000 43540000 55782000 52934000 40880000 57736000 49885000 46840000

69497000 81446000 52 52 1 24,2 24,2 0,6 286,58 0 284,11

6490800000 352

A0A286ZRX8;A0A5G2R269;F1SMN5;A0A5G2QY64;F1SGJ3;A0A286ZPG4;A0A287BLE0;A0A287AHQ3 A0A286ZRX8;A0A5G2R269;F1SMN5;A0A5G2QY64 108

tr|A0A286ZRX8|A0A286ZRX8_PIG Filamin C OS=Sus scrofa OX=9823 GN=FLNC PE=1 SV=1;tr|A0A5G2R269|A0A5G2R269_PIG Filamin C OS=Sus scrofa OX=9823 GN=FLNC PE=1 SV=1;tr|F1SMN5|F1SMN5_PIG Filamin C OS=Sus scrofa OX=9823 GN=FLNC PE=1 SV=1;tr|A0A5G2QY64|A0A5G2QY64_PI

18885000 13654000 14462000 25982000 11381000 14977000 23128000 11139000 12300000 28466000

12766000 11437000 4 4 2 26,4 26,4 13,8 19,104 0 17,193

454510000 52 A0A287BNU5;A0A286ZS27;A0A287ARC7;Q06AU6;A0A5G2R4N3

A0A287BNU5;A0A286ZS27;A0A287ARC7;Q06AU6;A0A5G2R4N3109

tr|A0A287BNU5|A0A287BNU5_PIG Uncharacterized protein OS=Sus scrofa OX=9823 GN=CDK2 PE=1 SV=2;tr|A0A286ZS27|A0A286ZS27_PIG Uncharacterized protein OS=Sus scrofa OX=9823 GN=CDK2 PE=1 SV=1;tr|A0A287ARC7|A0A287ARC7_PIG Protein kinase domain-containing protein

21576000 14152000 14378000 36747000 14422000 14235000 35240000 10574000 17821000 36872000

16340000 21500000 8 8 8 32,9 32,9 32,9 27,219 0 26,111

950790000 55 A0A5G2QW86;A0A5G2QGZ8;A0A286ZS77;A0A5G2RDX9;A0A5K1V4A5

A0A5G2QW86;A0A5G2QGZ8;A0A286ZS77;A0A5G2RDX9;A0A5K1V4A5 110

tr|A0A5G2QW86|A0A5G2QW86_PIG Basigin OS=Sus scrofa OX=9823 GN=BSG PE=1 SV=1;tr|A0A5G2QGZ8|A0A5G2QGZ8_PIG Basigin OS=Sus scrofa OX=9823 GN=BSG PE=1 SV=1;tr|A0A286ZS77|A0A286ZS77_PIG Basigin OS=Sus scrofa OX=9823 GN=BSG PE=1 SV=1;tr|A0A5G2RDX9|A0A5G2RDX9_PIG

275410000 163210000 177580000 265470000 194470000 240190000 275000000

115800000 175170000 278720000 162650000 153340000 2 2

2 8,3 8,3 8,3 22,076 0 9,9962 4642300000 32

A0A287A0T4;A0A287A9N4;A0A287AAB1;A0A286ZS99;A0A287AE95;A0A287B985;A0A5G2RB25

A0A287A0T4;A0A287A9N4;A0A287AAB1;A0A286ZS99;A0A287AE95;A0A287B985;A0A5G2RB25 111 tr|A0A287A0T4|A0A287A0T4_PIG Reticulon OS=Sus scrofa OX=9823 GN=RTN2 PE=4

SV=2;tr|A0A287A9N4|A0A287A9N4_PIG Reticulon OS=Sus scrofa OX=9823 GN=RTN2 PE=4 SV=2;tr|A0A287AAB1|A0A287AAB1_PIG Reticulon OS=Sus scrofa OX=9823 GN=RTN2 PE=4 SV=1;tr|A0A286ZS99|A0A28
17414000 17426000 11372000 18605000 10811000 0 24963000 0 0 28784000 0 0
3 3 3 15 15 15 26,251 0 17,68 303730000 16 A0A286ZSA7;I3LHC8
A0A286ZSA7;I3LHC8 112 tr|A0A286ZSA7|A0A286ZSA7_PIG 3-hydroxybutyrate dehydrogenase 2 OS=Sus scrofa OX=9823 GN=BDH2 PE=1 SV=1;tr|I3LHC8|I3LHC8_PIG 3-hydroxybutyrate dehydrogenase 2 OS=Sus scrofa OX=9823 GN=BDH2 PE=1 SV=1
6911900 0 0 11601000 0 0 9898200 0 0 11135000 0 7985800 8
8 8 33,3 33,3 33,3 37,18 0 37,243 172400000 18
F1S269;A0A286ZSH0;A0A5G2R4Y1;A0A5G2RF79
F1S269;A0A286ZSH0;A0A5G2R4Y1;A0A5G2RF79 113 tr|F1S269|F1S269_PIG RCSD domain containing 1 OS=Sus scrofa OX=9823 GN=RCSD1 PE=1 SV=4;tr|A0A286ZSH0|A0A286ZSH0_PIG RCSD domain containing 1 OS=Sus scrofa OX=9823 GN=RCSD1 PE=1 SV=1;tr|A0A5G2R4Y1|A0A5G2R4Y1_PIG RCSD domain containing 1 OS=Sus scrofa OX=982
0 0 0 0 0 0 0 0 0 0 0 0 1 1 1 5,2 5,2 5,2
32,226 0,0021645 2,1834 2374900 2 A0A286ZSM1 A0A286ZSM1 114
tr|A0A286ZSM1|A0A286ZSM1_PIG Very-long-chain (3R)-3-hydroxyacyl-CoA dehydratase OS=Sus scrofa OX=9823 GN=HACD1 PE=3 SV=1
14646000 9286500 10526000 0 6192200 7322400 5446400 0 7888600 7757900 0 8797700
7 7 7 16,3 16,3 16,3 63,165 0 20,651 237390000 25
F1S441;A0A286ZSW6;A0A287AME1;A0A5G2QQK5;A0A287AXF5;A0A286ZLE4;A0A287ASN5
F1S441;A0A286ZSW6;A0A287AME1;A0A5G2QQK5;A0A287AXF5;A0A286ZLE4;A0A287ASN5
115 tr|F1S441|F1S441_PIG Calnexin OS=Sus scrofa OX=9823 GN=CANX PE=1 SV=2;tr|A0A286ZSW6|A0A286ZSW6_PIG Calnexin OS=Sus scrofa OX=9823 GN=CANX PE=1 SV=2;tr|A0A287AME1|A0A287AME1_PIG Calnexin OS=Sus scrofa OX=9823 GN=CANX PE=1 SV=1;tr|A0A5G2QQK5|A0A5G2QQK5_PIG C
267000000 0 0 275250000 0 401180000 0 0 177370000 288080000
155510000 145930000 2 2 1 18,3 18,3 8,9 18,009 0 32,546
2679400000 37 A0A287BAB3;A0A286ZSX6;A0A286ZIM1
A0A287BAB3;A0A286ZSX6;A0A286ZIM1 116 tr|A0A287BAB3|A0A287BAB3_PIG Ig-like domain-containing protein OS=Sus scrofa OX=9823 PE=4 SV=2;tr|A0A286ZSX6|A0A286ZSX6_PIG Ig-like domain-containing protein OS=Sus scrofa OX=9823 PE=4 SV=2;tr|A0A286ZIM1|A0A286ZIM1_PIG Ig-like domain-containing protein OS=
1785400000 1147300000 1252000000 1578600000 741790000 1586000000 1884300000
645430000 699980000 2069500000 828480000 1075000000 44 44
31 60,2 60,2 43,7 67,168 0 323,31 188380000000 1635
A0A287AMK0;A0A286ZT13;P08835;F1RUN2;A0A287BAY9;A0A287AFS2;REV__A0A287BGS7
A0A287AMK0;A0A286ZT13;P08835;F1RUN2;A0A287BAY9;A0A287AFS2 117
tr|A0A287AMK0|A0A287AMK0_PIG Albumin OS=Sus scrofa OX=9823 GN=ALB PE=1 SV=1;tr|A0A286ZT13|A0A286ZT13_PIG Albumin OS=Sus scrofa OX=9823 GN=ALB PE=1 SV=1;sp|P08835|ALBU_PIG Albumin OS=Sus scrofa OX=9823 GN=ALB PE=1 SV=2;tr|F1RUN2|F1RUN2_PIG Albumin OS=Sus sc

18066000 11572000 13237000 3703300 9867200 0 15292000 0 8441800 8980800 6918300
6864300 10 10 10 22,9 22,9 22,9 48,162 0 15,615 392800000 33
A0A287BQI6;A0A286ZT34;I3LNP9;A0A287AEC0;A0A287BNK1
A0A287BQI6;A0A286ZT34;I3LNP9;A0A287AEC0 118 tr|A0A287BQI6|A0A287BQI6_PIG Basic leucine zipper and W2 domains 2 OS=Sus scrofa OX=9823 GN=BZW2 PE=1 SV=1;tr|A0A286ZT34|A0A286ZT34_PIG Basic leucine zipper and W2 domains 2 OS=Sus scrofa OX=9823 GN=BZW2 PE=1 SV=1;tr|I3LNP9|I3LNP9_PIG Basic leucine zipper
12146000 0 5907000 0 0 0 0 0 0 0 0 0 2 2 2 5,3
5,3 5,3 44,323 0,0032293 2,13 70735000 8
F1SID4;A0A5G2Q9I2;A0A286ZT52;A0A5G2QSU8
F1SID4;A0A5G2Q9I2;A0A286ZT52;A0A5G2QSU8 119 tr|F1SID4|F1SID4_PIG AAA domain-containing protein OS=Sus scrofa OX=9823 GN=PSMC3 PE=1 SV=3;tr|A0A5G2Q9I2|A0A5G2Q9I2_PIG AAA domain-containing protein OS=Sus scrofa OX=9823 GN=PSMC3 PE=1 SV=1;tr|A0A286ZT52|A0A286ZT52_PIG AAA domain-containing protein OS=Su
25734000 9330600 13321000 17415000 10132000 17437000 21486000 4229600 14130000 14198000
7831900 23796000 12 12 12 44,6 44,6 44,6 25,992 0 51,102
971710000 76 A0A286ZT82;A0A287AZK2 A0A286ZT82;A0A287AZK2 120
tr|A0A286ZT82|A0A286ZT82_PIG Transgelin OS=Sus scrofa OX=9823 GN=TAGLN PE=1 SV=1;tr|A0A287AZK2|A0A287AZK2_PIG Transgelin OS=Sus scrofa OX=9823 GN=TAGLN PE=1 SV=1
13047000 0 7157800 10362000 0 20443000 12784000 0 14997000 9450400 0 12548000
5 5 5 13,2 13,2 13,2 50,868 0 16,118 287380000 22
A0A287B5C1;A0A287ALC1;A0A287BM31;A0A286ZT94;A0A286ZTC9;A0A287ANX9
A0A287B5C1;A0A287ALC1;A0A287BM31;A0A286ZT94;A0A286ZTC9 121
tr|A0A287B5C1|A0A287B5C1_PIG Uncharacterized protein OS=Sus scrofa OX=9823 PE=4 SV=2;tr|A0A287ALC1|A0A287ALC1_PIG Uncharacterized protein OS=Sus scrofa OX=9823 PE=4 SV=2;tr|A0A287BM31|A0A287BM31_PIG Uncharacterized protein OS=Sus scrofa OX=9823 PE=4 SV=1;t
0 0 0 0 0 0 0 0 0 0 0 0 2 2 2 13 13 13
20,088 0 5,9107 10335000 2
A0A5G2QPH7;A0A5G2R902;A0A287BCR7;A0A286ZT96;F1RPV1
A0A5G2QPH7;A0A5G2R902;A0A287BCR7;A0A286ZT96;F1RPV1 122
tr|A0A5G2QPH7|A0A5G2QPH7_PIG TIP41-like protein OS=Sus scrofa OX=9823 GN=TIPRL PE=1 SV=1;tr|A0A5G2R902|A0A5G2R902_PIG TIP41-like protein OS=Sus scrofa OX=9823 GN=TIPRL PE=1 SV=1;tr|A0A287BCR7|A0A287BCR7_PIG TIP41-like protein OS=Sus scrofa OX=9823 GN=TIPRL
5071700 6627200 36952000 4970700 14404000 20472000 6278200 8024500 15056000 10848000
16637000 25902000 7 7 7 17,7 17,7 17,7 53,591 0 19,811
633480000 45
A0A5G2R406;A0A286ZTB0;A0A5G2QT34;F1ST01;A0A286ZVY0;A0A480MVX6;A0A5G2QX06;A0A5G2QLA7;A0A5G2R8J3;A0A286ZRR3
A0A5G2R406;A0A286ZTB0;A0A5G2QT34;F1ST01;A0A286ZVY0;A0A480MVX6;A0A5G2QX06;A0A5G2QLA7;A0A5G2R8J3;A0A286ZRR3123 tr|A0A5G2R406|A0A5G2R406_PIG Selenium

binding protein 1 OS=Sus scrofa OX=9823 GN=SELENBP1 PE=1 SV=1;tr|A0A286ZTB0|A0A286ZTB0_PIG Selenium binding protein 1 OS=Sus scrofa OX=9823 GN=SELENBP1 PE=1 SV=2;tr|A0A5G2QT34|A0A5G2QT34_PIG Selenium binding protein 1
0 0 0 0 0 0 0 0 0 0 0 0 3 3 3 25 25 25
16,145 0 6,5263 68860000 9
A0A5G2R4K1;A0A287B8I6;F6Q3P3;A0A5G2RFW2;A0A286ZTI3;A0A286ZZ57;A0A287B5T6;A0A5G2QSV6;B9V4F0;A0A287BM57;A0A5G2QS08;A0A287A7C1;A0A287A1L1;A0A5G2R3T3;A0A287A1M9
A0A5G2R4K1;A0A287B8I6;F6Q3P3;A0A5G2RFW2;A0A286ZTI3;A0A286ZZ57;A0A287B5T6;A0A5G2QSV6;B9V4F0;A0A287BM57;A0A5G2QS08;A0A287A7C1 124
tr|A0A5G2R4K1|A0A5G2R4K1_PIG Uncharacterized protein OS=Sus scrofa OX=9823 GN=FKBP11 PE=4 SV=1;tr|A0A287B8I6|A0A287B8I6_PIG Uncharacterized protein OS=Sus scrofa OX=9823 GN=FKBP11 PE=3 SV=2;tr|F6Q3P3|F6Q3P3_PIG ADP ribosylation factor 1 OS=Sus scrofa OX=98
2446000000 3688600000 7428100000 2910100000 10067000000 7222200000 2036900000
3276700000 5010000000 2261200000 6902000000 8650700000 8 8
8 30,2 30,2 30,2 31,341 0 30,864 65607000000 88
A0A287APA1;A0A5G2QPT6;A0A287ACQ7;A0A5G2QWH5;A0A287ABB8;A0A286ZTN4;A0A287BEE0
A0A287APA1;A0A5G2QPT6;A0A287ACQ7;A0A5G2QWH5;A0A287ABB8;A0A286ZTN4;A0A287BEE0 125 tr|A0A287APA1|A0A287APA1_PIG NIPSNAP domain-containing protein OS=Sus scrofa OX=9823 GN=NIPSNAP2 PE=1 SV=1;tr|A0A5G2QPT6|A0A5G2QPT6_PIG NIPSNAP domain-containing protein OS=Sus scrofa OX=9823 GN=NIPSNAP2 PE=1 SV=1;tr|A0A287ACQ7|A0A287ACQ7_PIG NIPSNAP domai
0 0 0 0 0 0 0 0 0 0 0 0 2 2 2 16,9 16,9 16,9
15,182 0 3,5431 15733000 4 A0A5G2QEE8;A0A286ZTS1 A0A5G2QEE8;A0A286ZTS1
126 tr|A0A5G2QEE8|A0A5G2QEE8_PIG Trans-3-hydroxy-L-proline dehydratase OS=Sus scrofa OX=9823 GN=L3HYPDH PE=3 SV=1;tr|A0A286ZTS1|A0A286ZTS1_PIG Trans-3-hydroxy-L-proline dehydratase OS=Sus scrofa OX=9823 GN=L3HYPDH PE=3 SV=1
0 0 0 0 0 0 0 0 0 0 0 0 1 1 1 1 1 1
141,27 0,0086207 1,3817 0 1
A0A5G2QWB8;A0A286ZTV9;A0A287A2J1;A0A287BSL5;A0A287AVK6;A0A287AMQ1;A0A480MFV9;F1SSN5;A0A286ZZV9
A0A5G2QWB8;A0A286ZTV9;A0A287A2J1;A0A287BSL5;A0A287AVK6;A0A287AMQ1;A0A480MFV9;F1SSN5;A0A286ZZV9 127 tr|A0A5G2QWB8|A0A5G2QWB8_PIG Kinectin 1 OS=Sus scrofa OX=9823 GN=KTN1 PE=1 SV=1;tr|A0A286ZTV9|A0A286ZTV9_PIG Kinectin 1 OS=Sus scrofa OX=9823 GN=KTN1 PE=1 SV=2;tr|A0A287A2J1|A0A287A2J1_PIG Kinectin 1 OS=Sus scrofa OX=9823 GN=KTN1 PE=1 SV=2;tr|A0A287BSL5|A0
0 0 0 0 0 0 0 0 0 0 0 0 1 1 1 4,6 4,6 4,6
35,766 0,0021692 2,2053 19486000 3
A0A5G2RGM3;A0A287ATT7;A0A286ZU25;F1S8D4
A0A5G2RGM3;A0A287ATT7;A0A286ZU25;F1S8D4 128
tr|A0A5G2RGM3|A0A5G2RGM3_PIG WD repeat domain 77 OS=Sus scrofa OX=9823

GN=WDR77 PE=1 SV=1;tr|A0A287ATT7|A0A287ATT7_PIG WD repeat domain 77 OS=Sus scrofa OX=9823 GN=WDR77 PE=1 SV=2;tr|A0A286ZU25|A0A286ZU25_PIG WD repeat domain 77 OS=Sus scrofa OX=9823 GN=WD
0 0 0 0 0 0 0 0 0 0 0 0 2 2 2 13,1 13,1 13,1
26,522 0 7,18 71882000 6 A0A286ZU36;I3LIA4 A0A286ZU36;I3LIA4 129
tr|A0A286ZU36|A0A286ZU36_PIG Guanidinoacetate N-methyltransferase OS=Sus scrofa OX=9823 GN=GAMT PE=1 SV=1;tr|I3LIA4|I3LIA4_PIG Guanidinoacetate N-methyltransferase OS=Sus scrofa OX=9823 GN=GAMT PE=1 SV=2
0 0 0 0 0 0 0 0 0 0 0 0 2 2 2 1,5 1,5 1,5
229,58 0 3,6584 22832000 4
A0A286ZU47;A0A287AXE7;F1RWD6;A0A287A071;F1RWD9;A0A287BT51;A0A287BHD1
A0A286ZU47;A0A287AXE7;F1RWD6;A0A287A071;F1RWD9;A0A287BT51;A0A287BHD1 130
tr|A0A286ZU47|A0A286ZU47_PIG Supervillin OS=Sus scrofa OX=9823 GN=SVIL PE=1 SV=1;tr|A0A287AXE7|A0A287AXE7_PIG Supervillin OS=Sus scrofa OX=9823 GN=SVIL PE=1 SV=2;tr|F1RWD6|F1RWD6_PIG Supervillin OS=Sus scrofa OX=9823 GN=SVIL PE=1 SV=4;tr|A0A287A071|A0A287A
0 0 0 0 0 0 0 0 0 0 0 0 2 2 2 4,6 4,6 4,6
60,341 0,0031579 1,9739 11576000 3
I3L7S4;A0A287AXR7;F1SNH3;A0A286ZUH0;I3L650
I3L7S4;A0A287AXR7;F1SNH3;A0A286ZUH0;I3L650 131 tr|I3L7S4|I3L7S4_PIG Caldesmon 1 OS=Sus scrofa OX=9823 GN=CALD1 PE=1 SV=3;tr|A0A287AXR7|A0A287AXR7_PIG Caldesmon 1 OS=Sus scrofa OX=9823 GN=CALD1 PE=1 SV=1;tr|F1SNH3|F1SNH3_PIG Caldesmon 1 OS=Sus scrofa OX=9823 GN=CALD1 PE=1 SV=2;tr|A0A286ZUH0|A0A286ZUH0_P
46738000 81591000 57967000 76916000 103880000 58321000 69193000 62524000 80105000 72224000
76852000 40415000 3 3 3 11,4 11,4 11,4 37,404 0 17,38
1160900000 50
A0A286ZUI9;A0A5G2R4T3;A0A5G2QYM2;F1SKC4;A0A287BSM6;A0A5G2R329;A0A287ASA2;A0A287BBS7;A0A287A756;A0A5G2R2E9 A0A286ZUI9;A0A5G2R4T3;A0A5G2QYM2;F1SKC4 132
tr|A0A286ZUI9|A0A286ZUI9_PIG Glycogenin 1 OS=Sus scrofa OX=9823 GN=GYG1 PE=1 SV=1;tr|A0A5G2R4T3|A0A5G2R4T3_PIG Glycogenin 1 OS=Sus scrofa OX=9823 GN=GYG1 PE=1 SV=1;tr|A0A5G2QYM2|A0A5G2QYM2_PIG Glycogenin 1 OS=Sus scrofa OX=9823 GN=GYG1 PE=1 SV=1;tr|F1SKC4|
35957000 43369000 28176000 48993000 36025000 36639000 34876000 27598000 33822000 33110000
31503000 28349000 22 22 22 31,6 31,6 31,6 89,302 0 75,554
2944700000 144 A0A480IPJ6;F1SIH8;A0A286ZUM8;P03974;A0A286ZSB5;A0A5G2RLZ5
A0A480IPJ6;F1SIH8;A0A286ZUM8;P03974;A0A286ZSB5;A0A5G2RLZ5 133
tr|A0A480IPJ6|A0A480IPJ6_PIG 15S Mg(2+)-ATPase p97 subunit OS=Sus scrofa OX=9823 GN=VCP PE=1 SV=1;tr|F1SIH8|F1SIH8_PIG 15S Mg(2+)-ATPase p97 subunit OS=Sus scrofa OX=9823 GN=VCP PE=1 SV=3;tr|A0A286ZUM8|A0A286ZUM8_PIG 15S Mg(2+)-ATPase p97 subunit OS=Sus sc
0 0 0 0 0 0 0 0 0 0 0 0 3 3 3 15,4 15,4 15,4
22,156 0 11,646 388580000 5 A0A286ZUN9;A0A287BDC0;A0A287BJQ2
A0A286ZUN9;A0A287BDC0;A0A287BJQ2 134 tr|A0A286ZUN9|A0A286ZUN9_PIG Complex

I-23kD OS=Sus scrofa OX=9823 GN=NDUFS8 PE=1 SV=2;tr|A0A287BDC0|A0A287BDC0_PIG Complex I-23kD OS=Sus scrofa OX=9823 GN=NDUFS8 PE=1 SV=2;tr|A0A287BJQ2|A0A287BJQ2_PIG Complex I-23kD OS=Sus scrofa OX=9823 GN=NDUFS8 PE=1 SV=
19933000 9257800 20509000 14437000 9228000 37669000 18622000 0 13153000 21291000 11062000 21006000 8 8 8 15,7 15,7 15,7 55,704 0 19,484 860910000 63
F1SC20;A0A287AAD5;A0A286ZUY9;CON__Q2KJF1 F1SC20;A0A287AAD5;A0A286ZUY9 135 tr|F1SC20|F1SC20_PIG Alpha-1B-glycoprotein OS=Sus scrofa OX=9823 GN=A1BG PE=1 SV=2;tr|A0A287AAD5|A0A287AAD5_PIG Alpha-1B-glycoprotein OS=Sus scrofa OX=9823 GN=A1BG PE=1 SV=2;tr|A0A286ZUY9|A0A286ZUY9_PIG Alpha-1B-glycoprotein OS=Sus scrofa OX=9823 GN=A1BG P
16442000 23808000 39650000 1449400 34704000 40662000 0 11398000 24608000 6665500 18690000 75918000 20 20 20 23,6 23,6 23,6 111,01 0 88,029 2004700000 116
A0A286ZV48;K9IVI1;A0A287B410;A0A287ARP4;F1SSH8;A0A5G2QAZ0;A0A5G2QGM1;F1SDW6;A0A286ZWR6
A0A286ZV48;K9IVI1;A0A287B410;A0A287ARP4;F1SSH8;A0A5G2QAZ0;A0A5G2QGM1 136
tr|A0A286ZV48|A0A286ZV48_PIG Oxoglutarate dehydrogenase (succinyl-transferring) OS=Sus scrofa OX=9823 GN=OGDH PE=1 SV=1;tr|K9IVI1|K9IVI1_PIG Oxoglutarate dehydrogenase (succinyl-transferring) OS=Sus scrofa OX=9823 GN=OGDH PE=1 SV=1;tr|A0A287B410|A0A287B410
32764000 18058000 19302000 24458000 11975000 17452000 33395000 11577000 15911000 26644000 12124000 10821000 17 17 17 18,1 18,1 18,1 120,3 0 92,825 1529000000 105
A0A286ZV56;A0A288CG38;A0A287AP40;A0A287BIN3;A0A287B843;A0A286ZNE8
A0A286ZV56;A0A288CG38;A0A287AP40;A0A287BIN3;A0A287B843 137
tr|A0A286ZV56|A0A286ZV56_PIG Calcium voltage-gated channel auxiliary subunit alpha2delta 1 OS=Sus scrofa OX=9823 GN=CACNA2D1 PE=1 SV=2;tr|A0A288CG38|A0A288CG38_PIG Calcium voltage-gated channel auxiliary subunit alpha2delta 1 OS=Sus scrofa OX=9823 GN=CACNA
22923000 0 34613000 6966800 11846000 9376800 12296000 27126000 10679000 16903000 25922000 20320000 12 12 12 22,4 22,4 22,4 60,264 0 46,384 790640000 36
A0A286ZV66 A0A286ZV66 138 tr|A0A286ZV66|A0A286ZV66_PIG Uncharacterized protein OS=Sus scrofa OX=9823 PE=4 SV=2
7566600 5753700 4695600 12681000 0 6161500 10445000 0 8012600 15798000 5719100 5621000 3 3 3 14,6 14,6 14,6 24,91 0 8,427 137550000 17
A0A286ZV95 A0A286ZV95 139 tr|A0A286ZV95|A0A286ZV95_PIG Transmembrane emp24 domain-containing protein 10 OS=Sus scrofa OX=9823 GN=TMED10 PE=1 SV=1
0 0 0 0 0 0 0 0 0 0 0 0 4 2 2 27,7 16,9 16,9 18,518 0,0011641 2,8118 101710000 3
P10668;A0A286ZVE7;K7GK75;A0A5S6IF30;P60982;A0A5G2REA6;A0A5G2RAG2;A0A287BRC5 P10668;A0A286ZVE7;K7GK75 140 sp|P10668|COF1_PIG Cofilin-1 OS=Sus scrofa OX=9823 GN=CFL1 PE=1 SV=3;tr|A0A286ZVE7|A0A286ZVE7_PIG Cofilin, non-muscle isoform OS=Sus scrofa OX=9823 GN=CFL1 PE=1 SV=1;tr|K7GK75|K7GK75_PIG Cofilin, non-muscle isoform OS=Sus scrofa OX=9823 GN=CFL1 PE=1 SV=3

0 0 0 0 0 0 0 0 0 0 0 0 2 2 2 3,4 3,4 3,4

82,219 0 7,3084 49288000 4 A0A5G2RJW0;A0A287B571;A0A286ZVF6;F1S4E0

A0A5G2RJW0;A0A287B571;A0A286ZVF6;F1S4E0 141 tr|A0A5G2RJW0|A0A5G2RJW0_PIG GB1/RHD3-type G domain-containing protein OS=Sus scrofa OX=9823 GN=LOC102161784 PE=1 SV=1;tr|A0A287B571|A0A287B571_PIG GB1/RHD3-type G domain-containing protein OS=Sus scrofa OX=9823 GN=LOC102161784 PE=1 SV=2;tr|A0A286ZVF6|A0A2

41056000 9863100 17930000 50323000 14676000 16393000 51109000 7965300 10906000 23269000

9483200 15941000 5 5 5 10,5 10,5 10,5 50,099 0 10,69

623510000 24 I3LS26;F1SU97;A0A287A0D2;A0A286ZVG0

I3LS26;F1SU97;A0A287A0D2;A0A286ZVG0 142 tr|I3LS26|I3LS26_PIG Prosaposin OS=Sus scrofa OX=9823 GN=PSAP PE=1 SV=2;tr|F1SU97|F1SU97_PIG Prosaposin OS=Sus scrofa OX=9823 GN=PSAP PE=1 SV=2;tr|A0A287A0D2|A0A287A0D2_PIG Prosaposin OS=Sus scrofa OX=9823 GN=PSAP PE=1 SV=2;tr|A0A286ZVG0|A0A286ZVG0_PIG Sap

38659000 0 25753000 41602000 0 0 49581000 0 0 44990000 0 23718000

3 3 2 47,7 47,7 31,8 5,0526 0 17,598 537020000 27

Q95274;A0A286ZVG3;CON__P21752;A0A5S6G4N7 Q95274;A0A286ZVG3 143

sp|Q95274|TYB4_PIG Thymosin beta-4 OS=Sus scrofa OX=9823 GN=TMSB4 PE=3 SV=3;tr|A0A286ZVG3|A0A286ZVG3_PIG Thymosin beta OS=Sus scrofa OX=9823 GN=LOC110257905 PE=3 SV=1

3550200000 1010900000 607790000 2726700000 911840000 1306500000 2132100000

1507400000 834400000 1390900000 850510000 297060000 13 13

2 71,7 71,7 14,1 20,632 0 201,11 70407000000 1028 A0A287BJF1;A0A286ZVM3

A0A287BJF1;A0A286ZVM3 144 tr|A0A287BJF1|A0A287BJF1_PIG Myosin light chain 1 OS=Sus scrofa OX=9823 GN=MYL1 PE=1 SV=2;tr|A0A286ZVM3|A0A286ZVM3_PIG Myosin light chain 1 OS=Sus scrofa OX=9823 GN=MYL1 PE=1 SV=2

8092000 7334900 42246000 12132000 15638000 31055000 0 0 17183000 11202000 0 49837000

4 4 1 10,2 10,2 3,1 45,755 0 10,293 550310000 30

A0A286ZVQ0;A0A287BCT2;A0A287B5Z7 A0A286ZVQ0;A0A287BCT2;A0A287B5Z7 145

tr|A0A286ZVQ0|A0A286ZVQ0_PIG Uncharacterized protein OS=Sus scrofa OX=9823 GN=ACOT4 PE=1 SV=2;tr|A0A287BCT2|A0A287BCT2_PIG Uncharacterized protein OS=Sus scrofa OX=9823 GN=ACOT4 PE=1 SV=1;tr|A0A287B5Z7|A0A287B5Z7_PIG Uncharacterized protein OS=Sus scrofa O

7721300 3962100 4896700 0 3196200 2832300 0 0 3121100 2446200 0 3022700

5 5 5 13 13 13 41,713 0 6,6387 93911000 8

F1RZH8;I3LG37;A0A286ZVT0;A0A287BL28;A0A5G2QKP1;A0A287B9F9;A0A287A8V7;A0A5G2QGC9

F1RZH8;I3LG37;A0A286ZVT0;A0A287BL28;A0A5G2QKP1;A0A287B9F9;A0A287A8V7;A0A5G2QGC9 146 tr|F1RZH8|F1RZH8_PIG Endoplasmic reticulum junction formation protein lunapark OS=Sus scrofa OX=9823 GN=LNPK PE=1 SV=3;tr|I3LG37|I3LG37_PIG Endoplasmic reticulum junction formation protein lunapark OS=Sus scrofa OX=9823 GN=LNPK PE=1 SV=2;tr|A0A286ZVT0|A0A2

0 0 0 0 0 0 0 0 0 0 0 0 1 1 1 3,2 3,2 3,2

55,417 0 4,2113 49151000 2

A0A287A8H1;A0A287A4Y0;A0A287B231;A0A286ZWA5;A0A287AYH3

A0A287A8H1;A0A287A4Y0;A0A287B231;A0A286ZWA5;A0A287AYH3 147

tr|A0A287A8H1|A0A287A8H1_PIG Ubiquilin 1 OS=Sus scrofa OX=9823 GN=UBQLN1 PE=1 SV=2;tr|A0A287A4Y0|A0A287A4Y0_PIG Ubiquilin 1 OS=Sus scrofa OX=9823 GN=UBQLN1 PE=1 SV=1;tr|A0A287B231|A0A287B231_PIG Ubiquilin 1 OS=Sus scrofa OX=9823 GN=UBQLN1 PE=1 SV=2;tr|A0A2

0 0 0 0 0 0 0 0 0 0 0 0 2 2 2 3,1 3,1 3,1

72,165 0,0030426 1,7643 11290000 3 A0A286ZWG1;I3L829;A0A287B880

A0A286ZWG1;I3L829;A0A287B880 148 tr|A0A286ZWG1|A0A286ZWG1_PIG NPL4 homolog, ubiquitin recognition factor OS=Sus scrofa OX=9823 GN=NPLOC4 PE=1 SV=2;tr|I3L829|I3L829_PIG NPL4 homolog, ubiquitin recognition factor OS=Sus scrofa OX=9823 GN=NPLOC4 PE=1 SV=3;tr|A0A287B880|A0A287B880_PIG NPL4 ho

993890000 2989400000 1206400000 1417500000 1086300000 968170000 1248900000

2792200000 1736600000 969440000 1979100000 1061300000 9 2

2 20,9 7,1 7,1 39,376 0 169,95 39641000000 415

A0A286ZWI1;A0A287B8Z2;F1RJ25;A0A286ZZC9;A0A286ZTA4;A0A5G2RHD6

A0A286ZWI1;A0A287B8Z2 149 tr|A0A286ZWI1|A0A286ZWI1_PIG Fructose-bisphosphate aldolase OS=Sus scrofa OX=9823 GN=ALDOC PE=1 SV=1;tr|A0A287B8Z2|A0A287B8Z2_PIG Fructose-bisphosphate aldolase OS=Sus scrofa OX=9823 GN=ALDOC PE=1 SV=1

8249400 0 21457000 0 0 7779800 7234500 0 0 0 0 11138000 4

4 4 11,8 11,8 11,8 41,708 0 8,2714 151440000 8

F1SFR6;A0A287BA45;A0A287ABW8;P53590;A0A286ZWJ9

F1SFR6;A0A287BA45;A0A287ABW8;P53590;A0A286ZWJ9 150 tr|F1SFR6|F1SFR6_PIG Succinate--CoA ligase [GDP-forming] subunit beta, mitochondrial OS=Sus scrofa OX=9823 GN=SUCLG2 PE=4 SV=4;tr|A0A287BA45|A0A287BA45_PIG Succinate--CoA ligase [GDP-forming] subunit beta, mitochondrial OS=Sus scrofa OX=9823 GN=SUCLG2 PE=3

219450000 178880000 142650000 444690000 198600000 194750000 479170000

246290000 213440000 338200000 238880000 225350000 25 25

21 37,1 37,1 33 71,881 0 276,98 25832000000 564

F1S9Q3;F1S9Q1;A0A286ZWK2;A0A286ZPN4;A0A286ZPC8;A0A287APM9

F1S9Q3;F1S9Q1;A0A286ZWK2;A0A286ZPN4;A0A286ZPC8;A0A287APM9 151

tr|F1S9Q3|F1S9Q3_PIG Uncharacterized protein OS=Sus scrofa OX=9823 GN=HSPA8 PE=1 SV=3;tr|F1S9Q1|F1S9Q1_PIG Uncharacterized protein OS=Sus scrofa OX=9823 GN=HSPA8 PE=1 SV=4;tr|A0A286ZWK2|A0A286ZWK2_PIG Uncharacterized protein OS=Sus scrofa OX=9823 GN=HSPA8

5357400 7124100 0 0 6301900 7805200 0 6759700 7612100 0 5020000 0

3 3 3 5,1 5,1 5,1 67,583 0 5,4663 79158000 17 F1SUR3;A0A286ZWR7

F1SUR3;A0A286ZWR7 152 tr|F1SUR3|F1SUR3_PIG Phosphoglucomutase 2 like 1 OS=Sus scrofa OX=9823 GN=PGM2L1 PE=1 SV=3;tr|A0A286ZWR7|A0A286ZWR7_PIG Phosphoglucomutase 2 like 1 OS=Sus scrofa OX=9823 GN=PGM2L1 PE=1 SV=1

0 0 0 0 0 0 0 0 0 0 0 0 2 2 2 20,5 20,5 20,5

9,5346 0,0049801 1,6157 10984000 3 A0A5G2QTG5;A0A286ZWS0

A0A5G2QTG5;A0A286ZWS0 153 tr|A0A5G2QTG5|A0A5G2QTG5_PIG SH3 domain

binding glutamate rich protein like 3 OS=Sus scrofa OX=9823 GN=SH3BGRL3 PE=1 SV=1;tr|A0A286ZWS0|A0A286ZWS0_PIG SH3 domain-binding glutamic acid-rich-like protein OS=Sus scrofa OX=9823 GN=SH3BGRL3 PE=1 SV=1
15212000 14457000 0 0 8380200 0 9664000 0 0 0 0 0 3 3 3 11 11 11 32,315 0 9,1689 117710000 9 A0A5G2R9R8;A0A286ZWU9;F1RJI1;A0A5G2RBB7 A0A5G2R9R8;A0A286ZWU9;F1RJI1;A0A5G2RBB7 154 tr|A0A5G2R9R8|A0A5G2R9R8_PIG Malectin domain-containing protein OS=Sus scrofa OX=9823 GN=CABP1 PE=1 SV=1;tr|A0A286ZWU9|A0A286ZWU9_PIG Malectin domain-containing protein OS=Sus scrofa OX=9823 GN=CABP1 PE=1 SV=1;tr|F1RJI1|F1RJI1_PIG Uncharacterized protein O
11407000 5941500 7877700 16275000 7320600 8384100 20595000 4768600 6593500 22456000 8001100 10161000 4 4 4 13 13 13 37,294 0 13,281 335660000 19 P00795;A0A286ZX26 P00795;A0A286ZX26 155 sp|P00795|CATD_PIG Cathepsin D OS=Sus scrofa OX=9823 GN=CTSD PE=1 SV=2;tr|A0A286ZX26|A0A286ZX26_PIG Cathepsin D OS=Sus scrofa OX=9823 GN=CTSD PE=1 SV=2
12347000 9113900 8283400 17642000 9265300 10991000 10924000 11765000 14125000 15743000 10124000 7009700 5 5 5 36,8 36,8 36,8 14,984 0 7,5291 275260000 24 Q29308;F1RGG1;A0A286ZX70;I3LQK3 Q29308;F1RGG1;A0A286ZX70 156 sp|Q29308|RS19_PIG 40S ribosomal protein S19 (Fragment) OS=Sus scrofa OX=9823 GN=RPS19 PE=2 SV=3;tr|F1RGG1|F1RGG1_PIG 40S ribosomal protein S19 OS=Sus scrofa OX=9823 GN=RPS19 PE=1 SV=3;tr|A0A286ZX70|A0A286ZX70_PIG 40S ribosomal protein S19 OS=Sus scrofa OX
52326000 38968000 56237000 70390000 44771000 55976000 78448000 35728000 46392000 80142000 46901000 53248000 6 6 6 60,7 60,7 60,7 13,374 0 32,296 2421400000 72 A0A286ZXA4 A0A286ZXA4 157 tr|A0A286ZXA4|A0A286ZXA4_PIG Uncharacterized protein OS=Sus scrofa OX=9823 GN=AAMDC PE=1 SV=1
8447500 0 4594600 12252000 4457700 7310300 11838000 0 4575100 12298000 0 8893800 12 12 12 7,4 7,4 7,4 228,62 0 68,474 350480000 37 A0A287B5L5;A0A287A4H3;A0A287BBL5;A0A287BPN0;A0A287AY86;A0A287BAB8;A0A286ZXD9;A0A287A3X7;I3L806 A0A287B5L5;A0A287A4H3;A0A287BBL5;A0A287BPN0;A0A287AY86;A0A287BAB8;A0A286ZXD9;A0A287A3X7;I3L806 158 tr|A0A287B5L5|A0A287B5L5_PIG Microtubule-associated protein OS=Sus scrofa OX=9823 GN=MAP4 PE=1 SV=2;tr|A0A287A4H3|A0A287A4H3_PIG Microtubule-associated protein OS=Sus scrofa OX=9823 GN=MAP4 PE=1 SV=2;tr|A0A287BBL5|A0A287BBL5_PIG Microtubule-associated prot
159650000 370500000 591770000 209820000 620730000 708620000 232300000 287570000 445760000 184600000 463780000 1058600000 6 6 6 14 14 14 53,631 0 22,582 6510900000 30 A0A286ZXF1 A0A286ZXF1 159 tr|A0A286ZXF1|A0A286ZXF1_PIG Cytochrome-b5 reductase OS=Sus scrofa OX=9823 GN=CYB5R1 PE=1 SV=1
0 0 0 0 0 0 0 0 0 0 0 0 1 1 1 3,1 3,1 3,1 43,612 0 5,6012 14360000 2 F1SFE6;A0A286ZXG4;A0A287BG14 F1SFE6;A0A286ZXG4;A0A287BG14 160 tr|F1SFE6|F1SFE6_PIG Proteasome 26S subunit,

ATPase 6 OS=Sus scrofa OX=9823 GN=PSMC6 PE=1 SV=3;tr|A0A286ZXG4|A0A286ZXG4_PIG Proteasome 26S subunit, ATPase 6 OS=Sus scrofa OX=9823 GN=PSMC6 PE=1 SV=2;tr|A0A287BG14|A0A287BG14_PIG Proteasome 26S subunit, ATPas
0 0 0 0 0 0 0 0 0 0 0 0 1 1 1 1,3 1,3 1,3
92,419 0,0031283 1,9232 3669600 1 A0A287AKX9;A0A286ZXI4
A0A287AKX9;A0A286ZXI4 161 tr|A0A287AKX9|A0A287AKX9_PIG Uncharacterized protein OS=Sus scrofa OX=9823 PE=4 SV=1;tr|A0A286ZXI4|A0A286ZXI4_PIG Uncharacterized protein OS=Sus scrofa OX=9823 PE=4 SV=1
14728000 13120000 8658600 18047000 7505200 15393000 21857000 10198000 13626000 19396000
7315200 7241500 9 9 4 20,4 20,4 10,2 56,393 0 16,117
516280000 35
A0A286ZXJ6;A0A287BDA6;F1S100;A0A5G2QSM3;A0A5G2R911;A0A5G2QAP9
A0A286ZXJ6;A0A287BDA6;F1S100;A0A5G2QSM3;A0A5G2R911;A0A5G2QAP9 162
tr|A0A286ZXJ6|A0A286ZXJ6_PIG Serine/threonine-protein phosphatase OS=Sus scrofa OX=9823 GN=PPP3CA PE=1 SV=2;tr|A0A287BDA6|A0A287BDA6_PIG Serine/threonine-protein phosphatase OS=Sus scrofa OX=9823 GN=PPP3CA PE=1 SV=1;tr|F1S100|F1S100_PIG Serine/threonine-pr
0 0 0 0 0 0 0 0 0 0 0 0 3 3 3 1,5 1,5 1,5
243,69 0 2,9931 0 3
A0A287B1J4;F1RR78;A0A287AMR4;A0A287BJQ1;A0A286ZXS0
A0A287B1J4;F1RR78;A0A287AMR4;A0A287BJQ1;A0A286ZXS0 163
tr|A0A287B1J4|A0A287B1J4_PIG Spectrin alpha, non-erythrocytic 1 OS=Sus scrofa OX=9823 GN=SPTAN1 PE=1 SV=2;tr|F1RR78|F1RR78_PIG Spectrin alpha, non-erythrocytic 1 OS=Sus scrofa OX=9823 GN=SPTAN1 PE=1 SV=4;tr|A0A287AMR4|A0A287AMR4_PIG Spectrin alpha, non-ery
0 0 0 0 0 0 0 0 0 0 0 0 10 5 5 4,1 2,4 2,4
281,07 0 10,584 28552000 5
A0A286ZXU2;A0A287A5T4;A0A287A4W0;A0A287AW81
A0A286ZXU2;A0A287A5T4;A0A287A4W0;A0A287AW81 164
tr|A0A286ZXU2|A0A286ZXU2_PIG Filamin A OS=Sus scrofa OX=9823 GN=FLNA PE=1 SV=2;tr|A0A287A5T4|A0A287A5T4_PIG Filamin A OS=Sus scrofa OX=9823 GN=FLNA PE=1 SV=1;tr|A0A287A4W0|A0A287A4W0_PIG Filamin A OS=Sus scrofa OX=9823 GN=FLNA PE=1 SV=1;tr|A0A287AW81|A0A28
26240000 10905000 17174000 0 10555000 14471000 10094000 11280000 12113000 17331000 8369200
16374000 4 4 4 10,2 10,2 10,2 45,515 0 9,6251 521210000 27
A0A286ZXU8;A0A287B283 A0A286ZXU8;A0A287B283 165
tr|A0A286ZXU8|A0A286ZXU8_PIG LanC like 1 OS=Sus scrofa OX=9823 GN=LANCL1 PE=1 SV=1;tr|A0A287B283|A0A287B283_PIG LanC like 1 OS=Sus scrofa OX=9823 GN=LANCL1 PE=1 SV=2
19179000 15886000 19840000 8795000 9343400 16026000 11941000 7763700 6883400 8855600
8238600 5878900 4 4 4 13 13 13 40,614 0 10,696
305930000 19 A0A286ZY32;A0A287AZ05 A0A286ZY32;A0A287AZ05 166
tr|A0A286ZY32|A0A286ZY32_PIG Muscle-restricted coiled-coil protein OS=Sus scrofa

OX=9823 GN=CAVIN4 PE=1 SV=1;tr|A0A287AZ05|A0A287AZ05_PIG Muscle-restricted coiled-coil protein OS=Sus scrofa OX=9823 GN=CAVIN4 PE=1 SV=1
0 0 0 0 0 0 0 0 0 0 0 0 1 1 1 1,1 1,1 1,1
120,94 0 3,1498 0 2 A0A5G2QFV1;A0A287AHE4;A0A5G2RFV8;A0A286ZY33
A0A5G2QFV1;A0A287AHE4;A0A5G2RFV8;A0A286ZY33 167
tr|A0A5G2QFV1|A0A5G2QFV1_PIG Integrin subunit alpha 7 OS=Sus scrofa OX=9823 GN=ITGA7 PE=3 SV=1;tr|A0A287AHE4|A0A287AHE4_PIG Integrin subunit alpha 7 OS=Sus scrofa OX=9823 GN=ITGA7 PE=3 SV=2;tr|A0A5G2RFV8|A0A5G2RFV8_PIG Integrin subunit alpha 7 OS=Sus scrof
0 0 0 0 0 0 0 0 0 0 0 0 3 3 3 4,4 4,4 4,4
90,885 0 19,93 128350000 10
A0A286ZY59;Q29243;A0A2C9F3F8;A0A287BAM0;A0A287BBR1
A0A286ZY59;Q29243;A0A2C9F3F8;A0A287BAM0;A0A287BBR1 168
tr|A0A286ZY59|A0A286ZY59_PIG Alpha-dystroglycan OS=Sus scrofa OX=9823 GN=DAG1 PE=1 SV=1;sp|Q29243|DAG1_PIG Dystroglycan 1 OS=Sus scrofa OX=9823 GN=DAG1 PE=2 SV=2;tr|A0A2C9F3F8|A0A2C9F3F8_PIG Alpha-dystroglycan OS=Sus scrofa OX=9823 GN=DAG1 PE=1 SV=2;tr|A0A
63985000 130990000 341790000 61061000 239740000 275310000 37066000 131200000
207870000 62079000 148060000 464180000 18 18 16 44,2 44,2 40,4
47,526 0 246,91 15434000000 312 P33198;A0A287BL23;A0A286ZYE6;F1SK00
P33198;A0A287BL23;A0A286ZYE6 169 sp|P33198|IDHP_PIG Isocitrate dehydrogenase [NADP], mitochondrial (Fragment) OS=Sus scrofa OX=9823 GN=IDH2 PE=1 SV=1;tr|A0A287BL23|A0A287BL23_PIG Isocitrate dehydrogenase [NADP] OS=Sus scrofa OX=9823 GN=IDH2 PE=3 SV=1;tr|A0A286ZYE6|A0A286ZYE6_PIG Isocitrat
146410000 54316000 133020000 273740000 129030000 128270000 243650000
59851000 96355000 282460000 120210000 220350000 2 2 2 13,7
13,7 13,7 17,475 0 10,842 3501300000 49 A0A286ZYL7;A0A5G2RN51
A0A286ZYL7;A0A5G2RN51170 tr|A0A286ZYL7|A0A286ZYL7_PIG ATP synthase F1 subunit delta OS=Sus scrofa OX=9823 GN=ATP5F1D PE=1 SV=1;tr|A0A5G2RN51|A0A5G2RN51_PIG ATP synthase F1 subunit delta OS=Sus scrofa OX=9823 GN=ATP5F1D PE=1 SV=1
81657000 71602000 151980000 131910000 164460000 158720000 105840000 78195000
99407000 119950000 170770000 294370000 8 8 8 33,8 33,8 33,8
22,169 0 71,237 5792100000 129
A0A5G2R4S0;A0A5G2RAC9;A0A5G2R1V1;A0A286ZYM6
A0A5G2R4S0;A0A5G2RAC9;A0A5G2R1V1;A0A286ZYM6 171
tr|A0A5G2R4S0|A0A5G2R4S0_PIG ATP synthase subunit b OS=Sus scrofa OX=9823 GN=ATP5PB PE=1 SV=1;tr|A0A5G2RAC9|A0A5G2RAC9_PIG ATP synthase subunit b OS=Sus scrofa OX=9823 GN=ATP5PB PE=1 SV=1;tr|A0A5G2R1V1|A0A5G2R1V1_PIG ATP synthase subunit b OS=Sus scrofa OX
0 0 0 6313100 0 0 8208300 0 0 3543700 0 0 3 3 3
5,1 5,1 5,1 70,203 0 2,919 49588000 11
A0A286ZYQ4;A0A287ANV7;F1RK02;A0A5K1TXL2;K7GQS5;F1RWT2;A0A5K1TXL1
A0A286ZYQ4;A0A287ANV7;F1RK02;A0A5K1TXL2;K7GQS5;F1RWT2;A0A5K1TXL1 172

tr|A0A286ZYQ4|A0A286ZYQ4_PIG Lymphocyte cytosolic protein 1 OS=Sus scrofa OX=9823 GN=LCP1 PE=1 SV=1;tr|A0A287ANV7|A0A287ANV7_PIG Lymphocyte cytosolic protein 1 OS=Sus scrofa OX=9823 GN=LCP1 PE=1 SV=1;tr|F1RK02|F1RK02_PIG Lymphocyte cytosolic protein 1 OS=S
7675000 5267200 6874000 0 0 4843100 0 0 4880000 4736500 0 0
6 6 6 17,6 17,6 17,6 38,044 0 11,828 111480000 12
F1SPH1;A0A286ZYQ6;I3LVF5;A0A287ACW2 F1SPH1;A0A286ZYQ6 173
tr|F1SPH1|F1SPH1_PIG Peptidase_M24 domain-containing protein OS=Sus scrofa OX=9823 GN=PA2G4 PE=1 SV=3;tr|A0A286ZYQ6|A0A286ZYQ6_PIG Peptidase_M24 domain-containing protein OS=Sus scrofa OX=9823 GN=PA2G4 PE=1 SV=1
0 0 0 0 0 0 0 0 0 0 0 0 2 2 2 3,2 3,2 3,2
53,499 0,0059113 1,5227 14828000 3 A0A286ZYS2 A0A286ZYS2 174
tr|A0A286ZYS2|A0A286ZYS2_PIG Aspartate--tRNA ligase, cytoplasmic OS=Sus scrofa OX=9823 GN=DARS1 PE=1 SV=1
21037000 6842600 8385700 11997000 10574000 0 27091000 3982700 5470500 13326000 4045500
8691900 15 15 15 5,2 5,2 5,2 437,21 0 54,849 404040000 47
I3LPD6;A0A286ZYS8 I3LPD6;A0A286ZYS8 175 tr|I3LPD6|I3LPD6_PIG Cardiomyopathy associated 5 OS=Sus scrofa OX=9823 GN=CMYA5 PE=1 SV=2;tr|A0A286ZYS8|A0A286ZYS8_PIG Cardiomyopathy associated 5 OS=Sus scrofa OX=9823 GN=CMYA5 PE=1 SV=2
6312500000 13643000000 5917400000 8526600000 11822000000 7750800000 9800700000
11480000000 7806100000 4486700000 10841000000 4724100000 28 28
3 67,1 67,1 10,6 39,817 0 323,31 780680000000 2790 A0A286ZYX8 A0A286ZYX8 176
tr|A0A286ZYX8|A0A286ZYX8_PIG Fructose-bisphosphate aldolase OS=Sus scrofa OX=9823 GN=ALDOA PE=1 SV=2
0 0 0 0 0 0 0 0 0 0 0 0 2 2 2 5,4 5,4 5,4
36,271 0,0030801 1,8302 25304000 5
A0A286ZZ18;A0A287B1J8;F1S3B4;A0A287A426;A0A287BIE8
A0A286ZZ18;A0A287B1J8;F1S3B4;A0A287A426;A0A287BIE8 177
tr|A0A286ZZ18|A0A286ZZ18_PIG IST1 homolog OS=Sus scrofa OX=9823 GN=IST1 PE=1 SV=1;tr|A0A287B1J8|A0A287B1J8_PIG IST1 homolog OS=Sus scrofa OX=9823 GN=IST1 PE=1 SV=1;tr|F1S3B4|F1S3B4_PIG IST1 homolog OS=Sus scrofa OX=9823 GN=IST1 PE=1 SV=3;tr|A0A287A426|A0A2
37718000 16987000 47200000 57983000 39432000 44582000 58587000 19371000 32429000 65669000
29354000 58956000 6 6 6 31,2 31,2 31,2 26,019 0 26,752
1566300000 52 F6PVY0;A0A286ZZ87 F6PVY0;A0A286ZZ87 178
tr|F6PVY0|F6PVY0_PIG Fatty acid binding protein 5 OS=Sus scrofa OX=9823 GN=FABP5 PE=1 SV=1;tr|A0A286ZZ87|A0A286ZZ87_PIG Fatty acid binding protein 5 OS=Sus scrofa OX=9823 GN=FABP5 PE=1 SV=1
34564000 37404000 54721000 10311000 12992000 22995000 6756300 7609200 8315100 9362800
9754500 11427000 9 9 9 20,3 20,3 20,3 50,365 0 28,184
784490000 26 A0A286ZZ97;A0A287A2G4 A0A286ZZ97;A0A287A2G4 179
tr|A0A286ZZ97|A0A286ZZ97_PIG Caveolae associated protein 1 OS=Sus scrofa OX=9823 GN=CAVIN1 PE=3 SV=1;tr|A0A287A2G4|A0A287A2G4_PIG Caveolae associated protein 1 OS=Sus scrofa OX=9823 GN=CAVIN1 PE=3 SV=1

0 0 0 0 0 0 0 0 0 0 0 0 4 1 1 19,3 4,3 4,3
21,026 0,0086789 1,4055 280710000 11 A0A286ZZB7;F6PYW2;P06348
A0A286ZZB7;F6PYW2 180 tr|A0A286ZZB7|A0A286ZZB7_PIG H1.4 linker histone, cluster member OS=Sus scrofa OX=9823 GN=H1-4 PE=3 SV=2;tr|F6PYW2|F6PYW2_PIG H1.3 linker histone, cluster member OS=Sus scrofa OX=9823 GN=H1-3 PE=1 SV=1
0 0 13239000 0 0 8653900 0 0 0 0 0 10602000 3 3 3
10,3 10,3 10,3 32,915 0 9,5542 45933000 9 A0A286ZZC3;A0A5G2QRC2;F1S2X3
A0A286ZZC3;A0A5G2QRC2;F1S2X3 181 tr|A0A286ZZC3|A0A286ZZC3_PIG Ethylmalonyl-CoA decarboxylase 1 OS=Sus scrofa OX=9823 GN=ECHDC1 PE=1 SV=1;tr|A0A5G2QRC2|A0A5G2QRC2_PIG Ethylmalonyl-CoA decarboxylase 1 OS=Sus scrofa OX=9823 GN=ECHDC1 PE=1 SV=1;tr|F1S2X3|F1S2X3_PIG Ethylmalonyl-CoA decarboxyl
5484900 0 8043000 0 0 0 0 0 7511800 6270600 0 0 2 2
2 18,7 18,7 18,7 14,078 0 3,6846 93516000 12 A0A286ZZM7;A0A5G2QQH2
A0A286ZZM7;A0A5G2QQH2 182 tr|A0A286ZZM7|A0A286ZZM7_PIG Thioredoxin domain-containing protein 17 OS=Sus scrofa OX=9823 GN=TXNDC17 PE=1 SV=1;tr|A0A5G2QQH2|A0A5G2QQH2_PIG Thioredoxin domain-containing protein 17 OS=Sus scrofa OX=9823 GN=TXNDC17 PE=1 SV=1
0 0 0 0 0 0 0 0 0 0 0 0 3 3 3 11,3 11,3 11,3
33,138 0 8,6023 29656000 8 A0A5G2QPY2;A0A287A1V9;A0A286ZZM8
A0A5G2QPY2;A0A287A1V9;A0A286ZZM8 183 tr|A0A5G2QPY2|A0A5G2QPY2_PIG Dehydrogenase/reductase 7B OS=Sus scrofa OX=9823 GN=DHRS7B PE=3 SV=1;tr|A0A287A1V9|A0A287A1V9_PIG Dehydrogenase/reductase 7B OS=Sus scrofa OX=9823 GN=DHRS7B PE=3 SV=2;tr|A0A286ZZM8|A0A286ZZM8_PIG Dehydrogenase/reductase 7B OS=S
0 0 0 0 0 0 0 0 0 0 0 0 2 2 2 6,5 6,5 6,5
48,909 0 3,6175 0 3 A0A286ZZV4;A0A287A7Q7 A0A286ZZV4;A0A287A7Q7 184
tr|A0A286ZZV4|A0A286ZZV4_PIG Eukaryotic translation initiation factor 5 OS=Sus scrofa OX=9823 GN=EIF5 PE=3 SV=1;tr|A0A287A7Q7|A0A287A7Q7_PIG Eukaryotic translation initiation factor 5 OS=Sus scrofa OX=9823 GN=EIF5 PE=3 SV=1
20305000 10322000 11784000 18557000 11047000 16027000 18491000 6825100 12348000 13850000
10842000 14940000 3 3 2 14,4 14,4 10,9 32,694 0 27,85
487580000 27 F1SQ46;A0A286ZZW7;I3LQF8;A0A5G2QPK7
F1SQ46;A0A286ZZW7;I3LQF8;A0A5G2QPK7 185 tr|F1SQ46|F1SQ46_PIG Y-box binding protein 3 OS=Sus scrofa OX=9823 GN=YBX3 PE=1 SV=2;tr|A0A286ZZW7|A0A286ZZW7_PIG Y-box binding protein 3 OS=Sus scrofa OX=9823 GN=YBX3 PE=1 SV=1;tr|I3LQF8|I3LQF8_PIG Y-box binding protein 3 OS=Sus scrofa OX=9823 GN=YBX3 PE=
0 0 0 0 0 0 0 15491000 18388000 0 0 0 2 2 2 20,4
20,4 20,4 11,873 0 3,8701 188070000 11 A0A287A021 A0A287A021 186
tr|A0A287A021|A0A287A021_PIG Nuclear transport factor 2 OS=Sus scrofa OX=9823 GN=NUTF2 PE=1 SV=1
0 0 0 0 0 0 0 0 0 0 0 0 1 1 1 3,4 3,4 3,4
67,603 0,0011574 2,7817 0 1
I3LBE2;A0A287AT40;A0A287A456;A0A287AKT7;A0A287A026;A0A5G2QRI8;I3LPP8;A0A287A8X4

I3LBE2;A0A287AT40;A0A287A456;A0A287AKT7;A0A287A026;A0A5G2QRI8;I3LPP8;A0A287A8X4 187 tr|I3LBE2|I3LBE2_PIG Protein arginine N-methyltransferase 5 OS=Sus scrofa OX=9823 GN=PRMT5 PE=1 SV=1;tr|A0A287AT40|A0A287AT40_PIG Protein arginine N-methyltransferase 5 OS=Sus scrofa OX=9823 GN=PRMT5 PE=1 SV=2;tr|A0A287A456|A0A287A456_PIG Protein arginine
8185100 0 0 25733000 8518300 17335000 17407000 10162000 13204000 26144000 13261000
10559000 15 15 15 8,8 8,8 8,8 218,77 0 86,746 523730000 62
A0A287A502;A0A287AP44;A0A287A027 A0A287A502;A0A287AP44;A0A287A027 188
tr|A0A287A502|A0A287A502_PIG Uncharacterized protein OS=Sus scrofa OX=9823 GN=LAMA2 PE=1 SV=1;tr|A0A287AP44|A0A287AP44_PIG Uncharacterized protein OS=Sus scrofa OX=9823 GN=LAMA2 PE=1 SV=1;tr|A0A287A027|A0A287A027_PIG Uncharacterized protein OS=Sus scrofa O
4559000 0 1427500 21528000 4759300 10822000 14446000 4338300 9061800 19876000 7479200
6930000 20 20 20 15,2 15,2 15,2 177,54 0 85,141 845630000 87
A0A287A078;F1S663;A0A5G2REQ7 A0A287A078;F1S663 189
tr|A0A287A078|A0A287A078_PIG Laminin subunit gamma 1 OS=Sus scrofa OX=9823 GN=LAMC1 PE=1 SV=2;tr|F1S663|F1S663_PIG Laminin subunit gamma 1 OS=Sus scrofa OX=9823 GN=LAMC1 PE=1 SV=3
0 0 0 0 0 0 0 0 0 0 0 0 4 4 4 14,6 14,6 14,6
36,108 0 7,7508 6468400 4 A0A287A096 A0A287A096 190
tr|A0A287A096|A0A287A096_PIG Eukaryotic translation initiation factor 2 subunit 1 OS=Sus scrofa OX=9823 GN=EIF2S1 PE=1 SV=1
0 0 0 0 0 0 0 0 0 0 0 0 1 1 1 6,1 6,1 6,1
20,125 0 3,1047 56491000 7 A0A5G2QLG0;A0A480Q2F5;A0A287A0B5
A0A5G2QLG0;A0A480Q2F5;A0A287A0B5 191 tr|A0A5G2QLG0|A0A5G2QLG0_PIG 5-demethoxyubiquinone hydroxylase, mitochondrial OS=Sus scrofa OX=9823 GN=COQ7 PE=3 SV=1;tr|A0A480Q2F5|A0A480Q2F5_PIG 5-demethoxyubiquinone hydroxylase, mitochondrial OS=Sus scrofa OX=9823 GN=COQ7 PE=3 SV=1;tr|A0A287A0B5|A0A287
4087900 14571000 25313000 2718100 15566000 35002000 2304000 16446000 28431000 2522200
18732000 20483000 7 7 7 14,3 14,3 14,3 56,692 0 17,772
744000000 42 F1SRF0;A0A287A0H7 F1SRF0;A0A287A0H7 192 tr|F1SRF0|F1SRF0_PIG Hedgehog acyltransferase like OS=Sus scrofa OX=9823 GN=HHATL PE=1 SV=1;tr|A0A287A0H7|A0A287A0H7_PIG Hedgehog acyltransferase like OS=Sus scrofa OX=9823 GN=HHATL PE=1 SV=1
28169000 10581000 14812000 17624000 10188000 16016000 25682000 0 11241000 25147000 9382300
7677300 2 2 2 12,9 12,9 12,9 19,3 0 15,517 315650000 25
A0A287A0I0;A0A5G2RDF7;A0A5G2QGP8;A0A287ACI6;A0A5G2Q9D7
A0A287A0I0;A0A5G2RDF7;A0A5G2QGP8;A0A287ACI6;A0A5G2Q9D7 193
tr|A0A287A0I0|A0A287A0I0_PIG Uncharacterized protein OS=Sus scrofa OX=9823 GN=PPP3R1 PE=1 SV=2;tr|A0A5G2RDF7|A0A5G2RDF7_PIG Uncharacterized protein OS=Sus scrofa OX=9823 GN=PPP3R1 PE=1 SV=1;tr|A0A5G2QGP8|A0A5G2QGP8_PIG Uncharacterized protein OS=Sus scrofa

25198000 20929000 14837000 60801000 26231000 30898000 57165000 21429000 22131000 38897000 21184000 22538000 25 25 25 40,3 40,3 40,3 62,238 0 47,524 2901300000 145 A0A287A0Q8;I3LNG8 A0A287A0Q8;I3LNG8 194 tr|A0A287A0Q8|A0A287A0Q8_PIG Stress induced phosphoprotein 1 OS=Sus scrofa OX=9823 GN=STIP1 PE=1 SV=1;tr|I3LNG8|I3LNG8_PIG Stress induced phosphoprotein 1 OS=Sus scrofa OX=9823 GN=STIP1 PE=1 SV=2

576930 46226000 17811000 0 1016000 0 0 921600 0 0 1245500 710360 12 12 12 11,3 11,3 11,3 118,43 0 25,616 418970000 24 A0A287A0V9;F1RP07;A0A287AGE8;A0A287BH45 A0A287A0V9;F1RP07;A0A287AGE8;A0A287BH45 195 tr|A0A287A0V9|A0A287A0V9_PIG Phosphorylase b kinase regulatory subunit OS=Sus scrofa OX=9823 GN=PHKB PE=1 SV=2;tr|F1RP07|F1RP07_PIG Phosphorylase b kinase regulatory subunit OS=Sus scrofa OX=9823 GN=PHKB PE=1 SV=4;tr|A0A287AGE8|A0A287AGE8_PIG Phosphorylase

0 0 0 5775800 0 4287700 4702800 0 6090700 7158500 3264500 0 2 2 2 2,9 2,9 2,9 101,25 0 5,6454 59203000 6 A0A287A0X6 A0A287A0X6 196 tr|A0A287A0X6|A0A287A0X6_PIG Uncharacterized protein OS=Sus scrofa OX=9823 PE=1 SV=2

0 0 0 0 0 0 0 0 0 0 0 0 2 2 2 12,8 12,8 12,8 26,04 0 5,792 6694900 3 A0A287A2E2;A0A287A0X8;A0A287A6C1 A0A287A2E2;A0A287A0X8;A0A287A6C1 197 tr|A0A287A2E2|A0A287A2E2_PIG Methyltransf_11 domain-containing protein OS=Sus scrofa OX=9823 PE=4 SV=2;tr|A0A287A0X8|A0A287A0X8_PIG Methyltransf_11 domain-containing protein OS=Sus scrofa OX=9823 PE=4 SV=2;tr|A0A287A6C1|A0A287A6C1_PIG Methyltransf_11 domai

0 0 0 0 0 0 0 0 0 0 0 0 1 1 1 17,9 17,9 17,9 9,5211 0 5,4661 0 1 A0A287A0Y1;A0A5G2QK10A0A287A0Y1;A0A5G2QK10198 tr|A0A287A0Y1|A0A287A0Y1_PIG Uncharacterized protein OS=Sus scrofa OX=9823 GN=PFDN5 PE=1 SV=1;tr|A0A5G2QK10|A0A5G2QK10_PIG Uncharacterized protein OS=Sus scrofa OX=9823 GN=PFDN5 PE=1 SV=1

0 0 0 0 0 0 0 0 0 0 0 0 1 1 1 2 2 2 63,07 0 2,9132 18608000 5 A0A287A133;F1S1X3 A0A287A133;F1S1X3 199 tr|A0A287A133|A0A287A133_PIG Asparagine--tRNA ligase OS=Sus scrofa OX=9823 GN=NARS1 PE=1 SV=1;tr|F1S1X3|F1S1X3_PIG Asparagine--tRNA ligase OS=Sus scrofa OX=9823 GN=NARS1 PE=1 SV=3

0 0 0 0 0 0 0 0 0 0 0 0 2 1 1 24,3 14,8 14,8 12,532 0 3,0187 17025000 2 A0A287A145;A0A287BI08;P68211;A0A5G2R5M0 A0A287A145;A0A287BI08;P68211;A0A5G2R5M0 200 tr|A0A287A145|A0A287A145_PIG Alpha-endosulfine OS=Sus scrofa OX=9823 GN=ENSA PE=3 SV=1;tr|A0A287BI08|A0A287BI08_PIG Alpha-endosulfine OS=Sus scrofa OX=9823 GN=ENSA PE=3 SV=1;sp|P68211|ENSA_PIG Alpha-endosulfine OS=Sus scrofa OX=9823 GN=ENSA PE=1 SV=1;tr|A0

6380200 4409000 6559900 6520300 3399100 5779700 6761500 0 3497600 6305000 4720900 5786500 4 4 4 16,8 16,8 16,8 32,838 0 14,635 117110000 16 A0A287A1I0;A0A287BPC7;I3LCJ3;A0A287APY8 A0A287A1I0;A0A287BPC7;I3LCJ3;A0A287APY8 201 tr|A0A287A1I0|A0A287A1I0_PIG 5-

nucleotidase OS=Sus scrofa OX=9823 GN=NT5C3A PE=1 SV=1;tr|A0A287BPC7|A0A287BPC7_PIG 5-nucleotidase OS=Sus scrofa OX=9823 GN=NT5C3A PE=1 SV=1;tr|I3LCJ3|I3LCJ3_PIG 5-nucleotidase OS=Sus scrofa OX=9823 GN=NT5C3A PE=1 SV=3;tr|

0 0 0 0 0 0 0 0 0 0 0 0 2 2 2 1,9 1,9 1,9

138,05 0 11,247 11274000 6 A0A287BLD2;A0A287A1S6;A0A5G2QQE9

A0A287BLD2;A0A287A1S6;A0A5G2QQE9 202 tr|A0A287BLD2|A0A287BLD2_PIG Collagen type I alpha 1 chain OS=Sus scrofa OX=9823 GN=COL1A1 PE=1 SV=1;tr|A0A287A1S6|A0A287A1S6_PIG Collagen alpha-1(I) chain preproprotein OS=Sus scrofa OX=9823 GN=COL1A1 PE=1 SV=1;tr|A0A5G2QQE9|A0A5G2QQE9_PIG Collagen type I

0 61580000 0 0 0 0 0 2098900000 304340000 0 16875000 0

27 2 0 46,1 5,5 0 55,384 0 68,889 3375600000 26

A0A287A1V5;A0A287BFY0 A0A287A1V5;A0A287BFY0 203

tr|A0A287A1V5|A0A287A1V5_PIG Fructose-bisphosphate aldolase OS=Sus scrofa OX=9823 GN=ALDOA PE=1 SV=2;tr|A0A287BFY0|A0A287BFY0_PIG Fructose-bisphosphate aldolase OS=Sus scrofa OX=9823 GN=ALDOA PE=1 SV=1

0 0 0 0 0 0 8374100 0 0 8153800 0 0 3 3 3 14,8

14,8 14,8 31,267 0 4,8005 55982000 8

F1S8G6;A0A287A1V6;A0A5G2R4Y3;A0A5G2QXK3

F1S8G6;A0A287A1V6;A0A5G2R4Y3;A0A5G2QXK3 204 tr|F1S8G6|F1S8G6_PIG Small glutamine rich tetratricopeptide repeat containing alpha OS=Sus scrofa OX=9823 GN=SGTA PE=1 SV=2;tr|A0A287A1V6|A0A287A1V6_PIG Small glutamine rich tetratricopeptide repeat containing alpha OS=Sus scrofa OX=9823 GN=SGTA PE=1 SV=1;

0 9131700 11613000 0 0 0 0 0 0 0 0 0 5 5 5 11,1

11,1 11,1 60,952 0 25,529 62381000 9

A0A287A217;A0A287A275;A0A5G2QGK1;A0A287AGU7;A0A287A6Y9;A0A287AZ37;F2Z5B2;P02554;A0A287B5H7;A0A287AHL4;Q767L7;F2Z5K5;A0A5G2QHG1;A0A287A0J1;A0A5G2QJF0;A0A287B749;A0A5G2RIM4;F1S6M7;A0A5G2R693;A0A5G2QDX3

A0A287A217;A0A287A275;A0A5G2QGK1;A0A287AGU7;A0A287A6Y9;A0A287AZ37;F2Z5B2;P02554;A0A287B5H7;A0A287AHL4;Q767L7;F2Z5K5;A0A5G2QHG1;A0A287A0J1 205

tr|A0A287A217|A0A287A217_PIG Tubulin beta 4B class IVb OS=Sus scrofa OX=9823 GN=TUBB4B PE=1 SV=2;tr|A0A287A275|A0A287A275_PIG Tubulin beta chain OS=Sus scrofa OX=9823 GN=TUBB4B PE=1 SV=2;tr|A0A5G2QGK1|A0A5G2QGK1_PIG Tubulin beta chain OS=Sus scrofa OX=9823

0 0 3432600 0 0 3652600 0 0 0 0 0 0 2 2 2 5,9

5,9 5,9 41,942 0 3,383 20787000 4

A0A287A248;I3LIW1;A0A287A9G0;A0A287AQ33

A0A287A248;I3LIW1;A0A287A9G0;A0A287AQ33 206 tr|A0A287A248|A0A287A248_PIG LanC like 2 OS=Sus scrofa OX=9823 GN=LANCL2 PE=1 SV=1;tr|I3LIW1|I3LIW1_PIG LanC like 2 OS=Sus scrofa OX=9823 GN=LANCL2 PE=1 SV=2;tr|A0A287A9G0|A0A287A9G0_PIG LanC like 2 OS=Sus scrofa OX=9823 GN=LANCL2 PE=1 SV=1;tr|A0A287AQ33|A

0 0 0 0 0 0 0 0 0 0 0 0 1 1 1 1,9 1,9 1,9

81,121 0,0049652 1,6025 6083800 5 F1SR61;A0A287A2J9 F1SR61;A0A287A2J9

207 tr|F1SR61|F1SR61_PIG Cullin 3 OS=Sus scrofa OX=9823 GN=CUL3 PE=1 SV=3;tr|A0A287A2J9|A0A287A2J9_PIG Cullin 3 OS=Sus scrofa OX=9823 GN=CUL3 PE=1 SV=2
0 0 0 0 0 0 0 0 0 0 0 0 2 2 2 9,9 9,9 9,9
20,702 0 4,158 40964000 3 A0A287A2R1;A0A5G2RFW9
A0A287A2R1;A0A5G2RFW9 208 tr|A0A287A2R1|A0A287A2R1_PIG Mesencephalic astrocyte derived neurotrophic factor OS=Sus scrofa OX=9823 GN=MANF PE=1 SV=1;tr|A0A5G2RFW9|A0A5G2RFW9_PIG Mesencephalic astrocyte derived neurotrophic factor OS=Sus scrofa OX=9823 GN=MANF PE=1 SV=1
214250000 135170000 126910000 45959000 94491000 30804000 90045000 35825000 36973000
54152000 35856000 61430000 13 10 2 49,4 39,6 8,2 29,174 0 49,011
5054600000 165
A0A287A2R9;A0A5G2QSR7;A0A5G2QK17;A0A5G2QJ52;A0A5G2Q6S6;A0A5G2QI44
A0A287A2R9;A0A5G2QSR7;A0A5G2QK17;A0A5G2QJ52 209
tr|A0A287A2R9|A0A287A2R9_PIG 14_3_3 domain-containing protein OS=Sus scrofa OX=9823 GN=CRK PE=1 SV=1;tr|A0A5G2QSR7|A0A5G2QSR7_PIG 14_3_3 domain-containing protein OS=Sus scrofa OX=9823 GN=CRK PE=1 SV=1;tr|A0A5G2QK17|A0A5G2QK17_PIG 14_3_3 domain-containing
78092000 42851000 116550000 153720000 102540000 99913000 136950000 48076000
90049000 188400000 114590000 190950000 8 8 8 46 46 46
18,515 0 18,164 5839700000 86
A0A287A2Y4;A0A5G2QY12;A0A287B0C3;A0A287B4I0;F1SMF9
A0A287A2Y4;A0A5G2QY12;A0A287B0C3;A0A287B4I0;F1SMF9 210
tr|A0A287A2Y4|A0A287A2Y4_PIG ATP synthase subunit d, mitochondrial OS=Sus scrofa OX=9823 GN=ATP5PD PE=1 SV=1;tr|A0A5G2QY12|A0A5G2QY12_PIG ATP synthase subunit d, mitochondrial OS=Sus scrofa OX=9823 GN=ATP5PD PE=1 SV=1;tr|A0A287B0C3|A0A287B0C3_PIG ATP synth
0 0 8248900 0 0 7222900 0 0 0 0 0 11722000 2 2 2
4,7 4,7 4,7 45,792 0,0032051 2,0881 54050000 5
A0A287A310;A0A5G2Q7D1;A0A5G2RBP2;F1SH10;A0A286ZLI9;A0A287AIJ9
A0A287A310;A0A5G2Q7D1;A0A5G2RBP2;F1SH10;A0A286ZLI9;A0A287AIJ9 211
tr|A0A287A310|A0A287A310_PIG Neutral cholesterol ester hydrolase 1 OS=Sus scrofa OX=9823 GN=NCEH1 PE=1 SV=1;tr|A0A5G2Q7D1|A0A5G2Q7D1_PIG Neutral cholesterol ester hydrolase 1 OS=Sus scrofa OX=9823 GN=NCEH1 PE=1 SV=1;tr|A0A5G2RBP2|A0A5G2RBP2_PIG Neutral cho
0 0 0 0 0 0 0 0 0 0 0 0 9 1 1 19 2,7 2,7
47,861 0,0030581 1,7979 12698000 1
A0A287A391;A0A288CG57;A0A287BJG5;A0A286ZUI3
A0A287A391;A0A288CG57;A0A287BJG5;A0A286ZUI3 212
tr|A0A287A391|A0A287A391_PIG Elongation factor 1-alpha OS=Sus scrofa OX=9823 GN=EEF1A1 PE=1 SV=2;tr|A0A288CG57|A0A288CG57_PIG Elongation factor 1-alpha OS=Sus scrofa OX=9823 GN=EEF1A1 PE=1 SV=2;tr|A0A287BJG5|A0A287BJG5_PIG Elongation factor 1-alpha OS=Sus

0 0 0 0 0 0 0 0 0 0 0 0 1 1 1 2,2 2,2 2,2
71,43 0,002193 2,2972 5448800 1 F1S458;A0A287A3Q5 F1S458;A0A287A3Q5 213
tr|F1S458|F1S458_PIG Lysine--tRNA ligase OS=Sus scrofa OX=9823 GN=KARS1 PE=1 SV=2;tr|A0A287A3Q5|A0A287A3Q5_PIG Lysine--tRNA ligase OS=Sus scrofa OX=9823 GN=KARS1 PE=1 SV=1

57111000 0 31524000 0 0 68982000 0 0 0 56254000 0 29758000 2
2 2 15,4 15,4 15,4 17,07 0 18,663 465260000 14
A0A287ALJ6;A0A287A3T6;A0A286ZLN6;F1S9H7;A0A5G2R9S9;A0A287BH28
A0A287ALJ6;A0A287A3T6;A0A286ZLN6;F1S9H7;A0A5G2R9S9;A0A287BH28 214
tr|A0A287ALJ6|A0A287ALJ6_PIG Ig-like domain-containing protein OS=Sus scrofa OX=9823 PE=4 SV=1;tr|A0A287A3T6|A0A287A3T6_PIG Ig-like domain-containing protein OS=Sus scrofa OX=9823 PE=4 SV=1;tr|A0A286ZLN6|A0A286ZLN6_PIG Ig-like domain-containing protein OS=

4901400 4081800 0 7245000 0 2754600 9251300 3829700 0 7417900 4105300 2839900
5 5 5 15,4 15,4 15,4 31,649 0 14,712 155310000 25
A0A287A3U6;A0A5G2QCP3;A0A5G2R398;A0A287A424;A0A5G2R6B1
A0A287A3U6;A0A5G2QCP3;A0A5G2R398;A0A287A424;A0A5G2R6B1 215
tr|A0A287A3U6|A0A287A3U6_PIG Nitrilase 1 OS=Sus scrofa OX=9823 GN=NIT1 PE=1 SV=1;tr|A0A5G2QCP3|A0A5G2QCP3_PIG Nitrilase 1 OS=Sus scrofa OX=9823 GN=NIT1 PE=1 SV=1;tr|A0A5G2R398|A0A5G2R398_PIG Nitrilase 1 OS=Sus scrofa OX=9823 GN=NIT1 PE=1 SV=1;tr|A0A287A424

1218400000 867940000 913540000 747020000 820100000 973850000 1047300000
677790000 965030000 1454900000 677240000 895840000 20 20
9 44,1 44,1 22,8 65,603 0 323,31 45023000000 588
A0A287A435;A0A287AM97;A0A286ZWE6;A0A287AYR8;F1SEN8
A0A287A435;A0A287AM97;A0A286ZWE6 216 tr|A0A287A435|A0A287A435_PIG Uncharacterized protein OS=Sus scrofa OX=9823 GN=LDB3 PE=1 SV=2;tr|A0A287AM97|A0A287AM97_PIG Uncharacterized protein OS=Sus scrofa OX=9823 GN=LDB3 PE=1 SV=1;tr|A0A286ZWE6|A0A286ZWE6_PIG Uncharacterized protein OS=Sus scrofa OX=

0 14726000 20658000 0 13071000 11663000 0 8454500 9946800 0 8578700 18119000
2 2 2 6,4 6,4 6,4 41,341 0 5,1752 196620000 11
A0A287A487;Q1G1K7;A0A5G2Q9X2 A0A287A487;Q1G1K7;A0A5G2Q9X2 217
tr|A0A287A487|A0A287A487_PIG Isocitrate dehydrogenase [NAD] subunit, mitochondrial OS=Sus scrofa OX=9823 GN=IDH3B PE=1 SV=1;tr|Q1G1K7|Q1G1K7_PIG Isocitrate dehydrogenase [NAD] subunit, mitochondrial OS=Sus scrofa OX=9823 GN=IDH3B PE=1 SV=1;tr|A0A5G2Q9X2|A0

0 0 0 0 0 0 0 0 0 0 0 0 2 2 2 11,3 11,3 11,3
24,123 0 6,9856 24685000 6 A0A287A4A5;F1SB09;A0A287BEE6
A0A287A4A5;F1SB09;A0A287BEE6 218 tr|A0A287A4A5|A0A287A4A5_PIG SEC22 vesicle-trafficking protein homolog B OS=Sus scrofa OX=9823 GN=SEC22B PE=1 SV=1;tr|F1SB09|F1SB09_PIG SEC22 vesicle-trafficking protein homolog B OS=Sus scrofa OX=9823 GN=SEC22B PE=1 SV=1;tr|A0A287BEE6|A0A287BEE6_PIG SEC22

1137600000 360440000 184260000 643220000 232800000 238650000 785560000
303290000 259940000 589270000 197780000 78789000 10 10 0

54,1 54,1 0 19,202 0 170,44 16421000000 238 A0A287A4C5 A0A287A4C5 219

tr|A0A287A4C5|A0A287A4C5_PIG Troponin C, skeletal muscle OS=Sus scrofa OX=9823 GN=TNNC2 PE=1 SV=1

11015000 0 7111000 9219200 10446000 11597000 15761000 0 7838300 13428000 7050300

9848600 6 6 6 15,8 15,8 15,8 49,39 0 9,5903 187990000 25

I3L8U8;A0A287A4K2;A0A287BRS1 I3L8U8;A0A287A4K2;A0A287BRS1 220

tr|I3L8U8|I3L8U8_PIG Secernin 3 OS=Sus scrofa OX=9823 GN=SCRN3 PE=1 SV=3;tr|A0A287A4K2|A0A287A4K2_PIG Secernin 3 OS=Sus scrofa OX=9823 GN=SCRN3 PE=1 SV=1;tr|A0A287BRS1|A0A287BRS1_PIG Secernin 3 OS=Sus scrofa OX=9823 GN=SCRN3 PE=1 SV=1

19442000 18204000 38064000 29820000 32006000 35780000 19256000 23041000 36215000 36227000

39401000 73610000 11 11 11 44,8 44,8 44,8 32,552 0 79,002

2467300000 122

A0A5G2QVH9;F1SJX1;A0A287A4T2;A0A5G2QRE7;A0A5G2QT70;A0A5G2QQV0;A0A286ZRJ3;A0A5G2QUW1;A0A5G2QKZ3;A0A287AY35

A0A5G2QVH9;F1SJX1;A0A287A4T2;A0A5G2QRE7;A0A5G2QT70;A0A5G2QQV0;A0A286ZRJ3;A0A5G2QUW1;A0A5G2QKZ3;A0A287AY35 221 tr|A0A5G2QVH9|A0A5G2QVH9_PIG Electron transfer flavoprotein subunit alpha OS=Sus scrofa OX=9823 GN=ETFA PE=1 SV=1;tr|F1SJX1|F1SJX1_PIG Electron transfer flavoprotein subunit alpha OS=Sus scrofa OX=9823 GN=ETFA PE=1 SV=2;tr|A0A287A4T2|A0A287A4T2_PIG Electro

7918300 0 0 16209000 0 3804100 13806000 7086200 4836500 12136000 0 3643200

3 3 3 15,3 15,3 15,3 24,132 0 6,6768 150250000 12

A0A287A4W6;A0A5G2QNH8;A0A287BED8 A0A287A4W6;A0A5G2QNH8 222

tr|A0A287A4W6|A0A287A4W6_PIG Ras-related protein Rab-21 OS=Sus scrofa OX=9823 GN=TMEM19 PE=1 SV=1;tr|A0A5G2QNH8|A0A5G2QNH8_PIG Ras-related protein Rab-21 OS=Sus scrofa OX=9823 GN=TMEM19 PE=1 SV=1

238860000 84042000 209630000 307170000 52963000 423060000 334630000 71078000

221630000 362350000 94555000 213970000 6 6 6 34,1 34,1 34,1

24,721 0 233,95 5767800000 114

A0A287A4Y3;F1STC5;A0A287B5G0;F1STC2;A0A286ZQQ8;A0A287ARL5;I3LBK0;A0A287AU93;A0A287BDV3;I3L6U3;A0A286ZK41;A0A5G2QWT2;A0A286ZRV9;A0A287AE24;A0A286ZYQ7;A0A286ZMU4;A0A287AFK6;A0A287BNX9

A0A287A4Y3;F1STC5;A0A287B5G0;F1STC2;A0A286ZQQ8;A0A287ARL5 223

tr|A0A287A4Y3|A0A287A4Y3_PIG Ig-like domain-containing protein OS=Sus scrofa OX=9823 PE=1 SV=1;tr|F1STC5|F1STC5_PIG Ig-like domain-containing protein OS=Sus scrofa OX=9823 PE=1 SV=5;tr|A0A287B5G0|A0A287B5G0_PIG Ig-like domain-containing protein OS=Sus scro

70364000 66476000 95739000 246790000 134130000 82630000 221570000 156050000

196570000 278860000 125560000 163780000 8 8 8 21,4 21,4

21,4 27,118 0 29,488 4147500000 61

A0A287A4Z2;A0A287APZ8;P28768;A0A287A8A8;A0A5G2RDT4

A0A287A4Z2;A0A287APZ8;P28768;A0A287A8A8;A0A5G2RDT4 224

tr|A0A287A4Z2|A0A287A4Z2_PIG Superoxide dismutase OS=Sus scrofa OX=9823 GN=SOD2 PE=1 SV=2;tr|A0A287APZ8|A0A287APZ8_PIG Superoxide dismutase [Mn], mitochondrial OS=Sus

scrofa OX=9823 GN=SOD2 PE=1 SV=2;sp|P28768|SODM_PIG Superoxide dismutase [Mn], mitochondr
11376000 0 0 17256000 6686000 10916000 17081000 0 0 22173000 10202000 9873400
4 4 4 15,1 15,1 15,1 40,779 0 36,873 253930000 17
A0A287A515;F1RVA3;A0A5G2QVC9 A0A287A515;F1RVA3;A0A5G2QVC9 225
tr|A0A287A515|A0A287A515_PIG Gamma-sarcoglycan OS=Sus scrofa OX=9823 GN=SGCG PE=1 SV=2;tr|F1RVA3|F1RVA3_PIG Gamma-sarcoglycan OS=Sus scrofa OX=9823 GN=SGCG PE=1 SV=4;tr|A0A5G2QVC9|A0A5G2QVC9_PIG Gamma-sarcoglycan OS=Sus scrofa OX=9823 GN=SGCG PE=1 SV=1
10604000 15786000 18768000 8552300 25340000 19906000 6714300 13576000 26011000 11594000
20701000 26758000 8 8 8 33,8 33,8 33,8 26,204 0 31,463
622350000 40 A0A287A518;F2Z543;A0A287ADF7;A0A5G2R6X6
A0A287A518;F2Z543;A0A287ADF7 226 tr|A0A287A518|A0A287A518_PIG Prohibitin OS=Sus scrofa OX=9823 GN=PHB PE=1 SV=1;tr|F2Z543|F2Z543_PIG Prohibitin OS=Sus scrofa OX=9823 GN=PHB PE=1 SV=3;tr|A0A287ADF7|A0A287ADF7_PIG Prohibitin OS=Sus scrofa OX=9823 GN=PHB PE=1 SV=1
53748000 72874000 120860000 100850000 150750000 141330000 60393000 93172000
140040000 96652000 163120000 218540000 15 15 15 39 39 39
52,698 0 84,584 8668000000 187
F1SKM0;A0A287A558;A0A287BHP7;A0A287AJH5;F1SB55;A0A480T8S4;A0A5G2QH82
F1SKM0;A0A287A558;A0A287BHP7;A0A287AJH5 227 tr|F1SKM0|F1SKM0_PIG Ubiquinol-cytochrome c reductase core protein 1 OS=Sus scrofa OX=9823 GN=UQCRC1 PE=1 SV=3;tr|A0A287A558|A0A287A558_PIG Ubiquinol-cytochrome c reductase core protein 1 OS=Sus scrofa OX=9823 GN=UQCRC1 PE=1 SV=2;tr|A0A287BHP7|A0A287BHP7_P
7619700 7013500 10154000 0 6490300 0 5873200 7159100 0 6124100 6019100 0
9 9 9 15,1 15,1 15,1 84,293 0 26,881 148600000 16
A0A287A5B4;F1RRD6;A0A287AH24;A0A287ANX4
A0A287A5B4;F1RRD6;A0A287AH24;A0A287ANX4 228 tr|A0A287A5B4|A0A287A5B4_PIG BRO1 domain-containing protein OS=Sus scrofa OX=9823 GN=PDCD6IP PE=1 SV=2;tr|F1RRD6|F1RRD6_PIG BRO1 domain-containing protein OS=Sus scrofa OX=9823 GN=PDCD6IP PE=1 SV=4;tr|A0A287AH24|A0A287AH24_PIG BRO1 domain-containing protei
0 0 0 0 0 0 0 0 0 0 0 0 1 1 1 1,7 1,7 1,7
60,997 0,0059172 1,525 0 1 F1RY43;A0A287A5F9 F1RY43;A0A287A5F9 229
tr|F1RY43|F1RY43_PIG Pyruvate dehyrogenase phosphatase catalytic subunit 1 OS=Sus scrofa OX=9823 GN=PDP1 PE=1 SV=1;tr|A0A287A5F9|A0A287A5F9_PIG Pyruvate dehyrogenase phosphatase catalytic subunit 1 OS=Sus scrofa OX=9823 GN=PDP1 PE=1 SV=1
13128000 27849000 20130000 26646000 0 33632000 14276000 0 19029000 16965000 12063000
13205000 11 3 3 34,5 11,4 11,4 40,103 0 7,0674 441280000 18
A0A5G2QZX1;A0A287AAR4;Q6QAQ1;I3LVD5;A0A287A5G1;A0A287AA77;A0A5S6I3N7;A0A287A4R1 A0A5G2QZX1;A0A287AAR4;Q6QAQ1;I3LVD5;A0A287A5G1;A0A287AA77;A0A5S6I3N7
230 tr|A0A5G2QZX1|A0A5G2QZX1_PIG Uncharacterized protein OS=Sus scrofa OX=9823 GN=ACTG1 PE=1 SV=1;tr|A0A287AAR4|A0A287AAR4_PIG Actin, cytoplasmic 1 OS=Sus scrofa

OX=9823 GN=ACTB PE=3 SV=1;sp|Q6QAQ1|ACTB_PIG Actin, cytoplasmic 1 OS=Sus scrofa OX=9823 GN=ACTB PE
0 0 0 0 0 0 0 0 0 0 0 0 1 1 1 6,2 6,2 6,2
22,037 0,0078201 1,4857 10062000 7 F1S533;A0A287A5G3 F1S533;A0A287A5G3
231 tr|F1S533|F1S533_PIG Transmembrane emp24 domain-containing protein 5 OS=Sus scrofa OX=9823 GN=TMED5 PE=1 SV=2;tr|A0A287A5G3|A0A287A5G3_PIG Transmembrane emp24 domain-containing protein 5 OS=Sus scrofa OX=9823 GN=TMED5 PE=1 SV=1
0 0 0 0 0 0 0 0 0 0 0 0 1 1 1 5,7 5,7 5,7
21,899 0 19,043 288530000 12
F1SUT0;A0A5G2QCT8;A0A5G2QGN9;A0A5G2QQB0;A0A5G2R1S2;A0A5G2QQ57;A0A287BI63;A0A287A5W5;I3LMP5;A0A287AMK7
F1SUT0;A0A5G2QCT8;A0A5G2QGN9;A0A5G2QQB0;A0A5G2R1S2;A0A5G2QQ57;A0A287BI63;A0A287A5W5;I3LMP5;A0A287AMK7 232 tr|F1SUT0|F1SUT0_PIG Uncharacterized protein OS=Sus scrofa OX=9823 GN=RAB6A PE=1 SV=3;tr|A0A5G2QCT8|A0A5G2QCT8_PIG RAB6B, member RAS oncogene family OS=Sus scrofa OX=9823 GN=RAB6B PE=1 SV=1;tr|A0A5G2QGN9|A0A5G2QGN9_PIG RAB6B, member RAS oncogene family OS=
74033000 47725000 42213000 79920000 26601000 38097000 83042000 42602000 34959000 82144000
32893000 28419000 21 21 8 32,2 32,2 6,1 235,79 0 53,528
2059600000 121 A0A287A608;A0A287A4Q2;A0A286ZP76
A0A287A608;A0A287A4Q2;A0A286ZP76 233 tr|A0A287A608|A0A287A608_PIG AHNAK nucleoprotein OS=Sus scrofa OX=9823 GN=AHNAK PE=1 SV=2;tr|A0A287A4Q2|A0A287A4Q2_PIG AHNAK nucleoprotein OS=Sus scrofa OX=9823 GN=AHNAK PE=1 SV=2;tr|A0A286ZP76|A0A286ZP76_PIG AHNAK nucleoprotein OS=Sus scrofa OX=9823 GN=AH
175750000 138560000 270560000 339880000 221510000 268360000 375500000
166600000 172720000 380490000 204510000 330370000 9 9
9 33,2 33,2 33,2 23,359 0 26,058 14838000000 231
A0A287AJ76;A0A287A690;F1SDX9;P52552;A0A5G2QIX9
A0A287AJ76;A0A287A690;F1SDX9;P52552234 tr|A0A287AJ76|A0A287AJ76_PIG
Peroxiredoxin-2 OS=Sus scrofa OX=9823 GN=PRDX2 PE=1 SV=1;tr|A0A287A690|A0A287A690_PIG Peroxiredoxin-2 OS=Sus scrofa OX=9823 GN=PRDX2 PE=1 SV=1;tr|F1SDX9|F1SDX9_PIG Peroxiredoxin-2 OS=Sus scrofa OX=9823 GN=PRDX2 PE=1 SV=4;sp|P52
9390000 6130500 7599800 0 0 0 0 0 0 0 0 0 3 3 3
8,4 8,4 8,4 48,501 0 5,9714 62928000 7
A0A287B646;A0A287A699;I3LQS0;A0A287BKQ9;A0A5G2RGD5
A0A287B646;A0A287A699;I3LQS0;A0A287BKQ9 235 tr|A0A287B646|A0A287B646_PIG Heterogeneous nuclear ribonucleoprotein K OS=Sus scrofa OX=9823 GN=HNRNPK PE=1 SV=2;tr|A0A287A699|A0A287A699_PIG Heterogeneous nuclear ribonucleoprotein K OS=Sus scrofa OX=9823 GN=HNRNPK PE=1 SV=2;tr|I3LQS0|I3LQS0_PIG Heterogen
6840100 8693700 5582300 0 5210800 0 0 0 4174500 0 0 0 3
3 3 8,1 8,1 8,1 50,58 0 4,1682 94105000 11
A0A287AT16;A0A287BSE8;A0A287AAK1;A0A287A6E6
A0A287AT16;A0A287BSE8;A0A287AAK1;A0A287A6E6 236
tr|A0A287AT16|A0A287AT16_PIG G protein pathway suppressor 1 OS=Sus scrofa OX=9823

GN=GPS1 PE=3 SV=2;tr|A0A287BSE8|A0A287BSE8_PIG G protein pathway suppressor 1 OS=Sus scrofa OX=9823 GN=GPS1 PE=3 SV=2;tr|A0A287AAK1|A0A287AAK1_PIG G protein pathway suppresso
2365500000 588640000 521790000 1942300000 459330000 952560000 982440000
700530000 443300000 742490000 418440000 130100000 15 15
15 79,6 79,6 79,6 18,852 0 125 59103000000 712
A0A287A6F0;Q5XLD2;A0A5G2R327;A0A5G2Q8Q3;A0A5G2RLS9;A0A5G2QIC4
A0A287A6F0;Q5XLD2;A0A5G2R327;A0A5G2Q8Q3;A0A5G2RLS9 237
tr|A0A287A6F0|A0A287A6F0_PIG Myosin light chain, phosphorylatable, fast skeletal muscle OS=Sus scrofa OX=9823 GN=MYLPF PE=1 SV=2;tr|Q5XLD2|Q5XLD2_PIG Myosin light chain, phosphorylatable, fast skeletal muscle OS=Sus scrofa OX=9823 GN=MYLPF PE=1 SV=1;tr|A0A
0 0 0 0 0 0 0 0 0 0 0 0 2 2 2 7,2 7,2 7,2
40,464 0,0087379 1,4351 6122400 2 A0A287A6F5 A0A287A6F5 238
tr|A0A287A6F5|A0A287A6F5_PIG Prostaglandin reductase 3 isoform 1 OS=Sus scrofa OX=9823 GN=ZADH2 PE=1 SV=1
0 0 0 0 0 0 0 0 0 0 0 0 1 1 1 4,5 4,5 4,5
33,119 0 3,0691 4537600 1
A0A287A6I6;A0A5G2R466;A0A287AYH4;A0A287BKH7;A0A5G2Q8L9;A0A5G2QBZ4;I3LFE8
A0A287A6I6;A0A5G2R466;A0A287AYH4;A0A287BKH7;A0A5G2Q8L9;A0A5G2QBZ4;I3LFE8
239 tr|A0A287A6I6|A0A287A6I6_PIG Inorganic diphosphatase OS=Sus scrofa OX=9823 GN=PPA2 PE=1 SV=2;tr|A0A5G2R466|A0A5G2R466_PIG Inorganic diphosphatase OS=Sus scrofa OX=9823 GN=PPA2 PE=1 SV=1;tr|A0A287AYH4|A0A287AYH4_PIG Inorganic diphosphatase OS=Sus scrofa OX=
0 0 6639500 7960300 6225700 7321400 7942300 0 7648700 0 5834000 7491100
4 4 4 7,4 7,4 7,4 69,319 0 5,8179 139390000 11
A0A287A6K7;A0A287BI42;A0A287AFN5 A0A287A6K7;A0A287BI42;A0A287AFN5 240
tr|A0A287A6K7|A0A287A6K7_PIG Adipocyte plasma membrane-associated protein OS=Sus scrofa OX=9823 GN=APMAP PE=1 SV=1;tr|A0A287BI42|A0A287BI42_PIG Adipocyte plasma membrane-associated protein OS=Sus scrofa OX=9823 GN=APMAP PE=1 SV=1;tr|A0A287AFN5|A0A287AFN5_P
73086000 65385000 30048000 82679000 67024000 56544000 91565000 55197000 34301000 108560000
62593000 31849000 7 7 7 29,4 29,4 29,4 28,153 0 17,257
2478100000 57 A0A287A6V9 A0A287A6V9 241 tr|A0A287A6V9|A0A287A6V9_PIG
Carboxymethylenebutenolidase homolog OS=Sus scrofa OX=9823 GN=CMBL PE=1 SV=1
0 0 0 0 0 0 0 0 0 0 0 0 3 3 3 8,2 8,2 8,2
48,244 0 3,5552 44415000 4
F1SR76;A0A287B1J1;A0A287A7C2;A0A5G2R9H6;A0A287ALM0
F1SR76;A0A287B1J1;A0A287A7C2;A0A5G2R9H6;A0A287ALM0 242 tr|F1SR76|F1SR76_PIG Aspartyl aminopeptidase OS=Sus scrofa OX=9823 GN=DNPEP PE=1 SV=4;tr|A0A287B1J1|A0A287B1J1_PIG Aspartyl aminopeptidase OS=Sus scrofa OX=9823 GN=DNPEP PE=1 SV=1;tr|A0A287A7C2|A0A287A7C2_PIG Aspartyl aminopeptidase OS=Sus scrofa OX=9823 G

0 8186600 19917000 3731000 22217000 28775000 0 11312000 19885000 5086700 18134000 31632000 7 7 7 25,8 25,8 25,8 29,882 0 12,929 524690000 33
A0A287A7F6;A0A287B037;A0A5G2QT83;I3LQN4;A0A5G2QCF6;A0A5G2QBR0
A0A287A7F6;A0A287B037;A0A5G2QT83;I3LQN4;A0A5G2QCF6 243
tr|A0A287A7F6|A0A287A7F6_PIG Prohibitin OS=Sus scrofa OX=9823 GN=PHB2 PE=1 SV=2;tr|A0A287B037|A0A287B037_PIG Prohibitin OS=Sus scrofa OX=9823 GN=PHB2 PE=1 SV=1;tr|A0A5G2QT83|A0A5G2QT83_PIG Prohibitin OS=Sus scrofa OX=9823 GN=PHB2 PE=1 SV=1;tr|I3LQN4|I3LQN4
0 6345500 0 0 0 6329900 0 0 5817100 0 0 7402700 5 5 2 15 15 5,9 37,377 0 5,5947 104910000 8
A0A287A7Q3;I3LSK5;A0A287AW90;A0A287A365;A0A287AHH8;F1SLU8
A0A287A7Q3;I3LSK5;A0A287AW90;A0A287A365 244 tr|A0A287A7Q3|A0A287A7Q3_PIG G protein subunit beta 1 OS=Sus scrofa OX=9823 GN=GNB1 PE=3 SV=1;tr|I3LSK5|I3LSK5_PIG G protein subunit beta 1 OS=Sus scrofa OX=9823 GN=GNB1 PE=3 SV=3;tr|A0A287AW90|A0A287AW90_PIG G protein subunit beta 1 OS=Sus scrofa OX=9823
4139600000 5124100000 2096900000 6052700000 3383400000 2541700000 6588600000 4356000000 3275200000 4454500000 3465500000 1624200000 44 44 44 33,6 33,6 33,6 126,28 0 323,31 441210000000 3779
A0A287AQJ5;A0A287A7R4;F1SHM0;A0A287B8G0;A0A287B7L9;F1SHL9;A0A287B785;A0A286ZI36;A0A286ZYV7
A0A287AQJ5;A0A287A7R4;F1SHM0;A0A287B8G0;A0A287B7L9;F1SHL9;A0A287B785;A0A286ZI36 245 tr|A0A287AQJ5|A0A287AQJ5_PIG Multifunctional fusion protein OS=Sus scrofa OX=9823 GN=PKM PE=1 SV=1;tr|A0A287A7R4|A0A287A7R4_PIG Multifunctional fusion protein OS=Sus scrofa OX=9823 GN=PKM PE=1 SV=1;tr|F1SHM0|F1SHM0_PIG Pyruvate kinase OS=Sus scrofa OX=9823
83941000 61942000 134060000 201560000 166670000 151960000 179630000 100000000 125040000 249350000 177220000 275300000 4 4 4 43 43 43 10,156 0 18,704 4459100000 133
A0A287A808;F1RLH7;A0A287AND7;A0A5G2QL31 A0A287A808;F1RLH7 246
tr|A0A287A808|A0A287A808_PIG Cytochrome c oxidase subunit OS=Sus scrofa OX=9823 GN=COX6B PE=1 SV=1;tr|F1RLH7|F1RLH7_PIG Uncharacterized protein OS=Sus scrofa OX=9823 GN=COX6B PE=1 SV=3
1547200 4115700 4462000 0 20384000 1160500 483780 32582000 670620 244490 32942000 4024600 55 55 55 13,3 13,3 13,3 513,05 0 143,19 1434700000 126
A0A287B217;A0A287BN04;A0A287BNK7;A0A287B0Y8;A0A287A8C7;A0A287B9H1;A0A287AA88
A0A287B217;A0A287BN04;A0A287BNK7;A0A287B0Y8;A0A287A8C7;A0A287B9H1;A0A287AA88 248 tr|A0A287B217|A0A287B217_PIG Plectin OS=Sus scrofa OX=9823 GN=PLEC PE=1 SV=2;tr|A0A287BN04|A0A287BN04_PIG Plectin OS=Sus scrofa OX=9823 GN=PLEC PE=1 SV=1;tr|A0A287BNK7|A0A287BNK7_PIG Plectin OS=Sus scrofa OX=9823 GN=PLEC PE=1 SV=2;tr|A0A287B0Y8|A0A287B0Y8_
0 0 0 6764300 0 0 0 0 0 4426400 0 0 2 2 2 6,9 6,9 6,9 29,445 0,002181 2,2655 21465000 4

F1RQ24;Q31072;A0A287ARX3;A0A287A8D7;A0A287BF11;A0A287AAB9

F1RQ24;Q31072;A0A287ARX3;A0A287A8D7;A0A287BF11;A0A287AAB9 249

tr|F1RQ24|F1RQ24_PIG Ig-like domain-containing protein OS=Sus scrofa OX=9823 GN=SLA-DRB1 PE=1 SV=4;tr|Q31072|Q31072_PIG HLA class II histocompatibility antigen, DRB1-4 beta chain isoform X2 OS=Sus scrofa OX=9823 GN=LA-DRB-d PE=1 SV=1;tr|A0A287ARX3|A0A287AR

13335000 7302800 6062400 21340000 0 7652200 17038000 0 6832100 14450000 0 4632300

5 5 5 27,7 27,7 27,7 20,698 0 21,727 213070000 24

A0A287A8F2;A0A5G2RL32;F1SUC9 A0A287A8F2;A0A5G2RL32;F1SUC9 250

tr|A0A287A8F2|A0A287A8F2_PIG Leucine rich repeat containing 20 OS=Sus scrofa OX=9823 GN=LRRC20 PE=1 SV=1;tr|A0A5G2RL32|A0A5G2RL32_PIG Leucine rich repeat containing 20 OS=Sus scrofa OX=9823 GN=LRRC20 PE=1 SV=1;tr|F1SUC9|F1SUC9_PIG Leucine rich repeat conta

0 7513000 7896000 0 7010600 7072600 5549400 0 5337800 6163400 7218600 9242300

6 4 4 14,3 10,1 10,1 43,394 0 8,6351 135060000 27

A0A287B465;A0A287A8M1;I3LDC7 A0A287B465;A0A287A8M1;I3LDC7 251

tr|A0A287B465|A0A287B465_PIG Isocitrate dehydrogenase [NADP] OS=Sus scrofa OX=9823 GN=IDH1 PE=1 SV=1;tr|A0A287A8M1|A0A287A8M1_PIG Isocitrate dehydrogenase [NADP] OS=Sus scrofa OX=9823 GN=IDH1 PE=1 SV=1;tr|I3LDC7|I3LDC7_PIG Isocitrate dehydrogenase [NADP] O

29185000 0 28202000 10754000 19628000 35405000 13811000 0 24302000 0 14896000 18558000

3 3 3 11,6 11,6 11,6 29,251 0 6,39 278540000 9 A0A287A8T0

A0A287A8T0 252 tr|A0A287A8T0|A0A287A8T0_PIG Ribosomal protein L7 OS=Sus scrofa OX=9823 GN=RPL7 PE=1 SV=2

8521900 6101400 24732000 11125000 14159000 18010000 6331000 6627900 12845000 11989000

15381000 31585000 7 7 7 19,8 19,8 19,8 42,811 0 28,498

559100000 47 A0A287BG49;A0A287A8Y8;A0A287AP64;P79273;F1RJH2

A0A287BG49;A0A287A8Y8;A0A287AP64;P79273;F1RJH2 253

tr|A0A287BG49|A0A287BG49_PIG Short-chain-specific acyl-CoA dehydrogenase, mitochondrial OS=Sus scrofa OX=9823 GN=ACADS PE=1 SV=1;tr|A0A287A8Y8|A0A287A8Y8_PIG Short-chain-specific acyl-CoA dehydrogenase, mitochondrial OS=Sus scrofa OX=9823 GN=ACADS PE=1 SV=

0 0 0 0 0 0 0 0 0 0 0 0 1 1 1 2,9 2,9 2,9

38,603 0 3,4765 36366000 2 F2Z540;A0A287A976 F2Z540;A0A287A976 254

tr|F2Z540|F2Z540_PIG COP9 signalosome subunit 5 OS=Sus scrofa OX=9823 GN=COPS5 PE=1 SV=3;tr|A0A287A976|A0A287A976_PIG COP9 signalosome subunit 5 OS=Sus scrofa OX=9823 GN=COPS5 PE=1 SV=2

8542700 0 0 0 4193400 0 7158700 0 0 5747300 0 4737500 6

6 6 10,7 10,7 10,7 70,754 0 17,398 95773000 12 F1S6Z6;A0A287A996

F1S6Z6;A0A287A996 255 tr|F1S6Z6|F1S6Z6_PIG Taxilin beta OS=Sus scrofa OX=9823 GN=TXLNB PE=1 SV=3;tr|A0A287A996|A0A287A996_PIG Taxilin beta OS=Sus scrofa OX=9823 GN=TXLNB PE=1 SV=1

6682900 4794100 6448900 0 5116800 0 0 0 0 0 0 0 3 3

3 6,8 6,8 6,8 58,808 0 16,93 46605000 11

A0A287BIR4;A0A287B6M8;A0A287A9E8;A0A287BEJ8;A0A287ADQ2;A0A286ZXY2;A0A481B

HF4;F1SDL5;A0A286ZW01;A0A480RJP3;A0A287BMV0

A0A287BIR4;A0A287B6M8;A0A287A9E8;A0A287BEJ8;A0A287ADQ2;A0A286ZXY2;A0A481BHF4;F1SDL5;A0A286ZW01;A0A480RJP3;A0A287BMV0 256 tr|A0A287BIR4|A0A287BIR4_PIG Dystrobrevin alpha OS=Sus scrofa OX=9823 GN=DTNA PE=1 SV=1;tr|A0A287B6M8|A0A287B6M8_PIG Dystrobrevin alpha OS=Sus scrofa OX=9823 GN=DTNA PE=1 SV=1;tr|A0A287A9E8|A0A287A9E8_PIG Dystrobrevin OS=Sus scrofa OX=9823 GN=DTNA PE=1 SV=

0 0 0 0 0 7064300 0 0 6434900 0 0 13703000 2 2 2

3,4 3,4 3,4 45,035 0,00499 1,6269 79501000 3

I3LLW3;A0A5G2QSG1;A0A5G2R4T0;A0A287A9I6

I3LLW3;A0A5G2QSG1;A0A5G2R4T0;A0A287A9I6 257 tr|I3LLW3|I3LLW3_PIG SAMM50 sorting and assembly machinery component OS=Sus scrofa OX=9823 GN=SAMM50 PE=1 SV=2;tr|A0A5G2QSG1|A0A5G2QSG1_PIG SAMM50 sorting and assembly machinery component OS=Sus scrofa OX=9823 GN=SAMM50 PE=1 SV=1;tr|A0A5G2R4T0|A0A5G2R4T0_P

29862000 39743000 131670000 0 111850000 115500000 0 42784000 59008000 12119000

58145000 212330000 7 7 7 26,6 26,6 26,6 30,337 0 15,338

1421600000 29 A0A287A9I8;A0A287AHM1 A0A287A9I8;A0A287AHM1 258

tr|A0A287A9I8|A0A287A9I8_PIG ATP synthase subunit gamma OS=Sus scrofa OX=9823 GN=ATP5F1C PE=1 SV=2;tr|A0A287AHM1|A0A287AHM1_PIG ATP synthase subunit gamma OS=Sus scrofa OX=9823 GN=ATP5F1C PE=3 SV=1

13757000 0 10984000 0 0 0 0 0 0 16853000 0 0 2 2 2

15 15 15 15,635 0 22,52 110530000 6

F1RIU9;A0A287BL36;A0A287AQI8;A0A287A9W3

F1RIU9;A0A287BL36;A0A287AQI8;A0A287A9W3 259 tr|F1RIU9|F1RIU9_PIG Coiled-coil-helix-coiled-coil-helix domain-containing protein 2 OS=Sus scrofa OX=9823 GN=CHCHD2 PE=1 SV=2;tr|A0A287BL36|A0A287BL36_PIG Uncharacterized protein OS=Sus scrofa OX=9823 PE=4 SV=1;tr|A0A287AQI8|A0A287AQI8_PIG Uncharacterized

0 0 9581700 0 0 0 0 0 0 0 0 6103900 2 2 2 5,7

5,7 5,7 36,694 0 7,5988 52205000 5

A0A287AQS0;F1S297;A0A287A9X1;A0A5G2QJF8;P41566

A0A287AQS0;F1S297;A0A287A9X1;A0A5G2QJF8;P41566 260

tr|A0A287AQS0|A0A287AQS0_PIG Isocitrate dehydrogenase [NAD] subunit, mitochondrial OS=Sus scrofa OX=9823 GN=IDH3G PE=1 SV=1;tr|F1S297|F1S297_PIG Isocitrate dehydrogenase [NAD] subunit, mitochondrial OS=Sus scrofa OX=9823 GN=IDH3G PE=1 SV=3;tr|A0A287A9X1|A0

0 0 0 0 0 0 0 0 0 0 0 0 1 1 1 3,4 3,4 3,4

39,025 0 12,559 52136000 7 A0A5K1UBB6;A0A5G2R0Q8;A0A287AA20

A0A5K1UBB6;A0A5G2R0Q8;A0A287AA20 261 tr|A0A5K1UBB6|A0A5K1UBB6_PIG Sarcoglycan alpha OS=Sus scrofa OX=9823 GN=SGCA PE=1 SV=1;tr|A0A5G2R0Q8|A0A5G2R0Q8_PIG Sarcoglycan alpha OS=Sus scrofa OX=9823 GN=SGCA PE=1 SV=1;tr|A0A287AA20|A0A287AA20_PIG Sarcoglycan alpha OS=Sus scrofa OX=9823 GN=SGCA PE=1

12662000 13030000 21816000 18753000 28018000 21269000 18010000 15015000 18951000 20266000

21022000 25253000 6 6 6 20,1 20,1 20,1 34,58 0 18,245

729830000 33 A0A287AA21;A0A287BF56 A0A287AA21;A0A287BF56 262

tr|A0A287AA21|A0A287AA21_PIG Enoyl-CoA hydratase, mitochondrial OS=Sus scrofa OX=9823 GN=ECHS1 PE=1 SV=2;tr|A0A287BF56|A0A287BF56_PIG Uncharacterized protein OS=Sus scrofa OX=9823 GN=ECHS1 PE=1 SV=1

13521000 11242000 17531000 16223000 13850000 15171000 15283000 12358000 11518000 17584000

12301000 13513000 4 4 4 28,5 28,5 28,5 15,907 0 15,696

277110000 16 A0A5G2QR20;A0A287AAE2;Q8MJ30;A0A5G2QZN1

A0A5G2QR20;A0A287AAE2;Q8MJ30;A0A5G2QZN1 263

tr|A0A5G2QR20|A0A5G2QR20_PIG Dihydropteridine reductase OS=Sus scrofa OX=9823 GN=QDPR PE=4 SV=1;tr|A0A287AAE2|A0A287AAE2_PIG Dihydropteridine reductase OS=Sus scrofa OX=9823 GN=QDPR PE=4 SV=1;sp|Q8MJ30|DHPR_PIG Dihydropteridine reductase OS=Sus scrofa OX=9

0 0 0 0 0 0 0 0 0 0 0 0 1 1 1 15,7 15,7 15,7

7,9431 0,0030303 1,7509 0 2 A0A287AAH8;A0A287AJT7

A0A287AAH8;A0A287AJT7 264 tr|A0A287AAH8|A0A287AAH8_PIG 60S ribosomal protein L28 OS=Sus scrofa OX=9823 GN=RPL28 PE=3 SV=1;tr|A0A287AJT7|A0A287AJT7_PIG 60S ribosomal protein L28 OS=Sus scrofa OX=9823 GN=RPL28 PE=1 SV=1

2047800000 470220000 320200000 1325000000 369690000 355810000 1356400000

432920000 411100000 912580000 320440000 147030000 14 14

2 27,1 27,1 3,4 31,547 0 106,4 23656000000 329

Q75NH2;A0A287ARW1;A0A287AFK2;A0A287AAI0;A0A5S6I3K1;A0A5G2R3I0;A0A287BHG2;A0A287A421

Q75NH2;A0A287ARW1;A0A287AFK2;A0A287AAI0;A0A5S6I3K1;A0A5G2R3I0;A0A287BHG2;A0A287A421 265 tr|Q75NH2|Q75NH2_PIG Troponin T, fast skeletal muscle OS=Sus scrofa OX=9823 GN=TNNT3 PE=2 SV=1;tr|A0A287ARW1|A0A287ARW1_PIG Troponin T, fast skeletal muscle OS=Sus scrofa OX=9823 GN=TNNT3 PE=3 SV=2;tr|A0A287AFK2|A0A287AFK2_PIG Troponin T, fast skeletal mus

23097000 11748000 14783000 25951000 15520000 17476000 21650000 10116000 11678000 27818000

13965000 18954000 6 6 6 27,7 27,7 27,7 28,937 0 24,709

535460000 51

A0A287AAI9;A0A287AZ17;F1RG31;A0A5G2QKD2;A0A5G2QP05;A0A287BIP5

A0A287AAI9;A0A287AZ17;F1RG31;A0A5G2QKD2;A0A5G2QP05 266

tr|A0A287AAI9|A0A287AAI9_PIG Hydroxyacylglutathione hydrolase OS=Sus scrofa OX=9823 GN=HAGH PE=1 SV=1;tr|A0A287AZ17|A0A287AZ17_PIG Hydroxyacylglutathione hydrolase OS=Sus scrofa OX=9823 GN=HAGH PE=1 SV=2;tr|F1RG31|F1RG31_PIG Hydroxyacylglutathione hydrolas

324280000 226560000 1146300000 98466000 501690000 1352300000 58955000

249130000 483270000 390330000 603090000 1620600000 18 18

6 52 52 15,5 38,749 0 160,98 40188000000 528

K7GSI9;K7GLQ8;A0A5K1UHX7;A0A287AAL6;K7GS06;A0A5K1U1D1

K7GSI9;K7GLQ8;A0A5K1UHX7;A0A287AAL6;K7GS06;A0A5K1U1D1 267

tr|K7GSI9|K7GSI9_PIG Four and a half LIM domains 1 OS=Sus scrofa OX=9823 GN=FHL1 PE=1

SV=2;tr|K7GLQ8|K7GLQ8_PIG Four and a half LIM domains 1 OS=Sus scrofa OX=9823 GN=FHL1 PE=1 SV=3;tr|A0A5K1UHX7|A0A5K1UHX7_PIG Four and a half LIM domains 1 OS=Sus scrofa O
0 0 0 0 0 0 0 0 0 0 0 0 2 2 2 21,9 21,9 21,9
10,963 0 3,8161 19196000 7
A0A287AAM0;A0A5G2QHV9;A0A287ABY4;A0A5G2Q9K4;A0A5G2QYQ6;A0A5G2QKQ3
A0A287AAM0;A0A5G2QHV9;A0A287ABY4;A0A5G2Q9K4;A0A5G2QYQ6;A0A5G2QKQ3
268 tr|A0A287AAM0|A0A287AAM0_PIG Dynein light chain roadblock OS=Sus scrofa OX=9823 GN=DYNLRB1 PE=1 SV=1;tr|A0A5G2QHV9|A0A5G2QHV9_PIG Dynein light chain roadblock-type 2 OS=Sus scrofa OX=9823 GN=DYNLRB2 PE=4 SV=1;tr|A0A287ABY4|A0A287ABY4_PIG Dynein light chain
0 4744600 8358500 4852400 5732300 4111800 0 8094700 10504000 3857700 5550200
6662200 4 4 4 18,5 18,5 18,5 34,339 0 7,2577 130070000 15
A0A287AAP0;A0A5G2R332 A0A287AAP0;A0A5G2R332 269
tr|A0A287AAP0|A0A287AAP0_PIG Fumarylacetoacetate hydrolase domain-containing protein 2 OS=Sus scrofa OX=9823 GN=LOC100522130 PE=1 SV=1;tr|A0A5G2R332|A0A5G2R332_PIG FAA_hydrolase domain-containing protein OS=Sus scrofa OX=9823 GN=LOC100522130 PE=1 SV=1
0 0 0 0 0 0 0 0 0 0 0 0 2 2 2 14,7 14,7 14,7
17,289 0,0022247 2,4036 33721000 4 A0A287AAR5;F1SAB6 A0A287AAR5;F1SAB6
270 tr|A0A287AAR5|A0A287AAR5_PIG Acyl carrier protein OS=Sus scrofa OX=9823 GN=NDUFAB1 PE=1 SV=1;tr|F1SAB6|F1SAB6_PIG Acyl carrier protein OS=Sus scrofa OX=9823 GN=NDUFAB1 PE=1 SV=3
0 0 0 0 0 0 0 0 0 0 0 0 1 1 1 10,1 10,1 10,1
12,421 0,0011299 2,5548 63823000 8
A0A287AAT2;A0A5G2R3A4;A0A287ARZ0;Q06AA8;A0A5G2RMT5;F2Z5P3
A0A287AAT2;A0A5G2R3A4;A0A287ARZ0;Q06AA8;A0A5G2RMT5;F2Z5P3 271
tr|A0A287AAT2|A0A287AAT2_PIG Ubiquitin conjugating enzyme E2 D1 OS=Sus scrofa OX=9823 GN=UBE2D1 PE=3 SV=1;tr|A0A5G2R3A4|A0A5G2R3A4_PIG Ubiquitin conjugating enzyme E2 D1 OS=Sus scrofa OX=9823 GN=UBE2D1 PE=3 SV=1;tr|A0A287ARZ0|A0A287ARZ0_PIG Ubiquitin conju
118690000 17212000 114960000 28585000 59139000 68196000 30868000 20268000 28515000
39777000 32247000 72458000 47 46 46 47,7 46,7 46,7 133,77 0 251,45
9129800000 422
A0A287AAY6;F1SRI8;A0A287BGX8;A0A287B5J2;A0A287B943;A0A287BQI3;A0A286ZZN7;A0A5G2QM90;A0A287ATS2
A0A287AAY6;F1SRI8;A0A287BGX8;A0A287B5J2;A0A287B943;A0A287BQI3;A0A286ZZN7;A0A5G2QM90;A0A287ATS2 272 tr|A0A287AAY6|A0A287AAY6_PIG Myosin binding protein C1 OS=Sus scrofa OX=9823 GN=MYBPC1 PE=1 SV=2;tr|F1SRI8|F1SRI8_PIG Myosin binding protein C1 OS=Sus scrofa OX=9823 GN=MYBPC1 PE=1 SV=3;tr|A0A287BGX8|A0A287BGX8_PIG Myosin binding protein C1 OS=Sus scrofa O
5802900 0 4100900 4072100 0 6054200 0 0 0 0 0 0 3 3
3 5 5 5 80,395 0 6,0842 69949000 10
A0A5G2RCK2;F1RQW6;A0A287AAZ9;K7GPT9

A0A5G2RCK2;F1RQW6;A0A287AAZ9;K7GPT9 273 tr|A0A5G2RCK2|A0A5G2RCK2_PIG C3/C5 convertase OS=Sus scrofa OX=9823 GN=CFB PE=1 SV=1;tr|F1RQW6|F1RQW6_PIG C3/C5 convertase OS=Sus scrofa OX=9823 GN=CFB PE=1 SV=2;tr|A0A287AAZ9|A0A287AAZ9_PIG C3/C5 convertase OS=Sus scrofa OX=9823 GN=CFB PE=1 SV=2;tr|K7GPT9
0 0 0 0 0 0 0 0 0 0 0 0 1 1 1 3,9 3,9 3,9
33,534 0,0021575 2,1461 17278000 1 F1RP25;A0A5G2R6V1;A0A287ABH0
F1RP25;A0A5G2R6V1;A0A287ABH0 274 tr|F1RP25|F1RP25_PIG Citramalyl-CoA lyase OS=Sus scrofa OX=9823 GN=CLYBL PE=1 SV=4;tr|A0A5G2R6V1|A0A5G2R6V1_PIG Citramalyl-CoA lyase OS=Sus scrofa OX=9823 GN=CLYBL PE=1 SV=1;tr|A0A287ABH0|A0A287ABH0_PIG Citramalyl-CoA lyase OS=Sus scrofa OX=9823 GN=CLYBL P
14866000 0 20804000 34448000 15179000 18469000 26707000 18885000 19661000 31742000 24552000
33213000 4 4 4 43,9 43,9 43,9 11,985 0 11,699 656240000 48
A0A5G2QG10;A0A287ABR3;A0A5G2QPG5;F1RP45;A0A287BBW3
A0A5G2QG10;A0A287ABR3;A0A5G2QPG5 275 tr|A0A5G2QG10|A0A5G2QG10_PIG ZnF_CDGSH domain-containing protein OS=Sus scrofa OX=9823 GN=CISD1 PE=4 SV=1;tr|A0A287ABR3|A0A287ABR3_PIG ZnF_CDGSH domain-containing protein OS=Sus scrofa OX=9823 GN=CISD1 PE=4 SV=1;tr|A0A5G2QPG5|A0A5G2QPG5_PIG ZnF_CDGSH domai
25438000 24216000 18735000 48277000 21797000 28822000 59354000 30290000 23123000 39643000
21188000 19578000 7 6 6 34,5 29,7 29,7 27,718 0 28,831
1550000000 54 A0A287AC42;A0A287BBB5;A0A5G2RB73
A0A287AC42;A0A287BBB5;A0A5G2RB73 276 tr|A0A287AC42|A0A287AC42_PIG VAMP associated protein A OS=Sus scrofa OX=9823 GN=VAPA PE=1 SV=1;tr|A0A287BBB5|A0A287BBB5_PIG VAMP associated protein A OS=Sus scrofa OX=9823 GN=VAPA PE=1 SV=1;tr|A0A5G2RB73|A0A5G2RB73_PIG VAMP associated protein A OS=Sus scro
0 0 0 0 0 0 0 0 0 0 0 0 1 1 1 5,1 5,1 5,1
19,603 0,00501 1,6561 29070000 3 A0A287ACA3;A0A5G2QJU8;I3LV38
A0A287ACA3;A0A5G2QJU8;I3LV38 277 tr|A0A287ACA3|A0A287ACA3_PIG Peptide-methionine (R)-S-oxide reductase OS=Sus scrofa OX=9823 GN=MSRB3 PE=1 SV=1;tr|A0A5G2QJU8|A0A5G2QJU8_PIG Peptide-methionine (R)-S-oxide reductase OS=Sus scrofa OX=9823 GN=MSRB3 PE=1 SV=1;tr|I3LV38|I3LV38_PIG Peptide-methi
163590000 0 119780000 165990000 161940000 179580000 209340000
136560000 160950000 191020000 155990000 140150000 9 8
8 12,3 11,3 11,3 84,683 0 34,61 2667000000 59
A0A287ACF3;F1SU55;A0A5G2R2B8 A0A287ACF3;F1SU55;A0A5G2R2B8 278
tr|A0A287ACF3|A0A287ACF3_PIG Synaptopodin 2 like OS=Sus scrofa OX=9823 GN=SYNPO2L PE=4 SV=1;tr|F1SU55|F1SU55_PIG Synaptopodin 2 like OS=Sus scrofa OX=9823 GN=SYNPO2L PE=4 SV=3;tr|A0A5G2R2B8|A0A5G2R2B8_PIG Synaptopodin 2 like OS=Sus scrofa OX=9823 GN=SYNPO2
0 0 0 0 0 0 0 0 0 0 0 0 1 1 1 18,7 18,7 18,7
7,7305 0,0030832 1,8523 5345500 1 A0A287ACN2 A0A287ACN2 279
tr|A0A287ACN2|A0A287ACN2_PIG Adipogenesis regulatory factor OS=Sus scrofa OX=9823 GN=ADIRF PE=1 SV=2

0 0 0 0 0 0 0 0 0 0 0 0 1 1 1 7,8 7,8 7,8
14,886 0,0087294 1,4336 3943900 2 F2Z5Q7;A0A287ACR8 F2Z5Q7;A0A287ACR8
280 tr|F2Z5Q7|F2Z5Q7_PIG N-acetyltransferase domain-containing protein OS=Sus scrofa OX=9823 GN=NAA50 PE=1 SV=3;tr|A0A287ACR8|A0A287ACR8_PIG N-acetyltransferase domain-containing protein OS=Sus scrofa OX=9823 GN=NAA50 PE=1 SV=1
8849300 0 0 10458000 6544200 5607800 0 0 5093900 9683400 5402800 4638800
2 2 2 9,4 9,4 9,4 24,612 0 7,7029 133210000 20
A0A287ACY5;A0A5G2QVK4 A0A287ACY5;A0A5G2QVK4 281
tr|A0A287ACY5|A0A287ACY5_PIG Synaptogyrin OS=Sus scrofa OX=9823 GN=SYNGR2 PE=3 SV=1;tr|A0A5G2QVK4|A0A5G2QVK4_PIG Synaptogyrin OS=Sus scrofa OX=9823 GN=SYNGR2 PE=3 SV=1
0 0 0 0 0 0 0 0 0 0 0 0 1 1 1 2,2 2,2 2,2
50,568 0,0031746 2,0334 13563000 2 A0A287AD42;I3LSP1 A0A287AD42;I3LSP1
282 tr|A0A287AD42|A0A287AD42_PIG Methionine aminopeptidase 2 OS=Sus scrofa OX=9823 GN=METAP2 PE=1 SV=1;tr|I3LSP1|I3LSP1_PIG Methionine aminopeptidase 2 OS=Sus scrofa OX=9823 GN=METAP2 PE=1 SV=2
0 0 0 0 0 0 0 0 0 0 0 0 2 2 2 3 3 3
72,496 0 2,9368 60209000 2
A0A5G2QEK1;A0A5G2QE98;A0A287BDZ6;A0A5G2QJI7;F1SFE3;A0A287AD53;A0A5G2QJL5
A0A5G2QEK1;A0A5G2QE98;A0A287BDZ6;A0A5G2QJI7;F1SFE3;A0A287AD53;A0A5G2QJL5
283 tr|A0A5G2QEK1|A0A5G2QEK1_PIG Fermitin family member 2 OS=Sus scrofa OX=9823 GN=FERMT2 PE=1 SV=1;tr|A0A5G2QE98|A0A5G2QE98_PIG Fermitin family member 2 OS=Sus scrofa OX=9823 GN=FERMT2 PE=1 SV=1;tr|A0A287BDZ6|A0A287BDZ6_PIG Fermitin family member 2 OS=Sus scr
0 0 0 0 0 0 0 0 0 0 0 0 1 1 1 4,1 4,1 4,1
28,027 0,0031315 1,9259 2050900 3 A0A287AD61 A0A287AD61 284
tr|A0A287AD61|A0A287AD61_PIG B-cell receptor-associated protein OS=Sus scrofa OX=9823 GN=BCAP29 PE=1 SV=1
0 0 0 0 0 0 0 0 0 0 0 0 1 1 1 5,1 5,1 5,1
24,289 0 4,2859 10241000 7 A0A287AD92 A0A287AD92 285
tr|A0A287AD92|A0A287AD92_PIG GrpE protein homolog OS=Sus scrofa OX=9823 GN=GRPEL1 PE=1 SV=2
0 0 0 0 0 0 0 0 0 0 0 0 1 1 1 4,5 4,5 4,5
25,887 0,0011628 2,806 10686000 2 F1S924;A0A287ADI3 F1S924;A0A287ADI3
286 tr|F1S924|F1S924_PIG Coiled-coil domain containing 124 OS=Sus scrofa OX=9823 GN=CCDC124 PE=1 SV=1;tr|A0A287ADI3|A0A287ADI3_PIG Coiled-coil domain containing 124 OS=Sus scrofa OX=9823 GN=CCDC124 PE=1 SV=1
13628000 15716000 32286000 24100000 28451000 35684000 16336000 22965000 32584000 15854000
34213000 55282000 16 16 16 29 29 29 70,122 0 81,532
1493900000 86 F1RGJ3;A0A287ADJ2 F1RGJ3;A0A287ADJ2 287 tr|F1RGJ3|F1RGJ3_PIG 75 kDa glucose-regulated protein OS=Sus scrofa OX=9823 GN=HSPA9 PE=1 SV=4;tr|A0A287ADJ2|A0A287ADJ2_PIG 75 kDa glucose-regulated protein OS=Sus scrofa OX=9823 GN=HSPA9 PE=1 SV=1

0 0 0 0 0 0 0 0 0 0 0 0 2 2 2 15,8 15,8 15,8
16,838 0 26,168 178360000 11 A0A287ADK7 A0A287ADK7 288
tr|A0A287ADK7|A0A287ADK7_PIG Mitochondrial fission 1 protein OS=Sus scrofa OX=9823 GN=FIS1 PE=1 SV=1
18949000 0 13872000 9726300 14037000 0 15780000 10400000 0 11441000 12491000 14659000
6 6 6 39,5 39,5 39,5 18,96 0 28,479 300850000 29
A4GR69;A0A287ADN4;A0A5G2RH32 A4GR69;A0A287ADN4;A0A5G2RH32 289
tr|A4GR69|A4GR69_PIG Telethonin OS=Sus scrofa OX=9823 GN=TCAP PE=4 SV=1;tr|A0A287ADN4|A0A287ADN4_PIG Titin-cap OS=Sus scrofa OX=9823 GN=TCAP PE=4 SV=1;tr|A0A5G2RH32|A0A5G2RH32_PIG Titin-cap OS=Sus scrofa OX=9823 GN=TCAP PE=4 SV=1
0 0 0 0 0 0 0 0 0 0 0 0 2 2 2 25 25 25
14,632 0 5,6243 35776000 2
A0A5G2QJZ9;A0A480PK57;A0A5G2RHV1;A0A287AWQ5;A0A287ADX8;A0A5G2R5K0
A0A5G2QJZ9;A0A480PK57;A0A5G2RHV1;A0A287AWQ5;A0A287ADX8;A0A5G2R5K0 290
tr|A0A5G2QJZ9|A0A5G2QJZ9_PIG SUZ domain-containing protein 1 OS=Sus scrofa OX=9823 GN=SZRD1 PE=3 SV=1;tr|A0A480PK57|A0A480PK57_PIG SUZ domain-containing protein 1 OS=Sus scrofa OX=9823 GN=SZRD1 PE=3 SV=1;tr|A0A5G2RHV1|A0A5G2RHV1_PIG SUZ domain-containing p
11061000 10989000 38984000 0 4213500 0 0 0 3916300 5108000 3848900 0
6 6 6 29,8 29,8 29,8 26,562 0 46,193 284770000 18
A0A287AE80;A0A287BL83;A0A287BA90;A0A287BJQ5;A0A287AJT0;A0A5G2R336;Q28960;I3L7L5;A0A287AAY9;A0A286ZID9
A0A287AE80;A0A287BL83;A0A287BA90;A0A287BJQ5;A0A287AJT0 291
tr|A0A287AE80|A0A287AE80_PIG Uncharacterized protein OS=Sus scrofa OX=9823 GN=LOC110256483 PE=1 SV=1;tr|A0A287BL83|A0A287BL83_PIG Uncharacterized protein OS=Sus scrofa OX=9823 GN=LOC110256483 PE=1 SV=1;tr|A0A287BA90|A0A287BA90_PIG Uncharacterized protein O
3243900 8615800 31252000 5127500 20487000 25208000 3357700 8568300 29758000 7601100
25713000 55848000 5 5 5 14,8 14,8 14,8 42,007 0 17,597
803230000 40 A0A287AE88;F1SL07 A0A287AE88;F1SL07 292
tr|A0A287AE88|A0A287AE88_PIG NADH:ubiquinone oxidoreductase subunit A9 OS=Sus scrofa OX=9823 GN=NDUFA9 PE=1 SV=1;tr|F1SL07|F1SL07_PIG NADH:ubiquinone oxidoreductase subunit A9 OS=Sus scrofa OX=9823 GN=NDUFA9 PE=1 SV=3
0 0 0 0 0 0 0 0 0 0 0 0 1 1 1 5,1 5,1 5,1
24,852 0,0030675 1,8033 23551000 8 A0A287AXK4;A0A287AEC1;A0A5G2R9Q5
A0A287AXK4;A0A287AEC1;A0A5G2R9Q5 293 tr|A0A287AXK4|A0A287AXK4_PIG MOB kinase activator 1A OS=Sus scrofa OX=9823 GN=MOB1A PE=4 SV=2;tr|A0A287AEC1|A0A287AEC1_PIG Uncharacterized protein OS=Sus scrofa OX=9823 GN=MOB1B PE=4 SV=2;tr|A0A5G2R9Q5|A0A5G2R9Q5_PIG Uncharacterized protein OS=Sus scrofa O
7664200 0 3968000 0 0 0 0 0 0 0 0 0 2 2 2 5,9
5,9 5,9 37,011 0,0032189 2,1174 35722000 5 A0A287AEH0A0A287AEH0294

tr|A0A287AEH0|A0A287AEH0_PIG Proteasome 26S subunit, non-ATPase 7 OS=Sus scrofa OX=9823 GN=PSMD7 PE=1 SV=1
21556000 18762000 21588000 15255000 30313000 17098000 29499000 19199000 21054000 30534000 17156000 24138000 6 6 6 16,7 16,7 16,7 43,714 0 20,477 650690000 24 A0A287AEK2;P05207;A0A286ZNQ7 A0A287AEK2 296

tr|A0A287AEK2|A0A287AEK2_PIG cAMP-dependent protein kinase type II-alpha regulatory subunit OS=Sus scrofa OX=9823 GN=PRKAR2A PE=1 SV=1
0 0 0 0 0 0 0 0 0 0 0 0 1 1 1 6,7 6,7 6,7 14,763 0,0031646 2,0066 136300000 12 A0A5G2QBD4;F2Z5E6;A0A287AEM2;A0A287AKC0 A0A5G2QBD4;F2Z5E6;A0A287AEM2;A0A287AKC0 298

tr|A0A5G2QBD4|A0A5G2QBD4_PIG Ribosomal protein S5 OS=Sus scrofa OX=9823 GN=RPS5 PE=1 SV=1;tr|F2Z5E6|F2Z5E6_PIG 40S ribosomal protein S5 OS=Sus scrofa OX=9823 GN=RPS5 PE=1 SV=1;tr|A0A287AEM2|A0A287AEM2_PIG 40S ribosomal protein S5 OS=Sus scrofa OX=9823 GN=R
15218000 11230000 0 0 7421000 3734800 11653000 0 0 7276800 0 0 2 2 1 5,6 5,6 2,9 42,602 0 7,3458 100440000 13 A0A287AZH1;A0A287AEQ6;F1RYA4 A0A287AZH1;A0A287AEQ6;F1RYA4 299

tr|A0A287AZH1|A0A287AZH1_PIG Nucleosome assembly protein 1-like 4 OS=Sus scrofa OX=9823 GN=NAP1L4 PE=1 SV=1;tr|A0A287AEQ6|A0A287AEQ6_PIG Nucleosome assembly protein 1 like 4 OS=Sus scrofa OX=9823 GN=NAP1L4 PE=1 SV=2;tr|F1RYA4|F1RYA4_PIG Nucleosome assembly
0 0 0 0 0 0 0 0 0 0 0 0 1 1 1 8,9 8,9 8,9 21,997 0 3,8163 93399000 5 A0A287AEV6 A0A287AEV6 300

tr|A0A287AEV6|A0A287AEV6_PIG Uncharacterized protein OS=Sus scrofa OX=9823 PE=3 SV=2
0 0 0 0 0 0 0 0 0 0 0 0 1 1 1 4,1 4,1 4,1 31,307 0,0031447 1,9666 3178300 1 A0A5G2RB79;A0A287AF07 A0A5G2RB79;A0A287AF07 301 tr|A0A5G2RB79|A0A5G2RB79_PIG NAD-dependent protein deacylase sirtuin-5, mitochondrial OS=Sus scrofa OX=9823 GN=SIRT5 PE=1 SV=1;tr|A0A287AF07|A0A287AF07_PIG NAD-dependent protein deacylase sirtuin-5, mitochondrial OS=Sus scrofa OX=9823 GN=SIRT5 PE=1 SV=1
0 0 0 0 0 0 0 0 0 0 0 0 2 2 2 0,4 0,4 0,4 521,82 0,0086538 1,3951 387060000 8 A0A287AF97;F1RFM8 A0A287AF97;F1RFM8 302 tr|A0A287AF97|A0A287AF97_PIG Dynein axonemal heavy chain 10 OS=Sus scrofa OX=9823 GN=DNAH10 PE=3 SV=1;tr|F1RFM8|F1RFM8_PIG Dynein axonemal heavy chain 10 OS=Sus scrofa OX=9823 GN=DNAH10 PE=3 SV=3
0 8305500 8444800 0 6245200 6564900 0 5542700 8917200 0 6321300 9754400 5 4 4 7 5,5 5,5 92,385 0 6,5889 147160000 11 A0A287AFA5;Q29092;F1SRK6 A0A287AFA5;Q29092;F1SRK6 303

tr|A0A287AFA5|A0A287AFA5_PIG Endoplasmin OS=Sus scrofa OX=9823 GN=HSP90B1 PE=1 SV=2;sp|Q29092|ENPL_PIG Endoplasmin OS=Sus scrofa OX=9823 GN=HSP90B1 PE=2 SV=3;tr|F1SRK6|F1SRK6_PIG Endoplasmin OS=Sus scrofa OX=9823 GN=HSP90B1 PE=1 SV=4

0 0 0 0 0 0 0 0 0 0 0 0 7 1 1 28,8 3 3
40,18 0,0022026 2,3453 60216000 3
A0A287AJS8;A0A287AFM5;A0A287B220;K7GKG4;F1RTG8;K7GNK3
A0A287AJS8;A0A287AFM5;A0A287B220;K7GKG4;F1RTG8;K7GNK3 304
tr|A0A287AJS8|A0A287AJS8_PIG Ig-like domain-containing protein OS=Sus scrofa OX=9823 GN=LOC100513601 PE=1 SV=1;tr|A0A287AFM5|A0A287AFM5_PIG Ig-like domain-containing protein OS=Sus scrofa OX=9823 GN=LOC100513601 PE=1 SV=1;tr|A0A287B220|A0A287B220_PIG Ig-li
7146700 7331900 4032000 9758400 5694400 6504200 12983000 0 10120000 16337000 0
4383800 7 7 7 16,5 16,5 16,5 52,155 0 13,417 248080000 30
K7GQF6;A0A287AFW2;A5GFT5;A0A481CXF3 K7GQF6;A0A287AFW2;A5GFT5;A0A481CXF3
305 tr|K7GQF6|K7GQF6_PIG Aminopeptidase like 1 OS=Sus scrofa OX=9823 GN=NPEPL1 PE=1 SV=1;tr|A0A287AFW2|A0A287AFW2_PIG Aminopeptidase like 1 OS=Sus scrofa OX=9823 GN=NPEPL1 PE=1 SV=2;tr|A5GFT5|A5GFT5_PIG Aminopeptidase-like 1 OS=Sus scrofa OX=9823 GN=NPEPL1 PE=
0 0 0 0 0 0 0 0 0 0 0 0 1 1 1 16,4 16,4 16,4
12,618 0 53,037 21760000 2 A0A287AI10;A0A287AG00 A0A287AI10;A0A287AG00
306 tr|A0A287AI10|A0A287AI10_PIG Uncharacterized protein OS=Sus scrofa OX=9823 PE=1 SV=1;tr|A0A287AG00|A0A287AG00_PIG Uncharacterized protein OS=Sus scrofa OX=9823 PE=1 SV=2
2625500 0 5352700 0 0 4104400 4372300 0 0 0 0 0 3 3
3 14,1 14,1 14,1 22,513 0 3,6281 47153000 10 A0A287AG70;Q8MJ14
A0A287AG70;Q8MJ14 307 tr|A0A287AG70|A0A287AG70_PIG Glutathione peroxidase OS=Sus scrofa OX=9823 GN=GPX1 PE=3 SV=2;sp|Q8MJ14|GPX1_PIG Glutathione peroxidase 1 OS=Sus scrofa OX=9823 GN=GPX1 PE=2 SV=2
485150000 178740000 122720000 393950000 193000000 169710000 543820000
165280000 154650000 349650000 150920000 56050000 6 6 6
38,2 38,2 38,2 20,9 0 37,944 9605200000 281 A0A287AGJ6;Q4JH15;A0A287AVB7
A0A287AGJ6;Q4JH15;A0A287AVB7 308 tr|A0A287AGJ6|A0A287AGJ6_PIG Troponin I2, fast skeletal type OS=Sus scrofa OX=9823 GN=TNNI2 PE=4 SV=1;tr|Q4JH15|Q4JH15_PIG Troponin I OS=Sus scrofa OX=9823 GN=TNNI2 PE=2 SV=1;tr|A0A287AVB7|A0A287AVB7_PIG Troponin I2, fast skeletal type OS=Sus scrofa OX=982
0 0 0 0 0 0 0 0 0 0 0 0 1 1 1 2,5 2,5 2,5
48,767 0,0011507 2,7415 31610000 4 A0A287AGM8;Q29381 A0A287AGM8;Q29381
309 tr|A0A287AGM8|A0A287AGM8_PIG Dolichyl-diphosphooligosaccharide--protein glycosyltransferase 48 kDa subunit OS=Sus scrofa OX=9823 GN=DDOST PE=1 SV=1;sp|Q29381|OST48_PIG Dolichyl-diphosphooligosaccharide--protein glycosyltransferase 48 kDa subunit OS=Sus scr
127500000 187810000 474300000 96310000 325370000 353400000 85062000
165500000 264410000 89006000 319910000 645270000 21 21 21
35,8 35,8 35,8 58,254 0 129,35 22730000000 389
F1RPS8;A0A287AGU2;P80021;A0A287BBS4;A0A5G2QY15;F1RPT1;A0A5G2R5C5;A0A287A874;A0A287BRD5

F1RPS8;A0A287AGU2;P80021;A0A287BBS4;A0A5G2QY15;F1RPT1;A0A5G2R5C5;A0A287A87 4;A0A287BRD5 310 tr|F1RPS8|F1RPS8_PIG ATP synthase subunit alpha OS=Sus scrofa OX=9823 GN=ATP5F1A PE=1 SV=3;tr|A0A287AGU2|A0A287AGU2_PIG ATP synthase subunit alpha OS=Sus scrofa OX=9823 GN=ATP5F1A PE=1 SV=1;sp|P80021|ATPA_PIG ATP synthase subunit alpha, mitochondrial OS=Su
5761400 4623600 7285900 11595000 11042000 9939300 9177700 5692200 8577100 11310000
11574000 15633000 5 5 5 26,5 26,5 26,5 25,64 0 18,582
343940000 24
A0A287AH85;A0A5G2QKQ8;A0A5G2QNB5;A0A5G2Q7V3;A0A5G2R079;A0A5G2QK65
A0A287AH85;A0A5G2QKQ8;A0A5G2QNB5;A0A5G2Q7V3;A0A5G2R079;A0A5G2QK65 311
tr|A0A287AH85|A0A287AH85_PIG Adenylate kinase 2, mitochondrial OS=Sus scrofa OX=9823 GN=AK2 PE=1 SV=1;tr|A0A5G2QKQ8|A0A5G2QKQ8_PIG Adenylate kinase 2, mitochondrial OS=Sus scrofa OX=9823 GN=AK2 PE=1 SV=1;tr|A0A5G2QNB5|A0A5G2QNB5_PIG Adenylate kinase 2, mit
10482000 10240000 18827000 14273000 6859100 20633000 14179000 11991000 15334000 17245000
8557900 14784000 4 4 4 18,9 18,9 18,9 26,369 0 30,982
344440000 24 Q6PP07;A0A287AHI7;A0A287ASX3 Q6PP07;A0A287AHI7;A0A287ASX3
312 tr|Q6PP07|Q6PP07_PIG Adiponectin OS=Sus scrofa OX=9823 GN=ADIPOQ PE=1 SV=1;tr|A0A287AHI7|A0A287AHI7_PIG Adiponectin, C1Q and collagen domain containing OS=Sus scrofa OX=9823 GN=ADIPOQ PE=1 SV=1;tr|A0A287ASX3|A0A287ASX3_PIG Adiponectin, C1Q and collagen dom
14591000 10190000 8385100 15043000 0 17116000 17366000 0 8657800 16962000 10309000
9662300 6 6 6 16,8 16,8 16,8 48,152 0 18,212 346260000 31
I3LN42;A0A287AHK1;CON__Q3MHN5;CON__ENSEMBL:ENSBTAP00000018229
I3LN42;A0A287AHK1 313 tr|I3LN42|I3LN42_PIG Gc-globulin OS=Sus scrofa OX=9823 GN=GC PE=1 SV=3;tr|A0A287AHK1|A0A287AHK1_PIG Gc-globulin OS=Sus scrofa OX=9823 GN=GC PE=1 SV=1
188760000 131300000 73444000 132980000 54034000 59775000 202880000 55355000
67679000 152120000 42807000 41967000 10 9 9 56,8 52 52 16,706
0 55,298 3347300000 130
A0A287AHS0;F2Z5G3;A0A5G2QWK6;A0A5G2QTD3;A0A287AUG4;A0A5G2Q7N5;A0A5G2Q EH3;F1S145;A0A287B857;A0A287ANW8;A0A287AUC1
A0A287AHS0;F2Z5G3;A0A5G2QWK6;A0A5G2QTD3;A0A287AUG4;A0A5G2Q7N5;A0A5G2Q EH3;F1S145 314 tr|A0A287AHS0|A0A287AHS0_PIG Uncharacterized protein OS=Sus scrofa OX=9823 GN=CALM3 PE=1 SV=1;tr|F2Z5G3|F2Z5G3_PIG Calmodulin-2 isoform 1 OS=Sus scrofa OX=9823 GN=STPG4 PE=1 SV=1;tr|A0A5G2QWK6|A0A5G2QWK6_PIG Calmodulin OS=Sus scrofa OX=9823 GN=CALM3 PE=1 S
4430700 27544000 14980000 0 0 0 0 0 0 0 0 0 6 6 3
15,3 15,3 7 52,175 0 10,785 117940000 11
A0A5G2QS23;A0A287AHX0;A0A5G2R491;A0A5G2R4J6;A0A2C9F383;Q95266;A0A5G2QQ20; A0A5G2RCS0;A0A5G2QUE4
A0A5G2QS23;A0A287AHX0;A0A5G2R491;A0A5G2R4J6;A0A2C9F383;Q95266;A0A5G2QQ20; A0A5G2RCS0;A0A5G2QUE4 315 tr|A0A5G2QS23|A0A5G2QS23_PIG Calcium/calmodulin-

dependent protein kinase OS=Sus scrofa OX=9823 GN=CAMK2D PE=3 SV=1;tr|A0A287AHX0|A0A287AHX0_PIG Calcium/calmodulin-dependent protein kinase OS=Sus scrofa OX=9823 GN=CAMK2D PE=3 SV=2;tr|A0A5G2R491|A0A5G2R491_P
0 3215800 0 0 0 0 0 0 5716800 0 0 0 3 3 3 3,1
3,1 3,1 114,95 0 4,3209 36603000 6 A0A287AKY9;A0A287AI85
A0A287AKY9;A0A287AI85 316 tr|A0A287AKY9|A0A287AKY9_PIG Formin homology 2 domain containing 1 OS=Sus scrofa OX=9823 GN=FHOD1 PE=1 SV=1;tr|A0A287AI85|A0A287AI85_PIG Formin homology 2 domain containing 1 OS=Sus scrofa OX=9823 GN=FHOD1 PE=1 SV=2
306310000 82302000 408100000 872220000 310600000 517210000 679110000
174250000 174920000 352150000 208720000 610590000 23 13
0 53,9 33,1 0 32,929 0 118,3 10043000000 202
A0A287AID2;A0A5G2RN24;A0A5G2RJ89 A0A287AID2;A0A5G2RN24;A0A5G2RJ89 317
tr|A0A287AID2|A0A287AID2_PIG Tropomyosin alpha-3 chain OS=Sus scrofa OX=9823 GN=TPM3 PE=1 SV=1;tr|A0A5G2RN24|A0A5G2RN24_PIG Tropomyosin alpha-3 chain OS=Sus scrofa OX=9823 GN=TPM3 PE=1 SV=1;tr|A0A5G2RJ89|A0A5G2RJ89_PIG Tropomyosin alpha-3 chain OS=Sus scro
83907000 27445000 61754000 8929000 32789000 31228000 23282000 20687000 41018000 24913000
24678000 51866000 14 14 13 23,1 23,1 22 67,554 0 71,923
2300600000 110 A0A287AIE4;A0A287BIK3;A0A5G2R498 A0A287AIE4;A0A287BIK3 318
tr|A0A287AIE4|A0A287AIE4_PIG BTB domain-containing protein OS=Sus scrofa OX=9823 GN=KLHL41 PE=1 SV=2;tr|A0A287BIK3|A0A287BIK3_PIG BTB domain-containing protein OS=Sus scrofa OX=9823 GN=KLHL41 PE=1 SV=1
0 0 0 0 0 0 0 0 0 0 0 0 1 1 1 2,6 2,6 2,6
38,663 0,0031881 2,0523 30570000 4 A0A287AIE6 A0A287AIE6 319
tr|A0A287AIE6|A0A287AIE6_PIG ATPase GET3 OS=Sus scrofa OX=9823 GN=GET3 PE=1 SV=2
27174000 17867000 39850000 37032000 27422000 33371000 42961000 16366000 19819000 42644000
15125000 47855000 2 2 2 4,9 4,9 4,9 45,938 0 4,2462
644400000 17 A0A287AIJ7;A0A5K1VA80;A0A5K1VA81;Q3HUX1;K7GQE1;A0A5G2QC71
A0A287AIJ7;A0A5K1VA80;A0A5K1VA81;Q3HUX1;K7GQE1;A0A5G2QC71 320
tr|A0A287AIJ7|A0A287AIJ7_PIG Glycoprotein IIIb OS=Sus scrofa OX=9823 GN=CD36 PE=1 SV=1;tr|A0A5K1VA80|A0A5K1VA80_PIG Glycoprotein IIIb OS=Sus scrofa OX=9823 GN=CD36 PE=1 SV=1;tr|A0A5K1VA81|A0A5K1VA81_PIG Glycoprotein IIIb OS=Sus scrofa OX=9823 GN=CD36 PE=1
16403000 12311000 14839000 0 12630000 0 0 0 0 0 0 0 6 6
6 12,6 12,6 12,6 42,921 0 12,821 165220000 14
P07802;A0A287AKY1;A0A287AJA4 P07802;A0A287AKY1;A0A287AJA4 321
sp|P07802|KAP0_PIG cAMP-dependent protein kinase type I-alpha regulatory subunit OS=Sus scrofa OX=9823 GN=PRKAR1A PE=1 SV=2;tr|A0A287AKY1|A0A287AKY1_PIG cAMP-dependent protein kinase type I-alpha regulatory subunit OS=Sus scrofa OX=9823 GN=PRKAR1A PE=3 SV=
0 4599800 6248800 6400900 0 6219500 0 0 6115000 6248300 0 0
2 2 2 15,2 15,2 15,2 17,818 0 5,9263 106320000 8
A0A5G2R7M9;A0A287AJH8;A0A5G2R3M1;A0A5G2QR46;A0A288CG20;A0A287AP66;I3LBH4

A0A5G2R7M9;A0A287AJH8;A0A5G2R3M1;A0A5G2QR46;A0A288CG20;A0A287AP66;I3LBH4 322 tr|A0A5G2R7M9|A0A5G2R7M9_PIG 60S ribosomal protein L12 OS=Sus scrofa OX=9823 GN=RPL12 PE=1 SV=1;tr|A0A287AJH8|A0A287AJH8_PIG 60S ribosomal protein L12 OS=Sus scrofa OX=9823 GN=RPL12 PE=1 SV=1;tr|A0A5G2R3M1|A0A5G2R3M1_PIG 60S ribosomal protein L12 OS=Sus sc

0 0 0 0 0 0 0 0 0 0 0 0 5 1 1 18,9 6,3 6,3 28,804 0 14,387 61701000 9 A0A287AJQ2 A0A287AJQ2 323 tr|A0A287AJQ2|A0A287AJQ2_PIG Phosphoglycerate mutase OS=Sus scrofa OX=9823 GN=PGAM1 PE=1 SV=1

26410000 11295000 37225000 22809000 40618000 28782000 28443000 33418000 20346000 39888000 55336000 59292000 13 13 13 23,6 23,6 23,6 70,759 0 72,444 1917000000 116 A0A287AJY7;F1RH92 A0A287AJY7;F1RH92 324 tr|A0A287AJY7|A0A287AJY7_PIG Uncharacterized protein OS=Sus scrofa OX=9823 GN=MYOT PE=4 SV=2;tr|F1RH92|F1RH92_PIG Uncharacterized protein OS=Sus scrofa OX=9823 GN=MYOT PE=4 SV=2

8875500 5392500 5828300 7526000 4417500 6460000 12192000 0 5085600 11210000 0 4565800 8 4 4 13,2 6,8 6,8 67,66 0 11,044 183810000 27 P26042;A0A287BED9;F1RTN3;A0A287AK19;A0A5G2R8G3 P26042;A0A287BED9;F1RTN3;A0A287AK19;A0A5G2R8G3 325 sp|P26042|MOES_PIG Moesin OS=Sus scrofa OX=9823 GN=MSN PE=2 SV=3;tr|A0A287BED9|A0A287BED9_PIG Moesin OS=Sus scrofa OX=9823 GN=MSN PE=1 SV=2;tr|F1RTN3|F1RTN3_PIG Moesin OS=Sus scrofa OX=9823 GN=MSN PE=1 SV=3;tr|A0A287AK19|A0A287AK19_PIG Moesin OS=Sus scrofa

9380700 0 8761300 13478000 4491900 6283900 14398000 0 6573200 12003000 0 11730000 5 5 5 24,6 24,6 24,6 22,377 0 11,022 246790000 21 A0A287AK21;A0A287BK36;F1RJ93;A0A287AIA2;A0A286ZN10 A0A287AK21;A0A287BK36;F1RJ93;A0A287AIA2 326 tr|A0A287AK21|A0A287AK21_PIG Transgelin OS=Sus scrofa OX=9823 GN=TAGLN2 PE=1 SV=1;tr|A0A287BK36|A0A287BK36_PIG Transgelin OS=Sus scrofa OX=9823 GN=TAGLN2 PE=1 SV=1;tr|F1RJ93|F1RJ93_PIG Transgelin OS=Sus scrofa OX=9823 GN=TAGLN2 PE=1 SV=3;tr|A0A287AIA2|A0A2

15957000 4195100 8386100 13178000 6327400 21828000 12113000 0 17180000 26275000 6017500 13480000 9 9 9 12,1 12,1 12,1 86,156 0 28,645 618030000 52 A0A287AKQ1;A0A287B0G1;A0A287B6D7;A0A287B794;A0A287AYR9;A0A287B3U0;A0A287BQ55 A0A287AKQ1;A0A287B0G1;A0A287B6D7;A0A287B794;A0A287AYR9;A0A287B3U0;A0A287BQ55 327 tr|A0A287AKQ1|A0A287AKQ1_PIG Sarcolemma associated protein OS=Sus scrofa OX=9823 GN=SLMAP PE=4 SV=2;tr|A0A287B0G1|A0A287B0G1_PIG Sarcolemma associated protein OS=Sus scrofa OX=9823 GN=SLMAP PE=4 SV=1;tr|A0A287B6D7|A0A287B6D7_PIG Sarcolemma associated prote

0 0 28880000 0 0 12085000 0 0 5423300 0 0 7323500 2 2 2 9,9 9,9 9,9 21,185 0,0021978 2,3387 133680000 7 A0A287AL10 A0A287AL10 328 tr|A0A287AL10|A0A287AL10_PIG Ferritin OS=Sus scrofa OX=9823 GN=FTH1 PE=1 SV=2

0 0 0 0 0 0 0 0 0 0 0 0 3 3 3 2,3 2,3 2,3
122,72 0 3,1587 51425000 5
A0A5G2R7W5;F1RLQ9;I3LEY6;A0A287BD60;A0A287AL38;A0A287AIK6
A0A5G2R7W5;F1RLQ9;I3LEY6;A0A287BD60;A0A287AL38 329
tr|A0A5G2R7W5|A0A5G2R7W5_PIG Tripeptidyl-peptidase 2 OS=Sus scrofa OX=9823 GN=TPP2 PE=1 SV=1;tr|F1RLQ9|F1RLQ9_PIG Tripeptidyl-peptidase 2 OS=Sus scrofa OX=9823 GN=TPP2 PE=1 SV=4;tr|I3LEY6|I3LEY6_PIG Tripeptidyl-peptidase 2 OS=Sus scrofa OX=9823 GN=TPP2 PE=
281500000 26464000 5202000 39194000 3389100 2752500 118380000 7329100 0
220870000 2950900 0 4 4 4 33,3 33,3 33,3 15,218 0 21,157
2102900000 57 A0A287ALJ2 A0A287ALJ2 331 tr|A0A287ALJ2|A0A287ALJ2_PIG Parvalbumin OS=Sus scrofa OX=9823 GN=PVALB PE=1 SV=1
17613000 13826000 19999000 18626000 10564000 22108000 23269000 7637100 15825000 23272000
11415000 10271000 8 8 8 13 13 13 78,227 0 27,58
626460000 52
A8D737;A0A287ALP4;A0A5G2QF74;A0A5G2Q9R1;A0A5G2QBL3;A0A5G2QB64;A0A5G2QPP
8 A8D737;A0A287ALP4;A0A5G2QF74;A0A5G2Q9R1;A0A5G2QBL3;A0A5G2QB64 332
tr|A8D737|A8D737_PIG Cadherin-13 OS=Sus scrofa OX=9823 GN=CDH13 PE=1 SV=1;tr|A0A287ALP4|A0A287ALP4_PIG Cadherin-13 OS=Sus scrofa OX=9823 GN=CDH13 PE=1 SV=1;tr|A0A5G2QF74|A0A5G2QF74_PIG Cadherin-13 OS=Sus scrofa OX=9823 GN=CDH13 PE=1 SV=1;tr|A0A5G2Q9R1|A0A5
0 0 0 0 0 0 0 0 0 0 0 0 49 1 0 26,5 0,6 0
223 0,0032328 2,13 250670000 11
A0A287AMA4;A0A5G2QRY9;F1SS62;A0A5G2QLY9;A0A5G2R196
A0A287AMA4;A0A5G2QRY9;F1SS62;A0A5G2QLY9;A0A5G2R196 333
tr|A0A287AMA4|A0A287AMA4_PIG Uncharacterized protein OS=Sus scrofa OX=9823 GN=MYH8 PE=3 SV=2;tr|A0A5G2QRY9|A0A5G2QRY9_PIG Uncharacterized protein OS=Sus scrofa OX=9823 GN=MYH8 PE=3 SV=1;tr|F1SS62|F1SS62_PIG Uncharacterized protein OS=Sus scrofa OX=9823 GN=
10352000 15247000 31724000 5621700 20822000 22126000 3782800 14194000 24406000 14725000
26989000 56664000 20 20 20 27,1 27,1 27,1 81,521 0 74,787
1592800000 102
A0A5G2QM23;A0A287AMM6;A0A287A2N6;I3LSM7;A0A5G2QEH4;A0A5G2QII1
A0A5G2QM23;A0A287AMM6;A0A287A2N6;I3LSM7;A0A5G2QEH4 334
tr|A0A5G2QM23|A0A5G2QM23_PIG MICOS complex subunit MIC60 OS=Sus scrofa OX=9823 GN=IMMT PE=1 SV=1;tr|A0A287AMM6|A0A287AMM6_PIG MICOS complex subunit MIC60 OS=Sus scrofa OX=9823 GN=IMMT PE=1 SV=1;tr|A0A287A2N6|A0A287A2N6_PIG MICOS complex subunit MIC60 OS=Su
0 25515000 26869000 41326000 20688000 30257000 22709000 45793000 66346000 44921000 40501000
63341000 2 2 2 16,3 16,3 16,3 16,465 0 4,9591 652500000 21
I3LQ38;A0A5G2QZZ2;A0A287AMN2I3LQ38;A0A5G2QZZ2;A0A287AMN2335
tr|I3LQ38|I3LQ38_PIG NADH dehydrogenase [ubiquinone] iron-sulfur protein 4, mitochondrial OS=Sus scrofa OX=9823 GN=NDUFS4 PE=1

SV=2;tr|A0A5G2QZZ2|A0A5G2QZZ2_PIG NADH dehydrogenase [ubiquinone] iron-sulfur protein 4, mitochondrial OS=Sus scrofa OX=9823 GN=N

0 0 0 0 0 0 0 0 0 0 0 0 1 1 1 1,2 1,2 1,2

110,22 0,0022002 2,3435 13749000000 25 I3LVA1;A0A287AMT7

I3LVA1;A0A287AMT7 336 tr|I3LVA1|I3LVA1_PIG HECT and RLD domain containing E3 ubiquitin protein ligase family member 6 OS=Sus scrofa OX=9823 GN=HERC6 PE=4 SV=2;tr|A0A287AMT7|A0A287AMT7_PIG HECT and RLD domain containing E3 ubiquitin protein ligase family member 6 OS=Sus scrofa O

3893600 9706100 7439000 4932600 7020700 8464900 3426000 9311900 8762700 2611200

6521800 7351300 8 8 8 15 15 15 56,55 0 15,605

293760000 44 A0A5G2Q9C7;A0A5G2R2Z6;A0A287AMZ2;A0A287AUX8;F1RP17

A0A5G2Q9C7;A0A5G2R2Z6;A0A287AMZ2;A0A287AUX8;F1RP17 337

tr|A0A5G2Q9C7|A0A5G2Q9C7_PIG T-complex protein 1 subunit gamma OS=Sus scrofa OX=9823 GN=CCT3 PE=1 SV=1;tr|A0A5G2R2Z6|A0A5G2R2Z6_PIG T-complex protein 1 subunit gamma OS=Sus scrofa OX=9823 GN=CCT3 PE=1 SV=1;tr|A0A287AMZ2|A0A287AMZ2_PIG T-complex protein 1 s

6714300 10090000 25445000 10154000 26206000 20549000 7457900 12756000 16518000 9022200

27525000 41700000 8 8 8 26,4 26,4 26,4 34,106 0 34,52

1371700000 78 A0A287AN76;A0A5G2QT96;F1RFX9;A0A5G2QC86

A0A287AN76;A0A5G2QT96;F1RFX9;A0A5G2QC86 339 tr|A0A287AN76|A0A287AN76_PIG Mitochondrial 2-oxoglutarate/malate carrier protein isoform 1 OS=Sus scrofa OX=9823 GN=SLC25A11 PE=1 SV=1;tr|A0A5G2QT96|A0A5G2QT96_PIG Solute carrier family 25 member 11 OS=Sus scrofa OX=9823 GN=SLC25A11 PE=1 SV=1;tr|F1RFX9|F1R

0 0 10183000 0 4880500 16854000 0 1575800 3335000 0 2186600 20564000

12 12 12 10 10 10 157,18 0 62,337 363380000 29

A0A5G2Q795;A0A287BKL4;A0A287ANE3 A0A5G2Q795;A0A287BKL4;A0A287ANE3 340

tr|A0A5G2Q795|A0A5G2Q795_PIG Myomesin 3 OS=Sus scrofa OX=9823 GN=MYOM3 PE=4 SV=1;tr|A0A287BKL4|A0A287BKL4_PIG Myomesin 3 OS=Sus scrofa OX=9823 GN=MYOM3 PE=4 SV=2;tr|A0A287ANE3|A0A287ANE3_PIG Myomesin 3 OS=Sus scrofa OX=9823 GN=MYOM3 PE=4 SV=1

1511500000 1088800000 563570000 1577200000 1031900000 655970000 1871300000

616890000 746080000 1159100000 840090000 301110000 5 1

1 15,6 2,3 2,3 42,649 0 15,555 21984000000 218

A0A287ANH6;A0A287AVF3;I3LCN1 A0A287ANH6;A0A287AVF3;I3LCN1 341

tr|A0A287ANH6|A0A287ANH6_PIG 2-phospho-D-glycerate hydro-lyase OS=Sus scrofa OX=9823 GN=ENO2 PE=1 SV=2;tr|A0A287AVF3|A0A287AVF3_PIG 2-phospho-D-glycerate hydro-lyase OS=Sus scrofa OX=9823 GN=ENO2 PE=1 SV=2;tr|I3LCN1|I3LCN1_PIG 2-phospho-D-glycerate hydro-l

0 0 0 0 0 0 0 0 0 0 0 0 5 1 1 18,3 2,8 2,8

34,393 0 3,8086 221050000 3 A0A287ANN7;F1SEV8;I3LT08

A0A287ANN7;F1SEV8;I3LT08 342 tr|A0A287ANN7|A0A287ANN7_PIG Uncharacterized protein OS=Sus scrofa OX=9823 GN=EEF1D PE=1 SV=2;tr|F1SEV8|F1SEV8_PIG Uncharacterized

protein OS=Sus scrofa OX=9823 GN=EEF1D PE=1 SV=4;tr|I3LT08|I3LT08_PIG Uncharacterized protein OS=Sus scrofa OX=9823 GN=EEF1D
0 0 0 0 0 0 0 0 0 0 0 0 3 3 3 10,2 10,2 10,2
29,974 0,0022148 2,3723 137960000 4
A0A5G2RIJ3;A9XFX6;A0A287AXJ3;A0A288CG52;A0PFK7;A0A287AP52;A0A5K1U188
A0A5G2RIJ3;A9XFX6;A0A287AXJ3;A0A288CG52;A0PFK7;A0A287AP52;A0A5K1U188 343
tr|A0A5G2RIJ3|A0A5G2RIJ3_PIG F-actin-capping protein subunit beta OS=Sus scrofa OX=9823 GN=CAPZB PE=1 SV=1;tr|A9XFX6|A9XFX6_PIG F-actin-capping protein subunit beta OS=Sus scrofa OX=9823 GN=CAPZB PE=1 SV=1;tr|A0A287AXJ3|A0A287AXJ3_PIG F-actin-capping prote
0 30808000 57548000 0 39218000 33720000 0 45221000 35664000 16852000 55695000 50560000
2 2 2 2,5 2,5 2,5 114,6 0,0031813 2,037 557420000 11
F1S6A0;A0A5G2R4T4;A0A287APG7;A0A5G2RFA4;A0A5G2R3C6
F1S6A0;A0A5G2R4T4;A0A287APG7;A0A5G2RFA4;A0A5G2R3C6 344
tr|F1S6A0|F1S6A0_PIG Axonemal dynein light chain domain containing 1 OS=Sus scrofa OX=9823 GN=AXDND1 PE=4 SV=4;tr|A0A5G2R4T4|A0A5G2R4T4_PIG Axonemal dynein light chain domain containing 1 OS=Sus scrofa OX=9823 GN=AXDND1 PE=4 SV=1;tr|A0A287APG7|A0A287APG7_P
12668000 0 8191500 0 0 8670300 0 0 0 0 0 0 2 2 2
12,4 12,4 12,4 23,276 0 11,197 66834000 7 A0A5G2QQY5;I3LL97;A0A287APN0
A0A5G2QQY5;I3LL97;A0A287APN0 345 tr|A0A5G2QQY5|A0A5G2QQY5_PIG Cysteine and glycine rich protein 1 OS=Sus scrofa OX=9823 GN=CSRP1 PE=1 SV=1;tr|I3LL97|I3LL97_PIG Cysteine and glycine rich protein 1 OS=Sus scrofa OX=9823 GN=CSRP1 PE=1 SV=2;tr|A0A287APN0|A0A287APN0_PIG Cysteine and glycine ri
0 0 0 0 0 0 0 0 0 0 0 0 1 1 1 9,5 9,5 9,5
16,177 0,0031612 1,9972 14123000 1 F1S935;A0A287APR1 F1S935;A0A287APR1
346 tr|F1S935|F1S935_PIG 60S ribosomal protein L18a OS=Sus scrofa OX=9823 GN=RPL18A PE=1 SV=4;tr|A0A287APR1|A0A287APR1_PIG 60S ribosomal protein L18a OS=Sus scrofa OX=9823 GN=RPL18A PE=1 SV=1
22880000 21030000 21175000 16764000 17894000 23744000 23561000 22991000 24467000 26705000
18930000 13747000 6 6 6 16,5 16,5 16,5 48,75 0 26,719
498330000 25 A0A287AR93;A0A287AQ12;F1SIC0;A0A287ATC5;A0A287BQ85
A0A287AR93;A0A287AQ12;F1SIC0;A0A287ATC5;A0A287BQ85 347
tr|A0A287AR93|A0A287AR93_PIG Protein kinase C and casein kinase substrate in neurons 3 OS=Sus scrofa OX=9823 GN=PACSIN3 PE=1 SV=1;tr|A0A287AQ12|A0A287AQ12_PIG Protein kinase C and casein kinase substrate in neurons 3 OS=Sus scrofa OX=9823 GN=PACSIN3 PE=1 S
29436000 11730000 14595000 10825000 0 11760000 23408000 17045000 12344000 35040000 6490900
13252000 5 5 5 21,9 21,9 21,9 30,2 0 16,731 393960000 28
A0A287AQC2;E7EI20;A0A287BSV0 A0A287AQC2;E7EI20;A0A287BSV0 348
tr|A0A287AQC2|A0A287AQC2_PIG Rho GDP dissociation inhibitor alpha OS=Sus scrofa OX=9823 GN=ARHGDIA PE=3 SV=1;tr|E7EI20|E7EI20_PIG Rho GDP dissociation inhibitor alpha OS=Sus scrofa OX=9823 GN=ARHGDIA PE=2 SV=1;tr|A0A287BSV0|A0A287BSV0_PIG Rho GDP dissociat

2978600 24643000 69312000 5754600 58117000 69251000 3152800 33696000 51692000 7845300

59179000 143300000 29 29 29 27,5 27,5 27,5 113,89 0 120,87

6403200000 196 F1SMG3;A0A287B1U9;A0A287AQG1;A0A286ZZT0

F1SMG3;A0A287B1U9;A0A287AQG1;A0A286ZZT0 349 tr|F1SMG3|F1SMG3_PIG Proton-translocating NAD(P)(+) transhydrogenase OS=Sus scrofa OX=9823 GN=NNT PE=1 SV=2;tr|A0A287B1U9|A0A287B1U9_PIG Proton-translocating NAD(P)(+) transhydrogenase OS=Sus scrofa OX=9823 GN=NNT PE=1 SV=1;tr|A0A287AQG1|A0A287AQG1_PIG Pro

14030000 0 12311000 37753000 0 13148000 30631000 0 7661900 45610000 0 10855000

8 8 2 32,4 32,4 6,6 39,979 0 98,575 740980000 29 A0A287AQG3

A0A287AQG3 350 tr|A0A287AQG3|A0A287AQG3_PIG Ig-like domain-containing protein OS=Sus scrofa OX=9823 GN=LOC100513601 PE=1 SV=1

0 0 0 0 0 0 0 0 0 0 0 0 2 2 2 4,1 4,1 4,1

62,656 0,0087634 1,4575 3816700 2 A0A287BGU5;A0A287AQG4;F2Z5D4

A0A287BGU5;A0A287AQG4;F2Z5D4 351 tr|A0A287BGU5|A0A287BGU5_PIG

Uncharacterized protein OS=Sus scrofa OX=9823 GN=SYNCRIP PE=1 SV=1;tr|A0A287AQG4|A0A287AQG4_PIG Uncharacterized protein OS=Sus scrofa OX=9823 GN=SYNCRIP PE=1 SV=2;tr|F2Z5D4|F2Z5D4_PIG Uncharacterized protein OS=Sus scrofa OX=98

43469000 81844000 50687000 5694900 73777000 13204000 23367000 44926000 31924000 11479000

33156000 22735000 20 20 12 30,2 30,2 18,4 84,774 0 120,29

3245700000 133 O02705;A0A287B8J5;A0A287AQK7 O02705;A0A287B8J5;A0A287AQK7

352 sp|O02705|HS90A_PIG Heat shock protein HSP 90-alpha OS=Sus scrofa OX=9823 GN=HSP90AA1 PE=2 SV=3;tr|A0A287B8J5|A0A287B8J5_PIG Heat shock protein HSP 90-alpha OS=Sus scrofa OX=9823 GN=HSP90AA1 PE=3 SV=2;tr|A0A287AQK7|A0A287AQK7_PIG Heat shock protein HSP 90-

82919000 26719000 155120000 17159000 61482000 84578000 14635000 20386000 57916000 49634000

60743000 159720000 5 5 5 38,5 38,5 38,5 16,75 0 23,807

2153700000 60 F1RM62;A0A287AQR8 F1RM62;A0A287AQR8 353

tr|F1RM62|F1RM62_PIG Heat shock protein family B (small) member 6 OS=Sus scrofa OX=9823 GN=HSPB6 PE=1 SV=2;tr|A0A287AQR8|A0A287AQR8_PIG Heat shock protein family B (small) member 6 OS=Sus scrofa OX=9823 GN=HSPB6 PE=1 SV=1

0 0 0 0 4431000 0 0 0 0 0 4450400 7958600 3 3 3

6,9 6,9 6,9 53,427 0 6,6707 47000000 12

A0A5G2QIN8;A0A287AXP0;A0A287AR05;F1RUE3

A0A5G2QIN8;A0A287AXP0;A0A287AR05;F1RUE3 354 tr|A0A5G2QIN8|A0A5G2QIN8_PIG Aldehyde dehydrogenase 5 family member A1 OS=Sus scrofa OX=9823 GN=ALDH5A1 PE=1 SV=1;tr|A0A287AXP0|A0A287AXP0_PIG Succinate-semialdehyde dehydrogenase OS=Sus scrofa OX=9823 GN=ALDH5A1 PE=1 SV=1;tr|A0A287AR05|A0A287AR05_PIG Succ

31925000 73395000 120770000 0 35880000 40995000 0 19630000 22390000 0 31912000

109850000 12 12 12 27,2 27,2 27,2 43,811 0 41,155 1471500000

44 F1RK10;A0A287AR48;O97580 F1RK10;A0A287AR48;O97580 355

tr|F1RK10|F1RK10_PIG Succinate--CoA ligase [ADP-forming] subunit beta, mitochondrial OS=Sus scrofa OX=9823 GN=SUCLA2 PE=1 SV=3;tr|A0A287AR48|A0A287AR48_PIG Succinate--CoA ligase [ADP-forming] subunit beta, mitochondrial OS=Sus scrofa OX=9823 GN=SUCLA2 PE=1

39062000 0 0 25716000 0 0 41085000 0 0 21224000 0 0 2 2 2 5,8 5,8 5,8 33,486 0,0030242 1,7407 288450000 8 F1SD96;A0A5G2QPT2;A0A287ARC6 F1SD96;A0A5G2QPT2;A0A287ARC6 356 tr|F1SD96|F1SD96_PIG UV excision repair protein RAD23 OS=Sus scrofa OX=9823 GN=RAD23A PE=1 SV=3;tr|A0A5G2QPT2|A0A5G2QPT2_PIG UV excision repair protein RAD23 OS=Sus scrofa OX=9823 GN=RAD23A PE=1 SV=1;tr|A0A287ARC6|A0A287ARC6_PIG UV excision repair protein

0 0 0 0 0 0 0 0 0 0 0 0 3 3 3 14,2 14,2 14,2 18,578 0 3,3226 18663000 5 A0A5G2R017;A0A5G2R6J9;A0A287ARU4;A0A5G2RC35 A0A5G2R017;A0A5G2R6J9;A0A287ARU4;A0A5G2RC35 357 tr|A0A5G2R017|A0A5G2R017_PIG S-phase kinase-associated protein 1 OS=Sus scrofa OX=9823 GN=SKP1 PE=3 SV=1;tr|A0A5G2R6J9|A0A5G2R6J9_PIG S-phase kinase-associated protein 1 OS=Sus scrofa OX=9823 GN=SKP1 PE=3 SV=1;tr|A0A287ARU4|A0A287ARU4_PIG S-phase kinase-as

0 0 0 0 0 0 0 0 0 0 0 0 11 1 1 46,9 6,2 6,2 27,209 0,0030769 1,8286 9999900 2 A0A287ARU7A0A287ARU7358 tr|A0A287ARU7|A0A287ARU7_PIG 14_3_3 domain-containing protein OS=Sus scrofa OX=9823 GN=CRK PE=1 SV=2

10238000 14745000 47607000 12025000 34572000 39743000 8349900 15270000 39920000 11088000 32180000 66924000 18 18 18 26 26 26 79,539 0 101,91 2438500000 111 F1SHD7;A0A287ARY3;A0A5G2RBR3;A0A287BLV7 F1SHD7;A0A287ARY3;A0A5G2RBR3;A0A287BLV7 359 tr|F1SHD7|F1SHD7_PIG NADH-ubiquinone oxidoreductase 75 kDa subunit, mitochondrial OS=Sus scrofa OX=9823 GN=NDUFS1 PE=1 SV=4;tr|A0A287ARY3|A0A287ARY3_PIG NADH-ubiquinone oxidoreductase 75 kDa subunit, mitochondrial OS=Sus scrofa OX=9823 GN=NDUFS1 PE=1 SV=1;

32990000 0 34719000 62013000 0 31253000 53440000 0 29201000 70997000 0 72884000 2 2 2 19,4 19,4 19,4 11,108 0 5,7337 640970000 20 A0A287AS93;F1SI50 A0A287AS93;F1SI50 360 tr|A0A287AS93|A0A287AS93_PIG Complex I-B12 OS=Sus scrofa OX=9823 GN=NDUFB3 PE=1 SV=1;tr|F1SI50|F1SI50_PIG Complex I-B12 OS=Sus scrofa OX=9823 GN=NDUFB3 PE=1 SV=2

0 0 0 0 0 0 0 0 0 0 0 0 1 1 1 4,8 4,8 4,8 29,48 0 4,6721 0 1 F1S530;A0A287ASD0;A0A5G2R941 F1S530;A0A287ASD0;A0A5G2R941 361 tr|F1S530|F1S530_PIG 60S ribosomal protein L5 OS=Sus scrofa OX=9823 GN=RPL5 PE=1 SV=2;tr|A0A287ASD0|A0A287ASD0_PIG 60S ribosomal protein L5 OS=Sus scrofa OX=9823 GN=RPL5 PE=1 SV=1;tr|A0A5G2R941|A0A5G2R941_PIG 60S ribosomal protein L5 OS=Sus scrofa OX=9823

0 0 0 0 0 0 0 0 0 0 0 0 1 1 1 1,3 1,3 1,3 113,3 0,0058939 1,5155 5114500 1 A0A287BTF2;A0A287ASM1;A0A287BNF6;I3LDM3;A0A287B2B4 A0A287BTF2;A0A287ASM1;A0A287BNF6;I3LDM3;A0A287B2B4 362 tr|A0A287BTF2|A0A287BTF2_PIG Importin 7 OS=Sus scrofa OX=9823 GN=IPO7 PE=1 SV=2;tr|A0A287ASM1|A0A287ASM1_PIG Importin 7 OS=Sus scrofa OX=9823 GN=IPO7 PE=1

SV=2;tr|A0A287BNF6|A0A287BNF6_PIG Importin 7 OS=Sus scrofa OX=9823 GN=IPO7 PE=1 SV=1;tr|I3LDM3|I3LDM3
0 0 0 0 0 0 0 0 0 0 0 0 3 3 3 12,4 12,4 12,4
29,132 0 5,3101 105290000 3 I3LJ87;A0A287ASU4;A0A5G2RA75;A0A5G2QQM9
I3LJ87;A0A287ASU4 363 tr|I3LJ87|I3LJ87_PIG 40S ribosomal protein S2 OS=Sus scrofa OX=9823 GN=RPS2 PE=1 SV=3;tr|A0A287ASU4|A0A287ASU4_PIG 40S ribosomal protein S2 OS=Sus scrofa OX=9823 GN=RPS2 PE=1 SV=1
0 0 0 0 0 6348900 0 0 0 0 0 14711000 2 2 2 5,4
5,4 5,4 48,335 0 2,9619 65360000 2 A0A287ASX8;F1S3H4;A0A5G2QCC0
A0A287ASX8;F1S3H4;A0A5G2QCC0 364 tr|A0A287ASX8|A0A287ASX8_PIG Ubiquinone biosynthesis monooxygenase COQ6, mitochondrial OS=Sus scrofa OX=9823 GN=COQ6 PE=1 SV=1;tr|F1S3H4|F1S3H4_PIG Ubiquinone biosynthesis monooxygenase COQ6, mitochondrial OS=Sus scrofa OX=9823 GN=COQ6 PE=1 SV=2;tr|A0A5G2Q
22474000 19989000 18496000 27260000 17967000 19071000 28430000 15030000 15655000 20965000
21350000 20855000 5 5 5 23,4 23,4 23,4 27,867 0 32,147
673020000 50 A0A287AT18;A0A287BRR7;F1SBA5 A0A287AT18;A0A287BRR7 365
tr|A0A287AT18|A0A287AT18_PIG Proteasome subunit alpha type OS=Sus scrofa OX=9823 GN=PSMA7 PE=1 SV=1;tr|A0A287BRR7|A0A287BRR7_PIG Proteasome subunit alpha type OS=Sus scrofa OX=9823 GN=PSMA7 PE=1 SV=2
0 0 0 0 0 0 0 0 0 0 0 0 9 3 2 24,8 12,1 8,3
46,976 0 9,7501 95366000 4 A0A287AT48;A0A5G2QRD3 A0A287AT48 366
tr|A0A287AT48|A0A287AT48_PIG SERPIN domain-containing protein OS=Sus scrofa OX=9823 GN=LOC100156325 PE=1 SV=1
0 0 0 0 0 0 0 0 0 0 0 0 4 4 4 9,9 9,9 9,9
69,51 0 18,86 52271000 12 A0A287AT83 A0A287AT83 367
tr|A0A287AT83|A0A287AT83_PIG Eukaryotic translation initiation factor 4B isoform 1 OS=Sus scrofa OX=9823 PE=4 SV=2
0 0 0 0 0 0 0 0 0 0 0 0 6 1 0 9,8 2 0
59,525 0,003055 1,7901 7305100 1 F1SGI7;A0A287ATD0;A0A287APM4
F1SGI7;A0A287ATD0;A0A287APM4 368 tr|F1SGI7|F1SGI7_PIG IF rod domain-containing protein OS=Sus scrofa OX=9823 GN=KRT75 PE=1 SV=4;tr|A0A287ATD0|A0A287ATD0_PIG IF rod domain-containing protein OS=Sus scrofa OX=9823 GN=KRT75 PE=1 SV=1;tr|A0A287APM4|A0A287APM4_PIG IF rod domain-containing prot
116960000 77736000 92545000 7016900 39173000 9264800 4703100 11548000 14894000 3570600
17476000 88477000 5 4 4 29,7 24,6 24,6 20,129 0 12,953
1690600000 53 Q7M2W6;A0A5S6I1M3;A0A287ATJ4 Q7M2W6;A0A5S6I1M3;A0A287ATJ4
369 sp|Q7M2W6|CRYAB_PIG Alpha-crystallin B chain OS=Sus scrofa OX=9823 GN=CRYAB PE=2 SV=1;tr|A0A5S6I1M3|A0A5S6I1M3_PIG Alpha(B)-crystallin OS=Sus scrofa OX=9823 GN=CRYAB PE=1 SV=1;tr|A0A287ATJ4|A0A287ATJ4_PIG Alpha(B)-crystallin OS=Sus scrofa OX=9823 GN=CRYAB
29382000 28760000 32421000 64005000 55388000 45399000 57375000 40360000 39206000 57870000
56850000 55650000 14 14 14 34,2 34,2 34,2 50,213 0 75,541

3387400000 129 A0A287ATN8A0A287ATN8370 tr|A0A287ATN8|A0A287ATN8_PIG 60 kDa chaperonin OS=Sus scrofa OX=9823 GN=HSPD1 PE=1 SV=1
58936000 33165000 38173000 64566000 36831000 46659000 78228000 24548000 34442000 68128000
33870000 49126000 4 4 0 19,8 19,8 0 21,379 0 9,0166
1476600000 42
A0A287ATV4;A0A5G2QQK3;A0A5G2R6S0;A0A5G2R1K1;I3LKU0;F1RSX1;A0A287B3L0;I3LRE4;A0A5G2QGK4;K9IWC9;A0A5G2R664;A0A5S6GXN2;A0A286ZSH1;F2Z5W2;Q06AT6;A0A5G2R5V4;A0A287B510 A0A287ATV4;A0A5G2QQK3;A0A5G2R6S0;A0A5G2R1K1;I3LKU0 371
tr|A0A287ATV4|A0A287ATV4_PIG Rac family small GTPase 3 OS=Sus scrofa OX=9823 GN=RAC3 PE=4 SV=1;tr|A0A5G2QQK3|A0A5G2QQK3_PIG Rac family small GTPase 1 OS=Sus scrofa OX=9823 GN=RAC1 PE=1 SV=1;tr|A0A5G2R6S0|A0A5G2R6S0_PIG Rac family small GTPase 2 OS=Sus scro
0 6715800 0 7151000 0 0 0 0 7814200 7300500 0 0 2 2
2 17,2 17,2 17,2 17,447 0 3,0483 93978000 7 A0A287AUE4 A0A287AUE4 373
tr|A0A287AUE4|A0A287AUE4_PIG E2 ubiquitin-conjugating enzyme OS=Sus scrofa OX=9823 GN=UBE2K PE=1 SV=2
56884000 8704800 16605000 25331000 30056000 17416000 33340000 42240000 21803000 21344000
40865000 26515000 13 13 12 13 13 12,3 116,65 0 54,117
1100100000 75 F1S156;A0A5G2QAN9;A0A5G2QHE6;A0A287AUH9
F1S156;A0A5G2QAN9;A0A5G2QHE6;A0A287AUH9 374 tr|F1S156|F1S156_PIG Synaptopodin 2 OS=Sus scrofa OX=9823 GN=SYNPO2 PE=1 SV=4;tr|A0A5G2QAN9|A0A5G2QAN9_PIG Synaptopodin 2 OS=Sus scrofa OX=9823 GN=SYNPO2 PE=1 SV=1;tr|A0A5G2QHE6|A0A5G2QHE6_PIG Synaptopodin 2 OS=Sus scrofa OX=9823 GN=SYNPO2 PE=1 SV=1;tr|A0A
0 0 0 0 0 0 0 0 0 0 0 0 1 1 1 11,2 11,2 11,2
12,667 0,0078508 1,501 5863900 3 A0A5G2QSG0;A0A287AUN4
A0A5G2QSG0;A0A287AUN4 375 tr|A0A5G2QSG0|A0A5G2QSG0_PIG Vesicle associated membrane protein 5 OS=Sus scrofa OX=9823 GN=VAMP5 PE=1 SV=1;tr|A0A287AUN4|A0A287AUN4_PIG Vesicle associated membrane protein 5 OS=Sus scrofa OX=9823 GN=VAMP5 PE=1 SV=2
5809200 0 4742100 14418000 0 0 9044800 0 6284300 9961700 0 4134800
8 8 8 17,8 17,8 17,8 53,702 0 7,4569 167310000 17
A0A5G2QY06;F1S4D7;A0A287AUT0;K7GLP8;A0A5G2QMY2;A0A287BSF6
A0A5G2QY06;F1S4D7;A0A287AUT0;K7GLP8;A0A5G2QMY2;A0A287BSF6 376
tr|A0A5G2QY06|A0A5G2QY06_PIG GB1/RHD3-type G domain-containing protein OS=Sus scrofa OX=9823 GN=LOC100523668 PE=1 SV=1;tr|F1S4D7|F1S4D7_PIG GB1/RHD3-type G domain-containing protein OS=Sus scrofa OX=9823 GN=LOC100523668 PE=1 SV=3;tr|A0A287AUT0|A0A287AUT0_P
0 0 0 0 0 0 0 0 0 0 0 0 6 6 6 20,6 20,6 20,6
37,527 0 9,2555 175830000 8 A0A287AUZ3;F1SMX5 A0A287AUZ3;F1SMX5 377
tr|A0A287AUZ3|A0A287AUZ3_PIG Calcium binding protein 39 OS=Sus scrofa OX=9823 GN=CAB39 PE=1 SV=1;tr|F1SMX5|F1SMX5_PIG Calcium-binding protein 39 OS=Sus scrofa OX=9823 GN=CAB39 PE=1 SV=1

1222600000 654080000 681950000 1202400000 701680000 823790000 1069100000
803640000 923890000 1346800000 974420000 487300000 10 10
10 20,2 20,2 20,2 45,161 0 132,2 36055000000 658 A0A287AV34;F1RJW7
A0A287AV34;F1RJW7 378 tr|A0A287AV34|A0A287AV34_PIG Calsequestrin OS=Sus scrofa OX=9823 GN=CASQ1 PE=1 SV=1;tr|F1RJW7|F1RJW7_PIG Calsequestrin OS=Sus scrofa OX=9823 GN=CASQ1 PE=1 SV=2
123330000 102580000 89756000 21008000 74783000 38380000 38907000 63690000 28361000
24336000 66326000 51550000 13 13 13 58,7 58,7 58,7 164,47 0 45,634
1772700000 79 F1S7K4;A0A287AV63;A0A287BNJ5 F1S7K4;A0A287AV63;A0A287BNJ5
379 tr|F1S7K4|F1S7K4_PIG Perilipin 4 OS=Sus scrofa OX=9823 GN=PLIN4 PE=1 SV=4;tr|A0A287AV63|A0A287AV63_PIG Perilipin 4 OS=Sus scrofa OX=9823 GN=PLIN4 PE=1 SV=2;tr|A0A287BNJ5|A0A287BNJ5_PIG Perilipin 4 OS=Sus scrofa OX=9823 GN=PLIN4 PE=1 SV=2
6045000 6338900 0 0 0 0 0 0 0 0 0 0 4 4 4 11,4
11,4 11,4 47,362 0 9,1287 47266000 7 F1RKW8;A0A287AV98;A0A5G2QZX3
F1RKW8;A0A287AV98;A0A5G2QZX3 380 tr|F1RKW8|F1RKW8_PIG Proteasome 26S subunit, non-ATPase 11 OS=Sus scrofa OX=9823 GN=PSMD11 PE=1 SV=3;tr|A0A287AV98|A0A287AV98_PIG Proteasome 26S subunit, non-ATPase 11 OS=Sus scrofa OX=9823 GN=PSMD11 PE=1 SV=1;tr|A0A5G2QZX3|A0A5G2QZX3_PIG Proteasome 26S su
0 16487000 14269000 18189000 14838000 17223000 19277000 16978000 21320000 17454000 13611000
14399000 6 6 6 31,5 31,5 31,5 22,842 0 11,314 380810000 20
A0A5G2R7J8;A0A5G2QMN2;A0A5G2QL91;F2Z5N0;A0A5G2QTK7;A0A287AVI5
A0A5G2R7J8;A0A5G2QMN2;A0A5G2QL91;F2Z5N0;A0A5G2QTK7;A0A287AVI5 381
tr|A0A5G2R7J8|A0A5G2R7J8_PIG Proteasome subunit alpha type OS=Sus scrofa OX=9823 GN=PRORP PE=1 SV=1;tr|A0A5G2QMN2|A0A5G2QMN2_PIG Uncharacterized protein OS=Sus scrofa OX=9823 GN=PRORP PE=1 SV=1;tr|A0A5G2QL91|A0A5G2QL91_PIG Uncharacterized protein OS=Sus sc
10286000 5571600 3672300 19808000 9711300 6770700 21427000 6377400 6309300 19962000
6451800 7565400 5 5 5 19,3 19,3 19,3 29,428 0 13,797
446070000 28 A0A5G2Q9Z0;O77591;A0A287AVN0;I3LTC6;A0A5G2QFA4
A0A5G2Q9Z0;O77591;A0A287AVN0;I3LTC6 382 tr|A0A5G2Q9Z0|A0A5G2Q9Z0_PIG Inositol-1-monophosphatase OS=Sus scrofa OX=9823 GN=IMPA1 PE=3 SV=1;sp|O77591|IMPA1_PIG Inositol monophosphatase 1 OS=Sus scrofa OX=9823 GN=IMPA1 PE=2 SV=1;tr|A0A287AVN0|A0A287AVN0_PIG Inositol-1-monophosphatase OS=Sus scrofa O
12520000 15056000 0 10169000 11030000 0 25264000 10668000 10039000 10528000 0 0
5 5 5 33,1 33,1 33,1 28,229 0 23,049 296210000 20
A0A287BG87;A0A287AVP4;A0A287BP50;F1S1X9
A0A287BG87;A0A287AVP4;A0A287BP50;F1S1X9 383 tr|A0A287BG87|A0A287BG87_PIG Thioredoxin like 1 OS=Sus scrofa OX=9823 GN=TXNL1 PE=1 SV=1;tr|A0A287AVP4|A0A287AVP4_PIG Thioredoxin like 1 OS=Sus scrofa OX=9823 GN=TXNL1 PE=1 SV=1;tr|A0A287BP50|A0A287BP50_PIG Thioredoxin like 1 OS=Sus scrofa OX=9823 GN=TXNL1
0 0 0 0 0 0 0 0 0 0 0 0 0 1 1 1 4,3 4,3 4,3
37,349 0,0049554 1,5848 2189600 1 A0A287AW94;A0A481CK36;A0A287BCD1

A0A287AW94;A0A481CK36;A0A287BCD1 384 tr|A0A287AW94|A0A287AW94_PIG Uncharacterized protein OS=Sus scrofa OX=9823 GN=ADRM1 PE=1 SV=2;tr|A0A481CK36|A0A481CK36_PIG Proteasomal ubiquitin receptor ADRM1 isoform 1 OS=Sus scrofa OX=9823 GN=ADRM1 PE=1 SV=1;tr|A0A287BCD1|A0A287BCD1_PIG Uncharacterized
6404100 2156400 13497000 15915000 7385800 11494000 10038000 3958000 8618300 17929000 9233000 17483000 7 7 7 25,1 25,1 25,1 43,166 0 48,005 610760000 51 A0A287BEK4;I3LRR4;A0A287AWH6 A0A287BEK4;I3LRR4;A0A287AWH6 385 tr|A0A287BEK4|A0A287BEK4_PIG Complex I-9kD OS=Sus scrofa OX=9823 GN=NDUFV3 PE=1 SV=1;tr|I3LRR4|I3LRR4_PIG Complex I-9kD OS=Sus scrofa OX=9823 GN=NDUFV3 PE=1 SV=3;tr|A0A287AWH6|A0A287AWH6_PIG Complex I-9kD OS=Sus scrofa OX=9823 GN=NDUFV3 PE=1 SV=2
0 0 12774000 0 7518700 11706000 0 0 0 0 0 15324000 5 5 5 11,4 11,4 11,4 47,05 0 6,4213 129590000 13 F1SAW8;A0A287AWJ1;O18934 F1SAW8;A0A287AWJ1 386 tr|F1SAW8|F1SAW8_PIG Calsequestrin OS=Sus scrofa OX=9823 GN=CASQ2 PE=1 SV=4;tr|A0A287AWJ1|A0A287AWJ1_PIG Calsequestrin OS=Sus scrofa OX=9823 GN=CASQ2 PE=1 SV=1
14458000 6599400 0 0 0 0 9591700 0 4445000 7117900 0 0 4 4 4 22,1 22,1 22,1 22,652 0 9,2335 136130000 15 A0A287AX77;A0A5G2Q9D0;A0A5G2RDG4 A0A287AX77;A0A5G2Q9D0;A0A5G2RDG4 387 tr|A0A287AX77|A0A287AX77_PIG Uncharacterized protein OS=Sus scrofa OX=9823 GN=PPP1R2 PE=1 SV=1;tr|A0A5G2Q9D0|A0A5G2Q9D0_PIG Uncharacterized protein OS=Sus scrofa OX=9823 GN=PPP1R2 PE=1 SV=1;tr|A0A5G2RDG4|A0A5G2RDG4_PIG Uncharacterized protein OS=Sus scrofa
12569000 5632000 9709700 24760000 11561000 7694600 18873000 7877600 13006000 29530000 10866000 10834000 4 4 4 24,5 24,5 24,5 17,665 0 12,936 423220000 22 I3LKD2;A0A287AXF7;A0A5G2QDB6 I3LKD2;A0A287AXF7;A0A5G2QDB6 388 tr|I3LKD2|I3LKD2_PIG Family with sequence similarity 162 member A OS=Sus scrofa OX=9823 GN=FAM162A PE=1 SV=1;tr|A0A287AXF7|A0A287AXF7_PIG Family with sequence similarity 162 member A OS=Sus scrofa OX=9823 GN=FAM162A PE=1 SV=1;tr|A0A5G2QDB6|A0A5G2QDB6_PIG F
25402000 24327000 20632000 8954400 15776000 11437000 15982000 6694700 8181200 12152000 8607000 18176000 9 6 5 32,9 22,2 18,4 26,683 0 64,305 488420000 38 A0A287AXR5;A0A480PLY3;A0A287A0Q6;F2Z558 A0A287AXR5;A0A480PLY3;A0A287A0Q6;F2Z558 390 tr|A0A287AXR5|A0A287AXR5_PIG Tyrosine 3-monooxygenase/tryptophan 5-monooxygenase activation protein zeta OS=Sus scrofa OX=9823 GN=YWHAZ PE=1 SV=1;tr|A0A480PLY3|A0A480PLY3_PIG 14-3-3 protein zeta/delta OS=Sus scrofa OX=9823 GN=YWHAZ PE=1 SV=1;tr|A0A287A0Q6|
38791000 64627000 58837000 35683000 55463000 68827000 40933000 39835000 69267000 40166000 53276000 44021000 3 3 3 18,7 18,7 18,7 30,658 0 57,279 1801100000 74 F1S5Z8;A0A287AXS3;A0A5G2R2H0 F1S5Z8;A0A287AXS3;A0A5G2R2H0 391 tr|F1S5Z8|F1S5Z8_PIG Synaptophysin like 2 OS=Sus scrofa OX=9823 GN=SYPL2 PE=1 SV=2;tr|A0A287AXS3|A0A287AXS3_PIG Synaptophysin like 2 OS=Sus scrofa OX=9823 GN=SYPL2

PE=1 SV=2;tr|A0A5G2R2H0|A0A5G2R2H0_PIG Synaptophysin like 2 OS=Sus scrofa OX=9823 GN=SYPL2 P
8001400 5943700 7687100 5848100 5432300 10636000 0 4811200 8709700 4335400 5487900
6321100 5 5 5 24,2 24,2 24,2 27,507 0 17,108 230660000 21
A0A287AY54;A0A5S6HR40;Q29214;A0A287BGL3;A0A5S6HGK5
A0A287AY54;A0A5S6HR40;Q29214;A0A287BGL3;A0A5S6HGK5 392
tr|A0A287AY54|A0A287AY54_PIG 60S acidic ribosomal protein P0 OS=Sus scrofa OX=9823 GN=RPLP0 PE=3 SV=2;tr|A0A5S6HR40|A0A5S6HR40_PIG 60S acidic ribosomal protein P0 OS=Sus scrofa OX=9823 GN=RPLP0 PE=3 SV=1;sp|Q29214|RLA0_PIG 60S acidic ribosomal protein P0 O
115050000 181630000 92258000 172790000 112560000 98009000 166680000
154910000 114990000 135690000 133400000 59484000 9 9 9
32,8 32,8 32,8 33,404 0 129,82 3554500000 145
A0A5G2RBY2;F1S4J7;A0A287AY74;A0A287BJI2;A0A286ZVB2;P00636
A0A5G2RBY2;F1S4J7;A0A287AY74 393 tr|A0A5G2RBY2|A0A5G2RBY2_PIG Fructose-bisphosphatase OS=Sus scrofa OX=9823 GN=FBP2 PE=1 SV=1;tr|F1S4J7|F1S4J7_PIG Fructose-bisphosphatase OS=Sus scrofa OX=9823 GN=FBP2 PE=1 SV=3;tr|A0A287AY74|A0A287AY74_PIG Fructose-bisphosphatase OS=Sus scrofa OX=9823 GN=
5836900 0 8361800 8630200 2407300 9553200 4925500 0 0 5041200 0 8984600
6 5 5 22,9 20,7 20,7 35,828 0 20,119 159650000 25
P08132;A0A287B583;A0A287AYJ2;F1SLC4 P08132;A0A287B583;A0A287AYJ2;F1SLC4 394
sp|P08132|ANXA4_PIG Annexin A4 OS=Sus scrofa OX=9823 GN=ANXA4 PE=1 SV=2;tr|A0A287B583|A0A287B583_PIG Annexin OS=Sus scrofa OX=9823 GN=ANXA4 PE=3 SV=2;tr|A0A287AYJ2|A0A287AYJ2_PIG Annexin OS=Sus scrofa OX=9823 GN=ANXA4 PE=3 SV=1;tr|F1SLC4|F1SLC4_PIG Annexin
0 0 0 0 0 0 0 0 0 0 0 0 77 1 1 29,6 0,4 0,4
332,94 0 2,8999 152600000 12 A0A287AYJ9 A0A287AYJ9 396
tr|A0A287AYJ9|A0A287AYJ9_PIG Nebulin OS=Sus scrofa OX=9823 GN=NEB PE=1 SV=1
50989000 46036000 22812000 77462000 25095000 35602000 83947000 47709000 44209000 122850000
25740000 35150000 5 5 5 35,5 35,5 35,5 19,673 0 41,663
1489600000 81
A0A5G2RH88;P80031;A0A287AZ91;A0A5G2QMB0;A0A287BQ81;A0A287AQ27;F1RVN0
A0A5G2RH88;P80031;A0A287AZ91;A0A5G2QMB0;A0A287BQ81 397
tr|A0A5G2RH88|A0A5G2RH88_PIG GST class-pi OS=Sus scrofa OX=9823 GN=LOC100739508 PE=1 SV=1;sp|P80031|GSTP1_PIG Glutathione S-transferase P OS=Sus scrofa OX=9823 GN=GSTP1 PE=1 SV=2;tr|A0A287AZ91|A0A287AZ91_PIG GST class-pi OS=Sus scrofa OX=9823 GN=LOC1007395
400440000 224670000 163460000 210300000 165000000 142880000 311940000
136910000 203680000 400340000 134950000 86197000 10 10 9
43,7 43,7 38,9 25,194 0 41,785 9827500000 190
A0A287AZ96;A0A287B9H3;A0A287BEH6 A0A287AZ96;A0A287B9H3;A0A287BEH6 398
tr|A0A287AZ96|A0A287AZ96_PIG PDZ and LIM domain 7 OS=Sus scrofa OX=9823 GN=PDLIM7 PE=1 SV=2;tr|A0A287B9H3|A0A287B9H3_PIG PDZ and LIM domain 7 OS=Sus scrofa

OX=9823 GN=PDLIM7 PE=1 SV=2;tr|A0A287BEH6|A0A287BEH6_PIG PDZ and LIM domain 7 OS=Sus scrofa OX=9823

0 0 0 0 0 0 0 0 0 0 0 0 1 1 1 3 3 3

44,303 0 13,359 0 2 A0A287AZA3A0A287AZA3399

tr|A0A287AZA3|A0A287AZA3_PIG COP9 signalosome subunit 3 OS=Sus scrofa OX=9823 GN=COPS3 PE=1 SV=2

602710000 480670000 279310000 543050000 255950000 444620000 738970000

431980000 327020000 659320000 339860000 358320000 5 5

5 36,7 36,7 36,7 14,728 0 49,466 6852700000 130

P63053;A0A287AZA7;A7U5U2;F1S911;P0CG68;A0A5G2QDJ2;A0A2C9F3G2

P63053;A0A287AZA7;A7U5U2;F1S911;P0CG68;A0A5G2QDJ2;A0A2C9F3G2 400

sp|P63053|RL40_PIG Ubiquitin-60S ribosomal protein L40 OS=Sus scrofa OX=9823 GN=UBA52 PE=1 SV=2;tr|A0A287AZA7|A0A287AZA7_PIG 40S ribosomal protein S27a OS=Sus scrofa OX=9823 GN=RPS27A PE=1 SV=2;tr|A7U5U2|A7U5U2_PIG Ubiquitin B OS=Sus scrofa OX=9823 GN=UBB

0 13143000 20704000 0 6348200 7484200 0 0 0 0 0 5531300 6

6 6 15,4 15,4 15,4 47,253 0 10,547 150890000 15

A0A287BSQ5;A0A287AZB0;F1S4D5 A0A287BSQ5;A0A287AZB0;F1S4D5 401

tr|A0A287BSQ5|A0A287BSQ5_PIG Cysteine-S-conjugate beta-lyase 2 OS=Sus scrofa OX=9823 GN=KYAT3 PE=1 SV=1;tr|A0A287AZB0|A0A287AZB0_PIG Cysteine-S-conjugate beta-lyase 2 OS=Sus scrofa OX=9823 GN=KYAT3 PE=1 SV=1;tr|F1S4D5|F1S4D5_PIG Cysteine-S-conjugate beta-l

5158700 4315100 5632600 0 4524800 0 0 0 0 0 0 5232500 2

2 2 8,3 8,3 8,3 31,66 0,0022099 2,3597 57336000 4

A0A5G2QSZ0;A0A5G2R5A3;A0A287AZD8;A0A5G2RKB0;A0A5G2QQ79

A0A5G2QSZ0;A0A5G2R5A3;A0A287AZD8;A0A5G2RKB0;A0A5G2QQ79 402

tr|A0A5G2QSZ0|A0A5G2QSZ0_PIG BCL2 like 13 OS=Sus scrofa OX=9823 GN=BCL2L13 PE=1 SV=1;tr|A0A5G2R5A3|A0A5G2R5A3_PIG BCL2 like 13 OS=Sus scrofa OX=9823 GN=BCL2L13 PE=1 SV=1;tr|A0A287AZD8|A0A287AZD8_PIG BCL2 like 13 OS=Sus scrofa OX=9823 GN=BCL2L13 PE=1 SV=2;t

80034000 69877000 85617000 188760000 106400000 92951000 140610000 60540000

108940000 178100000 103100000 156840000 7 7 7 22,7 22,7

22,7 35,442 0 25,981 2643800000 77 A0A287AZF9 A0A287AZF9 403

tr|A0A287AZF9|A0A287AZF9_PIG Cytochrome c1 OS=Sus scrofa OX=9823 GN=CYC1 PE=1 SV=1

0 0 0 0 0 0 0 0 0 0 0 0 1 1 1 5 5 5

27,045 0,0030864 1,8533 32700000 4

A0A5G2RBL1;A0A287AZM3;I3LUD5;A0A5G2QHB2

A0A5G2RBL1;A0A287AZM3;I3LUD5;A0A5G2QHB2 404

tr|A0A5G2RBL1|A0A5G2RBL1_PIG UBX domain protein 1 OS=Sus scrofa OX=9823 GN=UBXN1 PE=1 SV=1;tr|A0A287AZM3|A0A287AZM3_PIG UBX domain protein 1 OS=Sus scrofa OX=9823 GN=UBXN1 PE=1 SV=2;tr|I3LUD5|I3LUD5_PIG UBX domain protein 1 OS=Sus scrofa OX=9823 GN=UBXN1 P

12306000 14009000 11916000 13172000 15302000 14133000 12057000 13315000 8754200 11983000

13070000 13336000 7 7 7 23,8 23,8 23,8 38,801 0 16,265

456710000	45
A0A5G2QES7;F1S0C1;A0A287AZV4;A0A5G2QCU9;A0A5G2QGP7;A0A5G2QHM7;A0A287BPT2;A0A287A703;A0A5G2QDU0 A0A5G2QES7;F1S0C1;A0A287AZV4 405
tr|A0A5G2QES7|A0A5G2QES7_PIG S-(hydroxymethyl)glutathione dehydrogenase OS=Sus scrofa OX=9823 GN=ADH5 PE=1 SV=1;tr|F1S0C1|F1S0C1_PIG S-(hydroxymethyl)glutathione dehydrogenase OS=Sus scrofa OX=9823 GN=ADH5 PE=1 SV=2;tr|A0A287AZV4|A0A287AZV4_PIG S-(hydroxym
0 15272000 20051000 0 12194000 0 0 14915000 10695000 0 10621000 11272000
5 5 5 8 8 8 80,769 0 9,7681 209470000 13
P23687;A0A287AZX9;F1RYQ0;A0A5S6HMR6 P23687;A0A287AZX9;F1RYQ0;A0A5S6HMR6
406 sp|P23687|PPCE_PIG Prolyl endopeptidase OS=Sus scrofa OX=9823 GN=PREP PE=1 SV=1;tr|A0A287AZX9|A0A287AZX9_PIG Prolyl endopeptidase OS=Sus scrofa OX=9823 GN=PREP PE=3 SV=1;tr|F1RYQ0|F1RYQ0_PIG Prolyl endopeptidase OS=Sus scrofa OX=9823 GN=PREP PE=3 SV=3;tr|A
0 13022000 8101100 0 0 0 0 0 0 0 0 0 4 4 4 3,1
3,1 3,1 162,44 0 18,605 70971000 7
A0A5G2QK29;A0A5G2R4Z7;A0A5G2QSL4;A0A5G2QRH0;I3LIY3;A0A287BEQ2;A0A287B073;A0A5G2QW60
A0A5G2QK29;A0A5G2R4Z7;A0A5G2QSL4;A0A5G2QRH0;I3LIY3;A0A287BEQ2;A0A287B073;A0A5G2QW60 407 tr|A0A5G2QK29|A0A5G2QK29_PIG Voltage-dependent L-type calcium channel subunit alpha OS=Sus scrofa OX=9823 GN=CACNA1S PE=3 SV=1;tr|A0A5G2R4Z7|A0A5G2R4Z7_PIG Voltage-dependent L-type calcium channel subunit alpha OS=Sus scrofa OX=9823 GN=CACNA1S PE=3 SV=1;tr
0 0 16627000 0 8835400 10068000 0 0 8733500 0 7708200 24320000
4 4 4 5,4 5,4 5,4 84 0 12,935 208100000 17
I3LV35;A0A287B087;Q8HY46;A0A481BBQ2;A0A287AK71
I3LV35;A0A287B087;Q8HY46;A0A481BBQ2;A0A287AK71 408 tr|I3LV35|I3LV35_PIG Carnitine O-palmitoyltransferase OS=Sus scrofa OX=9823 GN=CPT1B PE=1 SV=2;tr|A0A287B087|A0A287B087_PIG Carnitine O-palmitoyltransferase OS=Sus scrofa OX=9823 GN=CPT1B PE=1 SV=1;sp|Q8HY46|CPT1B_PIG Carnitine O-palmitoyltransferase 1, mu
20165000 16847000 19119000 30524000 22499000 22922000 29920000 17696000 17606000 17677000
20219000 20438000 3 3 3 12,9 12,9 12,9 29,053 0 7,5376
518040000 18 A0A287B088;Q29384 A0A287B088 409
tr|A0A287B088|A0A287B088_PIG Proteasome subunit beta OS=Sus scrofa OX=9823 GN=PSMB4 PE=1 SV=1
8319100 0 5885600 17275000 0 9186000 22941000 0 0 0 0 0 3
3 3 7,7 7,7 7,7 37,338 0 4,9619 178750000 13 I3L5M4;A0A287B0I5
I3L5M4;A0A287B0I5 410 tr|I3L5M4|I3L5M4_PIG Carbonic anhydrase 14 OS=Sus scrofa OX=9823 GN=CA14 PE=1 SV=3;tr|A0A287B0I5|A0A287B0I5_PIG Carbonic anhydrase 14 OS=Sus scrofa OX=9823 GN=CA14 PE=1 SV=2
0 0 0 0 0 0 0 0 0 0 0 0 1 1 1 3,4 3,4 3,4
33,248 0 5,0234 52637000 4 A0A5K1UL95;F1RYV5;A0A287B0I7
A0A5K1UL95;F1RYV5;A0A287B0I7 411 tr|A0A5K1UL95|A0A5K1UL95_PIG Aldo_ket_red

domain-containing protein OS=Sus scrofa OX=9823 GN=AKR1C3 PE=1 SV=1;tr|F1RYV5|F1RYV5_PIG Aldo_ket_red domain-containing protein OS=Sus scrofa OX=9823 GN=AKR1C3 PE=1 SV=3;tr|A0A287B0I7|A0A287B0I7_PIG Aldo_ket_red do
0 0 0 0 0 0 0 0 0 0 0 0 1 1 1 9,4 9,4 9,4
22,302 0,004995 1,6306 2456900 1 A0A5G2QL69;A0A287B0N7
A0A5G2QL69;A0A287B0N7 412 tr|A0A5G2QL69|A0A5G2QL69_PIG CARD domain-containing protein OS=Sus scrofa OX=9823 GN=NOL3 PE=1 SV=1;tr|A0A287B0N7|A0A287B0N7_PIG CARD domain-containing protein OS=Sus scrofa OX=9823 GN=NOL3 PE=1 SV=1
0 0 0 7809500 0 0 7879200 0 0 0 0 3431000 3 3 3
12 12 12 23,318 0 5,1939 70397000 8 A0A287B1X5;A0A287B0P4;A0A5G2Q979
A0A287B1X5;A0A287B0P4 413 tr|A0A287B1X5|A0A287B1X5_PIG TPD52 like 2 OS=Sus scrofa OX=9823 GN=TPD52L2 PE=1 SV=2;tr|A0A287B0P4|A0A287B0P4_PIG TPD52 like 2 OS=Sus scrofa OX=9823 GN=TPD52L2 PE=1 SV=2
0 0 0 20206000 19811000 23979000 19466000 0 14637000 32592000 22018000 44214000
2 2 2 13,3 13,3 13,3 6,8176 0,0086705 1,4047 386230000 6
A0A287B105;A0A287BJ57 A0A287B105;A0A287BJ57 414
tr|A0A287B105|A0A287B105_PIG Uncharacterized protein OS=Sus scrofa OX=9823 GN=COX7B PE=1 SV=2;tr|A0A287BJ57|A0A287BJ57_PIG Cytochrome c oxidase subunit 7B, mitochondrial OS=Sus scrofa OX=9823 GN=COX7B PE=1 SV=1
30459000 21091000 21576000 39071000 24100000 28075000 47743000 12225000 22943000 49796000
16061000 26132000 8 8 7 33,7 33,7 28,8 27,037 0 47,276
1232300000 44 A0A287B186;A5GFS8;A0A2C9F396;A0A5G2Q7S1;A0A5G2QSD0
A0A287B186;A5GFS8;A0A2C9F396;A0A5G2Q7S1 415 tr|A0A287B186|A0A287B186_PIG Vesicle-associated membrane protein-associated protein B OS=Sus scrofa OX=9823 GN=VAPB PE=4 SV=1;sp|A5GFS8|VAPB_PIG Vesicle-associated membrane protein-associated protein B OS=Sus scrofa OX=9823 GN=VAPB PE=3 SV=1;tr|A0A2C9F396|
70665000 36204000 43059000 0 7861400 2436900 3814700 4548000 4638800 3153200 3055400
17226000 9 9 9 19,2 19,2 19,2 56,612 0 28,608 890330000 38
A0A287B1U8;F1SVD5;A0A287B1B5;A0A287ALA4
A0A287B1U8;F1SVD5;A0A287B1B5;A0A287ALA4 416 tr|A0A287B1U8|A0A287B1U8_PIG Histone-lysine N-methyltransferase SMYD1 isoform 1 OS=Sus scrofa OX=9823 GN=SMYD1 PE=1 SV=1;tr|F1SVD5|F1SVD5_PIG SET and MYND domain containing 1 OS=Sus scrofa OX=9823 GN=SMYD1 PE=1 SV=4;tr|A0A287B1B5|A0A287B1B5_PIG SET and MYN
4630800 0 9889000 0 10307000 0 0 0 3283900 2992300 9191900 10916000
3 3 3 7,7 7,7 7,7 47,26 0 9,461 139710000 13 F1SED0;A0A287B1V1
F1SED0;A0A287B1V1 417 tr|F1SED0|F1SED0_PIG Acyl-CoA dehydrogenase short/branched chain OS=Sus scrofa OX=9823 GN=ACADSB PE=1 SV=4;tr|A0A287B1V1|A0A287B1V1_PIG Acyl-CoA dehydrogenase short/branched chain OS=Sus scrofa OX=9823 GN=ACADSB PE=1 SV=2
14429000 31913000 21738000 0 7323100 17237000 0 11830000 12633000 0 5440200 18430000
6 6 6 9,2 9,2 9,2 76,485 0 17,229 342440000 26
A0A5G2QKR0;A0A287B216;A0A287B8F4;F1RIP1;A0A287A291;F1SR04;A0A287B5W1
A0A5G2QKR0;A0A287B216;A0A287B8F4;F1RIP1;A0A287A291 418

tr|A0A5G2QKR0|A0A5G2QKR0_PIG Glycogen [starch] synthase OS=Sus scrofa OX=9823 GN=GYS1 PE=1 SV=1;tr|A0A287B216|A0A287B216_PIG Glycogen [starch] synthase OS=Sus scrofa OX=9823 GN=GYS1 PE=1 SV=1;tr|A0A287B8F4|A0A287B8F4_PIG Glycogen [starch] synthase OS=Sus s
0 0 0 0 0 0 0 0 0 0 0 0 1 1 1 30,5 30,5 30,5
6,2952 0 5,4901 2011400 1 A0A287B262 A0A287B262 419
tr|A0A287B262|A0A287B262_PIG HMA domain-containing protein OS=Sus scrofa OX=9823 PE=1 SV=1
0 2562300 3125700 0 2276900 0 0 0 0 0 0 0 2 2 2
4 4 4 53,142 0,0022075 2,3575 16712000 3
F1S4Z9;A0A5G2Q9D9;A0A287B2G7 F1S4Z9;A0A5G2Q9D9;A0A287B2G7 420
tr|F1S4Z9|F1S4Z9_PIG Uncharacterized protein OS=Sus scrofa OX=9823 GN=CDK5RAP1 PE=1 SV=2;tr|A0A5G2Q9D9|A0A5G2Q9D9_PIG Uncharacterized protein OS=Sus scrofa OX=9823 GN=CDK5RAP1 PE=1 SV=1;tr|A0A287B2G7|A0A287B2G7_PIG Uncharacterized protein OS=Sus scrofa OX=
0 2555400 0 0 0 0 2766400 0 9420800 3256700 0 0 3 3
3 13,5 13,5 13,5 30,707 0 7,7041 43854000 8 A0A287B2M3;A0A5S8KR21;Q4GWZ2
A0A287B2M3;A0A5S8KR21;Q4GWZ2 421 tr|A0A287B2M3|A0A287B2M3_PIG 40S ribosomal protein SA OS=Sus scrofa OX=9823 GN=RPSA PE=1 SV=1;tr|A0A5S8KR21|A0A5S8KR21_PIG 40S ribosomal protein SA OS=Sus scrofa OX=9823 GN=RPSA PE=1 SV=1;sp|Q4GWZ2|RSSA_PIG 40S ribosomal protein SA OS=Sus scrofa OX=9823 GN
0 0 0 0 0 0 0 0 0 0 0 0 2 2 2 3,6 3,6 3,6
64,849 0,0078431 1,4975 42723000 3 A0A287B2W0;I3LNB4 A0A287B2W0;I3LNB4
422 tr|A0A287B2W0|A0A287B2W0_PIG Multifunctional fusion protein OS=Sus scrofa OX=9823 GN=ALDH4A1 PE=1 SV=1;tr|I3LNB4|I3LNB4_PIG Multifunctional fusion protein OS=Sus scrofa OX=9823 GN=ALDH4A1 PE=1 SV=3
0 0 0 0 0 0 0 0 0 0 0 0 3 3 3 3,4 3,4 3,4
100,39 0,0011377 2,618 8558200 4 Q29052;A0A287B3S3;F1SH96;CON__Q0VCM5
Q29052;A0A287B3S3 423 sp|Q29052|ITIH1_PIG Inter-alpha-trypsin inhibitor heavy chain H1 OS=Sus scrofa OX=9823 GN=ITIH1 PE=2 SV=1;tr|A0A287B3S3|A0A287B3S3_PIG Inter-alpha-trypsin inhibitor heavy chain H1 OS=Sus scrofa OX=9823 GN=ITIH1 PE=1 SV=2
0 0 5676900 0 0 0 0 0 0 5087200 0 14665000 4 4 4
16 16 16 34,373 0 5,8738 84346000 5
A0A287B462;A0A481BCG1;F1S9I9;P0C2C3;A0A5G2QEM1;F1S9I6;A0A287B6R1
A0A287B462;A0A481BCG1;F1S9I9;P0C2C3;A0A5G2QEM1 424
tr|A0A287B462|A0A287B462_PIG Mitochondrial amidoxime reducing component 2 OS=Sus scrofa OX=9823 GN=MTARC2 PE=1 SV=2;tr|A0A481BCG1|A0A481BCG1_PIG Mitochondrial amidoxime reducing component 2 OS=Sus scrofa OX=9823 GN=MTARC2 PE=1 SV=1;tr|F1S9I9|F1S9I9_PIG Mit
10731000 20030000 15572000 10698000 16970000 22032000 8498000 14680000 21032000 6417100
17409000 22079000 14 14 12 40,6 40,6 36,8 50,241 0 60,171
881890000 90 A0A287B4B7;A0A5G2QSK4;A0A287B6B3;F1RUK8;Q6Q7J2;A0A5G2RKF7

A0A287B4B7;A0A5G2QSK4;A0A287B6B3;F1RUK8;Q6Q7J2;A0A5G2RKF7 425

tr|A0A287B4B7|A0A287B4B7_PIG Rab GDP dissociation inhibitor OS=Sus scrofa OX=9823 GN=GDI2 PE=1 SV=2;tr|A0A5G2QSK4|A0A5G2QSK4_PIG Rab GDP dissociation inhibitor OS=Sus scrofa OX=9823 GN=GDI2 PE=1 SV=1;tr|A0A287B6B3|A0A287B6B3_PIG Rab GDP dissociation inhibi

7226600 14101000 14545000 9118000 7560600 5591500 8398200 15137000 12409000 10991000

9224000 7152800 2 2 2 15,7 15,7 15,7 19,595 0 6,0636

339820000 21 P61288;A0A5G2QHY7;A0A287B4E6;A0A287AMB0

P61288;A0A5G2QHY7;A0A287B4E6;A0A287AMB0 426 sp|P61288|TCTP_PIG Translationally-controlled tumor protein OS=Sus scrofa OX=9823 GN=TPT1 PE=2 SV=1;tr|A0A5G2QHY7|A0A5G2QHY7_PIG Translationally-controlled tumor protein OS=Sus scrofa OX=9823 GN=TPT1 PE=1 SV=1;tr|A0A287B4E6|A0A287B4E6_PIG Translationally-c

0 0 0 0 0 0 0 0 0 0 0 0 4 1 1 22,3 7,6 7,6

21,104 0,0021739 2,2228 57737000 4 A0A287B4G4;A0A5G2QVC6

A0A287B4G4;A0A5G2QVC6 427 tr|A0A287B4G4|A0A287B4G4_PIG Rac family small GTPase 1 OS=Sus scrofa OX=9823 GN=RAC1 PE=1 SV=2;tr|A0A5G2QVC6|A0A5G2QVC6_PIG Rac family small GTPase 1 OS=Sus scrofa OX=9823 GN=RAC1 PE=1 SV=1

8079400 9299700 7558600 9596900 8578100 9314400 6723200 7309600 8682300 9578800

8960400 10238000 7 7 7 16,7 16,7 16,7 47,694 0 8,9336

362070000 30 Q710C4;A0A287B4J2;A0A5G2R5W8;A0A5G2Q7X1;F1S4Y7

Q710C4;A0A287B4J2;A0A5G2R5W8;A0A5G2Q7X1;F1S4Y7 428 sp|Q710C4|SAHH_PIG Adenosylhomocysteinase OS=Sus scrofa OX=9823 GN=AHCY PE=3 SV=3;tr|A0A287B4J2|A0A287B4J2_PIG Adenosylhomocysteinase OS=Sus scrofa OX=9823 GN=AHCY PE=3 SV=1;tr|A0A5G2R5W8|A0A5G2R5W8_PIG Adenosylhomocysteinase OS=Sus scrofa OX=9823 GN=AHCY

6651300 5216100 7927700 6674100 5091700 9838600 7601800 0 7569400 6762200 6405200

6248400 3 3 3 13,4 13,4 13,4 26,425 0 5,51 174110000 12

A0A287B4K6;F2Z5C7;I3LN32 A0A287B4K6;F2Z5C7 429

tr|A0A287B4K6|A0A287B4K6_PIG 40S ribosomal protein S3a OS=Sus scrofa OX=9823 GN=RPS3A PE=1 SV=1;tr|F2Z5C7|F2Z5C7_PIG 40S ribosomal protein S3a OS=Sus scrofa OX=9823 GN=RPS3A PE=1 SV=1

0 5718200 4567200 4853500 0 4760600 4392300 0 5186300 0 4180100 4732600

4 2 2 10,4 6,4 6,4 48,153 0 4,1004 82741000 3

I3L893;A0A287B799;A0A287B4U1;A0A287BPJ1;A0A287BJD1

I3L893;A0A287B799;A0A287B4U1;A0A287BPJ1;A0A287BJD1 430 tr|I3L893|I3L893_PIG Rab GDP dissociation inhibitor OS=Sus scrofa OX=9823 GN=GDI1 PE=1 SV=2;tr|A0A287B799|A0A287B799_PIG Rab GDP dissociation inhibitor OS=Sus scrofa OX=9823 GN=GDI1 PE=1 SV=1;tr|A0A287B4U1|A0A287B4U1_PIG Rab GDP dissociation inhibitor OS=S

0 28551000 38632000 0 23758000 25003000 0 16082000 20580000 0 0 79357000

3 3 3 11,5 11,5 11,5 33,941 0 27,398 407530000 14

A0A287B574;A0A5G2R4W7;F1SNZ6;O19069;A0A480U8Y2;A0A287BA50;A0A5G2QKE5;F1SNZ7

A0A287B574;A0A5G2R4W7;F1SNZ6;O19069;A0A480U8Y2;A0A287BA50;A0A5G2QKE5;F1SN

Z7 431 tr|A0A287B574|A0A287B574_PIG Succinate--CoA ligase [ADP/GDP-forming] subunit alpha, mitochondrial OS=Sus scrofa OX=9823 GN=SUCLG1 PE=3 SV=2;tr|A0A5G2R4W7|A0A5G2R4W7_PIG Succinate--CoA ligase [ADP/GDP-forming] subunit alpha, mitochondrial OS=Sus scrofa OX=9

0 0 0 0 0 0 0 0 0 0 0 0 1 1 1 5,9 5,9 5,9 19,509 0,0031712 2,0249 5509800 3 F1RGQ5;A0A287B5D6 F1RGQ5;A0A287B5D6 432 tr|F1RGQ5|F1RGQ5_PIG Ankyrin repeat and FYVE domain containing 1 OS=Sus scrofa OX=9823 GN=ANKFY1 PE=3 SV=3;tr|A0A287B5D6|A0A287B5D6_PIG Ankyrin repeat and FYVE domain containing 1 OS=Sus scrofa OX=9823 GN=ANKFY1 PE=1 SV=2

115430000 36857000 103680000 37655000 68874000 100260000 76984000 55655000 57857000 84358000 71119000 101400000 192 192 47 33,3 33,3 8 772,72 0 323,31 24067000000 1141 F1SHX0;A0A287B5G8;A0A287ARH5;A0A286ZNE6;REV__A0A5G2R3Y3;REV__A0A287BG82;REV__I3LSJ0;REV__F1SM42;REV__A0A287A5J3;A0A287B9E8;F1RVG6;A0A287BFR4;A0A5G2QZ92;A0A287AHR6 F1SHX0;A0A287B5G8 433 tr|F1SHX0|F1SHX0_PIG Nebulin OS=Sus scrofa OX=9823 GN=NEB PE=1 SV=4;tr|A0A287B5G8|A0A287B5G8_PIG Nebulin OS=Sus scrofa OX=9823 GN=NEB PE=1 SV=2

6867900 3153600 3709300 0 2742600 0 0 0 0 3948000 0 0 3 3 3 2,6 2,6 2,6 130,46 0 3,4947 63128000 8 A0A287B7U5;A0A287B5N9;A0A287BD91;F1REW5 A0A287B7U5;A0A287B5N9;A0A287BD91;F1REW5 434 tr|A0A287B7U5|A0A287B7U5_PIG CAP-Gly domain containing linker protein 1 OS=Sus scrofa OX=9823 GN=CLIP1 PE=1 SV=2;tr|A0A287B5N9|A0A287B5N9_PIG CAP-Gly domain containing linker protein 1 OS=Sus scrofa OX=9823 GN=CLIP1 PE=1 SV=1;tr|A0A287BD91|A0A287BD91_PIG C

0 0 0 0 0 0 0 50210000 56848000 0 0 0 4 1 1 4,9 1,6 1,6 97,697 0 3,9619 185640000 2 A0A5G2QA41;A0A287B5Q4;A0A287AIT2;A0A5G2R927;A0A286ZT48;F1SQK1 A0A5G2QA41;A0A287B5Q4;A0A287AIT2;A0A5G2R927;A0A286ZT48;F1SQK1 435 tr|A0A5G2QA41|A0A5G2QA41_PIG Reticulon OS=Sus scrofa OX=9823 GN=RTN4 PE=1 SV=1;tr|A0A287B5Q4|A0A287B5Q4_PIG Reticulon OS=Sus scrofa OX=9823 GN=RTN4 PE=1 SV=2;tr|A0A287AIT2|A0A287AIT2_PIG Reticulon OS=Sus scrofa OX=9823 GN=RTN4 PE=1 SV=2;tr|A0A5G2R927|A0A5G

0 0 0 0 0 0 13349000 0 0 9708900 0 0 2 2 2 19 19 19 10,987 0,0011416 2,6732 104800000 11 A0A287B5X4 A0A287B5X4 437 tr|A0A287B5X4|A0A287B5X4_PIG Uncharacterized protein OS=Sus scrofa OX=9823 PE=3 SV=1

6862000 0 0 0 0 9766700 9760800 0 0 6283600 0 6707100 4 4 4 9,5 9,5 9,5 44,17 0 7,5367 122210000 14 A0A287B626 A0A287B626 438 tr|A0A287B626|A0A287B626_PIG Uncharacterized protein OS=Sus scrofa OX=9823 PE=1 SV=2

0 0 0 0 0 0 0 0 0 0 0 0 1 1 1 2,5 2,5 2,5 59,413 0 5,1685 3014200000 72 A0A287B699 A0A287B699 439 tr|A0A287B699|A0A287B699_PIG Ankyrin repeat and SOCS box containing 16 OS=Sus scrofa OX=9823 GN=ASB16 PE=4 SV=1

14253000 0 0 0 0 0 14913000 0 0 0 0 0 3 3 3 5,8
5,8 5,8 58,328 0 10,36 170480000 7
F1SKI5;A0A287BMK8;A0A287B6A4;A0A5G2QZ62;A0A5G2Q9V4;F1SML7
F1SKI5;A0A287BMK8;A0A287B6A4 440 tr|F1SKI5|F1SKI5_PIG Inosine-5-monophosphate dehydrogenase OS=Sus scrofa OX=9823 GN=QRICH1 PE=1 SV=3;tr|A0A287BMK8|A0A287BMK8_PIG Inosine-5-monophosphate dehydrogenase OS=Sus scrofa OX=9823 GN=QRICH1 PE=1 SV=2;tr|A0A287B6A4|A0A287B6A4_PIG Inosine-5-mono
13687000 8442300 22168000 2292100 16679000 19717000 0 7856100 10648000 8035700 16630000
37439000 10 10 10 18,8 18,8 18,8 62,921 0 35,141 850330000 71
A0A287B6E3;A0A5G2QM65;A0A287BPI1;P55931;A0A286ZXI8;F1RW89;A0A287A4K6
A0A287B6E3;A0A5G2QM65;A0A287BPI1;P55931;A0A286ZXI8;F1RW89;A0A287A4K6 441
tr|A0A287B6E3|A0A287B6E3_PIG Electron transfer flavoprotein-ubiquinone oxidoreductase OS=Sus scrofa OX=9823 GN=ETFDH PE=1 SV=1;tr|A0A5G2QM65|A0A5G2QM65_PIG Electron transfer flavoprotein-ubiquinone oxidoreductase OS=Sus scrofa OX=9823 GN=ETFDH PE=1 SV=1;tr
0 6044400 6498800 0 5686500 7899400 0 0 4574700 0 4312400 5876900
5 5 5 6,9 6,9 6,9 90,943 0 9,4342 132980000 20
F1RI15;A0A287B6J6;A0A5G2R0T1;A0A5G2RBW3
F1RI15;A0A287B6J6;A0A5G2R0T1;A0A5G2RBW3 442 tr|F1RI15|F1RI15_PIG Heat shock 70 kDa protein 4 OS=Sus scrofa OX=9823 GN=HSPA4 PE=1 SV=3;tr|A0A287B6J6|A0A287B6J6_PIG Heat shock 70 kDa protein 4 OS=Sus scrofa OX=9823 GN=HSPA4 PE=1 SV=1;tr|A0A5G2R0T1|A0A5G2R0T1_PIG Uncharacterized protein OS=Sus scrofa O
4000800 6736700 0 3614300 11489000 5824500 0 11721000 0 3403000 9846700 4299400
4 4 4 13,8 13,8 13,8 34,149 0 11,065 149290000 22
A0A287B6M0;F1RXC2;A0A5G2QRG3 A0A287B6M0;F1RXC2;A0A5G2QRG3 443
tr|A0A287B6M0|A0A287B6M0_PIG Carbonic anhydrase OS=Sus scrofa OX=9823 GN=CA2 PE=1 SV=2;tr|F1RXC2|F1RXC2_PIG Carbonic anhydrase OS=Sus scrofa OX=9823 GN=CA2 PE=1 SV=2;tr|A0A5G2QRG3|A0A5G2QRG3_PIG Carbonic anhydrase 2 OS=Sus scrofa OX=9823 GN=CA2 PE=1 SV=1
0 7329700 7334300 39603000 0 6267300 22519000 16300000 29113000 32415000 7252200
6098200 17 1 1 44,9 3,2 3,2 47,432 0 3,1952 233430000 22
A0A287B6S5 A0A287B6S5 444 tr|A0A287B6S5|A0A287B6S5_PIG 2-phospho-D-glycerate hydro-lyase OS=Sus scrofa OX=9823 GN=ENO1 PE=1 SV=1
0 7083800 0 15586000 0 0 10248000 9332900 0 18906000 0 0 2
2 2 7,7 7,7 7,7 44,765 0 5,932 142440000 5
I3LD55;A0A287B6T3;F1RXY4 I3LD55;A0A287B6T3;F1RXY4 445 tr|I3LD55|I3LD55_PIG Ubiquinone biosynthesis O-methyltransferase, mitochondrial OS=Sus scrofa OX=9823 GN=COQ3 PE=1 SV=2;tr|A0A287B6T3|A0A287B6T3_PIG Ubiquinone biosynthesis O-methyltransferase, mitochondrial OS=Sus scrofa OX=9823 GN=FAXC PE=1 SV=1;tr|F1RXY
0 0 0 0 0 0 0 0 0 0 0 0 2 2 2 20,9 20,9 20,9
12,784 0 6,0798 40868000 4 A0A287B6Y1;F2Z554 A0A287B6Y1;F2Z554 446
tr|A0A287B6Y1|A0A287B6Y1_PIG 60S ribosomal protein L30 OS=Sus scrofa OX=9823 GN=RPL30 PE=1 SV=1;tr|F2Z554|F2Z554_PIG 60S ribosomal protein L30 OS=Sus scrofa OX=9823 GN=RPL30 PE=1 SV=2

11198000 0 10921000 15356000 0 9118500 24161000 0 6360100 29319000 0 9412900
2 2 2 18 18 18 12,903 0 11,068 288720000 12
A0A5G2QVI0;Q07717;A0A5S6FYV9;A0A287B7B9;A0A287BEM9;A0A5S6I1G3
A0A5G2QVI0;Q07717;A0A5S6FYV9;A0A287B7B9;A0A287BEM9;A0A5S6I1G3 447
tr|A0A5G2QVI0|A0A5G2QVI0_PIG Beta-2-microglobulin OS=Sus scrofa OX=9823 GN=B2M PE=1 SV=1;sp|Q07717|B2MG_PIG Beta-2-microglobulin OS=Sus scrofa OX=9823 GN=B2M PE=1 SV=1;tr|A0A5S6FYV9|A0A5S6FYV9_PIG Beta-2-microglobulin OS=Sus scrofa OX=9823 GN=B2M PE=1 SV=1
0 0 0 0 0 0 0 0 0 0 0 0 2 2 2 2,8 2,8 2,8
76,244 0 3,5838 25589000 2
I3LH62;A0A5G2QCD0;A0A287B7I4;F1SGB0;K7GM30;A0A5G2R7C3;K7GNF4;I3LAK5;A0A5G2QTK9;F1RQ52;A0A287BTE5;A0A286ZS04;K7GQ00;A0A287BD79;K7GSF4;A0A5G2QEG2;F1ST39;A0A5G2QDJ5
I3LH62;A0A5G2QCD0;A0A287B7I4;F1SGB0;K7GM30;A0A5G2R7C3;K7GNF4;I3LAK5;A0A5G2QTK9;F1RQ52;A0A287BTE5;A0A286ZS04;K7GQ00;A0A287BD79;K7GSF4;A0A5G2QEG2;F1ST39;A0A5G2QDJ5 448 tr|I3LH62|I3LH62_PIG Fragile X mental retardation syndrome-related protein 1 isoform X1 OS=Sus scrofa OX=9823 GN=FXR1 PE=1 SV=1;tr|A0A5G2QCD0|A0A5G2QCD0_PIG Agenet-like domain-containing protein OS=Sus scrofa OX=9823 GN=FXR1 PE=1 SV=1;tr|A0A287B7I4|A0A287B
0 0 0 0 0 0 0 0 0 0 0 0 2 2 2 8,7 8,7 8,7
27,658 0 3,7353 15010000 3 A0A287B7J0;A0A287BD01;I3LIL5
A0A287B7J0;A0A287BD01;I3LIL5 449 tr|A0A287B7J0|A0A287B7J0_PIG Hydroxymethylglutaryl-CoA lyase OS=Sus scrofa OX=9823 GN=HMGCL PE=1 SV=1;tr|A0A287BD01|A0A287BD01_PIG Hydroxymethylglutaryl-CoA lyase OS=Sus scrofa OX=9823 GN=HMGCL PE=1 SV=1;tr|I3LIL5|I3LIL5_PIG Hydroxymethylglutaryl-CoA lyase
0 0 0 0 0 0 0 0 0 0 0 0 2 2 2 5,7 5,7 5,7
39,578 0 2,9284 122130000 12
I3L973;A0A287B7K9;A0A5G2QJH9;A0A5G2R257;A0A5G2QZ66;A0A5G2QCR7;A0A5G2R583;F1SJU4
I3L973;A0A287B7K9;A0A5G2QJH9;A0A5G2R257;A0A5G2QZ66;A0A5G2QCR7;A0A5G2R583;F1SJU4 450 tr|I3L973|I3L973_PIG Parvin alpha OS=Sus scrofa OX=9823 GN=PARVA PE=1 SV=3;tr|A0A287B7K9|A0A287B7K9_PIG Parvin alpha OS=Sus scrofa OX=9823 GN=PARVA PE=1 SV=1;tr|A0A5G2QJH9|A0A5G2QJH9_PIG Parvin alpha OS=Sus scrofa OX=9823 GN=PARVA PE=1 SV=1;tr|A0A5G2R257|A
0 0 0 0 0 0 0 0 0 0 0 0 1 1 1 2,4 2,4 2,4
63,661 0,0030211 1,7228 311120000 8 A0A287B7Q4 A0A287B7Q4 451
tr|A0A287B7Q4|A0A287B7Q4_PIG Family with sequence similarity 151 member A OS=Sus scrofa OX=9823 GN=FAM151A PE=3 SV=1
11930000 9590400 7747600 40880000 0 12246000 20465000 10303000 18727000 31558000 11472000
11725000 4 4 4 53 53 53 11,693 0 14,682 443480000 26
A0A287B7U0;F1RYZ0;Q29315;F1RNZ2 A0A287B7U0;F1RYZ0 452
tr|A0A287B7U0|A0A287B7U0_PIG 60S acidic ribosomal protein P2 OS=Sus scrofa OX=9823

GN=RPLP2 PE=1 SV=2;tr|F1RYZ0|F1RYZ0_PIG 60S acidic ribosomal protein P2 OS=Sus scrofa OX=9823 GN=RPLP2 PE=1 SV=2
9461400 6012900 6397300 0 3619600 0 0 0 0 0 0 0 3 3
3 7,7 7,7 7,7 39,751 0 4,0894 57194000 9
A0A5G2R0W9;A0A5G2QRG6;F1RGC9;A0A287B7X9;A0A5G2QHX0;A0A5G2R6X9
A0A5G2R0W9;A0A5G2QRG6;F1RGC9;A0A287B7X9 453
tr|A0A5G2R0W9|A0A5G2R0W9_PIG 26S proteasome non-ATPase regulatory subunit 13 OS=Sus scrofa OX=9823 GN=PSMD13 PE=1 SV=1;tr|A0A5G2QRG6|A0A5G2QRG6_PIG 26S proteasome non-ATPase regulatory subunit 13 OS=Sus scrofa OX=9823 GN=PSMD13 PE=1 SV=1;tr|F1RGC9|F1RGC9_P
38196000 20898000 19856000 62378000 24246000 24314000 64954000 18860000 21119000 47551000
20228000 21791000 8 8 8 25,1 25,1 25,1 36,864 0 51,697
2136400000 92 A0A5G2QYZ3;A0A287B8B1;F1SRB9;A0A5G2R2Q0
A0A5G2QYZ3;A0A287B8B1;F1SRB9;A0A5G2R2Q0 454 tr|A0A5G2QYZ3|A0A5G2QYZ3_PIG STI1 domain-containing protein OS=Sus scrofa OX=9823 GN=ST13 PE=1 SV=1;tr|A0A287B8B1|A0A287B8B1_PIG STI1 domain-containing protein OS=Sus scrofa OX=9823 GN=ST13 PE=1 SV=1;tr|F1SRB9|F1SRB9_PIG STI1 domain-containing protein OS=S
27133000 30089000 35191000 27857000 33223000 42155000 21608000 25791000 18930000 27633000
37875000 48349000 6 6 6 19,2 19,2 19,2 38,318 0 16,675
1177600000 38
A0A5G2QBD8;A0A2C9F3H3;A0A5G2RIA6;A0A287B8H8;P37111;A0A287BCN4;A0A5G2RAG0;I3LAP5;A0A286ZX40;A0A5S6IH59
A0A5G2QBD8;A0A2C9F3H3;A0A5G2RIA6;A0A287B8H8;P37111;A0A287BCN4;A0A5G2RAG0;I3LAP5;A0A286ZX40;A0A5S6IH59 455 tr|A0A5G2QBD8|A0A5G2QBD8_PIG N-acyl-L-amino-acid amidohydrolase OS=Sus scrofa OX=9823 GN=ACY1 PE=3 SV=1;tr|A0A2C9F3H3|A0A2C9F3H3_PIG N-acyl-L-amino-acid amidohydrolase OS=Sus scrofa OX=9823 GN=ACY1 PE=3 SV=1;tr|A0A5G2RIA6|A0A5G2RIA6_PIG N-acyl-L-amino-acid
0 0 0 0 0 0 0 0 0 0 0 0 1 1 1 12,5 12,5 12,5
12,455 0 3,1265 17666000 3 A0A287B950;F2Z545;A0A5G2QXY8
A0A287B950;F2Z545;A0A5G2QXY8 456 tr|A0A287B950|A0A287B950_PIG Elongin-C OS=Sus scrofa OX=9823 GN=ELOC PE=1 SV=1;tr|F2Z545|F2Z545_PIG Elongin-C OS=Sus scrofa OX=9823 GN=ELOC PE=1 SV=2;tr|A0A5G2QXY8|A0A5G2QXY8_PIG Elongin-C OS=Sus scrofa OX=9823 GN=ELOC PE=1 SV=1
0 0 0 0 0 11753000 8085600 0 0 8596700 0 0 3 3 3
15,6 15,6 15,6 18,412 0 6,9866 106420000 7 A0A287B975;A0A287AIM5
A0A287B975 457 tr|A0A287B975|A0A287B975_PIG CCHC-type zinc finger nucleic acid binding protein OS=Sus scrofa OX=9823 GN=CNBP PE=1 SV=2
0 0 0 0 0 0 0 0 0 0 0 0 2 2 2 4,3 4,3 4,3
57,043 0 3,1141 16987000 4 A0A287B9A6;F1S596 A0A287B9A6;F1S596 458
tr|A0A287B9A6|A0A287B9A6_PIG Glucosidase 2 subunit beta OS=Sus scrofa OX=9823 GN=PRKCSH PE=1 SV=1;tr|F1S596|F1S596_PIG Glucosidase 2 subunit beta OS=Sus scrofa OX=9823 GN=PRKCSH PE=1 SV=1

0 0 0 0 13972000 0 0 10839000 0 9436400 0 24892000 3 3 3 16,6 16,6 16,6 20,741 0 30,524 184070000 15 A0A5G2QT22;I3LK43;A0A287B9B0;A0A5G2RCS5;A0A5G2R526 A0A5G2QT22;I3LK43;A0A287B9B0;A0A5G2RCS5;A0A5G2R526 459 tr|A0A5G2QT22|A0A5G2QT22_PIG Complex I-20kD OS=Sus scrofa OX=9823 GN=NDUFS7 PE=1 SV=1;tr|I3LK43|I3LK43_PIG Complex I-20kD OS=Sus scrofa OX=9823 GN=NDUFS7 PE=1 SV=2;tr|A0A287B9B0|A0A287B9B0_PIG Complex I-20kD OS=Sus scrofa OX=9823 GN=NDUFS7 PE=1 SV=1;tr|A0A

11153000 0 16143000 31800000 16137000 21095000 25669000 0 22957000 32378000 17495000 33526000 7 7 7 20,9 20,9 20,9 43,656 0 16,62 409620000 39 A0A287B9G0;F1SDC7;A0A287BBJ7 A0A287B9G0;F1SDC7;A0A287BBJ7 460 tr|A0A287B9G0|A0A287B9G0_PIG Aldehyde dehydrogenase 3 family member A2 OS=Sus scrofa OX=9823 GN=ALDH3A2 PE=1 SV=2;tr|F1SDC7|F1SDC7_PIG Aldehyde dehydrogenase OS=Sus scrofa OX=9823 GN=ALDH3A2 PE=1 SV=4;tr|A0A287BBJ7|A0A287BBJ7_PIG Aldehyde dehydrogenase 3 f

94483000 57246000 120810000 68474000 30443000 183160000 72969000 40111000 65477000 79068000 37290000 130790000 8 3 2 17 7,3 5,7 46,798 0 32,057 1591600000 43 A0A287B9R5;F1SCD0 A0A287B9R5;F1SCD0 461 tr|A0A287B9R5|A0A287B9R5_PIG SERPIN domain-containing protein OS=Sus scrofa OX=9823 GN=LOC100156325 PE=1 SV=1;tr|F1SCD0|F1SCD0_PIG SERPIN domain-containing protein OS=Sus scrofa OX=9823 GN=LOC100156325 PE=1 SV=2

0 0 0 0 0 0 0 0 0 0 0 0 1 1 1 13 13 13 13,938 0,0032223 2,1176 38590000 1 F1S393;A0A287BA78;A0A5G2Q9T3;A0A5G2QSC0 F1S393;A0A287BA78;A0A5G2Q9T3;A0A5G2QSC0 463 tr|F1S393|F1S393_PIG Cytochrome b5 type B OS=Sus scrofa OX=9823 GN=CYB5B PE=1 SV=2;tr|A0A287BA78|A0A287BA78_PIG Cytochrome b5 type B OS=Sus scrofa OX=9823 GN=CYB5B PE=1 SV=2;tr|A0A5G2Q9T3|A0A5G2Q9T3_PIG Cytochrome b5 type B OS=Sus scrofa OX=9823 GN=CYB5B P

12121000 0 30656000 25635000 20322000 29954000 15479000 0 38057000 36225000 21005000 42282000 4 4 4 31 31 31 13,303 0 35,01 608170000 25 A0A287BAW0;A0A5G2RN85;A0A5G2QYE9;A0A5G2R584 A0A287BAW0;A0A5G2RN85;A0A5G2QYE9;A0A5G2R584 464 tr|A0A287BAW0|A0A287BAW0_PIG Complex I subunit B13 OS=Sus scrofa OX=9823 GN=NDUFA5 PE=1 SV=2;tr|A0A5G2RN85|A0A5G2RN85_PIG Complex I subunit B13 OS=Sus scrofa OX=9823 GN=NDUFA5 PE=1 SV=1;tr|A0A5G2QYE9|A0A5G2QYE9_PIG Complex I subunit B13 OS=Sus scrofa OX=98

15073000 11347000 0 28322000 8654700 0 0 0 11731000 17706000 0 0 3 3 3 15 15 15 23,684 0 8,7139 239570000 19 A0A287BAX8;A0A5G2QYW6;F1SN05;A0A5G2R3H6;I3LER8 A0A287BAX8;A0A5G2QYW6;F1SN05;A0A5G2R3H6;I3LER8 465 tr|A0A287BAX8|A0A287BAX8_PIG Eukaryotic translation initiation factor 3 subunit J OS=Sus

scrofa OX=9823 GN=EIF3J PE=1 SV=1;tr|A0A5G2QYW6|A0A5G2QYW6_PIG Eukaryotic translation initiation factor 3 subunit J OS=Sus scrofa OX=9823 GN=EIF3J PE=1 SV=1;tr|F1SN05|
0 0 0 0 0 0 0 0 0 0 0 0 2 2 2 2,3 2,3 2,3
102,52 0 11,496 110770000 8 A0A287BB12;A0A287AU07;I3L6I2;A0A286ZYM7
A0A287BB12;A0A287AU07;I3L6I2;A0A286ZYM7 466 tr|A0A287BB12|A0A287BB12_PIG Methylcrotonoyl-CoA carboxylase 1 OS=Sus scrofa OX=9823 GN=MCCC1 PE=1 SV=2;tr|A0A287AU07|A0A287AU07_PIG DCN1-like protein OS=Sus scrofa OX=9823 GN=DCUN1D2 PE=4 SV=1;tr|I3L6I2|I3L6I2_PIG DCN1-like protein OS=Sus scrofa OX=9823 G
16422000 0 22509000 0 13984000 0 0 0 0 0 0 13313000 4 4
4 12 12 12 45,618 0 10,247 186570000 8
A0A5S6GQX9;A0A287BB72;P54612;A0A5G2QGH9;A0A480F350
A0A5S6GQX9;A0A287BB72;P54612;A0A5G2QGH9;A0A480F350 467
tr|A0A5S6GQX9|A0A5S6GQX9_PIG Serine/threonine-protein phosphatase 2A 65 kDa regulatory subunit A alpha isoform OS=Sus scrofa OX=9823 GN=PPP2R1A PE=4 SV=1;tr|A0A287BB72|A0A287BB72_PIG Serine/threonine-protein phosphatase 2A 65 kDa regulatory subunit A alpha
38647000 14301000 8940000 84145000 11285000 6321900 73139000 15033000 10501000 65838000 0
0 6 6 6 35,8 35,8 35,8 27,946 0 79,739 1120400000 56
A0A5G2Q828;A0A287BB89;F1RWH7;A0A287BDC6
A0A5G2Q828;A0A287BB89;F1RWH7;A0A287BDC6 468 tr|A0A5G2Q828|A0A5G2Q828_PIG Pyridoxal 5-phosphate synthase OS=Sus scrofa OX=9823 GN=PNPO PE=1 SV=1;tr|A0A287BB89|A0A287BB89_PIG Pyridoxal 5-phosphate synthase OS=Sus scrofa OX=9823 GN=PNPO PE=1 SV=1;tr|F1RWH7|F1RWH7_PIG Pyridoxal 5-phosphate synthase O
0 0 5710200 0 0 0 0 0 5155100 0 0 0 2 2 2 8,2
8,2 8,2 30,189 0,0049456 1,5564 63361000 3 A0A5S6HDI4;A0A287BBI3;Q2YGT9
A0A5S6HDI4;A0A287BBI3;Q2YGT9 469 tr|A0A5S6HDI4|A0A5S6HDI4_PIG 60S ribosomal protein L6 OS=Sus scrofa OX=9823 GN=RPL6 PE=3 SV=1;tr|A0A287BBI3|A0A287BBI3_PIG 60S ribosomal protein L6 OS=Sus scrofa OX=9823 GN=RPL6 PE=3 SV=1;sp|Q2YGT9|RL6_PIG 60S ribosomal protein L6 OS=Sus scrofa OX=9823 GN=
0 0 0 0 0 50909000 0 0 0 10416000 0 0 6 5 5 36,4
27,3 27,3 14,922 0 15,573 265360000 8
P86412;A0A287BBN2;A0A5S6I907;A0A5S6HKQ5 P86412;A0A287BBN2;A0A5S6I907 470
sp|P86412|MYP2_PIG Myelin P2 protein OS=Sus scrofa OX=9823 GN=PMP2 PE=1 SV=1;tr|A0A287BBN2|A0A287BBN2_PIG Myelin P2 protein OS=Sus scrofa OX=9823 GN=PMP2 PE=3 SV=1;tr|A0A5S6I907|A0A5S6I907_PIG Myelin P2 protein OS=Sus scrofa OX=9823 GN=PMP2 PE=3 SV=1
0 0 0 0 0 0 0 0 0 0 0 0 1 1 1 7,6 7,6 7,6
19,881 0,0011614 2,7996 95495000 6 A0A287BCM2;F1RZR6 A0A287BCM2;F1RZR6
471 tr|A0A287BCM2|A0A287BCM2_PIG CutA divalent cation tolerance homolog OS=Sus scrofa OX=9823 GN=CUTA PE=1 SV=1;tr|F1RZR6|F1RZR6_PIG CutA divalent cation tolerance homolog OS=Sus scrofa OX=9823 GN=CUTA PE=1 SV=2
39086000 29074000 35566000 39054000 39030000 34008000 47817000 25620000 23671000 38977000
30055000 31229000 6 6 1 36,4 36,4 6,9 19,018 0 25,396

637000000 35

A0A5G2QCI0;I3L6D2;Q52NJ2;F2Z5U4;A0A287BD00;A0A5G2QTM4;A0A5G2Q820;A0A5G2RA85;A0A5G2QA22

A0A5G2QCI0;I3L6D2;Q52NJ2;F2Z5U4;A0A287BD00;A0A5G2QTM4;A0A5G2Q820;A0A5G2RA85 472 tr|A0A5G2QCI0|A0A5G2QCI0_PIG Ras-related protein Rab-1A OS=Sus scrofa OX=9823 GN=RAB1A PE=1 SV=1;tr|I3L6D2|I3L6D2_PIG Ras-related protein Rab-1A OS=Sus scrofa OX=9823 GN=RAB1A PE=1 SV=3;sp|Q52NJ2|RAB1A_PIG Ras-related protein Rab-1A OS=Sus scrofa OX=9823 G

37056000 35359000 47237000 183460000 27118000 65947000 92276000 159340000 54724000

119920000 47183000 88283000 22 2 0 55,4 10,2 0 33,109 0

73,106 1379900000 43 A0A287BDL6 A0A287BDL6 473

tr|A0A287BDL6|A0A287BDL6_PIG Tropomyosin alpha-3 chain OS=Sus scrofa OX=9823 GN=TPM3 PE=1 SV=2

0 0 0 0 0 0 0 0 0 0 0 0 1 1 1 25 25 25

5,5151 0 2,9217 6638700 1 A0A287BDP1 A0A287BDP1 474

tr|A0A287BDP1|A0A287BDP1_PIG Uncharacterized protein OS=Sus scrofa OX=9823 PE=1 SV=2

0 0 0 0 0 0 0 0 0 0 0 0 3 3 3 10,9 10,9 10,9

33,935 0 4,748 3430200 3 A0A287BHL2;A0A287BDT8 A0A287BHL2;A0A287BDT8

475 tr|A0A287BHL2|A0A287BHL2_PIG Phosphoglycolate phosphatase OS=Sus scrofa OX=9823 GN=PGP PE=1 SV=2;tr|A0A287BDT8|A0A287BDT8_PIG Phosphoglycolate phosphatase OS=Sus scrofa OX=9823 GN=PGP PE=1 SV=1

0 0 0 0 0 0 0 0 0 0 0 0 2 2 2 7,1 7,1 7,1

37,203 0,0030395 1,7633 2495600 2 A0A287BE42 A0A287BE42 476

tr|A0A287BE42|A0A287BE42_PIG NIMA related kinase 7 OS=Sus scrofa OX=9823 GN=NEK7 PE=1 SV=2

17324000 17700000 0 34165000 16880000 11851000 34254000 8992000 10941000 27951000 11337000

0 3 3 3 18,5 18,5 18,5 24,074 0 10,044 392160000 20

A0A287BEC7;A0A5G2R1F8;A0A287A9H6;A0A286ZY50;A0A5G2R276;A0A287BGB3;F1SL42;A0A287BBQ2;A0A5G2QT82;A0A5G2RDW5;A0A5G2R892

A0A287BEC7;A0A5G2R1F8;A0A287A9H6;A0A286ZY50;A0A5G2R276 477

tr|A0A287BEC7|A0A287BEC7_PIG Vesicle associated membrane protein 3 OS=Sus scrofa OX=9823 GN=VAMP3 PE=1 SV=2;tr|A0A5G2R1F8|A0A5G2R1F8_PIG Vesicle associated membrane protein 3 OS=Sus scrofa OX=9823 GN=VAMP3 PE=1 SV=1;tr|A0A287A9H6|A0A287A9H6_PIG Synaptobrev

6187800 0 9807500 14036000 6926300 8016300 13535000 0 0 15584000 0 7814400

2 2 2 20,8 20,8 20,8 18,379 0 10,53 180990000 7

A0A287BFU3;F2Z594;A0A287BEI7;P12682 A0A287BFU3;F2Z594;A0A287BEI7;P12682 478

tr|A0A287BFU3|A0A287BFU3_PIG High mobility group protein B1 OS=Sus scrofa OX=9823 GN=HMGB1 PE=1 SV=1;tr|F2Z594|F2Z594_PIG High mobility group protein 1 OS=Sus scrofa OX=9823 GN=HMGB1 PE=1 SV=3;tr|A0A287BEI7|A0A287BEI7_PIG High mobility group protein B1 OS=

0 0 0 0 0 0 0 0 0 0 0 0 1 1 1 11,2 11,2 11,2 11,213 0 4,0229 7782000 4 K9J4R8;A0A287BEK8 K9J4R8;A0A287BEK8 479 tr|K9J4R8|K9J4R8_PIG Protein S100-A13 OS=Sus scrofa OX=9823 GN=S100A13 PE=1 SV=1;tr|A0A287BEK8|A0A287BEK8_PIG S100 calcium binding protein A13 OS=Sus scrofa OX=9823 GN=S100A13 PE=1 SV=1

0 5263600 5319200 0 9779500 0 0 0 4718600 0 0 0 3 3 1 12,3 12,3 2,7 33,719 0 4,268 77809000 5 A0A5G2Q7R9;F1RL80;A0A287BER5;A0A5G2Q850;A0A5G2RDA5;F1RL81;A0A287B3D6 A0A5G2Q7R9;F1RL80;A0A287BER5;A0A5G2Q850;A0A5G2RDA5;F1RL81;A0A287B3D6 480 tr|A0A5G2Q7R9|A0A5G2Q7R9_PIG Branched-chain-amino-acid aminotransferase OS=Sus scrofa OX=9823 GN=BCAT2 PE=1 SV=1;tr|F1RL80|F1RL80_PIG Branched-chain-amino-acid aminotransferase OS=Sus scrofa OX=9823 GN=BCAT2 PE=1 SV=3;tr|A0A287BER5|A0A287BER5_PIG Branched-

0 0 0 0 0 0 0 0 0 0 0 0 1 1 1 3,8 3,8 3,8 32,282 0,003125 1,92 489560000 2 A0A287BF04 A0A287BF04 481 tr|A0A287BF04|A0A287BF04_PIG 2-oxoglutarate and iron dependent oxygenase domain containing 2 OS=Sus scrofa OX=9823 GN=OGFOD2 PE=4 SV=2

27985000 12169000 0 0 0 0 10879000 0 0 10575000 0 0 5 5 1 10,7 10,7 3,5 74,866 0 32,177 218920000 12 A0A287BF97 A0A287BF97 482 tr|A0A287BF97|A0A287BF97_PIG Uncharacterized protein OS=Sus scrofa OX=9823 GN=EEF1D PE=1 SV=2

6701700 6585100 8347000 0 4221200 5241900 0 0 6737700 0 5112300 4782100 5 5 5 9,3 9,3 9,3 66,955 0 8,8019 128760000 13 A0A287BFC9;Q9GMB0;A0A287BR84;A0A5G2R686;F1SPG2 A0A287BFC9;Q9GMB0;A0A287BR84;A0A5G2R686;F1SPG2 483 tr|A0A287BFC9|A0A287BFC9_PIG Dolichyl-diphosphooligosaccharide--protein glycosyltransferase subunit 1 OS=Sus scrofa OX=9823 GN=RPN1 PE=3 SV=1;sp|Q9GMB0|RPN1_PIG Dolichyl-diphosphooligosaccharide--protein glycosyltransferase subunit 1 OS=Sus scrofa OX=9823

8916700 9633000 0 3397800 6314100 3968200 10964000 5290200 3873800 4763700 0 3962800 3 3 3 20 20 20 16,357 0 5,2364 123580000 14 F1S9R0;A0A287BFL8;A0A287BG92;A0A287BJ15 F1S9R0;A0A287BFL8;A0A287BG92;A0A287BJ15 484 tr|F1S9R0|F1S9R0_PIG Uncharacterized protein OS=Sus scrofa OX=9823 GN=MSH4 PE=1 SV=1;tr|A0A287BFL8|A0A287BFL8_PIG Uncharacterized protein OS=Sus scrofa OX=9823 GN=MSH4 PE=1 SV=2;tr|A0A287BG92|A0A287BG92_PIG Geranylgeranyl transferase type-2 subunit beta OS

5486500 0 0 6373700 0 4002500 9399500 0 0 5281000 0 0 5 5 5 10,7 10,7 10,7 66,598 0 12,815 102220000 21 I3L9I6;A0A5G2QRV6;A0A5G2QY76;A0A5G2QW74;A0A287BFP3;K7GPM6 I3L9I6;A0A5G2QRV6;A0A5G2QY76;A0A5G2QW74;A0A287BFP3;K7GPM6 485 tr|I3L9I6|I3L9I6_PIG Basal cell adhesion molecule OS=Sus scrofa OX=9823 GN=BCAM PE=1 SV=2;tr|A0A5G2QRV6|A0A5G2QRV6_PIG Basal cell adhesion molecule OS=Sus scrofa OX=9823

GN=BCAM PE=1 SV=1;tr|A0A5G2QY76|A0A5G2QY76_PIG Basal cell adhesion molecule OS=Sus scr
7122500 0 0 4266700 0 0 0 0 0 0 0 0 2 2 2 8,8
8,8 8,8 21,065 0 2,8805 31961000 6 A0A287BFZ5;A0A287BG47;F1S398
A0A287BFZ5;A0A287BG47;F1S398 486 tr|A0A287BFZ5|A0A287BFZ5_PIG Clathrin light chain OS=Sus scrofa OX=9823 GN=CLTB PE=1 SV=1;tr|A0A287BG47|A0A287BG47_PIG Clathrin light chain OS=Sus scrofa OX=9823 GN=CLTB PE=1 SV=1;tr|F1S398|F1S398_PIG Clathrin light chain OS=Sus scrofa OX=9823 GN=CLTB PE=1
82995000 81677000 180530000 101070000 147720000 181540000 73449000 67323000
140620000 118650000 150650000 281050000 7 7 7 20,3 20,3
20,3 39,888 0 23,765 6272200000 104
F1SQT3;A0A5G2Q9K8;A0A287BGP7;A0A5G2QMB3;A0A287AB71
F1SQT3;A0A5G2Q9K8;A0A287BGP7;A0A5G2QMB3;A0A287AB71 487
tr|F1SQT3|F1SQT3_PIG Phosphate carrier protein, mitochondrial OS=Sus scrofa OX=9823 GN=SLC25A3 PE=1 SV=2;tr|A0A5G2Q9K8|A0A5G2Q9K8_PIG Phosphate carrier protein, mitochondrial OS=Sus scrofa OX=9823 GN=SLC25A3 PE=1 SV=1;tr|A0A287BGP7|A0A287BGP7_PIG Phosphate
15002000 10862000 9754300 0 0 8334100 0 0 9039100 6198400 0 7775600
7 7 7 5 5 5 182,11 0 10,941 241500000 10
A0A5G2QZ02;A0A287BGV6;F1S285;K7GT00;A0A5G2QE84;A0A480I6I0;A0A5G2R816;A0A5G2QYZ9 A0A5G2QZ02;A0A287BGV6;F1S285;K7GT00;A0A5G2QE84;A0A480I6I0 488
tr|A0A5G2QZ02|A0A5G2QZ02_PIG Collagen type XIV alpha 1 chain OS=Sus scrofa OX=9823 GN=COL14A1 PE=1 SV=1;tr|A0A287BGV6|A0A287BGV6_PIG Collagen type XIV alpha 1 chain OS=Sus scrofa OX=9823 GN=COL14A1 PE=1 SV=1;tr|F1S285|F1S285_PIG Collagen type XIV alpha 1 c
0 0 0 0 0 0 0 0 0 0 0 0 1 1 1 5,1 5,1 5,1
34,143 0 5,0203 33904000 4 A0A287BGW4 A0A287BGW4 489
tr|A0A287BGW4|A0A287BGW4_PIG Delta-aminolevulinic acid dehydratase OS=Sus scrofa OX=9823 GN=ALAD PE=1 SV=2
78616000 149480000 350990000 124560000 296940000 323880000 81190000
171440000 252360000 109600000 296120000 467990000 17 17
17 39,5 39,5 39,5 47,436 0 131,98 14962000000 268
P00506;A0A287BH33;A0A5G2Q7P6;A0A5G2R2E7;A0A5S6HCM2
P00506;A0A287BH33;A0A5G2Q7P6;A0A5G2R2E7;A0A5S6HCM2 490 sp|P00506|AATM_PIG Aspartate aminotransferase, mitochondrial OS=Sus scrofa OX=9823 GN=GOT2 PE=1 SV=2;tr|A0A287BH33|A0A287BH33_PIG Aspartate aminotransferase OS=Sus scrofa OX=9823 GN=GOT2 PE=1 SV=1;tr|A0A5G2Q7P6|A0A5G2Q7P6_PIG Aspartate aminotransferase OS=
2256200000 802670000 347380000 3958200000 514790000 680690000 3047700000
636740000 702480000 1587200000 478970000 318450000 25 25
4 57 57 10,4 35,381 0 323,31 123470000000 951
A0A287BHM1;A0A287B5N7;A0A5G2QG60;A0A286ZRE1;A0A287BCP0;F2Z5B6;A0A287BRY0;A0A5G2R733 A0A287BHM1;A0A287B5N7;A0A5G2QG60;A0A286ZRE1;A0A287BCP0;F2Z5B6 491
tr|A0A287BHM1|A0A287BHM1_PIG Tropomyosin alpha-1 chain OS=Sus scrofa OX=9823

GN=TPM1 PE=1 SV=2;tr|A0A287B5N7|A0A287B5N7_PIG Tropomyosin alpha-1 chain OS=Sus scrofa OX=9823 GN=TPM1 PE=1 SV=2;tr|A0A5G2QG60|A0A5G2QG60_PIG Tropomyosin alpha-1 chain OS=Sus scro

63931000 57743000 38227000 95097000 44509000 40486000 111860000 43146000 32064000 79276000 37271000 38950000 12 12 10 28 28 25,1 56,92 0 107,21 3175500000 83 Q2XQV4;A0A287BHY7;A0A287A8H8;A0A2C9F3B1;A0A5G2R5I9;I3LSK6;I3LK62;A0A5G2RN58;F1ST54 Q2XQV4;A0A287BHY7;A0A287A8H8;A0A2C9F3B1 492 sp|Q2XQV4|ALDH2_PIG Aldehyde dehydrogenase, mitochondrial OS=Sus scrofa OX=9823 GN=ALDH2 PE=2 SV=1;tr|A0A287BHY7|A0A287BHY7_PIG Aldehyde dehydrogenase, mitochondrial OS=Sus scrofa OX=9823 GN=ALDH2 PE=1 SV=2;tr|A0A287A8H8|A0A287A8H8_PIG Aldehyde dehydrogena

30916000 9546900 18496000 20950000 16028000 33008000 16655000 6866600 19263000 42476000 18248000 29449000 3 3 3 17,6 17,6 17,6 20,318 0 8,1736 500930000 27 A0A287BI30 A0A287BI30 493 tr|A0A287BI30|A0A287BI30_PIG Calponin-homology (CH) domain-containing protein OS=Sus scrofa OX=9823 GN=SMTNL1 PE=4 SV=2

212710000 163520000 210960000 246430000 222840000 299110000 286970000 150000000 255270000 353810000 197630000 256070000 9 8 8 51,4 46,3 46,3 23,839 0 49,355 8140200000 144 A0A287BI36;A0A287A7G2;F1RWW4;A0A287A1G4;A0A287A3D2 A0A287BI36;A0A287A7G2;F1RWW4;A0A287A1G4;A0A287A3D2 494 tr|A0A287BI36|A0A287BI36_PIG PDZ and LIM domain 5 OS=Sus scrofa OX=9823 GN=PDLIM5 PE=1 SV=1;tr|A0A287A7G2|A0A287A7G2_PIG PDZ and LIM domain 5 OS=Sus scrofa OX=9823 GN=PDLIM5 PE=1 SV=1;tr|F1RWW4|F1RWW4_PIG PDZ and LIM domain 5 OS=Sus scrofa OX=9823 GN=PDLIM

24243000 20038000 19576000 25327000 17690000 24554000 22403000 14967000 20405000 23307000 18840000 21959000 17 16 16 29,7 27,2 27,2 73,008 0 70,538 1902200000 128 A0A287BIL8;P34935 A0A287BIL8 495 tr|A0A287BIL8|A0A287BIL8_PIG 78 kDa glucose-regulated protein OS=Sus scrofa OX=9823 GN=HSPA5 PE=1 SV=2

0 0 0 0 0 0 0 0 0 0 0 0 1 1 1 4,5 4,5 4,5 40,23 0 3,1939 18163000 1 F2Z521;A0A287BJ33;Q9GL51;A0A480UJL5 F2Z521;A0A287BJ33;Q9GL51;A0A480UJL5 496 tr|F2Z521|F2Z521_PIG Platelet-activating factor acetylhydrolase IB subunit alpha OS=Sus scrofa OX=9823 GN=PAFAH1B1 PE=1 SV=2;tr|A0A287BJ33|A0A287BJ33_PIG Platelet-activating factor acetylhydrolase IB subunit alpha OS=Sus scrofa OX=9823 GN=PAFAH1B1 PE=1 SV=

0 0 0 0 0 0 0 0 0 0 0 0 2 2 2 5,5 5,5 5,5 48,194 0 6,8169 23639000 6 F1RWI8;A0A287BJ40;A0A5G2QKI6 F1RWI8;A0A287BJ40;A0A5G2QKI6 497 tr|F1RWI8|F1RWI8_PIG Secernin-2 OS=Sus scrofa OX=9823 GN=SCRN2 PE=1 SV=2;tr|A0A287BJ40|A0A287BJ40_PIG Secernin 2 OS=Sus scrofa OX=9823 GN=SCRN2 PE=1 SV=1;tr|A0A5G2QKI6|A0A5G2QKI6_PIG Secernin 2 OS=Sus scrofa OX=9823 GN=SCRN2 PE=1 SV=1

0 0 0 0 0 0 0 0 0 0 0 0 3 3 3 6,9 6,9 6,9

48,043 0 2,9662 12254000 3 K9IVQ0;F1SI55;A0A287BJF3 K9IVQ0;F1SI55;A0A287BJF3

498 tr|K9IVQ0|K9IVQ0_PIG Basic leucine zipper and W2 domain-containing protein 1 OS=Sus scrofa OX=9823 GN=BZW1 PE=1 SV=1;tr|F1SI55|F1SI55_PIG Basic leucine zipper and W2 domains 1 OS=Sus scrofa OX=9823 GN=BZW1 PE=1 SV=3;tr|A0A287BJF3|A0A287BJF3_PIG Basic leuci

6366900 4836900 0 8846600 0 4578200 10576000 0 14632000 11137000 5287900 5274700

6 6 6 13,8 13,8 13,8 58,571 0 18,226 210330000 31

F1S5Z3;A0A287BJI6 F1S5Z3;A0A287BJI6 499 tr|F1S5Z3|F1S5Z3_PIG Seryl-tRNA synthetase OS=Sus scrofa OX=9823 GN=SARS1 PE=1 SV=3;tr|A0A287BJI6|A0A287BJI6_PIG Seryl-tRNA synthetase OS=Sus scrofa OX=9823 GN=SARS1 PE=1 SV=2

98075000 312920000 116870000 8035700 158200000 27553000 2393900 88405000 66652000

3543200 90444000 11227000 22 22 22 27,3 27,3 27,3 86,502 0 69,135

5252300000 148

B5SYT7;A0A287BJN4;I3LCG9;A0A287BD33;A0A287ATV5;A0A286ZPN9;A0A287A3U3

B5SYT7;A0A287BJN4 500 tr|B5SYT7|B5SYT7_PIG AMP deaminase OS=Sus scrofa OX=9823 GN=AMPD1 PE=1 SV=1;tr|A0A287BJN4|A0A287BJN4_PIG AMP deaminase OS=Sus scrofa OX=9823 GN=AMPD1 PE=1 SV=2

23685000 13729000 11165000 27280000 15510000 15297000 30335000 12520000 13456000 27854000

13227000 10742000 7 7 7 26,3 26,3 26,3 37,115 0 22,815

850150000 59 A0A287BK05 A0A287BK05 501 tr|A0A287BK05|A0A287BK05_PIG Junctional sarcoplasmic reticulum protein 1 OS=Sus scrofa OX=9823 GN=JSRP1 PE=4 SV=1

7464700 8602400 7399800 9969500 6390000 7475000 13406000 0 6613900 13478000 7904500

4817400 6 6 6 19,4 19,4 19,4 38,732 0 20,232 286570000 15

A0A5G2QPL5;A0A287BKA5 A0A5G2QPL5;A0A287BKA5 502

tr|A0A5G2QPL5|A0A5G2QPL5_PIG Uncharacterized protein OS=Sus scrofa OX=9823 GN=PRR33 PE=4 SV=1;tr|A0A287BKA5|A0A287BKA5_PIG Uncharacterized protein OS=Sus scrofa OX=9823 GN=PRR33 PE=4 SV=1

13903000 9666100 11006000 19890000 13736000 15469000 20466000 6966800 9984100 19762000

12811000 13990000 12 12 12 19,6 19,6 19,6 78,993 0 64,733

836150000 77 A0A287BL58;F1RGP4 A0A287BL58;F1RGP4 503

tr|A0A287BL58|A0A287BL58_PIG Pre-B-cell leukemia transcription factor-interacting protein 1 isoform 1 OS=Sus scrofa OX=9823 GN=PBXIP1 PE=1 SV=1;tr|F1RGP4|F1RGP4_PIG Uncharacterized protein OS=Sus scrofa OX=9823 GN=PBXIP1 PE=1 SV=3

0 16559000 12893000 0 11892000 11273000 0 8691100 11974000 0 14855000 8354000

2 2 2 5,1 5,1 5,1 37,945 0,0022124 2,3714 224630000 10

A0A5G2QTJ4;A0A5G2RGJ8;A0A5G2RBK5;A0A287BMG2;A0A287BLP6

A0A5G2QTJ4;A0A5G2RGJ8;A0A5G2RBK5;A0A287BMG2;A0A287BLP6 505

tr|A0A5G2QTJ4|A0A5G2QTJ4_PIG Very-long-chain enoyl-CoA reductase OS=Sus scrofa OX=9823 GN=TECR PE=1 SV=1;tr|A0A5G2RGJ8|A0A5G2RGJ8_PIG Very-long-chain enoyl-CoA reductase OS=Sus scrofa OX=9823 GN=TECR PE=1 SV=1;tr|A0A5G2RBK5|A0A5G2RBK5_PIG Trans-2,3-enoyl-C

3929300 0 0 3411100 0 0 0 0 0 0 0 0 3 3 3 7,7

7,7 7,7 44,855 0 3,1618 30546000 4 A0A287BM29;F1SJT7;O46409

A0A287BM29;F1SJT7;O46409 506 tr|A0A287BM29|A0A287BM29_PIG Apolipoprotein A-IV OS=Sus scrofa OX=9823 GN=APOA4 PE=1 SV=1;tr|F1SJT7|F1SJT7_PIG Apolipoprotein A-IV OS=Sus scrofa OX=9823 GN=APOA4 PE=1 SV=4;sp|O46409|APOA4_PIG Apolipoprotein A-IV OS=Sus scrofa OX=9823 GN=APOA4 PE=2 SV=1

27829000 21268000 65539000 8733600 48142000 14158000 37014000 19883000 36084000 33300000 34553000 34705000 24 24 24 34 34 34 78,032 0 113,67 3270200000 172

A0A287BMY7;A0A287BM44;A0A5G2QI99;A0A480QCC9;A0A5G2QBV9;F1RSZ3;A0A287AIP7;A0A5G2QCS2;A0A5G2RDH5;A0A5G2R2U1;F1RJV7;A0A287ALQ2;A0A5G2Q912;A0A480HMS5;A0A287BLC7;A0A480ZTJ9;A0A5G2QMM8

A0A287BMY7;A0A287BM44;A0A5G2QI99;A0A480QCC9;A0A5G2QBV9;F1RSZ3;A0A287AIP7;A0A5G2QCS2;A0A5G2RDH5;A0A5G2R2U1 507 tr|A0A287BMY7|A0A287BMY7_PIG Acyl-CoA synthetase long chain family member 1 OS=Sus scrofa OX=9823 GN=ACSL1 PE=1 SV=1;tr|A0A287BM44|A0A287BM44_PIG Acyl-CoA synthetase long chain family member 1 OS=Sus scrofa OX=9823 GN=ACSL1 PE=1 SV=1;tr|A0A5G2QI99|A0A5G2QI

0 7447800 13648000 9852900 9429100 7355100 5673200 14383000 13472000 10634000 9766400 17893000 7 7 7 13,9 13,9 13,9 61,142 0 11,929 374310000 33

A0A287BMB4;P13191 A0A287BMB4 508 tr|A0A287BMB4|A0A287BMB4_PIG Alanine aminotransferase 1 OS=Sus scrofa OX=9823 GN=GPT PE=1 SV=1

37195000 16845000 36448000 59381000 28818000 31463000 46390000 0 28131000 98023000 24797000 66852000 3 3 3 26,2 26,2 26,2 18,442 0 35,005 799670000 35

A0A287BMW6;A0A5G2R381 A0A287BMW6;A0A5G2R381 509

tr|A0A287BMW6|A0A287BMW6_PIG Complex I-ESSS OS=Sus scrofa OX=9823 GN=NDUFB11 PE=1 SV=2;tr|A0A5G2R381|A0A5G2R381_PIG Complex I-ESSS OS=Sus scrofa OX=9823 GN=NDUFB11 PE=1 SV=1

3204800 0 4223200 10468000 3162600 9445300 8337800 0 4664700 9068000 4213400 4398100 11 11 11 10,2 10,2 10,2 132,93 0 23,604 281660000 31

I3LJT1;A0A287BMX9;F1RGY5 I3LJT1;A0A287BMX9;F1RGY5 510 tr|I3LJT1|I3LJT1_PIG Nidogen 1 OS=Sus scrofa OX=9823 GN=NID1 PE=1 SV=3;tr|A0A287BMX9|A0A287BMX9_PIG Nidogen 1 OS=Sus scrofa OX=9823 GN=NID1 PE=1 SV=1;tr|F1RGY5|F1RGY5_PIG Nidogen 1 OS=Sus scrofa OX=9823 GN=NID1 PE=1 SV=4

21605000 0 20422000 0 0 18785000 0 0 0 0 0 13556000 152 16 0 31,1 3,8 0 644,24 0 40,478 654450000 47 A0A287BN18 A0A287BN18 511

tr|A0A287BN18|A0A287BN18_PIG Nebulin OS=Sus scrofa OX=9823 GN=NEB PE=1 SV=2

0 0 0 0 0 0 0 0 0 0 0 0 3 1 1 15,7 5,6 5,6 23,466 0,0022051 2,346 0 1 A0A287BN36 A0A287BN36 512

tr|A0A287BN36|A0A287BN36_PIG RAB5C, member RAS oncogene family OS=Sus scrofa OX=9823 GN=RAB5C PE=1 SV=1

0 0 0 0 0 0 0 0 0 0 0 0 3 3 3 6,6 6,6 6,6 58,158 0 3,6026 41164000 5 A0A287BND4;F1SKZ8;A0A480UKD4

A0A287BND4;F1SKZ8;A0A480UKD4 513 tr|A0A287BND4|A0A287BND4_PIG Translocase of outer mitochondrial membrane 70 OS=Sus scrofa OX=9823 GN=TOMM70 PE=1

SV=2;tr|F1SKZ8|F1SKZ8_PIG Mitochondrial import receptor subunit TOM70 OS=Sus scrofa OX=9823 GN=TOMM70 PE=1 SV=3;tr|A0A480UKD4|A0A480UKD4_PIG Mi
0 0 0 0 0 0 6580700 0 0 8084300 0 0 3 3 3 14
14 14 24,728 0 9,535 77703000 16 A0A287BNE4 A0A287BNE4 514
tr|A0A287BNE4|A0A287BNE4_PIG Ubiquitin conjugating enzyme E2 M OS=Sus scrofa OX=9823 GN=UBE2M PE=1 SV=2
31460000 0 36249000 5349300 15245000 9740800 12756000 32704000 6696700 18333000 35506000
32013000 11 11 11 22,7 22,7 22,7 61,815 0 55,023 1105700000 50
A0A287BNL5 A0A287BNL5 515 tr|A0A287BNL5|A0A287BNL5_PIG Uncharacterized protein OS=Sus scrofa OX=9823 PE=4 SV=2
9739900 0 8589600 13355000 0 7796500 17382000 0 10166000 11933000 10140000 0
4 4 4 20,5 20,5 20,5 20,801 0 7,2966 229790000 13
A0A5G2QM16;A0A287BNW6 A0A5G2QM16;A0A287BNW6 516
tr|A0A5G2QM16|A0A5G2QM16_PIG Endothelial differentiation related factor 1 OS=Sus scrofa OX=9823 GN=EDF1 PE=1 SV=1;tr|A0A287BNW6|A0A287BNW6_PIG Endothelial differentiation related factor 1 OS=Sus scrofa OX=9823 GN=EDF1 PE=1 SV=1
0 0 4182000 0 0 8223800 0 0 0 0 0 9034800 4 4 4
9,4 9,4 9,4 58,922 0 8,9272 74587000 8 A0A287BNZ5;F1SJ30;A0A287BL80
A0A287BNZ5;F1SJ30;A0A287BL80 517 tr|A0A287BNZ5|A0A287BNZ5_PIG Mannose-6-phosphate isomerase OS=Sus scrofa OX=9823 GN=MPI PE=1 SV=2;tr|F1SJ30|F1SJ30_PIG Mannose-6-phosphate isomerase OS=Sus scrofa OX=9823 GN=MPI PE=1 SV=3;tr|A0A287BL80|A0A287BL80_PIG Mannose-6-phosphate isomerase OS=Sus sc
0 3379000 6092200 0 0 2843400 0 0 0 0 0 0 2 2 2
5,4 5,4 5,4 49,613 0 6,3331 35617000 3
Q29090;A0A287BPB7;F2Z509;A0A5G2QQ55 Q29090;A0A287BPB7;F2Z509;A0A5G2QQ55
518 sp|Q29090|2ABA_PIG Serine/threonine-protein phosphatase 2A 55 kDa regulatory subunit B alpha isoform (Fragment) OS=Sus scrofa OX=9823 GN=PPP2R2A PE=2 SV=1;tr|A0A287BPB7|A0A287BPB7_PIG Serine/threonine-protein phosphatase 2A 55 kDa regulatory subunit B OS=S
0 5461300 6078000 0 0 6131200 0 0 8085300 0 0 0 2 2
2 11,5 11,5 11,5 15,297 0,0087464 1,4421 63521000 12
Q29197;A0A287BPF2;I3LEX0 Q29197;A0A287BPF2;I3LEX0 519 sp|Q29197|RS9_PIG 40S ribosomal protein S9 (Fragment) OS=Sus scrofa OX=9823 GN=RPS9 PE=2 SV=1;tr|A0A287BPF2|A0A287BPF2_PIG 40S ribosomal protein S9 OS=Sus scrofa OX=9823 GN=RPS9 PE=3 SV=1;tr|I3LEX0|I3LEX0_PIG 40S ribosomal protein S9 OS=Sus scrofa OX=9823
0 0 0 0 0 0 0 0 0 0 0 0 1 1 1 0,7 0,7 0,7
136,93 0,0032258 2,1216 0 1 A0A5G2R855;I3LH91;F1RIW3;A0A287BPI5
A0A5G2R855;I3LH91;F1RIW3;A0A287BPI5 520 tr|A0A5G2R855|A0A5G2R855_PIG Uncharacterized protein OS=Sus scrofa OX=9823 GN=PALLD PE=1 SV=1;tr|I3LH91|I3LH91_PIG Uncharacterized protein OS=Sus scrofa OX=9823 GN=PALLD PE=1 SV=2;tr|F1RIW3|F1RIW3_PIG Palladin isoform X1 OS=Sus scrofa OX=9823 GN=PALLD PE=1
650970000 1022800000 2972500000 870990000 2076400000 2948800000 572990000
1085800000 1952500000 895110000 1916900000 4449400000 14 14

14 31,9 31,9 31,9 48,561 0 45,155 25529000000 154
A0A5G2QGJ8;A0A5G2QGB1;A0A287BPJ7;A0A5G2R343;F1SDN2;A0A5G2RB89;A0A5G2QL38;A0A5G2QPP5;A0A5G2REA8
A0A5G2QGJ8;A0A5G2QGB1;A0A287BPJ7;A0A5G2R343;F1SDN2;A0A5G2RB89;A0A5G2QL38;A0A5G2QPP5;A0A5G2REA8 521 tr|A0A5G2QGJ8|A0A5G2QGJ8_PIG Uncharacterized protein OS=Sus scrofa OX=9823 GN=HADHB PE=1 SV=1;tr|A0A5G2QGB1|A0A5G2QGB1_PIG Uncharacterized protein OS=Sus scrofa OX=9823 GN=HADHB PE=1 SV=1;tr|A0A287BPJ7|A0A287BPJ7_PIG Trifunctional enzyme subunit beta, mito
47658000 54602000 34523000 28990000 37188000 32384000 53210000 26577000 48566000 47838000
24275000 23323000 9 9 9 45,3 45,3 45,3 26,38 0 33,499
1813200000 66 A0A287BPK4;A0A5G2QM25;A0A287AAV2
A0A287BPK4;A0A5G2QM25;A0A287AAV2 522 tr|A0A287BPK4|A0A287BPK4_PIG Myozenin 3 OS=Sus scrofa OX=9823 GN=MYOZ3 PE=1 SV=1;tr|A0A5G2QM25|A0A5G2QM25_PIG Myozenin 3 OS=Sus scrofa OX=9823 GN=MYOZ3 PE=1 SV=1;tr|A0A287AAV2|A0A287AAV2_PIG Myozenin 3 OS=Sus scrofa OX=9823 GN=MYOZ3 PE=1 SV=1
9970100 15102000 15950000 19182000 12326000 16823000 18942000 16240000 12878000 10745000
15161000 15978000 5 5 5 30,7 30,7 30,7 22,419 0 8,4298
497000000 26 I3LQ51;A0A287BPP9 I3LQ51;A0A287BPP9 523 tr|I3LQ51|I3LQ51_PIG Proteasome 20S subunit beta 1 OS=Sus scrofa OX=9823 GN=PSMB1 PE=1 SV=2;tr|A0A287BPP9|A0A287BPP9_PIG Proteasome subunit beta OS=Sus scrofa OX=9823 GN=PSMB1 PE=1 SV=1
8207500 9474900 7571100 14945000 8862200 9562100 11277000 11714000 8322700 17165000
10625000 11406000 5 5 5 16,7 16,7 16,7 37,656 0 9,4719
411580000 30 F1SKL2;A0A287BPT5 F1SKL2;A0A287BPT5 524
tr|F1SKL2|F1SKL2_PIG Sulfurtransferase OS=Sus scrofa OX=9823 GN=TST PE=1 SV=2;tr|A0A287BPT5|A0A287BPT5_PIG Sulfurtransferase OS=Sus scrofa OX=9823 GN=TST PE=1 SV=2
26489000 18704000 65316000 25645000 31763000 47052000 22486000 21968000 43617000 39594000
31383000 84484000 3 3 3 35,3 35,3 35,3 13,759 0 36,765
806620000 34 A0A5G2QVR1;A0A287BPW8 A0A5G2QVR1;A0A287BPW8 525
tr|A0A5G2QVR1|A0A5G2QVR1_PIG Complex I-B15 OS=Sus scrofa OX=9823 GN=NDUFB4 PE=1 SV=1;tr|A0A287BPW8|A0A287BPW8_PIG Complex I-B15 OS=Sus scrofa OX=9823 GN=NDUFB4 PE=1 SV=1
22388000 11923000 13629000 5518800 11393000 6850500 12047000 5019600 4640400 7217200
4923000 10000000 9 3 3 36,5 15,4 15,4 27,568 0 40,014
309020000 26 A0A5G2QTR9;F1SDR7;A0A287BPX7 A0A5G2QTR9;F1SDR7;A0A287BPX7
526 tr|A0A5G2QTR9|A0A5G2QTR9_PIG Tyrosine 3-monooxygenase/tryptophan 5-monooxygenase activation protein beta OS=Sus scrofa OX=9823 GN=YWHAB PE=1 SV=1;tr|F1SDR7|F1SDR7_PIG Tyrosine 3-monooxygenase/tryptophan 5-monooxygenase activation protein beta OS=Sus scrofa
0 0 0 0 0 0 0 0 0 0 0 0 2 2 2 3,8 3,8 3,8
69,534 0 4,2996 22513000 2 F1SV06;A0A287BQ02 F1SV06;A0A287BQ02 527
tr|F1SV06|F1SV06_PIG Polyadenylate-binding protein OS=Sus scrofa OX=9823 GN=PABPC4

PE=1 SV=1;tr|A0A287BQ02|A0A287BQ02_PIG Polyadenylate-binding protein OS=Sus scrofa OX=9823 GN=PABPC4 PE=1 SV=2
0 0 0 4097800 0 0 0 0 0 4491500 0 0 3 3 3 26,9 26,9 26,9 20,688 0 8,2422 31398000 9 A0A287BQ14;A0A5G2QR13;A0A5G2R194 A0A287BQ14;A0A5G2QR13 528 tr|A0A287BQ14|A0A287BQ14_PIG PDGFA associated protein 1 OS=Sus scrofa OX=9823 GN=PDAP1 PE=1 SV=1;tr|A0A5G2QR13|A0A5G2QR13_PIG PDGFA associated protein 1 OS=Sus scrofa OX=9823 GN=PDAP1 PE=1 SV=1
14143000 12443000 10858000 17622000 8333400 10395000 22695000 6011500 0 16412000 8915900 7757200 5 5 5 16,3 16,3 16,3 37,418 0 60,823 326460000 28 F1RYY6;A0A287BQ72 F1RYY6;A0A287BQ72 529 tr|F1RYY6|F1RYY6_PIG Transaldolase OS=Sus scrofa OX=9823 GN=TALDO1 PE=1 SV=3;tr|A0A287BQ72|A0A287BQ72_PIG Transaldolase OS=Sus scrofa OX=9823 GN=TALDO1 PE=1 SV=1
0 13566000 13925000 17758000 0 19003000 15259000 12274000 17811000 13182000 0 10582000 2 2 2 19,3 19,3 19,3 13,333 0 4,3744 241850000 6 A1XQU9;A0A287BQP4;A0A5G2Q7Q1;F1SMQ1 A1XQU9;A0A287BQP4;A0A5G2Q7Q1;F1SMQ1 530 sp|A1XQU9|RS20_PIG 40S ribosomal protein S20 OS=Sus scrofa OX=9823 GN=RPS20 PE=1 SV=1;tr|A0A287BQP4|A0A287BQP4_PIG 40S ribosomal protein S20 OS=Sus scrofa OX=9823 GN=RPS20 PE=3 SV=1;tr|A0A5G2Q7Q1|A0A5G2Q7Q1_PIG 40S ribosomal protein S20 OS=Sus scrofa OX=98
86915000 63952000 76554000 158810000 94785000 102640000 165000000 109830000 118160000 91962000 104720000 109890000 22 19 10 41,3 37,1 21,2 70,097 0 156,61 9313900000 269 Q6S4N2;A0A287BQU9;P34930;P34934 Q6S4N2;A0A287BQU9;P34930 531 sp|Q6S4N2|HS71B_PIG Heat shock 70 kDa protein 1B OS=Sus scrofa OX=9823 GN=HSPA1B PE=2 SV=1;tr|A0A287BQU9|A0A287BQU9_PIG Uncharacterized protein OS=Sus scrofa OX=9823 PE=3 SV=2;sp|P34930|HS71A_PIG Heat shock 70 kDa protein 1A OS=Sus scrofa OX=9823 GN=HSPA1A
0 0 0 0 0 0 0 0 0 0 0 0 1 1 1 5,9 5,9 5,9 36,606 0 2,891 6778300 2 A0A287BR32;F1S1J9 A0A287BR32;F1S1J9 532 tr|A0A287BR32|A0A287BR32_PIG Eukaryotic translation initiation factor 3 subunit H OS=Sus scrofa OX=9823 GN=EIF3H PE=1 SV=2;tr|F1S1J9|F1S1J9_PIG Eukaryotic translation initiation factor 3 subunit H OS=Sus scrofa OX=9823 GN=EIF3H PE=1 SV=2
0 0 0 9302800 0 0 0 0 5471100 6129000 0 0 4 2 2 8,4 4,9 4,9 46,989 0 3,8276 57691000 9 A0A287BRG6;A0A5G2R4G6;A0A5G2QQ66;I3LRS5;A0A5G2QF01;A0A5G2QBN6;A0A5G2REH1;I3LTV1;I3LK50;F1SR94;A0A5G2QN23;A0A480JMD7 A0A287BRG6;A0A5G2R4G6;A0A5G2QQ66;I3LRS5;A0A5G2QF01;A0A5G2QBN6;A0A5G2REH1;I3LTV1;I3LK50;F1SR94;A0A5G2QN23;A0A480JMD7 533 tr|A0A287BRG6|A0A287BRG6_PIG Aldehyde dehydrogenase 1 family member A1 OS=Sus scrofa OX=9823 GN=ALDH1A1 PE=1 SV=1;tr|A0A5G2R4G6|A0A5G2R4G6_PIG Aldehyde dehydrogenase 1 family member A1 OS=Sus scrofa OX=9823 GN=ALDH1A1 PE=1 SV=1;tr|A0A5G2QQ66|A0A5G2QQ66_PIG
0 8644600 0 10170000 0 0 15553000 0 0 17533000 0 0 3 3 3 16,3 16,3 16,3 31,583 0 22,265 142270000 16 A0A287BRL8 A0A287BRL8 534

tr|A0A287BRL8|A0A287BRL8_PIG Myristoylated alanine rich protein kinase C substrate OS=Sus scrofa OX=9823 GN=MARCKS PE=1 SV=1
29646000 17795000 16413000 0 10767000 0 0 0 0 0 0 0 4 4
4 26,3 26,3 26,3 24,195 0 10,71 204570000 12
A0A5G2QK14;F1RHF0;A0A5G2RLD8;A0A287BRT0;A0A5G2QHZ0;A0A5G2QNC6;Q06AB3
A0A5G2QK14;F1RHF0;A0A5G2RLD8;A0A287BRT0;A0A5G2QHZ0;A0A5G2QNC6 535
tr|A0A5G2QK14|A0A5G2QK14_PIG Ubiquitin carboxyl-terminal hydrolase OS=Sus scrofa OX=9823 GN=UCHL3 PE=1 SV=1;tr|F1RHF0|F1RHF0_PIG Ubiquitin carboxyl-terminal hydrolase OS=Sus scrofa OX=9823 GN=UCHL3 PE=1 SV=4;tr|A0A5G2RLD8|A0A5G2RLD8_PIG Ubiquitin carboxyl-
0 0 0 0 0 0 0 0 0 0 0 0 2 2 2 6,6 6,6 6,6
47,279 0 4,9709 99602000 2 F1SAI9;A0A287BS88 F1SAI9;A0A287BS88 536
tr|F1SAI9|F1SAI9_PIG Solute carrier family 37 member 4 OS=Sus scrofa OX=9823 GN=SLC37A4 PE=1 SV=3;tr|A0A287BS88|A0A287BS88_PIG Solute carrier family 37 member 4 OS=Sus scrofa OX=9823 GN=SLC37A4 PE=1 SV=2
5079800000 4537800000 1716900000 8226200000 3318400000 2166300000 8796000000
5546900000 3227800000 6484000000 3811800000 1623400000 20 20
20 91,1 91,1 91,1 26,667 0 323,31 308770000000 1507
D0G7F6;A0A288CFT0;Q29371;A0A286ZRV2 D0G7F6;A0A288CFT0;Q29371;A0A286ZRV2
537 tr|D0G7F6|D0G7F6_PIG Triosephosphate isomerase OS=Sus scrofa OX=9823 GN=TPI1 PE=1 SV=1;tr|A0A288CFT0|A0A288CFT0_PIG Triosephosphate isomerase OS=Sus scrofa OX=9823 GN=TPI1 PE=1 SV=1;sp|Q29371|TPIS_PIG Triosephosphate isomerase OS=Sus scrofa OX=9823 GN=TPI1
0 0 0 0 0 0 0 0 0 0 0 0 2 2 2 17,6 17,6 17,6
10,485 0,0021716 2,2196 37288000 3 A0A288CFW1;A0A5S6GB93;Q95250
A0A288CFW1;A0A5S6GB93;Q95250 538 tr|A0A288CFW1|A0A288CFW1_PIG Progesterone receptor membrane component 2 OS=Sus scrofa OX=9823 GN=PGRMC2 PE=1 SV=2;tr|A0A5S6GB93|A0A5S6GB93_PIG Membrane-associated progesterone receptor component 1 OS=Sus scrofa OX=9823 GN=PGRMC1 PE=1 SV=1;sp|Q95250|PGRC1_P
15171000 17544000 51556000 8912700 52442000 43872000 4713300 18733000 28985000 13717000
48444000 62193000 7 7 7 16,9 16,9 16,9 42,415 0 12,174
1542800000 44
A0A5G2RC01;A0A5S6HU82;A0A2C9F350;P41367;A0A5G2QVK0;A0A5G2R006
A0A5G2RC01;A0A5S6HU82;A0A2C9F350;P41367;A0A5G2QVK0;A0A5G2R006 539
tr|A0A5G2RC01|A0A5G2RC01_PIG Medium-chain specific acyl-CoA dehydrogenase, mitochondrial OS=Sus scrofa OX=9823 GN=ACADM PE=3 SV=1;tr|A0A5S6HU82|A0A5S6HU82_PIG Medium-chain-specific acyl-CoA dehydrogenase, mitochondrial OS=Sus scrofa OX=9823 GN=ACADM PE=3 S
0 0 0 0 0 0 0 0 0 0 0 0 2 2 2 13,9 13,9 13,9
17,14 0 5,3915 43875000 7
A0A5G2QX13;A0A5G2Q7T0;A0A2C9F352;Q8WNV7;A0A5G2QSV2;A0A5G2QWG0;A0A5G2Q806
A0A5G2QX13;A0A5G2Q7T0;A0A2C9F352;Q8WNV7;A0A5G2QSV2;A0A5G2QWG0;A0A5G2Q

806 540 tr|A0A5G2QX13|A0A5G2QX13_PIG Dehydrogenase/reductase SDR family member 4 OS=Sus scrofa OX=9823 GN=DHRS4 PE=1 SV=1;tr|A0A5G2Q7T0|A0A5G2Q7T0_PIG Dehydrogenase/reductase SDR family member 4 OS=Sus scrofa OX=9823 GN=DHRS4 PE=1 SV=1;tr|A0A2C9F352|A0A2C9F352_PIG

246790000 113400000 147930000 28719000 94329000 72315000 39541000 37375000 56902000 55792000 57330000 110070000 10 10 9 45,4 45,4 41,1 22,941 0 57,969 5626300000 142 Q5S1U1;A0A2C9F366;A0A5S6G3Y8

Q5S1U1;A0A2C9F366;A0A5S6G3Y8 541 sp|Q5S1U1|HSPB1_PIG Heat shock protein beta-1 OS=Sus scrofa OX=9823 GN=HSPB1 PE=2 SV=1;tr|A0A2C9F366|A0A2C9F366_PIG Heat shock 27 kDa protein OS=Sus scrofa OX=9823 GN=HSPB1 PE=3 SV=1;tr|A0A5S6G3Y8|A0A5S6G3Y8_PIG Heat shock 27 kDa protein OS=Sus scrofa OX=9

0 0 10255000 0 6255200 16503000 5094300 0 9998000 0 7967900 0 4 4 4 11,5 11,5 11,5 52,193 0 15,634 125480000 15 A0A2C9F385;P79381

A0A2C9F385;P79381 542 tr|A0A2C9F385|A0A2C9F385_PIG Epoxide hydrolase OS=Sus scrofa OX=9823 GN=EPHX1 PE=3 SV=1;sp|P79381|HYEP_PIG Epoxide hydrolase 1 OS=Sus scrofa OX=9823 GN=EPHX1 PE=2 SV=1

39715000 51582000 97520000 47065000 67414000 66625000 26647000 63294000 88080000 62816000 103250000 161260000 6 6 6 34,6 34,6 34,6 22,503 0 38,347 2436500000 94 A0A5G2QCN5;Q2EN81;A0A2C9F3A3

A0A5G2QCN5;Q2EN81;A0A2C9F3A3 543 tr|A0A5G2QCN5|A0A5G2QCN5_PIG ATP synthase peripheral stalk subunit OSCP OS=Sus scrofa OX=9823 GN=ATP5PO PE=3 SV=1;sp|Q2EN81|ATPO_PIG ATP synthase subunit O, mitochondrial OS=Sus scrofa OX=9823 GN=ATP5PO PE=1 SV=1;tr|A0A2C9F3A3|A0A2C9F3A3_PIG ATP synthase p

22729000 0 41794000 31296000 0 21080000 13764000 0 0 10391000 0 37966000 8 5 5 26,9 22,3 22,3 28,934 0 19,421 419390000 20

A0A5S6GU87;A0A2C9F3E2;Q75ZZ6;A0A5G2QKD4;A0A5G2RDN5;A0A5G2R687;A0A5S6IDN1;A0A5G2RGN3;A0A286ZQY4;A0A5G2Q8N0;I3LS66

A0A5S6GU87;A0A2C9F3E2;Q75ZZ6;A0A5G2QKD4;A0A5G2RDN5;A0A5G2R687;A0A5S6IDN1 544 tr|A0A5S6GU87|A0A5S6GU87_PIG Troponin T, slow skeletal muscle OS=Sus scrofa OX=9823 GN=TNNT1 PE=1 SV=1;tr|A0A2C9F3E2|A0A2C9F3E2_PIG Troponin T, slow skeletal muscle OS=Sus scrofa OX=9823 GN=TNNT1 PE=1 SV=2;sp|Q75ZZ6|TNNT1_PIG Troponin T, slow skeletal musc

11313000 0 5158200 0 0 0 12266000 0 3977200 0 0 0 2 2 2 1,7 1,7 1,7 144,66 0 4,3652 98030000 10 A0A480EA26;A0A287AVF9;I3LIB5

A0A480EA26;A0A287AVF9;I3LIB5 545 tr|A0A480EA26|A0A480EA26_PIG Myopalladin isoform a OS=Sus scrofa OX=9823 GN=MYPN PE=4 SV=1;tr|A0A287AVF9|A0A287AVF9_PIG Uncharacterized protein OS=Sus scrofa OX=9823 GN=MYPN PE=4 SV=1;tr|I3LIB5|I3LIB5_PIG Uncharacterized protein OS=Sus scrofa OX=9823 GN=MY

0 0 0 0 0 0 0 0 0 0 0 0 1 1 1 2,2 2,2 2,2 60,675 0,0031679 2,0116 66598000 3 A0A480EEK4 A0A480EEK4 546

tr|A0A480EEK4|A0A480EEK4_PIG Katanin p60 ATPase-containing subunit A-like 2 OS=Sus scrofa OX=9823 GN=KATNAL2 PE=3 SV=1

8050200 3380600 5292700 13997000 0 3017700 18189000 0 0 16704000 0 3860300
6 6 6 29 29 29 27,004 0 22,231 190840000 29
A0A5G2QE76;Q64L94;A0A480F3K2;F1SGM1;A0A5G2QYQ3
A0A5G2QE76;Q64L94;A0A480F3K2;F1SGM1;A0A5G2QYQ3 547
tr|A0A5G2QE76|A0A5G2QE76_PIG Proteasome activator complex subunit 1 OS=Sus scrofa OX=9823 GN=PSME1 PE=3 SV=1;sp|Q64L94|PSME1_PIG Proteasome activator complex subunit 1 OS=Sus scrofa OX=9823 GN=PSME1 PE=2 SV=1;tr|A0A480F3K2|A0A480F3K2_PIG Proteasome activat
11081000 17790000 22710000 42196000 17190000 19811000 19266000 47712000 80029000 43679000
22116000 38461000 4 4 4 33,6 33,6 33,6 14,306 0 15,536
525160000 30 A0A480JRW3;A0A287AZV7 A0A480JRW3;A0A287AZV7 548
tr|A0A480JRW3|A0A480JRW3_PIG NADH dehydrogenase [ubiquinone] 1 subunit C2 OS=Sus scrofa OX=9823 GN=NDUFC2 PE=1 SV=1;tr|A0A287AZV7|A0A287AZV7_PIG NADH dehydrogenase [ubiquinone] 1 subunit C2 OS=Sus scrofa OX=9823 GN=NDUFC2 PE=1 SV=2
0 0 25381000 26649000 21019000 22178000 27223000 0 19699000 34878000 0 36433000
2 2 2 16,5 16,5 16,5 11,719 0,0022447 2,486 440540000 11
F6Q0K3;A0A5G2QGD4;Q29259;A0A480KM09;A0A286ZVX2
F6Q0K3;A0A5G2QGD4;Q29259;A0A480KM09;A0A286ZVX2 549 tr|F6Q0K3|F6Q0K3_PIG Complex I-B17 OS=Sus scrofa OX=9823 GN=NDUFB6 PE=1 SV=1;tr|A0A5G2QGD4|A0A5G2QGD4_PIG Complex I-B17 OS=Sus scrofa OX=9823 GN=NDUFB6 PE=1 SV=1;sp|Q29259|NDUB6_PIG NADH dehydrogenase [ubiquinone] 1 beta subcomplex subunit 6 OS=Sus scrofa
0 0 0 0 0 0 0 0 0 0 0 0 2 2 2 4,8 4,8 4,8
76,502 0 4,8547 16217000 2
A0A5G2QWK2;F1SK65;A0A5G2QE35;A0A480PP98;A0A5G2QYJ4;A0A287AII8
A0A5G2QWK2;F1SK65;A0A5G2QE35;A0A480PP98;A0A5G2QYJ4;A0A287AII8 550
tr|A0A5G2QWK2|A0A5G2QWK2_PIG 1,4-alpha-glucan branching enzyme OS=Sus scrofa OX=9823 GN=GBE1 PE=1 SV=1;tr|F1SK65|F1SK65_PIG 1,4-alpha-glucan branching enzyme OS=Sus scrofa OX=9823 GN=GBE1 PE=1 SV=4;tr|A0A5G2QE35|A0A5G2QE35_PIG 1,4-alpha-glucan branching en
0 0 0 0 0 0 0 0 0 0 0 0 1 1 1 6,1 6,1 6,1
23,481 0 4,1526 105370000 9
A0A5G2RGV9;A0A480PPD4;I3L594;I3LRD5;A0A5G2QQC0;A0A5G2R4B8;A0A5G2QP32
A0A5G2RGV9;A0A480PPD4;I3L594;I3LRD5;A0A5G2QQC0;A0A5G2R4B8;A0A5G2QP32 551
tr|A0A5G2RGV9|A0A5G2RGV9_PIG Eukaryotic translation initiation factor 4H OS=Sus scrofa OX=9823 GN=EIF4H PE=1 SV=1;tr|A0A480PPD4|A0A480PPD4_PIG Eukaryotic translation initiation factor 4H OS=Sus scrofa OX=9823 GN=EIF4H PE=1 SV=1;tr|I3L594|I3L594_PIG Eukaryo
85618000 53348000 47884000 26403000 52205000 37956000 39416000 39430000 32428000 54068000
46349000 52544000 12 12 12 31 31 31 52,799 0 38,906
2535600000 89 A0A480QW05;F1RIS3;A0A5G2RAP2 A0A480QW05;F1RIS3;A0A5G2RAP2
552 tr|A0A480QW05|A0A480QW05_PIG Mitsugumin-53 OS=Sus scrofa OX=9823 GN=TRIM72 PE=1 SV=1;tr|F1RIS3|F1RIS3_PIG Mitsugumin-53 OS=Sus scrofa OX=9823 GN=TRIM72 PE=1

SV=4;tr|A0A5G2RAP2|A0A5G2RAP2_PIG Mitsugumin-53 OS=Sus scrofa OX=9823 GN=TRIM72 PE=1 SV=1

43424000 19885000 25039000 23515000 16728000 31248000 16823000 33720000 20905000 13206000

25355000 9279900 24 24 18 30,1 30,1 23,3 101,68 0 117,73

2511200000 166

A0A5G2QU26;A0A480REY2;A0A5G2RET9;F1RU49;F1RUT3;A0A5G2R6T7;I3LLY3;A0A287A4Q7;F1RI39;A0A287BAZ5;A0A287AA30;A0A287A7I2;A0A287BQV2

A0A5G2QU26;A0A480REY2;A0A5G2RET9;F1RU49;F1RUT3 553

tr|A0A5G2QU26|A0A5G2QU26_PIG Actinin alpha 3 OS=Sus scrofa OX=9823 GN=ACTN3 PE=1 SV=1;tr|A0A480REY2|A0A480REY2_PIG Actinin alpha 3 OS=Sus scrofa OX=9823 GN=ACTN3 PE=1 SV=1;tr|A0A5G2RET9|A0A5G2RET9_PIG Actinin alpha 3 OS=Sus scrofa OX=9823 GN=ACTN3 PE=1 SV=

0 0 0 0 0 0 0 0 0 0 0 0 1 1 1 2,8 2,8 2,8

50,698 0 3,8728 8304300 1 F1RSM2;A0A5G2RF93;A0A480SMR6

F1RSM2;A0A5G2RF93;A0A480SMR6 554 tr|F1RSM2|F1RSM2_PIG Proteasome 26S subunit, non-ATPase 12 OS=Sus scrofa OX=9823 GN=PSMD12 PE=1 SV=2;tr|A0A5G2RF93|A0A5G2RF93_PIG Proteasome 26S subunit, non-ATPase 12 OS=Sus scrofa OX=9823 GN=PSMD12 PE=1 SV=1;tr|A0A480SMR6|A0A480SMR6_PIG 26S proteasome no

18613000 17933000 19400000 13156000 17041000 15509000 24707000 12287000 21587000 17575000

17015000 18748000 3 3 3 19,5 19,5 19,5 20,551 0 9,386

527770000 34 A0A480UG54;I3LCX3 A0A480UG54;I3LCX3 555

tr|A0A480UG54|A0A480UG54_PIG Dual specificity protein phosphatase OS=Sus scrofa OX=9823 GN=DUSP3 PE=1 SV=1;tr|I3LCX3|I3LCX3_PIG Dual specificity protein phosphatase OS=Sus scrofa OX=9823 GN=DUSP3 PE=1 SV=2

7216000 0 6403400 6814800 0 6651000 6145500 0 0 6609000 6895500 6959800

4 4 4 17,3 17,3 17,3 34,776 0 13,691 140890000 11

A0A480VIL3;I3LS60;A0A5G2QRH4 A0A480VIL3;I3LS60;A0A5G2QRH4 556

tr|A0A480VIL3|A0A480VIL3_PIG ATP-dependent (S)-NAD(P)H-hydrate dehydratase OS=Sus scrofa OX=9823 GN=NAXD PE=1 SV=1;tr|I3LS60|I3LS60_PIG ATP-dependent (S)-NAD(P)H-hydrate dehydratase OS=Sus scrofa OX=9823 GN=NAXD PE=1 SV=2;tr|A0A5G2QRH4|A0A5G2QRH4_PIG ATP-d

1889600000 853690000 1217000000 2720300000 888620000 961170000 2820200000

485840000 1101000000 2764700000 796750000 791730000 19 12

2 45,6 27 4,1 47,287 0 277,55 29283000000 625

A0A480WPU1;I3LK59;A0A287A8S8 A0A480WPU1;I3LK59;A0A287A8S8 557

tr|A0A480WPU1|A0A480WPU1_PIG 2-phospho-D-glycerate hydro-lyase OS=Sus scrofa OX=9823 GN=ENO1 PE=1 SV=1;tr|I3LK59|I3LK59_PIG 2-phospho-D-glycerate hydro-lyase OS=Sus scrofa OX=9823 GN=ENO1 PE=1 SV=3;tr|A0A287A8S8|A0A287A8S8_PIG 2-phospho-D-glycerate hydro-l

18920000 0 13324000 22274000 9732300 12248000 21276000 9069500 13824000 27725000 13187000

13937000 4 4 4 21,2 21,2 21,2 23,518 0 26,791 399810000 16

A0A480X841;F1RT87;A0A5G2Q9X9;A0A5G2RFW4;A0A286ZZD9;A0A287A2L2;A0A5G2QFY1;F1S8J6;A0A5G2Q9L1;A0A5G2RBX5

A0A480X841;F1RT87;A0A5G2Q9X9;A0A5G2RFW4;A0A286ZZD9;A0A287A2L2;A0A5G2QFY1;F1S8J6;A0A5G2Q9L1 558 tr|A0A480X841|A0A480X841_PIG RAB2A, member RAS oncogene family OS=Sus scrofa OX=9823 GN=RAB2A PE=1 SV=1;tr|F1RT87|F1RT87_PIG RAB2A, member RAS oncogene family OS=Sus scrofa OX=9823 GN=RAB2A PE=1 SV=4;tr|A0A5G2Q9X9|A0A5G2Q9X9_PIG RAB2A, member RAS oncogene
0 0 0 14714000 0 14005000 13142000 0 10535000 15923000 0 10278000
4 4 4 13,7 13,7 13,7 38,371 0 9,4151 211020000 10
A0A480YV73;F1S3H7;A0A287AI29 A0A480YV73;F1S3H7 559
tr|A0A480YV73|A0A480YV73_PIG 15-oxoprostaglandin 13-reductase OS=Sus scrofa OX=9823 GN=PTGR2 PE=1 SV=1;tr|F1S3H7|F1S3H7_PIG 15-oxoprostaglandin 13-reductase OS=Sus scrofa OX=9823 GN=PTGR2 PE=1 SV=4
19919000 39576000 50790000 3835700 41803000 57438000 0 32236000 79514000 4308500 40642000
54485000 17 17 17 36,6 36,6 36,6 56,943 0 66,644 2966900000 113
A0A481C881;P79303;A0A286ZI08;A0A5G2QMF9 A0A481C881;P79303;A0A286ZI08 560
tr|A0A481C881|A0A481C881_PIG UTP--glucose-1-phosphate uridylyltransferase OS=Sus scrofa OX=9823 GN=UGP2 PE=1 SV=1;sp|P79303|UGPA_PIG UTP--glucose-1-phosphate uridylyltransferase OS=Sus scrofa OX=9823 GN=UGP2 PE=2 SV=3;tr|A0A286ZI08|A0A286ZI08_PIG UTP--gluc
0 0 0 0 0 0 0 0 0 0 0 0 1 1 1 1,4 1,4 1,4
88,416 0,0087549 1,4495 9651000 2
A0A5G2R3H5;F1SLU6;A0A5G2Q6L8;A0A5G2QPU0
A0A5G2R3H5;F1SLU6;A0A5G2Q6L8;A0A5G2QPU0 561
tr|A0A5G2R3H5|A0A5G2R3H5_PIG Ubiquitin carboxyl-terminal hydrolase OS=Sus scrofa OX=9823 GN=USP5 PE=1 SV=1;tr|F1SLU6|F1SLU6_PIG Ubiquitin carboxyl-terminal hydrolase OS=Sus scrofa OX=9823 GN=USP5 PE=1 SV=4;tr|A0A5G2Q6L8|A0A5G2Q6L8_PIG Ubiquitin carboxyl-te
0 0 0 0 0 0 0 0 0 0 0 0 2 2 2 16,5 16,5 16,5
14,844 0,0021763 2,2492 8824200 3
A0A5G2Q6V1;A0A5G2QBU2;A0A5G2QTM2;F1SL99
A0A5G2Q6V1;A0A5G2QBU2;A0A5G2QTM2;F1SL99 562
tr|A0A5G2Q6V1|A0A5G2Q6V1_PIG Prostaglandin E synthase 3 OS=Sus scrofa OX=9823 GN=PTGES3 PE=1 SV=1;tr|A0A5G2QBU2|A0A5G2QBU2_PIG Prostaglandin E synthase 3 OS=Sus scrofa OX=9823 GN=PTGES3 PE=1 SV=1;tr|A0A5G2QTM2|A0A5G2QTM2_PIG Prostaglandin E synthase 3 OS=S
0 11479000 38727000 15478000 23967000 24376000 13951000 0 21861000 29564000 15654000
52710000 4 4 4 32,4 32,4 32,4 16,338 0 10,519 422860000 18
A0A5G2Q729;F1S6Q1 A0A5G2Q729;F1S6Q1 563 tr|A0A5G2Q729|A0A5G2Q729_PIG NADH dehydrogenase [ubiquinone] 1 alpha subcomplex subunit 13 OS=Sus scrofa OX=9823 GN=NDUFA13 PE=1 SV=1;tr|F1S6Q1|F1S6Q1_PIG NADH dehydrogenase [ubiquinone] 1 alpha subcomplex subunit 13 OS=Sus scrofa OX=9823 GN=NDUFA13 PE=1 S
10859000 0 11763000 0 0 12945000 0 0 13541000 0 0 43894000 68
7 1 35,3 4,7 0,8 223,15 0 100,11 358010000 20
Q9TV63;A0A5G2Q7D6;F1SS64;A0A287AZK1 Q9TV63;A0A5G2Q7D6;F1SS64;A0A287AZK1

564 sp|Q9TV63|MYH2_PIG Myosin-2 OS=Sus scrofa OX=9823 GN=MYH2 PE=2 SV=1;tr|A0A5G2Q7D6|A0A5G2Q7D6_PIG Myosin-4 OS=Sus scrofa OX=9823 GN=MYH4 PE=3 SV=1;tr|F1SS64|F1SS64_PIG Myosin-4 OS=Sus scrofa OX=9823 GN=MYH4 PE=3 SV=2;tr|A0A287AZK1|A0A287AZK1_PIG Myosin-4 OS
23988000 33844000 63557000 41520000 65728000 53966000 24016000 38767000 42792000 39987000
68780000 96832000 17 17 17 32,3 32,3 32,3 67,423 0 92,707
4109400000 139 A0A5G2QMG3;A0A5G2Q7D7;Q0QF01;A0A5G2R6Z8
A0A5G2QMG3;A0A5G2Q7D7;Q0QF01;A0A5G2R6Z8 565
tr|A0A5G2QMG3|A0A5G2QMG3_PIG Succinate dehydrogenase [ubiquinone] flavoprotein subunit, mitochondrial OS=Sus scrofa OX=9823 GN=SDHA PE=1 SV=1;tr|A0A5G2Q7D7|A0A5G2Q7D7_PIG Succinate dehydrogenase [ubiquinone] flavoprotein subunit, mitochondrial OS=Sus scrof
0 0 0 0 0 0 0 0 0 0 0 0 2 2 2 7,1 7,1 7,1
47,63 0 6,5238 24998000 4
A0A5G2RA90;A0A5G2R6R2;A0A5G2Q895;A0A5K1UH01
A0A5G2RA90;A0A5G2R6R2;A0A5G2Q895;A0A5K1UH01 566
tr|A0A5G2RA90|A0A5G2RA90_PIG Protein disulfide-isomerase A6 OS=Sus scrofa OX=9823 GN=PDIA6 PE=1 SV=1;tr|A0A5G2R6R2|A0A5G2R6R2_PIG Protein disulfide-isomerase A6 OS=Sus scrofa OX=9823 GN=PDIA6 PE=1 SV=1;tr|A0A5G2Q895|A0A5G2Q895_PIG Protein disulfide-isomera
6970900 7010800 0 0 0 0 0 0 0 0 0 0 3 3 3 8,8
8,8 8,8 53,603 0 4,9783 43812000 6
A0A5G2Q8F9;Q863I2;A0A5S8KP39;A0A5G2Q8B4;A0A480I155
A0A5G2Q8F9;Q863I2;A0A5S8KP39;A0A5G2Q8B4;A0A480I155 567
tr|A0A5G2Q8F9|A0A5G2Q8F9_PIG Non-specific serine/threonine protein kinase OS=Sus scrofa OX=9823 GN=OXSR1 PE=4 SV=1;sp|Q863I2|OXSR1_PIG Serine/threonine-protein kinase OSR1 OS=Sus scrofa OX=9823 GN=OXSR1 PE=2 SV=1;tr|A0A5S8KP39|A0A5S8KP39_PIG Non-specific s
10520000 5034000 7568800 13671000 0 22665000 12567000 0 8249300 9960800 4564100
14194000 12 12 12 8,9 8,9 8,9 163,99 0 32,639 594850000 49
A0A5G2Q8I9;K7GQ48;F1SLX2;CON__ENSEMBL:ENSBTAP00000024146
A0A5G2Q8I9;K7GQ48;F1SLX2 568 tr|A0A5G2Q8I9|A0A5G2Q8I9_PIG Uncharacterized protein OS=Sus scrofa OX=9823 GN=A2M PE=1 SV=1;tr|K7GQ48|K7GQ48_PIG Uncharacterized protein OS=Sus scrofa OX=9823 GN=A2M PE=1 SV=3;tr|F1SLX2|F1SLX2_PIG Uncharacterized protein OS=Sus scrofa OX=9823 GN=A2M PE=1 S
0 0 0 0 0 0 0 0 0 0 0 0 2 2 2 3,8 3,8 3,8
69,683 0,0030612 1,8028 3631300 2
A0A5G2R4G9;F1SP18;A0A5G2Q976;A0A5G2QVT3
A0A5G2R4G9;F1SP18;A0A5G2Q976;A0A5G2QVT3 569 tr|A0A5G2R4G9|A0A5G2R4G9_PIG Threonyl-tRNA synthetase OS=Sus scrofa OX=9823 GN=TARS1 PE=1 SV=1;tr|F1SP18|F1SP18_PIG Threonyl-tRNA synthetase OS=Sus scrofa OX=9823 GN=TARS1 PE=1 SV=4;tr|A0A5G2Q976|A0A5G2Q976_PIG Threonyl-tRNA synthetase OS=Sus scrofa OX=982

0 0 0 0 0 0 0 0 0 0 0 0 1 1 1 3,2 3,2 3,2

31,51 0,0031915 2,0575 39467000 2 F1RW98;A0A5G2Q990 F1RW98;A0A5G2Q990

570 tr|F1RW98|F1RW98_PIG Ras suppressor protein 1 OS=Sus scrofa OX=9823 GN=RSU1 PE=1 SV=3;tr|A0A5G2Q990|A0A5G2Q990_PIG Ras suppressor protein 1 OS=Sus scrofa OX=9823 GN=RSU1 PE=1 SV=1

31597000 26382000 42599000 45970000 28978000 60885000 49107000 23645000 37640000 47987000

35697000 74771000 6 6 6 41,4 41,4 41,4 17,176 0 21,895

1584500000 55 Q2EN76;A0A5G2Q9S0;A0A287AS29;A0A5G2RK94;A0A5G2QTY2

Q2EN76;A0A5G2Q9S0;A0A287AS29 571 sp|Q2EN76|NDKB_PIG Nucleoside diphosphate kinase B OS=Sus scrofa OX=9823 GN=NME2 PE=2 SV=1;tr|A0A5G2Q9S0|A0A5G2Q9S0_PIG Nucleoside diphosphate kinase OS=Sus scrofa OX=9823 GN=NME2 PE=1 SV=1;tr|A0A287AS29|A0A287AS29_PIG Nucleoside diphosphate kinase OS=Sus

22346000 21178000 18285000 44504000 18200000 18875000 43077000 15811000 15433000 44507000

21196000 26054000 14 14 14 32,9 32,9 32,9 56,046 0 63,366

1747400000 91 P28839;A0A5G2QA84;A0A5G2QAG3;A0A5G2R3A2

P28839;A0A5G2QA84;A0A5G2QAG3;A0A5G2R3A2 572 sp|P28839|AMPL_PIG Cytosol aminopeptidase OS=Sus scrofa OX=9823 GN=LAP3 PE=1 SV=2;tr|A0A5G2QA84|A0A5G2QA84_PIG Cysteinylglycine-S-conjugate dipeptidase OS=Sus scrofa OX=9823 GN=LAP3 PE=1 SV=1;tr|A0A5G2QAG3|A0A5G2QAG3_PIG Cysteinylglycine-S-conjugate dipept

9749600 8100400 8799800 5817100 7166200 7615000 12769000 8502900 10819000 8912100

6474300 5939100 7 7 7 10,2 10,2 10,2 94,859 0 11,864

234090000 17 A0A5G2QAB7;F1S166;A0A5G2QDP0;A0A287B0D1

A0A5G2QAB7;F1S166;A0A5G2QDP0;A0A287B0D1 573 tr|A0A5G2QAB7|A0A5G2QAB7_PIG Protein unc-45 homolog B OS=Sus scrofa OX=9823 GN=UNC45B PE=4 SV=1;tr|F1S166|F1S166_PIG Protein unc-45 homolog B OS=Sus scrofa OX=9823 GN=UNC45B PE=4 SV=3;tr|A0A5G2QDP0|A0A5G2QDP0_PIG Protein unc-45 homolog B OS=Sus scrofa OX=9

0 0 0 0 0 0 4155500 0 0 3476300 0 0 2 2 2 13,9

13,9 13,9 16,93 0,003096 1,8647 24775000 2 P60662;A0A5S6FSE5;A0A5G2QAD4

P60662;A0A5S6FSE5;A0A5G2QAD4 574 sp|P60662|MYL6_PIG Myosin light polypeptide 6 OS=Sus scrofa OX=9823 GN=MYL6 PE=1 SV=2;tr|A0A5S6FSE5|A0A5S6FSE5_PIG Uncharacterized protein OS=Sus scrofa OX=9823 GN=MYL6B PE=4 SV=1;tr|A0A5G2QAD4|A0A5G2QAD4_PIG Uncharacterized protein OS=Sus scrofa OX=9823 G

0 0 0 0 0 0 0 0 0 0 0 0 1 1 1 9,5 9,5 9,5

13,651 0,0030992 1,8658 0 2 A0A5G2QAJ6;I3LBW5 A0A5G2QAJ6;I3LBW5 575

tr|A0A5G2QAJ6|A0A5G2QAJ6_PIG Peptidyl-prolyl cis-trans isomerase OS=Sus scrofa OX=9823 GN=PIN4 PE=1 SV=1;tr|I3LBW5|I3LBW5_PIG Peptidyl-prolyl cis-trans isomerase OS=Sus scrofa OX=9823 GN=PIN4 PE=1 SV=1

27869000 17441000 17375000 22749000 15674000 16666000 24173000 10987000 14583000 23820000

14413000 14458000 5 4 4 22,9 17,2 17,2 22,102 0 6,0195

652470000 42

A0A5G2RJU1;F2Z5F2;A0A5G2QAT3;F1RFW5;A0A287AGC0;A0A287A3W8;A0A286ZUS1;F1S091;A0A287AXQ6;A0A287B2E5;F2Z5I8 A0A5G2RJU1;F2Z5F2;A0A5G2QAT3 576

tr|A0A5G2RJU1|A0A5G2RJU1_PIG RAB10, member RAS oncogene family OS=Sus scrofa

OX=9823 GN=RAB10 PE=1 SV=1;tr|F2Z5F2|F2Z5F2_PIG RAB10, member RAS oncogene family OS=Sus scrofa OX=9823 GN=RAB10 PE=1 SV=1;tr|A0A5G2QAT3|A0A5G2QAT3_PIG RAB10, member RAS oncogene

715540000 166810000 242540000 72047000 220620000 116580000 331380000
342730000 192160000 169160000 290950000 60223000 55 55 0
46,5 46,5 0 136,3 0 323,31 42242000000 870
A0A5G2QB13;A0A5G2QJR5;F1SID7;A0A287AFL5;A0A5G2RFI9 A0A5G2QB13 577
tr|A0A5G2QB13|A0A5G2QB13_PIG Myosin binding protein C2 OS=Sus scrofa OX=9823 GN=MYBPC2 PE=1 SV=1

6192700 7868600 7600400 0 8046600 8904700 0 0 6646600 5574800 7328200 8727000
7 7 7 10,9 10,9 10,9 79,891 0 16,824 197420000 26
A0A5G2QPE1;A0A5G2QB32;I3LQD3;P43367 A0A5G2QPE1;A0A5G2QB32;I3LQD3 578
tr|A0A5G2QPE1|A0A5G2QPE1_PIG Calcium-activated neutral proteinase 2 OS=Sus scrofa OX=9823 GN=CAPN2 PE=1 SV=1;tr|A0A5G2QB32|A0A5G2QB32_PIG Calcium-activated neutral proteinase 2 OS=Sus scrofa OX=9823 GN=CAPN2 PE=1 SV=1;tr|I3LQD3|I3LQD3_PIG Calcium-activated

0 0 0 0 0 0 0 0 0 0 0 0 1 1 1 3,7 3,7 3,7
30,309 0,0095602 1,3632 2138600 3 A0A5G2QB67;I3LT95 A0A5G2QB67;I3LT95
579 tr|A0A5G2QB67|A0A5G2QB67_PIG Proteasome assembly chaperone 1 OS=Sus scrofa OX=9823 GN=BRWD1 PE=1 SV=1;tr|I3LT95|I3LT95_PIG Proteasome assembly chaperone 1 OS=Sus scrofa OX=9823 GN=BRWD1 PE=1 SV=2

199160000 0 153200000 0 0 0 0 0 0 0 0 0 7 7
7 9,6 9,6 9,6 104,01 0 9,5175 405420000 9
A0A5G2R5V7;F1RYU9;A0A5G2QB74;A0A287AZ09;A0A287AJL0;A0A286ZLU4
A0A5G2R5V7;F1RYU9;A0A5G2QB74;A0A287AZ09;A0A287AJL0;A0A286ZLU4 580
tr|A0A5G2R5V7|A0A5G2R5V7_PIG USO1 vesicle transport factor OS=Sus scrofa OX=9823 GN=USO1 PE=1 SV=1;tr|F1RYU9|F1RYU9_PIG USO1 vesicle transport factor OS=Sus scrofa OX=9823 GN=USO1 PE=1 SV=3;tr|A0A5G2QB74|A0A5G2QB74_PIG General vesicular transport factor p1

0 0 0 0 0 0 0 0 0 0 0 0 1 1 1 4,9 4,9 4,9
31,261 0,0030272 1,7416 61001000 2
A0A5G2RF55;F1RR69;A0A5G2QKJ5;A0A5G2QKF6;A0A5G2QBC5
A0A5G2RF55;F1RR69;A0A5G2QKJ5;A0A5G2QKF6;A0A5G2QBC5 581
tr|A0A5G2RF55|A0A5G2RF55_PIG Uncharacterized protein OS=Sus scrofa OX=9823 GN=SET PE=1 SV=1;tr|F1RR69|F1RR69_PIG Uncharacterized protein OS=Sus scrofa OX=9823 GN=SET PE=1 SV=3;tr|A0A5G2QKJ5|A0A5G2QKJ5_PIG Uncharacterized protein OS=Sus scrofa OX=9823 GN=SE

30783000 33026000 23637000 50240000 27583000 25045000 53464000 32314000 39228000 48126000
25925000 22174000 7 5 5 26,1 20,6 20,6 30,722 0 12,597
1101600000 44 K7GLN4;F1SQ01;A0A5G2QBQ4 K7GLN4;F1SQ01;A0A5G2QBQ4 582
tr|K7GLN4|K7GLN4_PIG Peroxiredoxin 4 OS=Sus scrofa OX=9823 GN=PRDX4 PE=1 SV=2;tr|F1SQ01|F1SQ01_PIG Peroxiredoxin-4 OS=Sus scrofa OX=9823 GN=PRDX4 PE=1 SV=1;tr|A0A5G2QBQ4|A0A5G2QBQ4_PIG Peroxiredoxin 4 OS=Sus scrofa OX=9823 GN=PRDX4 PE=1 SV=1

0 0 0 0 0 0 0 0 0 0 0 0 1 1 1 9,3 9,3 9,3

14,692 0 3,571 5039800 4 A0A5G2QBU9;A5GHK3 A0A5G2QBU9;A5GHK3

583 tr|A0A5G2QBU9|A0A5G2QBU9_PIG Prefoldin subunit 4 OS=Sus scrofa OX=9823 GN=PFDN4 PE=1 SV=1;tr|A5GHK3|A5GHK3_PIG Prefoldin subunit 4 OS=Sus scrofa OX=9823 GN=PFDN4 PE=1 SV=1

0 0 0 0 0 0 0 0 0 0 0 0 15 1 1 41,9 4,2 4,2

42,215 0,003112 1,8943 153210000 2 A0A5G2QBV6 A0A5G2QBV6 584

tr|A0A5G2QBV6|A0A5G2QBV6_PIG Creatine kinase OS=Sus scrofa OX=9823 GN=CKM PE=1 SV=1

8300900 7522300 8177800 10613000 0 8685600 9605200 6387800 7172500 10266000 7173200

8273500 8 8 8 19,1 19,1 19,1 47,567 0 15,427 208420000 19

A0A5G2QCI8;I3LR43;F1RNW4;A0A5G2QM19;A0A5G2QMV2;A0A5G2QW46;A0A5G2QQV1; A0A5G2RE96

A0A5G2QCI8;I3LR43;F1RNW4;A0A5G2QM19;A0A5G2QMV2;A0A5G2QW46;A0A5G2QQV1; A0A5G2RE96 585 tr|A0A5G2QCI8|A0A5G2QCI8_PIG Peptidase D OS=Sus scrofa OX=9823 GN=PEPD PE=1 SV=1;tr|I3LR43|I3LR43_PIG Peptidase D OS=Sus scrofa OX=9823 GN=PEPD PE=1 SV=2;tr|F1RNW4|F1RNW4_PIG Peptidase D OS=Sus scrofa OX=9823 GN=PEPD PE=1 SV=3;tr|A0A5G2QM19|A0A5G2QM19_PIG

0 0 0 0 0 0 0 0 0 0 0 0 2 2 2 8,8 8,8 8,8

25,048 0 3,2736 98226000 5 A0A5G2QCJ7;F1SKK1 A0A5G2QCJ7;F1SKK1 586

tr|A0A5G2QCJ7|A0A5G2QCJ7_PIG Solute carrier family 25 member 20 OS=Sus scrofa OX=9823 GN=SLC25A20 PE=3 SV=1;tr|F1SKK1|F1SKK1_PIG Mitochondrial carnitine/acylcarnitine carrier protein OS=Sus scrofa OX=9823 GN=SLC25A20 PE=3 SV=1

0 0 0 0 0 0 0 0 0 0 0 0 1 1 1 6,1 6,1 6,1

20,292 0,0059055 1,518 15839000 4 A0A5G2R081;Q29561;A0A5G2QCQ6

A0A5G2R081;Q29561;A0A5G2QCQ6 587 tr|A0A5G2R081|A0A5G2R081_PIG Nucleoside-diphosphate kinase OS=Sus scrofa OX=9823 GN=CMPK1 PE=1 SV=1;sp|Q29561|KCY_PIG UMP-CMP kinase OS=Sus scrofa OX=9823 GN=CMPK1 PE=1 SV=1;tr|A0A5G2QCQ6|A0A5G2QCQ6_PIG Nucleoside-diphosphate kinase OS=Sus scrofa OX=9823 G

0 0 0 0 0 0 0 0 0 0 0 0 2 2 2 11,1 11,1 11,1

25,496 0 5,9993 28117000 4 F1SRV4;A0A5G2QCY7;A0A5G2RLM8;A0A5G2Q9E2

F1SRV4;A0A5G2QCY7;A0A5G2RLM8;A0A5G2Q9E2 588 tr|F1SRV4|F1SRV4_PIG Glutathione S-transferase kappa OS=Sus scrofa OX=9823 GN=GSTK1 PE=1 SV=1;tr|A0A5G2QCY7|A0A5G2QCY7_PIG DSBA domain-containing protein OS=Sus scrofa OX=9823 GN=GSTK1 PE=1 SV=1;tr|A0A5G2RLM8|A0A5G2RLM8_PIG DSBA domain-containing protein O

12399000 5431000 4470600 0 0 0 6993000 0 0 6632500 0 0 8

3 3 16,7 6,4 6,4 56,479 0 5,8694 117080000 3

A0A5G2QD35;K7GMY6;A0A480QFW6;A0A5G2R8A6;F1SU57;A0A287B026;F1RMA9;F1RMA8; A0A481CE02

A0A5G2QD35;K7GMY6;A0A480QFW6;A0A5G2R8A6;F1SU57;A0A287B026;F1RMA9;F1RMA8; A0A481CE02 589 tr|A0A5G2QD35|A0A5G2QD35_PIG Serine/threonine-protein phosphatase OS=Sus scrofa OX=9823 GN=PPP3CB PE=1 SV=1;tr|K7GMY6|K7GMY6_PIG Serine/threonine-

protein phosphatase OS=Sus scrofa OX=9823 GN=PPP3CB PE=1 SV=2;tr|A0A480QFW6|A0A480QFW6_PIG Serine/threonine-pr
0 0 0 0 0 0 0 0 0 0 0 0 1 1 1 7,8 7,8 7,8
26,933 0,0022422 2,4809 3994500 1 F1SLA7;A0A5G2QD86 F1SLA7;A0A5G2QD86
590 tr|F1SLA7|F1SLA7_PIG Signal recognition particle receptor subunit beta OS=Sus scrofa OX=9823 GN=SRPRB PE=1 SV=4;tr|A0A5G2QD86|A0A5G2QD86_PIG Signal recognition particle receptor subunit beta OS=Sus scrofa OX=9823 GN=SRPRB PE=1 SV=1
15995000 0 9306100 0 0 0 0 0 0 0 0 0 6 5 5 12,6
11 11 50,186 0 12,732 67423000 7 A0A5G2QE62;P02543;A0A5S6H025
A0A5G2QE62;P02543;A0A5S6H025 591 tr|A0A5G2QE62|A0A5G2QE62_PIG Vimentin OS=Sus scrofa OX=9823 GN=VIM PE=1 SV=1;sp|P02543|VIME_PIG Vimentin OS=Sus scrofa OX=9823 GN=VIM PE=1 SV=2;tr|A0A5S6H025|A0A5S6H025_PIG Vimentin OS=Sus scrofa OX=9823 GN=VIM PE=1 SV=1
37755000 18353000 17156000 33350000 19303000 13965000 40292000 0 14005000 37165000 14495000
11959000 6 6 2 27,2 27,2 6,7 20,037 0 12,126 663620000 31
A0A5G2RBP9;A0A5G2QE74;Q52NJ1;A0A5G2R7B1;A0A287BGV3
A0A5G2RBP9;A0A5G2QE74;Q52NJ1;A0A5G2R7B1 592 tr|A0A5G2RBP9|A0A5G2RBP9_PIG Uncharacterized protein OS=Sus scrofa OX=9823 GN=RAB11B PE=1 SV=1;tr|A0A5G2QE74|A0A5G2QE74_PIG Ras-related protein Rab-11A OS=Sus scrofa OX=9823 GN=RAB11A PE=1 SV=1;sp|Q52NJ1|RB11A_PIG Ras-related protein Rab-11A OS=Sus scrofa
0 0 0 0 0 0 0 0 0 0 0 0 2 2 2 12 12 12
21,058 0,0087209 1,4283 4077300 2
A0A5G2QEC7;F1RZ28;A0A5G2QG45;A0A5G2QAN6
A0A5G2QEC7;F1RZ28;A0A5G2QG45;A0A5G2QAN6 593
tr|A0A5G2QEC7|A0A5G2QEC7_PIG S10_plectin domain-containing protein OS=Sus scrofa OX=9823 GN=NUDT3 PE=1 SV=1;tr|F1RZ28|F1RZ28_PIG S10_plectin domain-containing protein OS=Sus scrofa OX=9823 GN=NUDT3 PE=1 SV=3;tr|A0A5G2QG45|A0A5G2QG45_PIG Nudix hydrolase dom
9206900 16165000 13890000 5562300 14947000 10734000 1923500 16462000 13745000 5862500
14088000 12460000 15 15 15 19,9 19,9 19,9 96,823 0 35,715
839370000 73 A0A5G2RND7;F1RWJ6;A0A5G2QES1;A0A5G2R745
A0A5G2RND7;F1RWJ6;A0A5G2QES1;A0A5G2R745 594
tr|A0A5G2RND7|A0A5G2RND7_PIG Aminopeptidase OS=Sus scrofa OX=9823 GN=NPEPPS PE=1 SV=1;tr|F1RWJ6|F1RWJ6_PIG Aminopeptidase OS=Sus scrofa OX=9823 GN=NPEPPS PE=1 SV=3;tr|A0A5G2QES1|A0A5G2QES1_PIG Aminopeptidase OS=Sus scrofa OX=9823 GN=NPEPPS PE=1 SV=1;tr|A0A
5716700 0 0 5489100 0 0 9431200 0 0 6733700 0 0 4 4
4 17,5 17,5 17,5 25,918 0 6,3443 72940000 9
A0A5G2QF42;Q863Z0;A0A5G2QKZ5;F1SGM3;A0A5G2QTP4
A0A5G2QF42;Q863Z0;A0A5G2QKZ5;F1SGM3;A0A5G2QTP4 595
tr|A0A5G2QF42|A0A5G2QF42_PIG Proteasome activator complex subunit 2 OS=Sus scrofa OX=9823 GN=PSME2 PE=3 SV=1;sp|Q863Z0|PSME2_PIG Proteasome activator complex subunit 2

OS=Sus scrofa OX=9823 GN=PSME2 PE=2 SV=3;tr|A0A5G2QKZ5|A0A5G2QKZ5_PIG Proteasome activat

229190000 216700000 505700000 396680000 415600000 460830000 332970000 263610000 327140000 451110000 403160000 808790000 15 15 15 43,4 43,4 43,4 36,454 0 85,724 25291000000 340 P11708;A0A5G2QPX2;A0A5G2QFC2;A0A5S8KKT9;A0A5G2QP21;A0A5G2QP84 P11708;A0A5G2QPX2;A0A5G2QFC2;A0A5S8KKT9;A0A5G2QP21;A0A5G2QP84596 sp|P11708|MDHC_PIG Malate dehydrogenase, cytoplasmic OS=Sus scrofa OX=9823 GN=MDH1 PE=1 SV=4;tr|A0A5G2QPX2|A0A5G2QPX2_PIG Malate dehydrogenase OS=Sus scrofa OX=9823 GN=MDH1 PE=1 SV=1;tr|A0A5G2QFC2|A0A5G2QFC2_PIG Malate dehydrogenase OS=Sus scrofa OX=9823 G

51194000 32025000 31452000 62809000 34624000 41608000 77234000 28384000 32204000 58413000 34809000 37841000 4 4 2 23,1 23,1 11,2 16,146 0 10,472 1226200000 49 A0A5G2R7H3;A0A5G2QFJ7;I3LFW6;A0A5G2QXY1;A0A5G2QIZ7;A0A5G2QPC7;A0A5G2RJK 2;A0A287BSL7 A0A5G2R7H3;A0A5G2QFJ7;I3LFW6;A0A5G2QXY1;A0A5G2QIZ7;A0A5G2QPC7;A0A5G2RJK 2;A0A287BSL7 597 tr|A0A5G2R7H3|A0A5G2R7H3_PIG Ubiquitin conjugating enzyme E2 V1 OS=Sus scrofa OX=9823 GN=UBE2V1 PE=1 SV=1;tr|A0A5G2QFJ7|A0A5G2QFJ7_PIG Ubiquitin conjugating enzyme E2 V1 OS=Sus scrofa OX=9823 GN=UBE2V1 PE=1 SV=1;tr|I3LFW6|I3LFW6_PIG Ubiquitin conjugating e

0 0 0 0 0 0 0 0 0 0 0 0 1 1 1 6,4 6,4 6,4 19,166 0 3,4164 117430001 A0A5G2QFL2;A0A5G2QI52 A0A5G2QFL2;A0A5G2QI52 598 tr|A0A5G2QFL2|A0A5G2QFL2_PIG Calcineurin like phosphoesterase domain containing 1 OS=Sus scrofa OX=9823 GN=CPPED1 PE=1 SV=1;tr|A0A5G2QI52|A0A5G2QI52_PIG Calcineurin-like phosphoesterase domain-containing protein 1 OS=Sus scrofa OX=9823 GN=CPPED1 PE=1 SV=1

0 0 0 0 0 0 0 0 0 0 0 0 3 3 3 15,3 15,3 15,3 27,06 0 5,5295 66881000 11 F1RHJ2;A0A5G2QHZ3;A0A5G2QFP2 F1RHJ2;A0A5G2QHZ3;A0A5G2QFP2599 tr|F1RHJ2|F1RHJ2_PIG Heparin binding growth factor OS=Sus scrofa OX=9823 GN=HDGF PE=1 SV=2;tr|A0A5G2QHZ3|A0A5G2QHZ3_PIG Heparin binding growth factor OS=Sus scrofa OX=9823 GN=HDGF PE=1 SV=1;tr|A0A5G2QFP2|A0A5G2QFP2_PIG Heparin binding growth factor OS=Sus

0 0 0 0 0 8924300 0 0 8163800 0 0 0 2 2 2 19,2 19,2 19,2 14,431 0,0021954 2,3328 67600000 6 A0A5G2QFP8;P67985;A0A5G2QW71 A0A5G2QFP8;P67985;A0A5G2QW71 600 tr|A0A5G2QFP8|A0A5G2QFP8_PIG Isoprenylcysteine carboxyl methyltransferase OS=Sus scrofa OX=9823 GN=ICMT PE=1 SV=1;sp|P67985|RL22_PIG 60S ribosomal protein L22 OS=Sus scrofa OX=9823 GN=RPL22 PE=1 SV=2;tr|A0A5G2QW71|A0A5G2QW71_PIG Protein-S-isoprenylcysteine

5166600 8747700 4929200 0 0 0 0 0 0 0 0 0 8 8 8 7,4 7,4 7,4 134,94 0 10,491 76993000 12 F1SQ89;A0A5G2QG48;A0A5G2QRG1 F1SQ89;A0A5G2QG48 601 tr|F1SQ89|F1SQ89_PIG TIP120 domain-containing protein OS=Sus scrofa OX=9823 GN=CAND2 PE=1 SV=1;tr|A0A5G2QG48|A0A5G2QG48_PIG TIP120 domain-containing protein OS=Sus scrofa OX=9823 GN=CAND2 PE=1 SV=1

0 0 2555100 4368400 2507200 3329200 8915100 0 0 4225100 0 0
3 3 3 14,6 14,6 14,6 21,269 0 3,899 66654000 5
A0A5G2QG77;A0A5G2R457;F1S710 A0A5G2QG77;A0A5G2R457;F1S710 602
tr|A0A5G2QG77|A0A5G2QG77_PIG Calcyclin-binding protein OS=Sus scrofa OX=9823 GN=CACYBP PE=1 SV=1;tr|A0A5G2R457|A0A5G2R457_PIG Calcyclin-binding protein OS=Sus scrofa OX=9823 GN=CACYBP PE=1 SV=1;tr|F1S710|F1S710_PIG Calcyclin-binding protein OS=Sus scrofa O
0 7297100 4594800 0 4549700 0 0 0 4996800 0 0 0 14 3
3 19,9 4 4 100,48 0 5,4732 70215000 9
A0A5G2QG93;A0A5G2QLD6;D2WKD6;A0A5G2QZU7;F1SAX3;A0A5G2QRQ1;P05024;A0A5G2R8S7;I3LT88
A0A5G2QG93;A0A5G2QLD6;D2WKD6;A0A5G2QZU7;F1SAX3;A0A5G2QRQ1;P05024;A0A5G2R8S7;I3LT88 603 tr|A0A5G2QG93|A0A5G2QG93_PIG Sodium/potassium-transporting ATPase subunit alpha OS=Sus scrofa OX=9823 GN=ATP1A1 PE=1 SV=1;tr|A0A5G2QLD6|A0A5G2QLD6_PIG Sodium/potassium-transporting ATPase subunit alpha OS=Sus scrofa OX=9823 GN=ATP1A1 PE=1 SV=1;tr|D2WKD6|D2
34633000 27016000 53015000 62126000 61339000 47044000 50391000 32928000 45869000 72452000
59714000 74821000 11 11 11 32,7 32,7 32,7 41,61 0 83,351
3535500000 151 A0A5G2QGS5;I3LP02;A0A286ZXW1;A0A5G2R766
A0A5G2QGS5;I3LP02;A0A286ZXW1 604 tr|A0A5G2QGS5|A0A5G2QGS5_PIG Acetyl-CoA acetyltransferase 1 OS=Sus scrofa OX=9823 GN=ACAT1 PE=1 SV=1;tr|I3LP02|I3LP02_PIG Acetyl-CoA acetyltransferase 1 OS=Sus scrofa OX=9823 GN=ACAT1 PE=1 SV=2;tr|A0A286ZXW1|A0A286ZXW1_PIG Acetyl-CoA acetyltransferase 1 OS
14713000 19319000 12395000 14088000 12296000 12008000 23952000 14322000 18432000 16975000
13521000 9108600 12 12 12 17,9 17,9 17,9 93,763 0 68,898
800370000 85
A0A5G2QUZ9;P43368;A0A5G2QH29;A0A5G2R959;A0A5G2QPB9;A0A5G2QTE5;F1SI23;A0A287BSN0;F1SI20;A0A5G2R032;A0A287AQ25
A0A5G2QUZ9;P43368;A0A5G2QH29;A0A5G2R959;A0A5G2QPB9;A0A5G2QTE5;F1SI23;A0A287BSN0;F1SI20 605 tr|A0A5G2QUZ9|A0A5G2QUZ9_PIG Calpain-3 OS=Sus scrofa OX=9823 GN=CAPN3 PE=3 SV=1;sp|P43368|CAN3_PIG Calpain-3 OS=Sus scrofa OX=9823 GN=CAPN3 PE=2 SV=2;tr|A0A5G2QH29|A0A5G2QH29_PIG Calpain-3 OS=Sus scrofa OX=9823 GN=CAPN3 PE=3 SV=1;tr|A0A5G2R959|A0A5G2R959_P
4004600 25856000 33015000 26223000 23975000 34993000 16331000 29613000 52075000 25904000
29415000 38863000 6 6 6 20,8 20,8 20,8 30,731 0 42,848
936920000 51 F1SE26;A0A5G2QH97;Q29380;F1SE27
F1SE26;A0A5G2QH97;Q29380;F1SE27 606 tr|F1SE26|F1SE26_PIG Voltage-dependent anion-selective channel protein 3 OS=Sus scrofa OX=9823 GN=VDAC3 PE=1 SV=3;tr|A0A5G2QH97|A0A5G2QH97_PIG Voltage-dependent anion-selective channel protein 3 OS=Sus scrofa OX=9823 GN=VDAC3 PE=1 SV=1;sp|Q29380|VDAC3_PIG
0 0 0 0 0 0 0 0 0 0 0 0 1 1 1 6,2 6,2 6,2
18,477 0,0011287 2,5473 4915700 1
A0A5G2QHN7;A0A5S8KXS2;A0A5G2RDZ7;A0A5G2REB0;Q29221;A0A5K1UD05

A0A5G2QHN7;A0A5S8KXS2;A0A5G2RDZ7;A0A5G2REB0;Q29221;A0A5K1UD05 607
tr|A0A5G2QHN7|A0A5G2QHN7_PIG F-actin-capping protein subunit alpha OS=Sus scrofa OX=9823 GN=CAPZA2 PE=1 SV=1;tr|A0A5S8KXS2|A0A5S8KXS2_PIG F-actin-capping protein subunit alpha OS=Sus scrofa OX=9823 GN=CAPZA2 PE=1 SV=1;tr|A0A5G2RDZ7|A0A5G2RDZ7_PIG F-actin-c
0 10321000 9365900 5434100 6390700 7133300 4679300 0 6403200 3532000 4491300
7670700 4 4 4 7,8 7,8 7,8 53,234 0 5,3571 179270000 20
A0A5G2R5Y6;A0A5G2QHR6;I3L9J4;A0A5G2R446;F1RIU3;I3LT97;Q29236
A0A5G2R5Y6;A0A5G2QHR6;I3L9J4;A0A5G2R446;F1RIU3;I3LT97 608
tr|A0A5G2R5Y6|A0A5G2R5Y6_PIG Uncharacterized protein OS=Sus scrofa OX=9823 GN=CCT6A PE=1 SV=1;tr|A0A5G2QHR6|A0A5G2QHR6_PIG Uncharacterized protein OS=Sus scrofa OX=9823 GN=CCT6A PE=1 SV=1;tr|I3L9J4|I3L9J4_PIG Uncharacterized protein OS=Sus scrofa OX=9823 G
19418000 11866000 10920000 27805000 11434000 14575000 26514000 10663000 8453900 22678000
9204600 12585000 7 7 7 25,3 25,3 25,3 30,224 0 15,506
696490000 41 A0A5G2QI19;F2Z5L7;A0A5G2QQW4 A0A5G2QI19;F2Z5L7;A0A5G2QQW4
609 tr|A0A5G2QI19|A0A5G2QI19_PIG Proteasome subunit alpha type OS=Sus scrofa OX=9823 GN=PSMA1 PE=1 SV=1;tr|F2Z5L7|F2Z5L7_PIG Proteasome subunit alpha type OS=Sus scrofa OX=9823 GN=PSMA1 PE=1 SV=1;tr|A0A5G2QQW4|A0A5G2QQW4_PIG Proteasome subunit alpha type OS=Su
0 0 0 0 0 0 0 0 0 0 0 0 2 2 2 4 4 4
50,835 0 3,7709 39561000 2 A0A5G2R2E3;A0A5G2QIC3;I3LRU5;I3LIN5;I3L945
A0A5G2R2E3;A0A5G2QIC3;I3LRU5;I3LIN5;I3L945 610 tr|A0A5G2R2E3|A0A5G2R2E3_PIG Ubiquitin carboxyl-terminal hydrolase OS=Sus scrofa OX=9823 GN=USP14 PE=1 SV=1;tr|A0A5G2QIC3|A0A5G2QIC3_PIG Ubiquitin carboxyl-terminal hydrolase OS=Sus scrofa OX=9823 GN=USP14 PE=1 SV=1;tr|I3LRU5|I3LRU5_PIG Ubiquitin carboxyl-
45463000 31740000 30993000 36358000 14736000 34423000 43971000 21365000 17147000 47088000
20782000 30932000 4 4 4 32,1 32,1 32,1 15,13 0 37,399
1096700000 38 A0A5S6GPG9;A0A5G2QIE9;P50390;A0A5G2R5I8
A0A5S6GPG9;A0A5G2QIE9;P50390;A0A5G2R5I8 611 tr|A0A5S6GPG9|A0A5S6GPG9_PIG Transthyretin OS=Sus scrofa OX=9823 GN=TTR PE=3 SV=1;tr|A0A5G2QIE9|A0A5G2QIE9_PIG Transthyretin OS=Sus scrofa OX=9823 GN=TTR PE=3 SV=1;sp|P50390|TTHY_PIG Transthyretin OS=Sus scrofa OX=9823 GN=TTR PE=1 SV=1;tr|A0A5G2R5I8|A0A5G2
0 0 0 0 0 0 0 0 0 0 0 0 4 3 2 7,1 5,4 4,1
60,569 0,0032086 2,1032 21892000 4
A0A5G2RHH2;A0A5G2QIL8;F1RQR4;A0A287A229
A0A5G2RHH2;A0A5G2QIL8;F1RQR4;A0A287A229 612
tr|A0A5G2RHH2|A0A5G2RHH2_PIG EH domain containing 1 OS=Sus scrofa OX=9823 GN=EHD1 PE=1 SV=1;tr|A0A5G2QIL8|A0A5G2QIL8_PIG EH domain containing 1 OS=Sus scrofa OX=9823 GN=EHD1 PE=1 SV=1;tr|F1RQR4|F1RQR4_PIG EH domain containing 1 OS=Sus scrofa OX=9823 GN=EHD
53220000 29350000 67514000 89289000 52721000 62843000 56971000 31932000 44053000 82511000
48405000 101630000 10 10 10 26,4 26,4 26,4 46,402 0 87,063

4013500000 130 A0A5S6FV10;A0A5G2QYJ3;A0A5G2QIZ1;Q9N0F1;A0A5G2QV56

A0A5S6FV10;A0A5G2QYJ3;A0A5G2QIZ1;Q9N0F1 613 tr|A0A5S6FV10|A0A5S6FV10_PIG Dihydrolipoyllysine-residue succinyltransferase component of 2-oxoglutarate dehydrogenase complex, mitochondrial OS=Sus scrofa OX=9823 GN=DLST PE=3 SV=1;tr|A0A5G2QYJ3|A0A5G2QYJ3_PIG Dihydrolipoyllysine-residue succinyltransfera

0 0 0 0 0 0 0 0 0 0 0 0 1 1 1 9,8 9,8 9,8

10,999 0,0031381 1,9505 33365000 4

F2Z5P1;A0A5G2RBH4;B1PEY3;A0A5G2R318;A0A5G2QJ24;A0A5G2R9I5

F2Z5P1;A0A5G2RBH4;B1PEY3;A0A5G2R318;A0A5G2QJ24;A0A5G2R9I5 614

tr|F2Z5P1|F2Z5P1_PIG Histone H2A OS=Sus scrofa OX=9823 GN=H2AZ2 PE=3 SV=3;tr|A0A5G2RBH4|A0A5G2RBH4_PIG Histone H2A OS=Sus scrofa OX=9823 GN=H2AZ2 PE=3 SV=1;tr|B1PEY3|B1PEY3_PIG Histone H2A OS=Sus scrofa OX=9823 GN=H2A.Z PE=2 SV=1;tr|A0A5G2R318|A0A5G2R318_P

4333400 0 3633000 9766500 0 3652800 8516600 0 4145600 7495500 0 4669600

4 4 4 27,8 27,8 27,8 20,901 0 8,9103 108060000 13

A0A5G2QWD0;A0A5G2QJ69;A0A5G2QW88;I3LC07;A0A5G2RML0;A0A5G2QBQ6;A0A5G2QUG2

A0A5G2QWD0;A0A5G2QJ69;A0A5G2QW88;I3LC07;A0A5G2RML0;A0A5G2QBQ6;A0A5G2QUG2615 tr|A0A5G2QWD0|A0A5G2QWD0_PIG Ras-related protein Rab-18 OS=Sus scrofa OX=9823 GN=RAB18 PE=1 SV=1;tr|A0A5G2QJ69|A0A5G2QJ69_PIG RAB18, member RAS oncogene family OS=Sus scrofa OX=9823 GN=RAB18 PE=1 SV=1;tr|A0A5G2QW88|A0A5G2QW88_PIG RAB18, member RAS oncogene

0 0 0 0 0 0 0 0 0 0 0 0 4 4 4 2,7 2,7 2,7

178,31 0 5,7913 24565000 6

A0A5G2QME3;A0A5G2RG14;I3LTB8;A0A5G2R5F3;F1SBS4;A0A5G2QJA9;P01025;CON__Q2UVX4 A0A5G2QME3;A0A5G2RG14;I3LTB8;A0A5G2R5F3;F1SBS4;A0A5G2QJA9;P01025 616

tr|A0A5G2QME3|A0A5G2QME3_PIG C3-beta-c OS=Sus scrofa OX=9823 GN=C3 PE=1 SV=1;tr|A0A5G2RG14|A0A5G2RG14_PIG C3-beta-c OS=Sus scrofa OX=9823 GN=C3 PE=1 SV=1;tr|I3LTB8|I3LTB8_PIG C3-beta-c OS=Sus scrofa OX=9823 GN=C3 PE=1 SV=2;tr|A0A5G2R5F3|A0A5G2R5F3_PIG C3-b

56531000 43602000 47027000 62940000 47830000 49186000 83479000 43658000 43842000 90622000

42894000 43141000 7 7 7 35,7 35,7 35,7 24,646 0 38,111

1847700000 77 P80895;A0A5G2QK30;J9JIK8;A0A5G2QND0

P80895;A0A5G2QK30;J9JIK8;A0A5G2QND0 618 sp|P80895|PIMT_PIG Protein-L-isoaspartate(D-aspartate) O-methyltransferase OS=Sus scrofa OX=9823 GN=PCMT1 PE=1 SV=3;tr|A0A5G2QK30|A0A5G2QK30_PIG Protein-L-isoaspartate O-methyltransferase OS=Sus scrofa OX=9823 GN=PCMT1 PE=3 SV=1;tr|J9JIK8|J9JIK8_PIG Protei

20065000 33383000 44625000 14139000 43872000 30429000 19434000 46131000 75850000 29386000

27625000 48854000 11 11 11 17,7 17,7 17,7 68,251 0 27,109

1833000000 80

A0A5G2QK83;A0A5G2QLQ1;A0A5G2R3I6;A0A5G2QFC4;A0A5G2QIE6;F1SMB2;A0A5G2R750

A0A5G2QK83;A0A5G2QLQ1;A0A5G2R3I6;A0A5G2QFC4;A0A5G2QIE6;F1SMB2;A0A5G2R75

0 619 tr|A0A5G2QK83|A0A5G2QK83_PIG Acetyltransferase component of pyruvate dehydrogenase complex OS=Sus scrofa OX=9823 GN=DLAT PE=1 SV=1;tr|A0A5G2QLQ1|A0A5G2QLQ1_PIG Acetyltransferase component of pyruvate dehydrogenase complex OS=Sus scrofa OX=9823 GN=DLAT PE=1
0 0 6802400 7857800 0 9653100 9560900 0 6325500 8784800 0 6571600
4 4 4 16,6 16,6 16,6 30,633 0 5,8803 141580000 17
F1SUE3;A0A5G2QTT3;A0A5G2QMG2;A0A5G2QKC9;A0A5G2QU23
F1SUE3;A0A5G2QTT3;A0A5G2QMG2;A0A5G2QKC9;A0A5G2QU23 620
tr|F1SUE3|F1SUE3_PIG Inorganic diphosphatase OS=Sus scrofa OX=9823 GN=PPA1 PE=1 SV=3;tr|A0A5G2QTT3|A0A5G2QTT3_PIG Inorganic diphosphatase OS=Sus scrofa OX=9823 GN=PPA1 PE=1 SV=1;tr|A0A5G2QMG2|A0A5G2QMG2_PIG Inorganic diphosphatase OS=Sus scrofa OX=9823 GN=
58139000 52776000 69421000 136740000 92713000 98401000 90950000 69196000 90171000
105300000 93692000 117460000 6 6 6 23,8 23,8 23,8 28,466 0
17,269 2979100000 89 A0A5G2QKD9;F1S418 A0A5G2QKD9;F1S418 621
tr|A0A5G2QKD9|A0A5G2QKD9_PIG Peroxiredoxin 3 OS=Sus scrofa OX=9823 GN=PRDX3 PE=1 SV=1;tr|F1S418|F1S418_PIG Peroxiredoxin 3 OS=Sus scrofa OX=9823 GN=PRDX3 PE=1 SV=1
0 0 7986400 8229000 8954900 8694100 7829900 0 8853200 12111000 0 0
3 3 3 16,7 16,7 16,7 19,339 0 3,9585 139280000 8
A0A5G2QKN3;A0A5G2RHC5;A0A5K1UWY1;K7GQV5;A0A5G2R9S6;A0A5G2R0R7;F1S2N0
A0A5G2QKN3;A0A5G2RHC5;A0A5K1UWY1;K7GQV5;A0A5G2R9S6;A0A5G2R0R7;F1S2N0
622 tr|A0A5G2QKN3|A0A5G2QKN3_PIG Glutathione S-transferase zeta 1 OS=Sus scrofa OX=9823 GN=GSTZ1 PE=1 SV=1;tr|A0A5G2RHC5|A0A5G2RHC5_PIG Maleylacetoacetate isomerase OS=Sus scrofa OX=9823 GN=GSTZ1 PE=1 SV=1;tr|A0A5K1UWY1|A0A5K1UWY1_PIG Maleylacetoacetate isomer
21207000 10993000 8495900 11521000 10535000 9057300 10157000 0 0 6652100 0 9694300
4 4 4 13,5 13,5 13,5 29,579 0 5,2342 223630000 8
A0A5G2QKS9;F1RRY2;I3LUP6 A0A5G2QKS9;F1RRY2;I3LUP6 623
tr|A0A5G2QKS9|A0A5G2QKS9_PIG Nucleophosmin OS=Sus scrofa OX=9823 GN=NPM1 PE=1 SV=1;tr|F1RRY2|F1RRY2_PIG Nucleoplasmin domain-containing protein OS=Sus scrofa OX=9823 GN=NPM1 PE=1 SV=4;tr|I3LUP6|I3LUP6_PIG Nucleophosmin OS=Sus scrofa OX=9823 GN=NPM1 PE=1 SV
0 0 0 0 0 0 0 0 0 0 0 0 2 2 2 4,3 4,3 4,3
46,153 0,0011534 2,7627 26225000 3
A0A5G2QKT7;A0A5K1VGL1;F1RUJ0;A0A5G2R676;A0A5G2QDC7
A0A5G2QKT7;A0A5K1VGL1;F1RUJ0;A0A5G2R676;A0A5G2QDC7 624
tr|A0A5G2QKT7|A0A5G2QKT7_PIG Adenylyl cyclase-associated protein OS=Sus scrofa OX=9823 GN=CAP2 PE=1 SV=1;tr|A0A5K1VGL1|A0A5K1VGL1_PIG Adenylyl cyclase-associated protein OS=Sus scrofa OX=9823 GN=CAP2 PE=1 SV=1;tr|F1RUJ0|F1RUJ0_PIG Adenylyl cyclase-associat
22516000 11453000 15423000 5009300 19350000 8053700 13582000 0 9025400 7615300 7010600
20361000 4 4 0 20,7 20,7 0 37,106 0 16,044 253090000 33

A0A5G2QKX2 A0A5G2QKX2 626 tr|A0A5G2QKX2|A0A5G2QKX2_PIG Ankyrin repeat domain 2 OS=Sus scrofa OX=9823 GN=ANKRD2 PE=1 SV=1
15502000 17799000 12499000 21216000 11604000 17149000 26031000 11579000 15278000 13525000 18211000 15578000 6 6 6 19,5 19,5 19,5 29,483 0 6,4053 520860000 25 A0A5G2R1I2;A0A5G2QL81;A0A5G2QYN2;A0A5G2QCU1;A0A287BIV4 A0A5G2R1I2;A0A5G2QL81;A0A5G2QYN2;A0A5G2QCU1 627 tr|A0A5G2R1I2|A0A5G2R1I2_PIG Proteasome subunit alpha type OS=Sus scrofa OX=9823 GN=PSMA4 PE=1 SV=1;tr|A0A5G2QL81|A0A5G2QL81_PIG Proteasome subunit alpha type OS=Sus scrofa OX=9823 GN=PSMA4 PE=1 SV=1;tr|A0A5G2QYN2|A0A5G2QYN2_PIG Proteasome subunit beta OS=
0 0 0 0 0 0 0 0 0 0 0 0 3 3 3 42,3 42,3 42,3 12,504 0 28,125 72453000 9 F2Z553;A0A5G2QLD8;P61220 F2Z553;A0A5G2QLD8;P61220 628 tr|F2Z553|F2Z553_PIG Eukaryotic translation initiation factor 1 OS=Sus scrofa OX=9823 GN=EIF1 PE=3 SV=2;tr|A0A5G2QLD8|A0A5G2QLD8_PIG Eukaryotic translation initiation factor 1 OS=Sus scrofa OX=9823 GN=EIF1 PE=1 SV=1;sp|P61220|EIF1B_PIG Eukaryotic translati
0 0 0 0 0 0 0 0 0 0 0 0 2 2 2 9,5 9,5 9,5 21,29 0,0021882 2,291 29426000 3 A0A5G2QLU1;P27917;A0A5S6HRA3 A0A5G2QLU1;P27917;A0A5S6HRA3 629 tr|A0A5G2QLU1|A0A5G2QLU1_PIG Apolipoprotein C-III OS=Sus scrofa OX=9823 GN=APOC3 PE=1 SV=1;sp|P27917|APOC3_PIG Apolipoprotein C-III OS=Sus scrofa OX=9823 GN=APOC3 PE=1 SV=2;tr|A0A5S6HRA3|A0A5S6HRA3_PIG Apolipoprotein C-III OS=Sus scrofa OX=9823 GN=APOC3 PE
29808000 0 40066000 3106100 15991000 10054000 11882000 33751000 7767400 18416000 37338000 32797000 16 16 16 31 31 31 60,046 0 77,718 1864100000 90 A0A5G2QM05 A0A5G2QM05 630 tr|A0A5G2QM05|A0A5G2QM05_PIG Uncharacterized protein OS=Sus scrofa OX=9823 PE=4 SV=1
30660000 33790000 59011000 55852000 54500000 48944000 58147000 26413000 40598000 67411000 48797000 73033000 13 13 13 33,4 33,4 33,4 40,257 0 37,46 4742000000 163 A0A5G2QMB8;I3LCI2;P29804;A0A5K1VBH1;A0A5G2RNK8 A0A5G2QMB8;I3LCI2;P29804;A0A5K1VBH1 632 tr|A0A5G2QMB8|A0A5G2QMB8_PIG Pyruvate dehydrogenase E1 component subunit alpha, somatic form, mitochondrial OS=Sus scrofa OX=9823 GN=PDHA1 PE=1 SV=1;tr|I3LCI2|I3LCI2_PIG Pyruvate dehydrogenase E1 component subunit alpha OS=Sus scrofa OX=9823 GN=PDHA1 PE=1
10418000 11947000 0 0 11310000 0 8698000 0 0 0 0 0 2 2 2 3,7 3,7 3,7 56,662 0 4,2983 117560000 17 A0A5G2R9E6;A0A5G2QMC1;C6K7I1;A0A5G2R9L2;A0A287ATD3 A0A5G2R9E6;A0A5G2QMC1;C6K7I1;A0A5G2R9L2;A0A287ATD3 633 tr|A0A5G2R9E6|A0A5G2R9E6_PIG Importin subunit alpha OS=Sus scrofa OX=9823 GN=KPNA3 PE=1 SV=1;tr|A0A5G2QMC1|A0A5G2QMC1_PIG Importin subunit alpha OS=Sus scrofa OX=9823 GN=KPNA3 PE=1 SV=1;tr|C6K7I1|C6K7I1_PIG Importin subunit alpha OS=Sus scrofa OX=9823 GN=K

0 0 0 0 0 0 0 0 0 0 0 0 22 1 1 56,3 4,6 4,6
32,8 0 3,2474 13342000 3 F1SG00;A0A5G2QMK3;A0A287AD38
F1SG00;A0A5G2QMK3;A0A287AD38 634 tr|F1SG00|F1SG00_PIG Tropomyosin 2 OS=Sus scrofa OX=9823 GN=TPM2 PE=1 SV=2;tr|A0A5G2QMK3|A0A5G2QMK3_PIG Tropomyosin 2 OS=Sus scrofa OX=9823 GN=TPM2 PE=1 SV=1;tr|A0A287AD38|A0A287AD38_PIG Tropomyosin 2 OS=Sus scrofa OX=9823 GN=TPM2 PE=1 SV=2
103230000 10394000 34676000 35521000 13364000 53047000 26489000 12077000 26409000 55328000
16760000 34919000 7 7 4 22,7 22,7 13 33,483 0 11,566
1709100000 51
A0A5G2QML3;F2Z584;A0A287BID4;A0A287A7G8;I3LAJ9;A0A287AWZ3;F2Z580;F1RTQ6;A0A5G2RG11;A0A5G2R2P2;A0A5G2QI50;F2Z581;A0A287ARA0;F1RFJ3
A0A5G2QML3;F2Z584;A0A287BID4;A0A287A7G8;I3LAJ9 635
tr|A0A5G2QML3|A0A5G2QML3_PIG Histone H2B OS=Sus scrofa OX=9823 GN=H1-3 PE=1 SV=1;tr|F2Z584|F2Z584_PIG Histone H2B OS=Sus scrofa OX=9823 GN=HIST1H2BD PE=3 SV=1;tr|A0A287BID4|A0A287BID4_PIG Histone H2B OS=Sus scrofa OX=9823 GN=H2BC21 PE=3 SV=1;tr|A0A287A7G8|
54841000 34838000 35414000 44047000 20485000 30826000 53926000 22060000 42666000 63216000
27892000 34659000 10 10 10 22,9 22,9 22,9 54,377 0 58,326
2296100000 103 A0A5G2QN66;G9F6X8;A0A5G2RDD7;A0A287AQH8
A0A5G2QN66;G9F6X8;A0A5G2RDD7;A0A287AQH8 636
tr|A0A5G2QN66|A0A5G2QN66_PIG Protein disulfide-isomerase OS=Sus scrofa OX=9823 GN=P4HB PE=3 SV=1;tr|G9F6X8|G9F6X8_PIG Protein disulfide-isomerase OS=Sus scrofa OX=9823 GN=P4HB PE=2 SV=1;tr|A0A5G2RDD7|A0A5G2RDD7_PIG Protein disulfide-isomerase OS=Sus scrofa
0 0 0 0 0 0 0 0 0 0 0 0 4 4 4 6,7 6,7 6,7
61,73 0 3,7284 47413000 8 I3LPT1;A0A5G2RF77;A0A5G2QNL3
I3LPT1;A0A5G2RF77;A0A5G2QNL3 637 tr|I3LPT1|I3LPT1_PIG Transketolase OS=Sus scrofa OX=9823 GN=TKT PE=1 SV=1;tr|A0A5G2RF77|A0A5G2RF77_PIG Transketolase OS=Sus scrofa OX=9823 GN=TKT PE=1 SV=1;tr|A0A5G2QNL3|A0A5G2QNL3_PIG Transketolase OS=Sus scrofa OX=9823 GN=TKT PE=1 SV=1
43656000 38654000 45721000 14403000 11834000 7371200 23522000 16139000 0 19355000 14131000
13895000 9 9 9 18,7 18,7 18,7 66,576 0 27,782 861770000 43
F1S7H3;A0A5G2QNQ1 F1S7H3;A0A5G2QNQ1 638 tr|F1S7H3|F1S7H3_PIG Myosin light chain kinase 2, skeletal/cardiac muscle OS=Sus scrofa OX=9823 GN=MYLK2 PE=1 SV=3;tr|A0A5G2QNQ1|A0A5G2QNQ1_PIG Myosin light chain kinase 2, skeletal/cardiac muscle OS=Sus scrofa OX=9823 GN=MYLK2 PE=1 SV=1
0 0 3983800 5226000 0 0 5418600 0 0 6736200 0 3321000 4
4 4 24,4 24,4 24,4 18,69 0 6,0171 77985000 15
A0A5G2QNT1;K7GKM7;K7GNY9;F1RZQ7;A0A5G2QMU1
A0A5G2QNT1;K7GKM7;K7GNY9;F1RZQ7;A0A5G2QMU1 639
tr|A0A5G2QNT1|A0A5G2QNT1_PIG Prefoldin subunit 3 OS=Sus scrofa OX=9823 GN=VBP1 PE=1 SV=1;tr|K7GKM7|K7GKM7_PIG Prefoldin subunit 3 OS=Sus scrofa OX=9823 GN=VBP1 PE=1

SV=3;tr|K7GNY9|K7GNY9_PIG Uncharacterized protein OS=Sus scrofa OX=9823 GN=VBP1 PE=1 SV=2;t
14409000 10541000 9672200 0 5573700 0 5689000 0 0 6093600 0 0
5 5 5 22,7 22,7 22,7 29,222 0 12,436 130340000 17
F1RLB5;F1RLB6;A0A5G2QQW2;A0A5G2QNT7
F1RLB5;F1RLB6;A0A5G2QQW2;A0A5G2QNT7 640 tr|F1RLB5|F1RLB5_PIG NSF attachment protein alpha OS=Sus scrofa OX=9823 GN=NAPA PE=1 SV=4;tr|F1RLB6|F1RLB6_PIG NSF attachment protein alpha OS=Sus scrofa OX=9823 GN=NAPA PE=1 SV=3;tr|A0A5G2QQW2|A0A5G2QQW2_PIG Alpha-soluble NSF attachment protein OS=Sus scr
0 0 0 0 0 0 12498000 0 7032700 14048000 11015000 0 3 3
3 10 10 10 42,971 0 5,9967 133690000 10 A0A5G2QNW1;F1S827
A0A5G2QNW1;F1S827 641 tr|A0A5G2QNW1|A0A5G2QNW1_PIG SERPINE1 mRNA binding protein 1 OS=Sus scrofa OX=9823 GN=SERBP1 PE=1 SV=1;tr|F1S827|F1S827_PIG Plasminogen activator inhibitor 1 RNA-binding protein isoform 1 OS=Sus scrofa OX=9823 GN=SERBP1 PE=1 SV=2
157710000 77826000 213570000 330430000 197360000 199250000 265820000
87111000 176930000 444880000 188020000 325480000 8 8 8
42 42 42 19,64 0 44,813 9579300000 184
I3LER5;A0A5G2QPD2;Q95283;A0A5G2QNW4;A0A5G2R611
I3LER5;A0A5G2QPD2;Q95283;A0A5G2QNW4;A0A5G2R611 642 tr|I3LER5|I3LER5_PIG Cytochrome c oxidase subunit 4 OS=Sus scrofa OX=9823 GN=COX4I1 PE=1 SV=2;tr|A0A5G2QPD2|A0A5G2QPD2_PIG Cytochrome c oxidase subunit 4 OS=Sus scrofa OX=9823 GN=COX4I1 PE=1 SV=1;sp|Q95283|COX41_PIG Cytochrome c oxidase subunit 4 isoform 1
20127000 15646000 9951400 25006000 13073000 16618000 27199000 9773900 10343000 17806000
11879000 12056000 3 3 3 10,5 10,5 10,5 29,716 0 3,0579
369360000 13 A0A5G2QPJ2;A1XQU1 A0A5G2QPJ2;A1XQU1 643
tr|A0A5G2QPJ2|A0A5G2QPJ2_PIG Proteasome subunit beta OS=Sus scrofa OX=9823 GN=PSMB7 PE=1 SV=1;sp|A1XQU1|PSB7_PIG Proteasome subunit beta type-7 OS=Sus scrofa OX=9823 GN=PSMB7 PE=2 SV=2
0 5741300 14744000 10095000 11455000 10799000 5868500 7506500 5255200 8379800 12198000
23312000 7 7 7 35,7 35,7 35,7 25,593 0 15,641 344310000 21
A0A5G2QPK4;F1SK45;A0A5G2QI32 A0A5G2QPK4;F1SK45;A0A5G2QI32 644
tr|A0A5G2QPK4|A0A5G2QPK4_PIG GTP:AMP phosphotransferase AK3, mitochondrial OS=Sus scrofa OX=9823 GN=AK3 PE=1 SV=1;tr|F1SK45|F1SK45_PIG Nucleoside-diphosphate kinase OS=Sus scrofa OX=9823 GN=AK3 PE=1 SV=2;tr|A0A5G2QI32|A0A5G2QI32_PIG Nucleoside-diphosphate
45952000 57910000 97138000 108610000 101620000 104970000 85644000 50410000 77706000
94177000 90677000 140390000 7 7 7 15,2 15,2 15,2 53,296 0
92,293 3936300000 114
A0A5G2QPK6;P09623;F1SAF0;A0A5G2QXX6;A0A480N1D9;A0A5G2QGG6;A0A5G2QH34
A0A5G2QPK6;P09623;F1SAF0;A0A5G2QXX6;A0A480N1D9;A0A5G2QGG6;A0A5G2QH34
645 tr|A0A5G2QPK6|A0A5G2QPK6_PIG Dihydrolipoyl dehydrogenase OS=Sus scrofa OX=9823 GN=DLD PE=1 SV=1;sp|P09623|DLDH_PIG Dihydrolipoyl dehydrogenase, mitochondrial

OS=Sus scrofa OX=9823 GN=DLD PE=1 SV=1;tr|F1SAF0|F1SAF0_PIG Dihydrolipoyl dehydrogenase OS=Sus scr
0 0 6638500 0 7682300 7702100 0 0 7612000 5581300 0 9956800
3 3 3 16,4 16,4 16,4 24,491 0 4,9043 95534000 6
K7GP05;F1S1Q7;A0A5G2R976;A0A5G2RHS1;A0A5G2QPN0;A0A5G2QZI2
K7GP05;F1S1Q7;A0A5G2R976;A0A5G2RHS1;A0A5G2QPN0;A0A5G2QZI2 646
tr|K7GP05|K7GP05_PIG MICOS complex subunit OS=Sus scrofa OX=9823 GN=APOOL PE=1 SV=2;tr|F1S1Q7|F1S1Q7_PIG MICOS complex subunit OS=Sus scrofa OX=9823 GN=APOOL PE=1 SV=3;tr|A0A5G2R976|A0A5G2R976_PIG MICOS complex subunit OS=Sus scrofa OX=9823 GN=APOOL PE=1 S
82258000 73521000 57120000 88749000 57802000 75013000 93395000 48564000 72322000 93425000
62197000 54896000 26 26 26 13,4 13,4 13,4 239,15 0 67,003
2711800000 174
A0A5G2QPQ0;F1SLE7;A0A5G2QZY1;A0A5G2R7H9;A0A5G2QU72;A0A5G2RDH2;A0A5G2R9Y7;A0A5G2QS33;F1SC82;A0A287B6F4;A0A5G2Q8T2;A0A287AHC3;A0A287BPY3
A0A5G2QPQ0;F1SLE7;A0A5G2QZY1;A0A5G2R7H9;A0A5G2QU72 647
tr|A0A5G2QPQ0|A0A5G2QPQ0_PIG Dysferlin OS=Sus scrofa OX=9823 GN=DYSF PE=1 SV=1;tr|F1SLE7|F1SLE7_PIG Dysferlin OS=Sus scrofa OX=9823 GN=DYSF PE=1 SV=4;tr|A0A5G2QZY1|A0A5G2QZY1_PIG Dysferlin OS=Sus scrofa OX=9823 GN=DYSF PE=1 SV=1;tr|A0A5G2R7H9|A0A5G2R7H9_PI
32239000 0 24057000 57143000 23786000 0 47094000 0 24325000 72313000 27915000 0
3 3 1 13,3 13,3 3,4 39,955 0 17,893 504180000 24 A0A5G2QR28
A0A5G2QR28648 tr|A0A5G2QR28|A0A5G2QR28_PIG 3-hydroxyisobutyrate dehydrogenase, mitochondrial OS=Sus scrofa OX=9823 GN=HIBADH PE=1 SV=1
0 0 0 0 0 0 0 0 0 0 0 0 2 2 2 7,3 7,3 7,3
32,724 0 5,4249 37509000 3 A0A5G2QRL2;F1SC51 A0A5G2QRL2;F1SC51 649
tr|A0A5G2QRL2|A0A5G2QRL2_PIG PDZ and LIM domain 1 OS=Sus scrofa OX=9823 GN=PDLIM1 PE=1 SV=1;tr|F1SC51|F1SC51_PIG PDZ and LIM domain 1 OS=Sus scrofa OX=9823 GN=PDLIM1 PE=1 SV=4
0 0 13732000 0 0 0 0 0 4292400 0 0 0 2 2 2 11,7
11,7 11,7 24,859 0 9,0236 53572000 4 Q6SEG5;A0A5G2QRS7;A0A5G2RGW1
Q6SEG5;A0A5G2QRS7;A0A5G2RGW1 650 sp|Q6SEG5|UCHL1_PIG Ubiquitin carboxyl-terminal hydrolase isozyme L1 OS=Sus scrofa OX=9823 GN=UCHL1 PE=2 SV=1;tr|A0A5G2QRS7|A0A5G2QRS7_PIG Ubiquitin carboxyl-terminal hydrolase OS=Sus scrofa OX=9823 GN=UCHL1 PE=1 SV=1;tr|A0A5G2RGW1|A0A5G2RGW1_PIG Ubiquitin
17438000 0 0 20141000 0 13283000 17606000 0 9896800 19826000 0 13182000
3 3 3 7,7 7,7 7,7 68,001 0 32,679 350650000 32 A0A5G2QRX8;F1RIN7
A0A5G2QRX8;F1RIN7 651 tr|A0A5G2QRX8|A0A5G2QRX8_PIG Histidine rich calcium binding protein OS=Sus scrofa OX=9823 GN=HRC PE=1 SV=1;tr|F1RIN7|F1RIN7_PIG Histidine rich calcium binding protein OS=Sus scrofa OX=9823 GN=HRC PE=1 SV=2
54220000 24460000 57601000 89495000 55387000 61926000 93376000 31360000 44353000 127810000
48000000 90252000 7 7 7 21,2 21,2 21,2 43,972 0 76,222
3217000000 130 A0A5G2QS58;A0A287AT07;I3LHS7;A0A5G2QGD9;F6PTP3

A0A5G2QS58;A0A287AT07;I3LHS7;A0A5G2QGD9 652 tr|A0A5G2QS58|A0A5G2QS58_PIG Ubiquinone biosynthesis protein OS=Sus scrofa OX=9823 GN=COQ9 PE=1 SV=1;tr|A0A287AT07|A0A287AT07_PIG Ubiquinone biosynthesis protein OS=Sus scrofa OX=9823 GN=COQ9 PE=1 SV=1;tr|I3LHS7|I3LHS7_PIG Ubiquinone biosynthesis protein O
6660600 10396000 18546000 6505300 15567000 11099000 8329500 8148000 15922000 8757200
10612000 15540000 6 6 2 12,2 12,2 4,8 51,179 0 8,0881
377170000 35 F1SGT3;A0A5G2QSG9 F1SGT3;A0A5G2QSG9 653
tr|F1SGT3|F1SGT3_PIG Dihydrolipoamide acetyltransferase component of pyruvate dehydrogenase complex OS=Sus scrofa OX=9823 GN=PDHX PE=1 SV=2;tr|A0A5G2QSG9|A0A5G2QSG9_PIG Dihydrolipoamide acetyltransferase component of pyruvate dehydrogenase complex OS=Sus s
0 0 0 0 0 0 0 0 0 0 0 0 4 4 4 12,6 12,6 12,6
43,843 0 19,811 61617000 20 A0A5G2QST4;D0G6X4 A0A5G2QST4;D0G6X4 654
tr|A0A5G2QST4|A0A5G2QST4_PIG Farnesyl diphosphate synthase OS=Sus scrofa OX=9823 GN=FDPS PE=1 SV=1;tr|D0G6X4|D0G6X4_PIG Farnesyl diphosphate synthase OS=Sus scrofa OX=9823 GN=FDPS PE=1 SV=1
48361000 33137000 70848000 78788000 62489000 49213000 79265000 36194000 44684000 99414000
56490000 94015000 10 10 10 34,3 34,3 34,3 37,128 0 76,359
3636300000 147 A0A5G2R718;F1SGH5;A0A5G2QSU5 A0A5G2R718;F1SGH5;A0A5G2QSU5
655 tr|A0A5G2R718|A0A5G2R718_PIG Pyruvate dehydrogenase E1 component subunit beta OS=Sus scrofa OX=9823 GN=PDHB PE=1 SV=1;tr|F1SGH5|F1SGH5_PIG Pyruvate dehydrogenase E1 component subunit beta OS=Sus scrofa OX=9823 GN=PDHB PE=1 SV=1;tr|A0A5G2QSU5|A0A5G2QSU5_PIG
4504500 6097700 5500000 0 0 0 0 0 0 0 0 0 5 5 5
4,7 4,7 4,7 141,76 0 10,038 33294000 6
A0A5G2R988;A0A5G2QY27;Q5GN48;I3LG08;A0A5G2QTV4;F1SPU7;A0A286ZSQ2;I3LPH8
A0A5G2R988;A0A5G2QY27;Q5GN48;I3LG08;A0A5G2QTV4;F1SPU7;A0A286ZSQ2 656
tr|A0A5G2R988|A0A5G2R988_PIG Dystrophin OS=Sus scrofa OX=9823 GN=DMD PE=4 SV=1;tr|A0A5G2QY27|A0A5G2QY27_PIG Dystrophin OS=Sus scrofa OX=9823 GN=DMD PE=4 SV=1;sp|Q5GN48|DMD_PIG Dystrophin OS=Sus scrofa OX=9823 GN=DMD PE=1 SV=1;tr|I3LG08|I3LG08_PIG Dystrophi
0 0 0 0 0 0 0 0 0 0 0 0 2 2 2 5,8 5,8 5,8
47,208 0,0022272 2,4308 23649000 6 F2Z5E2;A0A5G2QUE0 F2Z5E2;A0A5G2QUE0
657 tr|F2Z5E2|F2Z5E2_PIG Serpin family C member 1 OS=Sus scrofa OX=9823 GN=SERPINC1 PE=1 SV=2;tr|A0A5G2QUE0|A0A5G2QUE0_PIG Antithrombin-III OS=Sus scrofa OX=9823 GN=SERPINC1 PE=1 SV=1
0 8539300 10655000 7102100 7788800 15108000 5320600 5904200 10773000 5978900 8565100
10450000 6 6 6 25,9 25,9 25,9 26,688 0 8,4618 324110000 21
Q0Z8U2;A0A5G2QUQ6;A0A5G2QSB4;A0A5S6H050
Q0Z8U2;A0A5G2QUQ6;A0A5G2QSB4;A0A5S6H050 658 sp|Q0Z8U2|RS3_PIG 40S ribosomal protein S3 OS=Sus scrofa OX=9823 GN=RPS3 PE=1 SV=1;tr|A0A5G2QUQ6|A0A5G2QUQ6_PIG 40S ribosomal protein S3 OS=Sus scrofa OX=9823

GN=RPS3 PE=1 SV=1;tr|A0A5G2QSB4|A0A5G2QSB4_PIG 40S ribosomal protein S3 OS=Sus scrofa OX=9823 GN=
23875000 17134000 12544000 29742000 16489000 17763000 37551000 15033000 18619000 29025000 0
0 4 4 4 23,4 23,4 23,4 17,861 0 7,1991 459670000 19
B8Y648;A0A5G2RDX3;A0A5G2QUT6 B8Y648;A0A5G2RDX3;A0A5G2QUT6 659
tr|B8Y648|B8Y648_PIG Ubiquitin-conjugating enzyme E2L 3 OS=Sus scrofa OX=9823 GN=UBE2L3 PE=1 SV=1;tr|A0A5G2RDX3|A0A5G2RDX3_PIG UBC core domain-containing protein OS=Sus scrofa OX=9823 GN=UBE2L3 PE=1 SV=1;tr|A0A5G2QUT6|A0A5G2QUT6_PIG UBC core domain-contain
0 0 66570000 13320000 67503000 98056000 0 23917000 10021000 0 43543000 37953000
10 10 9 42,9 42,9 37,1 24,026 0 35,43 1003800000 49
F1SM01;A0A5G2QUW6;F1SLZ3;A0A5G2QF69 F1SM01;A0A5G2QUW6;F1SLZ3;A0A5G2QF69
660 tr|F1SM01|F1SM01_PIG Uncharacterized protein OS=Sus scrofa OX=9823 GN=MYL6B PE=1 SV=1;tr|A0A5G2QUW6|A0A5G2QUW6_PIG Uncharacterized protein OS=Sus scrofa OX=9823 GN=MYL6B PE=1 SV=1;tr|F1SLZ3|F1SLZ3_PIG Uncharacterized protein OS=Sus scrofa OX=9823 GN=MYL6B
0 0 0 0 0 0 0 0 0 0 0 0 1 1 1 6,4 6,4 6,4
19,663 0,0087041 1,4182 16795000 1 A0A5G2QVD3;F1SKI6 A0A5G2QVD3;F1SKI6
661 tr|A0A5G2QVD3|A0A5G2QVD3_PIG NADH:ubiquinone oxidoreductase complex assembly factor 3 OS=Sus scrofa OX=9823 GN=NDUFAF3 PE=1 SV=1;tr|F1SKI6|F1SKI6_PIG NADH dehydrogenase [ubiquinone] 1 alpha subcomplex assembly factor 3 OS=Sus scrofa OX=9823 GN=NDUFAF3 PE=1
0 0 3007000 0 0 2934300 0 0 5197700 0 0 0 2 2 2
15,1 15,1 15,1 17,992 0 3,6813 31703000 6
A0A5G2R5R3;A0A5G2QVU1;Q95342;A0A5S8L0N9;A0A5G2QVW0
A0A5G2R5R3;A0A5G2QVU1;Q95342;A0A5S8L0N9;A0A5G2QVW0 663
tr|A0A5G2R5R3|A0A5G2R5R3_PIG 60S ribosomal protein L18 OS=Sus scrofa OX=9823 GN=RPL18 PE=1 SV=1;tr|A0A5G2QVU1|A0A5G2QVU1_PIG 60S ribosomal protein L18 OS=Sus scrofa OX=9823 GN=RPL18 PE=1 SV=1;sp|Q95342|RL18_PIG 60S ribosomal protein L18 OS=Sus scrofa OX=98
0 7354400 0 6738900 9999000 7424600 9047100 0 7491000 6562100 0 0
5 5 5 8,8 8,8 8,8 53,841 0 6,2138 178870000 9
A0A5G2R4N8;I3LR32;A0A5G2QVV0 A0A5G2R4N8;I3LR32;A0A5G2QVV0 664
tr|A0A5G2R4N8|A0A5G2R4N8_PIG Chaperonin containing TCP1 subunit 5 OS=Sus scrofa OX=9823 GN=CCT5 PE=1 SV=1;tr|I3LR32|I3LR32_PIG CCT-epsilon OS=Sus scrofa OX=9823 GN=CCT5 PE=1 SV=2;tr|A0A5G2QVV0|A0A5G2QVV0_PIG CCT-epsilon OS=Sus scrofa OX=9823 GN=CCT5 PE=1 S
0 0 0 0 0 0 0 0 0 0 0 0 2 1 1 7,3 4,6 4,6
44,939 0 8,1757 58445000 6
I3LFV4;A0A5G2QVX8;F1RWB7;A0A287AJD3;A0A5G2QN07;F1SFU0
I3LFV4;A0A5G2QVX8;F1RWB7;A0A287AJD3;A0A5G2QN07;F1SFU0 665
tr|I3LFV4|I3LFV4_PIG Y-box binding protein 1 OS=Sus scrofa OX=9823 GN=YBX1 PE=1 SV=3;tr|A0A5G2QVX8|A0A5G2QVX8_PIG Y-box binding protein 1 OS=Sus scrofa OX=9823

GN=YBX1 PE=1 SV=1;tr|F1RWB7|F1RWB7_PIG CSD domain-containing protein OS=Sus scrofa OX=9823 PE=4
9343300 0 5602800 0 0 9681200 0 0 0 0 0 8426600 4 4
4 4,9 4,9 4,9 112,62 0 5,8099 97727000 10
K7GQB8;A0A5G2QW05;K7GMZ8;F1SKB1;K7GKN3
K7GQB8;A0A5G2QW05;K7GMZ8;F1SKB1;K7GKN3 666 tr|K7GQB8|K7GQB8_PIG Uncharacterized protein OS=Sus scrofa OX=9823 GN=CP PE=1 SV=2;tr|A0A5G2QW05|A0A5G2QW05_PIG Uncharacterized protein OS=Sus scrofa OX=9823 GN=CP PE=1 SV=1;tr|K7GMZ8|K7GMZ8_PIG Uncharacterized protein OS=Sus scrofa OX=9823 GN=CP PE=1 SV=3
0 0 0 0 0 0 0 0 0 0 0 0 5 1 0 46 9,7 0
14,041 0,008629 1,3882 6646200 3 A0A5G2QWP1 A0A5G2QWP1 667
tr|A0A5G2QWP1|A0A5G2QWP1_PIG Programmed cell death 5 OS=Sus scrofa OX=9823 GN=PDCD5 PE=1 SV=1
0 0 0 0 0 0 0 0 0 0 0 0 3 3 3 11,3 11,3 11,3
43,534 0 16,148 26524000 4 A0A5G2QWP7;A0A5G2R8S6;F1SSR4
A0A5G2QWP7;A0A5G2R8S6;F1SSR4 668 tr|A0A5G2QWP7|A0A5G2QWP7_PIG Butyryl-CoA dehydrogenase OS=Sus scrofa OX=9823 GN=IVD PE=1 SV=1;tr|A0A5G2R8S6|A0A5G2R8S6_PIG Butyryl-CoA dehydrogenase OS=Sus scrofa OX=9823 GN=IVD PE=1 SV=1;tr|F1SSR4|F1SSR4_PIG Butyryl-CoA dehydrogenase OS=Sus scrofa OX=9823
0 0 0 0 0 0 0 0 0 0 0 0 2 2 2 20 20 20
15,336 0,0030928 1,864 19298000 5 A0A5G2QWQ4;I3LE14;A0A5G2R9I4
A0A5G2QWQ4;I3LE14;A0A5G2R9I4 669 tr|A0A5G2QWQ4|A0A5G2QWQ4_PIG Coiled-coil domain-containing protein 58 OS=Sus scrofa OX=9823 GN=MIX23 PE=1 SV=1;tr|I3LE14|I3LE14_PIG Coiled-coil domain-containing protein 58 OS=Sus scrofa OX=9823 GN=MIX23 PE=1 SV=3;tr|A0A5G2R9I4|A0A5G2R9I4_PIG Coiled-coil d
0 0 0 0 0 0 0 0 0 0 0 0 1 1 1 1,7 1,7 1,7
75,649 0,0011587 2,7863 9570300 1 A0A5G2QXD8;F1SMV6
A0A5G2QXD8;F1SMV6 670 tr|A0A5G2QXD8|A0A5G2QXD8_PIG Nucleolin OS=Sus scrofa OX=9823 GN=NCL PE=1 SV=1;tr|F1SMV6|F1SMV6_PIG Nucleolin OS=Sus scrofa OX=9823 GN=NCL PE=1 SV=2
0 0 0 0 0 0 0 0 0 0 0 0 2 2 2 1,8 1,8 1,8
134,76 0,0049505 1,5845 0 2 I3LE33;A0A5G2QXM4 I3LE33;A0A5G2QXM4 671
tr|I3LE33|I3LE33_PIG Zinc finger and BTB domain containing 38 OS=Sus scrofa OX=9823 GN=ZBTB38 PE=4 SV=1;tr|A0A5G2QXM4|A0A5G2QXM4_PIG Zinc finger and BTB domain containing 38 OS=Sus scrofa OX=9823 GN=ZBTB38 PE=4 SV=1
0 0 0 0 0 0 0 0 0 0 0 0 3 3 3 4,7 4,7 4,7
74,35 0,0011338 2,5948 1124000 3 A0A5G2QXN3;O11780;F1RHA7
A0A5G2QXN3;O11780;F1RHA7 672 tr|A0A5G2QXN3|A0A5G2QXN3_PIG Transforming growth factor-beta-induced protein ig-h3 OS=Sus scrofa OX=9823 GN=TGFBI PE=1 SV=1;sp|O11780|BGH3_PIG Transforming growth factor-beta-induced protein ig-h3 OS=Sus scrofa OX=9823 GN=TGFBI PE=1 SV=1;tr|F1RHA7|F1RHA7_P

80292000 0 66196000 78551000 0 119980000 68830000 51380000 79532000 93830000 35693000 85099000 5 5 4 12,6 12,6 9,5 53,519 0 10,684 1523500000 69 A0A5G2QXT5;A0A286ZQF6 A0A5G2QXT5;A0A286ZQF6 673 tr|A0A5G2QXT5|A0A5G2QXT5_PIG Uncharacterized protein OS=Sus scrofa OX=9823 PE=1 SV=1;tr|A0A286ZQF6|A0A286ZQF6_PIG Uncharacterized protein OS=Sus scrofa OX=9823 PE=1 SV=1

16771000 17168000 14758000 32400000 11829000 13222000 24249000 22283000 23266000 25242000 13030000 14346000 2 2 2 12,5 12,5 12,5 25,39 0 12,403 432130000 33 Q2VTP6;A0A5G2QXY7;A0A5G2QMX4 Q2VTP6;A0A5G2QXY7;A0A5G2QMX4 674 tr|Q2VTP6|Q2VTP6_PIG Peptidylprolyl isomerase OS=Sus scrofa OX=9823 GN=FKBP1A PE=1 SV=2;tr|A0A5G2QXY7|A0A5G2QXY7_PIG Peptidylprolyl isomerase OS=Sus scrofa OX=9823 GN=FKBP1A PE=1 SV=1;tr|A0A5G2QMX4|A0A5G2QMX4_PIG Peptidylprolyl isomerase OS=Sus scrofa OX=9

26112000 30788000 28013000 29661000 19758000 28753000 25983000 19219000 22704000 24122000 20726000 31854000 10 10 5 30,6 30,6 16,8 36,668 0 18,923 1033200000 39 A0A5G2QXZ4;A0A5G2R4D9 A0A5G2QXZ4;A0A5G2R4D9 675 tr|A0A5G2QXZ4|A0A5G2QXZ4_PIG Glutathione S-transferase OS=Sus scrofa OX=9823 PE=1 SV=1;tr|A0A5G2R4D9|A0A5G2R4D9_PIG Glutathione S-transferase OS=Sus scrofa OX=9823 PE=1 SV=1

0 0 0 0 0 0 0 0 0 0 0 0 3 2 2 13 7,4 7,4 24,742 0 6,5329 0 2 A0A5G2QY21 A0A5G2QY21 676 tr|A0A5G2QY21|A0A5G2QY21_PIG Reticulocalbin 2 OS=Sus scrofa OX=9823 GN=RCN2 PE=1 SV=1

0 0 14019000 0 0 0 0 15805000 15691000 21144000 0 12837000 3 3 3 21,7 21,7 21,7 18,952 0 9,2504 200990000 14 F1SPZ9;A0A5G2QYC2;A0A5G2REC4;K7GPW3;A0A5G2R5P0 F1SPZ9;A0A5G2QYC2;A0A5G2REC4;K7GPW3;A0A5G2R5P0 677 tr|F1SPZ9|F1SPZ9_PIG MICOS complex subunit OS=Sus scrofa OX=9823 GN=APOO PE=1 SV=4;tr|A0A5G2QYC2|A0A5G2QYC2_PIG MICOS complex subunit OS=Sus scrofa OX=9823 GN=APOO PE=1 SV=1;tr|A0A5G2REC4|A0A5G2REC4_PIG MICOS complex subunit OS=Sus scrofa OX=9823 GN=APOO P

8171000 0 17584000 6577700 0 24083000 7688700 0 9418500 10986000 0 14574000 6 6 6 11,1 11,1 11,1 56,078 0 22,503 414660000 23 A0A5G2QYD6;F1SFI5 A0A5G2QYD6;F1SFI5 678 tr|A0A5G2QYD6|A0A5G2QYD6_PIG Histidine rich glycoprotein OS=Sus scrofa OX=9823 GN=HRG PE=1 SV=1;tr|F1SFI5|F1SFI5_PIG Histidine rich glycoprotein OS=Sus scrofa OX=9823 GN=HRG PE=1 SV=3

17812000 11726000 12207000 16402000 9654500 8946700 18458000 8989100 10342000 21026000 9490800 10916000 3 3 3 35,9 35,9 35,9 14,333 0 13,083 388780000 16 A0A5G2RAT8;P00172;A0A5G2QYD9;A0A5G2QFV0 A0A5G2RAT8;P00172;A0A5G2QYD9;A0A5G2QFV0 679 tr|A0A5G2RAT8|A0A5G2RAT8_PIG Cytochrome b5 OS=Sus scrofa OX=9823 GN=CYB5A PE=1 SV=1;sp|P00172|CYB5_PIG Cytochrome b5 OS=Sus scrofa OX=9823 GN=CYB5A PE=1

SV=3;tr|A0A5G2QYD9|A0A5G2QYD9_PIG Cytochrome b5 OS=Sus scrofa OX=9823 GN=CYB5A PE=1 SV=1;tr|A0A5G2QFV0|
54502000 76868000 55961000 71058000 74315000 98316000 75293000 64545000 71000000 64816000 77027000 71170000 5 3 3 10,4 6,6 6,6 41,277 0,0011364 2,6179 1008100000 14 A0A5G2QYS6;I3LLU0;A0A287AV28 A0A5G2QYS6;I3LLU0;A0A287AV28 680 tr|A0A5G2QYS6|A0A5G2QYS6_PIG Glycerol-3-phosphate dehydrogenase [NAD(+)] OS=Sus scrofa OX=9823 GN=GPD1L PE=1 SV=1;tr|I3LLU0|I3LLU0_PIG Glycerol-3-phosphate dehydrogenase [NAD(+)] OS=Sus scrofa OX=9823 GN=GPD1L PE=1 SV=2;tr|A0A287AV28|A0A287AV28_PIG Glycero
12719000 21479000 17247000 21547000 18473000 21521000 17059000 16975000 23008000 16412000 22234000 18307000 4 4 4 8,4 8,4 8,4 56,71 0 10,606 376730000 26 F6QAM9;A0A5G2QYT3;A0A5G2RD31 F6QAM9;A0A5G2QYT3;A0A5G2RD31 681 tr|F6QAM9|F6QAM9_PIG Adenylosuccinate lyase OS=Sus scrofa OX=9823 GN=ADSL PE=1 SV=2;tr|A0A5G2QYT3|A0A5G2QYT3_PIG Adenylosuccinate lyase OS=Sus scrofa OX=9823 GN=ADSL PE=1 SV=1;tr|A0A5G2RD31|A0A5G2RD31_PIG Adenylosuccinase OS=Sus scrofa OX=9823 GN=ADSL PE=1
0 0 0 0 0 0 0 0 0 0 0 0 33 1 0 16,5 0,4 0 222,34 0,0086873 1,4059 186950000 8 A0A5G2QYU9;F1SS61;A0A5G2QTZ6 A0A5G2QYU9;F1SS61;A0A5G2QTZ6 682 tr|A0A5G2QYU9|A0A5G2QYU9_PIG Myosin heavy chain 3 OS=Sus scrofa OX=9823 GN=MYH3 PE=1 SV=1;tr|F1SS61|F1SS61_PIG Myosin heavy chain 3 OS=Sus scrofa OX=9823 GN=MYH3 PE=1 SV=2;tr|A0A5G2QTZ6|A0A5G2QTZ6_PIG Myosin heavy chain 3 OS=Sus scrofa OX=9823 GN=MYH3 PE=1
7402500 5956600 6710200 0 10397000 5758200 8219700 0 6534600 0 7677000 7722000 3 3 3 3,4 3,4 3,4 97,224 0 6,075 130510000 17 F1RWJ5;A0A5G2QZ16 F1RWJ5;A0A5G2QZ16 683 tr|F1RWJ5|F1RWJ5_PIG Importin subunit beta-1 OS=Sus scrofa OX=9823 GN=KPNB1 PE=1 SV=3;tr|A0A5G2QZ16|A0A5G2QZ16_PIG Karyopherin subunit beta 1 OS=Sus scrofa OX=9823 GN=KPNB1 PE=1 SV=1
5139400 0 0 4076300 0 0 0 0 4559200 7233600 0 4695700 3 3 3 13 13 13 34,509 0 4,428 73919000 6 A0A5G2QZ46;Q9GKX6;A0A5G2QHL8;A0A5S6G4Y0;A0A5G2R1N9;A0A5G2R053 A0A5G2QZ46;Q9GKX6;A0A5G2QHL8;A0A5S6G4Y0;A0A5G2R1N9 684 tr|A0A5G2QZ46|A0A5G2QZ46_PIG Galactose mutarotase OS=Sus scrofa OX=9823 GN=GALM PE=4 SV=1;sp|Q9GKX6|GALM_PIG Galactose mutarotase OS=Sus scrofa OX=9823 GN=GALM PE=2 SV=1;tr|A0A5G2QHL8|A0A5G2QHL8_PIG Galactose mutarotase OS=Sus scrofa OX=9823 GN=GALM PE=4 S
25662000 35200000 37638000 0 21138000 0 6673800 29202000 8818000 10019000 24398000 11050000 10 10 10 7,1 7,1 7,1 779,55 0 43,287 564120000 39 A0A5G2QZ79;A0A287BRL9 A0A5G2QZ79 685 tr|A0A5G2QZ79|A0A5G2QZ79_PIG Uncharacterized protein OS=Sus scrofa OX=9823 GN=OBSCN PE=3 SV=1
41247000 24476000 29148000 27001000 28803000 28848000 27769000 21632000 27631000 27434000 19335000 22642000 3 3 1 25 25 7,6 16,512 0 39,144 652530000 37 A0A5G2RGI4;A0A5G2QZC6 A0A5G2RGI4;A0A5G2QZC6 686

tr|A0A5G2RGI4|A0A5G2RGI4_PIG UBC core domain-containing protein OS=Sus scrofa OX=9823 GN=UBE2N PE=1 SV=1;tr|A0A5G2QZC6|A0A5G2QZC6_PIG UBC core domain-containing protein OS=Sus scrofa OX=9823 GN=UBE2N PE=1 SV=1
0 0 0 0 0 0 0 0 0 0 0 0 1 1 1 4,7 4,7 4,7
25,898 0 4,4435 60684000 11 I3LAB6;A0A5G2QZH5 I3LAB6;A0A5G2QZH5 687
tr|I3LAB6|I3LAB6_PIG Proteasome subunit alpha type OS=Sus scrofa OX=9823 GN=PSMA2 PE=1 SV=1;tr|A0A5G2QZH5|A0A5G2QZH5_PIG Uncharacterized protein OS=Sus scrofa OX=9823 GN=PSMA2 PE=1 SV=1
8427000000 13341000000 7487200000 5204000000 10374000000 7736700000 5806600000
10539000000 7517300000 7234100000 9609200000 6654100000 25 25
11 62,5 62,5 25,2 43,059 0 323,31 726280000000 5071
Q5XLD3;A0A5G2QZN6;A0A287AMP3 Q5XLD3;A0A5G2QZN6;A0A287AMP3 688
sp|Q5XLD3|KCRM_PIG Creatine kinase M-type OS=Sus scrofa OX=9823 GN=CKM PE=2 SV=1;tr|A0A5G2QZN6|A0A5G2QZN6_PIG Creatine kinase OS=Sus scrofa OX=9823 GN=CKM PE=1 SV=1;tr|A0A287AMP3|A0A287AMP3_PIG Creatine kinase OS=Sus scrofa OX=9823 GN=CKM PE=1 SV=1
6076800 8096000 7639500 0 6330200 0 0 0 0 0 0 0 4 4
4 4,7 4,7 4,7 101,5 0 6,611 83628000 8 F1RUY9;A0A5G2QZP0;A0A287BLD0
F1RUY9;A0A5G2QZP0 689 tr|F1RUY9|F1RUY9_PIG Carnosine synthase 1 OS=Sus scrofa OX=9823 GN=CARNS1 PE=1 SV=2;tr|A0A5G2QZP0|A0A5G2QZP0_PIG Carnosine synthase 1 OS=Sus scrofa OX=9823 GN=CARNS1 PE=1 SV=1
24672000 38024000 33805000 26803000 37781000 36298000 29481000 27759000 32878000 17712000
21646000 26581000 4 4 4 18 18 18 25,126 0 16,813
1029700000 35 A0A5G2QZY6;F1RFQ7;A0A5G2QD71
A0A5G2QZY6;F1RFQ7;A0A5G2QD71 690 tr|A0A5G2QZY6|A0A5G2QZY6_PIG GTP-binding nuclear protein Ran OS=Sus scrofa OX=9823 GN=RAN PE=1 SV=1;tr|F1RFQ7|F1RFQ7_PIG GTP-binding nuclear protein Ran OS=Sus scrofa OX=9823 GN=RAN PE=1 SV=2;tr|A0A5G2QD71|A0A5G2QD71_PIG GTP-binding nuclear protein Ran OS=
0 25053000 20519000 59470000 0 0 0 0 0 0 0 0 3 3 0
26,6 26,6 0 19,67 0 17,589 407760000 19 A0A5G2R0B0 A0A5G2R0B0 691
tr|A0A5G2R0B0|A0A5G2R0B0_PIG Protein phosphatase 1 regulatory inhibitor subunit 1A OS=Sus scrofa OX=9823 GN=PPP1R1A PE=1 SV=1
19276000 15783000 33389000 34374000 34729000 36888000 27403000 18717000 30101000 44033000
33844000 67274000 9 9 9 39,7 39,7 39,7 25,455 0 29,859
2535900000 104 A0A5G2R0H0;Q6UAQ8;A0A5G2R9W5;A0A5G2QX18
A0A5G2R0H0;Q6UAQ8;A0A5G2R9W5;A0A5G2QX18 692
tr|A0A5G2R0H0|A0A5G2R0H0_PIG Electron transfer flavoprotein subunit beta OS=Sus scrofa OX=9823 GN=ETFB PE=1 SV=1;sp|Q6UAQ8|ETFB_PIG Electron transfer flavoprotein subunit beta OS=Sus scrofa OX=9823 GN=ETFB PE=1 SV=3;tr|A0A5G2R9W5|A0A5G2R9W5_PIG Electron tr
0 25155000 57360000 83673000 49176000 43710000 49279000 28567000 62522000 74951000 51204000
59493000 2 2 2 44,9 44,9 44,9 5,7487 0 25,366 983810000 46
A0A5G2R0H7;Q95339;A0A5S6G3W0 A0A5G2R0H7;Q95339;A0A5S6G3W0 693
tr|A0A5G2R0H7|A0A5G2R0H7_PIG ATP synthase membrane subunit f OS=Sus scrofa

OX=9823 GN=ATP5MF PE=3 SV=1;sp|Q95339|ATPK_PIG ATP synthase subunit f, mitochondrial OS=Sus scrofa OX=9823 GN=ATP5MF PE=1 SV=4;tr|A0A5S6G3W0|A0A5S6G3W0_PIG ATP synthase membrane sub
0 7870900 12145000 0 10181000 11348000 0 7341000 7229000 7747400 8740500 21146000
5 5 5 17,4 17,4 17,4 37,149 0 23,196 319070000 18
A0A5G2R8L9;A0A5G2R0Z8;F1SNA8;K7GNL4 A0A5G2R8L9;A0A5G2R0Z8;F1SNA8;K7GNL4
694 tr|A0A5G2R8L9|A0A5G2R8L9_PIG Hydroxysteroid dehydrogenase like 2 OS=Sus scrofa OX=9823 GN=HSDL2 PE=1 SV=1;tr|A0A5G2R0Z8|A0A5G2R0Z8_PIG Hydroxysteroid dehydrogenase like 2 OS=Sus scrofa OX=9823 GN=HSDL2 PE=1 SV=1;tr|F1SNA8|F1SNA8_PIG Hydroxysteroid dehydrog
11446000 10746000 12050000 15997000 6196000 7375100 18742000 11265000 17662000 22111000
6797500 12102000 5 5 5 27,9 27,9 27,9 23,491 0 15,799
502230000 31 A0A5G2R1E6;Q9N1F5;F1S5N8 A0A5G2R1E6;Q9N1F5 695
tr|A0A5G2R1E6|A0A5G2R1E6_PIG Glutathione-dependent dehydroascorbate reductase OS=Sus scrofa OX=9823 GN=GSTO1 PE=1 SV=1;sp|Q9N1F5|GSTO1_PIG Glutathione S-transferase omega-1 OS=Sus scrofa OX=9823 GN=GSTO1 PE=1 SV=2
0 0 0 0 0 0 0 0 0 0 0 0 3 3 3 3,1 3,1 3,1
100,63 0 3,5627 17185000 3 A0A5G2R1I5;F1SBU7;I3L7T4;A0A5G2R1B2
A0A5G2R1I5;F1SBU7;I3L7T4;A0A5G2R1B2 696 tr|A0A5G2R1I5|A0A5G2R1I5_PIG Lon protease homolog, mitochondrial OS=Sus scrofa OX=9823 GN=LONP1 PE=1 SV=1;tr|F1SBU7|F1SBU7_PIG Lon protease homolog, mitochondrial OS=Sus scrofa OX=9823 GN=LONP1 PE=1 SV=2;tr|I3L7T4|I3L7T4_PIG Lon protease homolog, mitochond
0 0 0 0 0 0 0 0 0 0 0 0 2 2 2 6,4 6,4 6,4
31,98 0,0031217 1,909 25066000 3
I3LQA8;A0A5G2R1L7;A0A287BQJ3;F6Q3L0;A0A286ZPA2
I3LQA8;A0A5G2R1L7;A0A287BQJ3;F6Q3L0;A0A286ZPA2 697 tr|I3LQA8|I3LQA8_PIG Microtubule-associated protein RP/EB family member 3 OS=Sus scrofa OX=9823 GN=MAPRE3 PE=1 SV=2;tr|A0A5G2R1L7|A0A5G2R1L7_PIG Microtubule associated protein RP/EB family member 3 OS=Sus scrofa OX=9823 GN=MAPRE3 PE=1 SV=1;tr|A0A287BQJ3|A0
19894000 21502000 42807000 42982000 46051000 40730000 34108000 25617000 35270000 36973000
53478000 63256000 6 6 6 19,5 19,5 19,5 36,301 0 42,037
1206000000 53 A0A5G2R1P2;I3LDC1;Q007T0 A0A5G2R1P2;I3LDC1;Q007T0 698
tr|A0A5G2R1P2|A0A5G2R1P2_PIG Succinate dehydrogenase [ubiquinone] iron-sulfur subunit, mitochondrial OS=Sus scrofa OX=9823 GN=SDHB PE=1 SV=1;tr|I3LDC1|I3LDC1_PIG Succinate dehydrogenase [ubiquinone] iron-sulfur subunit, mitochondrial OS=Sus scrofa OX=9823
4256000 0 0 0 0 0 7107200 0 3663200 4233500 0 0 4 4
4 21,6 21,6 21,6 27,05 0 8,0558 52169000 10
A0A5G2R267;A0A5G2QPH4;F1SGT2;K7GLU2;A0A5G2Q9S9;A0A287ALG9;A0A286ZZL6
A0A5G2R267;A0A5G2QPH4;F1SGT2;K7GLU2;A0A5G2Q9S9;A0A287ALG9;A0A286ZZL6 699
tr|A0A5G2R267|A0A5G2R267_PIG Methylthioribulose-1-phosphate dehydratase OS=Sus scrofa OX=9823 GN=APIP PE=1 SV=1;tr|A0A5G2QPH4|A0A5G2QPH4_PIG Methylthioribulose-1-phosphate dehydratase OS=Sus scrofa OX=9823 GN=APIP PE=1 SV=1;tr|F1SGT2|F1SGT2_PIG Methylthior

11928000 7699600 0 0 7379100 0 7775300 0 0 6024800 0 0 5
5 5 14,4 14,4 14,4 49,065 0 10,647 114180000 16 F1RGN8;A0A5G2R298
F1RGN8;A0A5G2R298 700 tr|F1RGN8|F1RGN8_PIG Smoothelin like 2 OS=Sus scrofa OX=9823 GN=SMTNL2 PE=1 SV=1;tr|A0A5G2R298|A0A5G2R298_PIG Smoothelin like 2 OS=Sus scrofa OX=9823 GN=SMTNL2 PE=1 SV=1
5080900 8257000 12536000 8485900 18123000 17087000 4488300 10894000 15863000 9141300
19504000 31628000 8 8 8 16,1 16,1 16,1 66,118 0 42,795
595670000 59 A0A5G2R2C9;F1RTH3;A0A5G2QTA3;A0A5G2QQE8;A0A5G2R2M9
A0A5G2R2C9;F1RTH3;A0A5G2QTA3;A0A5G2QQE8 701
tr|A0A5G2R2C9|A0A5G2R2C9_PIG Apoptosis inducing factor mitochondria associated 1 OS=Sus scrofa OX=9823 GN=AIFM1 PE=1 SV=1;tr|F1RTH3|F1RTH3_PIG Apoptosis inducing factor mitochondria associated 1 OS=Sus scrofa OX=9823 GN=AIFM1 PE=1 SV=1;tr|A0A5G2QTA3|A0A5G2
0 0 8709000 18140000 11937000 11445000 22234000 9707500 12689000 0 10205000 10611000
5 5 5 20,6 20,6 20,6 26,029 0 6,6635 262600000 14
A0A5G2R2Y1;F1SSL6;A0A5G2QWE9 A0A5G2R2Y1;F1SSL6;A0A5G2QWE9 702
tr|A0A5G2R2Y1|A0A5G2R2Y1_PIG Proteasome subunit alpha type OS=Sus scrofa OX=9823 GN=PSMA3 PE=1 SV=1;tr|F1SSL6|F1SSL6_PIG Proteasome subunit alpha type OS=Sus scrofa OX=9823 GN=PSMA3 PE=1 SV=1;tr|A0A5G2QWE9|A0A5G2QWE9_PIG Proteasome subunit alpha type OS=Su
16392000 19899000 10636000 21843000 18725000 0 25807000 16289000 12264000 24630000 0 0
8 8 8 31 31 31 37,422 0 25,004 473270000 14
A0A5G2R384;A0A5G2QH28;F1RUJ4;A0A287AC11;A0A5G2R668;A0A287AYH2;A0A5G2QD15;A0A287B1Y7;F1SGN8;A0A5G2R2M5;A0A5G2QD33;A0A287A4Y2;F1RPP3;A0A287AHW1
A0A5G2R384;A0A5G2QH28;F1RUJ4;A0A287AC11 703 tr|A0A5G2R384|A0A5G2R384_PIG GMP reductase OS=Sus scrofa OX=9823 GN=GMPR PE=1 SV=1;tr|A0A5G2QH28|A0A5G2QH28_PIG GMP reductase OS=Sus scrofa OX=9823 GN=GMPR PE=1 SV=1;tr|F1RUJ4|F1RUJ4_PIG GMP reductase OS=Sus scrofa OX=9823 GN=GMPR PE=1 SV=2;tr|A0A287AC11|A
11952000 17558000 43766000 20445000 42309000 39518000 11274000 32214000 30197000 17223000
42564000 80190000 13 13 13 22,7 22,7 22,7 69,844 0 67,459
2143400000 105 F1ST43;A0A5G2R3E4 F1ST43;A0A5G2R3E4 704 tr|F1ST43|F1ST43_PIG Acyl-CoA dehydrogenase very long chain OS=Sus scrofa OX=9823 GN=ACADVL PE=1 SV=4;tr|A0A5G2R3E4|A0A5G2R3E4_PIG Acyl-CoA dehydrogenase very long chain OS=Sus scrofa OX=9823 GN=ACADVL PE=1 SV=1
0 0 4128900 6858700 0 0 8512500 0 0 7634200 0 0 2 2
2 5 5 5 42,518 0 3,489 66666000 9 A0A5G2R3I8;F1RN44
A0A5G2R3I8;F1RN44 705 tr|A0A5G2R3I8|A0A5G2R3I8_PIG Lysosomal associated membrane protein 1 OS=Sus scrofa OX=9823 GN=LAMP1 PE=1 SV=1;tr|F1RN44|F1RN44_PIG Lysosomal associated membrane protein 1 OS=Sus scrofa OX=9823 GN=LAMP1 PE=1 SV=2
23709000 0 12138000 30591000 33814000 41921000 30844000 0 15957000 44085000 22902000
25886000 6 6 6 7 7 7 80,864 0 6,302 595330000 22
A0A5G2R3M0;A0A5G2R0M3;I3LL94 A0A5G2R3M0;A0A5G2R0M3 706

tr|A0A5G2R3M0|A0A5G2R3M0_PIG Triadin OS=Sus scrofa OX=9823 GN=TRDN PE=1 SV=1;tr|A0A5G2R0M3|A0A5G2R0M3_PIG Triadin OS=Sus scrofa OX=9823 GN=TRDN PE=1 SV=1
14050000 18547000 45862000 41741000 29809000 42293000 17180000 20137000 47164000 35532000 35840000 84624000 6 6 6 11,4 11,4 11,4 52,886 0 16,264 1055900000 50 A0A5G2R3N7;F1S1A8 A0A5G2R3N7;F1S1A8 707

tr|A0A5G2R3N7|A0A5G2R3N7_PIG Complex I-49kD OS=Sus scrofa OX=9823 GN=NDUFS2 PE=1 SV=1;tr|F1S1A8|F1S1A8_PIG Complex I-49kD OS=Sus scrofa OX=9823 GN=NDUFS2 PE=1 SV=2
11537000 0 8137700 10125000 8982400 8093200 17439000 0 7864700 16103000 0 8337500 4 4 4 14,7 14,7 14,7 39,752 0 13,333 248250000 10

F6Q4F9;A0A5G2R4U3;Q684M6 F6Q4F9;A0A5G2R4U3;Q684M6 708

tr|F6Q4F9|F6Q4F9_PIG Hsp90 chaperone protein kinase-targeting subunit OS=Sus scrofa OX=9823 GN=CDC37 PE=1 SV=1;tr|A0A5G2R4U3|A0A5G2R4U3_PIG Hsp90 chaperone protein kinase-targeting subunit OS=Sus scrofa OX=9823 GN=CDC37 PE=1 SV=1;tr|Q684M6|Q684M6_PIG Hsp90
0 0 0 0 0 0 0 0 0 0 0 0 1 1 1 6,3 6,3 6,3 27,455 0,0011601 2,7978 27126000 3 A0A5K1VDT5;F2Z567;A0A5G2R4W2

A0A5K1VDT5;F2Z567;A0A5G2R4W2 709 tr|A0A5K1VDT5|A0A5K1VDT5_PIG 60S ribosomal protein L8 OS=Sus scrofa OX=9823 GN=RPL8 PE=1 SV=1;tr|F2Z567|F2Z567_PIG 60S ribosomal protein L8 OS=Sus scrofa OX=9823 GN=RPL8 PE=1 SV=1;tr|A0A5G2R4W2|A0A5G2R4W2_PIG 60S ribosomal protein L8 OS=Sus scrofa OX=9823
0 0 0 0 0 0 0 0 0 0 0 0 2 2 2 1,4 1,4 1,4 188,61 0 5,6652 140084000 7 I3LGD4;A0A5K1U8P1;A0A5G2R4Y7

I3LGD4;A0A5K1U8P1;A0A5G2R4Y7 710 tr|I3LGD4|I3LGD4_PIG Clathrin heavy chain OS=Sus scrofa OX=9823 GN=CLTC PE=1 SV=3;tr|A0A5K1U8P1|A0A5K1U8P1_PIG Clathrin heavy chain OS=Sus scrofa OX=9823 GN=CLTC PE=1 SV=1;tr|A0A5G2R4Y7|A0A5G2R4Y7_PIG Clathrin heavy chain OS=Sus scrofa OX=9823 GN=CLTC PE=1
0 0 0 0 0 0 0 0 0 0 0 0 1 1 1 2,8 2,8 2,8 43,037 0,0077973 1,4785 4640300 1 A0A5G2R529;F1SF13 A0A5G2R529;F1SF13

711 tr|A0A5G2R529|A0A5G2R529_PIG Cobl domain-containing protein OS=Sus scrofa OX=9823 PE=4 SV=1;tr|F1SF13|F1SF13_PIG Cobl domain-containing protein OS=Sus scrofa OX=9823 PE=4 SV=3
0 17095000 21464000 13808000 16668000 25465000 9940600 16014000 27374000 10415000 19716000 25837000 5 5 5 24 24 24 26,515 0 11,079 409350000 28 A0A5S6FWI2;Q9TV69;A0A5G2R5E5;A0A5G2R1Z9;F1RIQ4;A0A5G2RFU3

A0A5S6FWI2;Q9TV69;A0A5G2R5E5;A0A5G2R1Z9;F1RIQ4 712

tr|A0A5S6FWI2|A0A5S6FWI2_PIG Trans-1,2-dihydrobenzene-1,2-diol dehydrogenase OS=Sus scrofa OX=9823 GN=DHDH PE=4 SV=1;sp|Q9TV69|DHDH_PIG Trans-1,2-dihydrobenzene-1,2-diol dehydrogenase OS=Sus scrofa OX=9823 GN=DHDH PE=1 SV=1;tr|A0A5G2R5E5|A0A5G2R5E5_PIG Tra
0 0 0 0 0 0 0 0 0 0 0 0 2 2 2 21,3 21,3 21,3 15,016 0 108,46 82568000 12 A0A5G2R5U8;K7GNZ3 A0A5G2R5U8;K7GNZ3

713 tr|A0A5G2R5U8|A0A5G2R5U8_PIG NAC-A/B domain-containing protein OS=Sus scrofa

OX=9823 GN=NACA PE=1 SV=1;tr|K7GNZ3|K7GNZ3_PIG NAC-A/B domain-containing protein OS=Sus scrofa OX=9823 GN=NACA PE=1 SV=2
7631800 9109100 35401000 34469000 23758000 28363000 30591000 8151100 20884000 34712000
19914000 37299000 8 8 8 24,1 24,1 24,1 37,655 0 32,691
1248700000 80 A0A5G2R5W7;A0A5G2QV44;A0A5K1U5Q1
A0A5G2R5W7;A0A5G2QV44;A0A5K1U5Q1 714 tr|A0A5G2R5W7|A0A5G2R5W7_PIG 2,4-dienoyl-CoA reductase 1 OS=Sus scrofa OX=9823 GN=DECR1 PE=1 SV=1;tr|A0A5G2QV44|A0A5G2QV44_PIG 2,4-dienoyl-CoA reductase 1 OS=Sus scrofa OX=9823 GN=DECR1 PE=1 SV=1;tr|A0A5K1U5Q1|A0A5K1U5Q1_PIG 2,4-dienoyl-CoA reductase 1 OS=
13312000 9310000 6536100 17922000 6393900 7857700 20166000 6383400 6206300 14185000
7799800 6641400 5 5 0 45,7 45,7 0 14,389 0 17,336
336060000 38 A0A5G2R631;A0A5G2QQ27;A0A5G2Q930 A0A5G2R631;A0A5G2QQ27
715 tr|A0A5G2R631|A0A5G2R631_PIG Programmed cell death 5 OS=Sus scrofa OX=9823 GN=PDCD5 PE=1 SV=1;tr|A0A5G2QQ27|A0A5G2QQ27_PIG Programmed cell death 5 OS=Sus scrofa OX=9823 GN=PDCD5 PE=1 SV=1
0 9796900 35940000 0 0 0 0 0 0 0 0 5785200 4 4 4
19,4 19,4 19,4 28,133 0 7,4627 156370000 7
I3LJ05;A0A5G2R6F6;A0A5G2QJL9;A0A287ANM8;A0A5G2R3V5
I3LJ05;A0A5G2R6F6;A0A5G2QJL9;A0A287ANM8 716 tr|I3LJ05|I3LJ05_PIG Uncharacterized protein OS=Sus scrofa OX=9823 GN=GATD3A PE=1 SV=3;tr|A0A5G2R6F6|A0A5G2R6F6_PIG Uncharacterized protein OS=Sus scrofa OX=9823 GN=GATD3A PE=1 SV=1;tr|A0A5G2QJL9|A0A5G2QJL9_PIG Uncharacterized protein OS=Sus scrofa OX=9823
0 0 0 0 0 0 0 0 0 0 0 0 8 1 1 13,7 1,7 1,7
71,108 0 8,7947 4462300000 21 Q04967;A0A5G2R717 Q04967;A0A5G2R717 717
sp|Q04967|HSP76_PIG Heat shock 70 kDa protein 6 OS=Sus scrofa OX=9823 GN=HSPA6 PE=1 SV=1;tr|A0A5G2R717|A0A5G2R717_PIG Heat shock 70 kDa protein 6 OS=Sus scrofa OX=9823 GN=HSPA6 PE=3 SV=1
0 0 0 0 0 0 0 0 0 0 0 0 1 1 1 2,3 2,3 2,3
55,261 0,001139 2,6445 29875000 4 A0A5G2R740;F1RM25 A0A5G2R740;F1RM25 718
tr|A0A5G2R740|A0A5G2R740_PIG Serine/threonine-protein phosphatase OS=Sus scrofa OX=9823 GN=PPP5C PE=1 SV=1;tr|F1RM25|F1RM25_PIG Serine/threonine-protein phosphatase OS=Sus scrofa OX=9823 GN=PPP5C PE=1 SV=3
30264000 20309000 24486000 57156000 28011000 32035000 54216000 19644000 44498000 51983000
23977000 23637000 6 6 6 13,5 13,5 13,5 57,464 0 13,625
1035100000 31 I3L9F5;A0A5G2R787;A0A287BMH4 I3L9F5;A0A5G2R787;A0A287BMH4
719 tr|I3L9F5|I3L9F5_PIG Prenylcysteine oxidase 1 OS=Sus scrofa OX=9823 GN=PCYOX1 PE=1 SV=3;tr|A0A5G2R787|A0A5G2R787_PIG Prenylcysteine oxidase 1 OS=Sus scrofa OX=9823 GN=PCYOX1 PE=1 SV=1;tr|A0A287BMH4|A0A287BMH4_PIG Prenylcysteine oxidase 1 OS=Sus scrofa OX=9
0 0 14870000 0 0 13164000 0 0 0 0 0 11567000 6 2 1
22,9 7,3 3,2 25,444 0,0022497 2,5122 99202000 4 A0A5G2R7F0 A0A5G2R7F0 720
tr|A0A5G2R7F0|A0A5G2R7F0_PIG Glutathione transferase OS=Sus scrofa OX=9823 PE=1 SV=1

0 0 0 0 0 0 0 0 0 0 0 0 3 3 3 6,2 6,2 6,2

64,901 0 9,6476 7752400 3 A5A779;A0A5G2R7L5 A5A779;A0A5G2R7L5 721

sp|A5A779|PGTA_PIG Geranylgeranyl transferase type-2 subunit alpha OS=Sus scrofa OX=9823 GN=RABGGTA PE=2 SV=1;tr|A0A5G2R7L5|A0A5G2R7L5_PIG Geranylgeranyl transferase type-2 subunit alpha OS=Sus scrofa OX=9823 GN=RABGGTA PE=1 SV=1

0 0 0 0 0 0 0 0 0 0 0 0 1 1 1 8,1 8,1 8,1

19,307 0 9,1232 12247000 3 A0A5G2R7V4;A0A5G2RIY7;F1SM07

A0A5G2R7V4;A0A5G2RIY7;F1SM07 722 tr|A0A5G2R7V4|A0A5G2R7V4_PIG COP9 signalosome subunit 8 OS=Sus scrofa OX=9823 GN=COPS8 PE=1 SV=1;tr|A0A5G2RIY7|A0A5G2RIY7_PIG COP9 signalosome complex subunit 8 OS=Sus scrofa OX=9823 GN=COPS8 PE=1 SV=1;tr|F1SM07|F1SM07_PIG COP9 signalosome complex subunit

0 0 0 7434800 0 0 5519900 0 4391400 0 0 0 4 4 4

18,1 18,1 18,1 31,82 0 3,2044 58672000 5 A0A5G2R815;A0A5G2QKM6;I3LRS8

A0A5G2R815;A0A5G2QKM6;I3LRS8 723 tr|A0A5G2R815|A0A5G2R815_PIG Phosphatidylinositol transfer protein alpha OS=Sus scrofa OX=9823 GN=PITPNA PE=1 SV=1;tr|A0A5G2QKM6|A0A5G2QKM6_PIG Phosphatidylinositol transfer protein alpha OS=Sus scrofa OX=9823 GN=PITPNA PE=1 SV=1;tr|I3LRS8|I3LRS8_PIG Phosp

0 0 0 0 0 0 0 0 0 0 0 0 4 4 4 5,5 5,5 5,5

73,966 0 6,2288 32502000 6 A0A5G2R8A9;A0A287AYD0;F1S764

A0A5G2R8A9;A0A287AYD0;F1S764 724 tr|A0A5G2R8A9|A0A5G2R8A9_PIG Carnitine palmitoyltransferase 2 OS=Sus scrofa OX=9823 GN=CPT2 PE=1 SV=1;tr|A0A287AYD0|A0A287AYD0_PIG Carnitine palmitoyltransferase 2 OS=Sus scrofa OX=9823 GN=CPT2 PE=1 SV=1;tr|F1S764|F1S764_PIG Carnitine palmitoyltransferase

5662700 8405800 38308000 0 4368200 4231800 0 0 2139000 2669700 0 13966000

8 8 8 12,7 12,7 12,7 66,187 0 11,891 381280000 13

K9IVR7;A0A5G2R8D7;A0A5G2QAB3 K9IVR7;A0A5G2R8D7 725 tr|K9IVR7|K9IVR7_PIG WD repeat domain 1 OS=Sus scrofa OX=9823 GN=WDR1 PE=2 SV=1;tr|A0A5G2R8D7|A0A5G2R8D7_PIG WD repeat domain 1 OS=Sus scrofa OX=9823 GN=WDR1 PE=4 SV=1

0 0 0 10517000 0 0 7708600 0 0 9155300 0 0 2 2 2

17,5 17,5 17,5 11,202 0 2,9182 89954000 5 A0A5G2R965 A0A5G2R965 726

tr|A0A5G2R965|A0A5G2R965_PIG Uncharacterized protein OS=Sus scrofa OX=9823 GN=LOC102167522 PE=1 SV=1

5734100 0 0 0 0 0 0 0 0 7292800 0 0 2 2 2 8,7

8,7 8,7 28,544 0 3,3034 71191000 13 A0A5G2R9C3;F1RJM2 A0A5G2R9C3;F1RJM2

728 tr|A0A5G2R9C3|A0A5G2R9C3_PIG Endoplasmic reticulum resident protein 29 OS=Sus scrofa OX=9823 GN=ERP29 PE=1 SV=1;tr|F1RJM2|F1RJM2_PIG Endoplasmic reticulum resident protein 29 OS=Sus scrofa OX=9823 GN=ERP29 PE=1 SV=2

0 0 0 0 0 0 0 0 0 0 0 0 1 1 1 13,7 13,7 13,7

10,606 0 9,5123 1490100000 13 A0A5G2R9F7 A0A5G2R9F7 729

tr|A0A5G2R9F7|A0A5G2R9F7_PIG Uncharacterized protein OS=Sus scrofa OX=9823 GN=FKBP1A PE=1 SV=1

0 0 0 0 0 0 0 0 0 0 0 0 1 1 1 3,5 3,5 3,5
31,111 0,0031546 1,9728 37345000 6 I3LIH2;A0A5G2R9X9 I3LIH2;A0A5G2R9X9
730 tr|I3LIH2|I3LIH2_PIG Inositol-1-monophosphatase OS=Sus scrofa OX=9823 GN=IMPA2 PE=3 SV=3;tr|A0A5G2R9X9|A0A5G2R9X9_PIG CIDE-N domain-containing protein OS=Sus scrofa OX=9823 GN=IMPA2 PE=3 SV=1
108970000 74466000 42106000 14711000 64601000 7785600 94464000 31440000 56059000 67849000
32590000 6679000 12 12 12 37,1 37,1 37,1 40,927 0 63,348
2430600000 74 F1RYS9;A0A5G2R9Y3 F1RYS9;A0A5G2R9Y3 731
tr|F1RYS9|F1RYS9_PIG CBM20 domain-containing protein OS=Sus scrofa OX=9823 GN=STBD1 PE=1 SV=3;tr|A0A5G2R9Y3|A0A5G2R9Y3_PIG CBM20 domain-containing protein OS=Sus scrofa OX=9823 GN=STBD1 PE=1 SV=1
13793000 13064000 18333000 14510000 18636000 11225000 15067000 9946000 10339000 19434000
13910000 13694000 12 12 12 24,3 24,3 24,3 57,704 0 19,43
644350000 35 F1S3H1;A0A5G2RA17;A0A5G2RB26 F1S3H1;A0A5G2RA17;A0A5G2RB26
732 tr|F1S3H1|F1S3H1_PIG Aldehyde dehydrogenase 6 family member A1 OS=Sus scrofa OX=9823 GN=ALDH6A1 PE=1 SV=2;tr|A0A5G2RA17|A0A5G2RA17_PIG Aldehyde dehydrogenase 6 family member A1 OS=Sus scrofa OX=9823 GN=ALDH6A1 PE=1 SV=1;tr|A0A5G2RB26|A0A5G2RB26_PIG Aldehyd
0 0 15918000 0 25211000 0 0 0 0 12636000 0 14858000 2 2
2 10,8 10,8 10,8 16,74 0 3,213 200490000 12 A0A5G2RA78;A5GZW8;F1SMA9
A0A5G2RA78;A5GZW8;F1SMA9 733 tr|A0A5G2RA78|A0A5G2RA78_PIG Succinate dehydrogenase [ubiquinone] cytochrome b small subunit OS=Sus scrofa OX=9823 GN=SDHD PE=3 SV=1;sp|A5GZW8|DHSD_PIG Succinate dehydrogenase [ubiquinone] cytochrome b small subunit, mitochondrial OS=Sus scrofa OX=9823 GN=
6002200 7672400 6269900 10228000 9649000 8859700 7684900 11307000 9439900 0 9222900
8926200 5 5 5 11,2 11,2 11,2 59,473 0 12,581 235350000 26
D0G0C9;A0A5G2RAV7;A0A287AKF5;A0A5G2R1J8;A0A5G2RH19
D0G0C9;A0A5G2RAV7;A0A287AKF5;A0A5G2R1J8 735 tr|D0G0C9|D0G0C9_PIG T-complex protein 1 subunit eta OS=Sus scrofa OX=9823 GN=CCT7 PE=1 SV=1;tr|A0A5G2RAV7|A0A5G2RAV7_PIG T-complex protein 1 subunit eta OS=Sus scrofa OX=9823 GN=CCT7 PE=1 SV=1;tr|A0A287AKF5|A0A287AKF5_PIG 60 kDa chaperonin OS=Sus scrofa O
5703200 0 0 3884400 17663000 100580000 0 0 0 19935000 0 0
6 6 6 24,1 24,1 24,1 26,215 0 12,733 580450000 19 A0A5G2RB71;F1S1B2
A0A5G2RB71;F1S1B2 736 tr|A0A5G2RB71|A0A5G2RB71_PIG Myelin peripheral protein OS=Sus scrofa OX=9823 GN=MPZ PE=1 SV=1;tr|F1S1B2|F1S1B2_PIG Myelin peripheral protein OS=Sus scrofa OX=9823 GN=MPZ PE=1 SV=1
13069000 20831000 16105000 20121000 20161000 22357000 21342000 14675000 21392000 20628000
19978000 12438000 6 6 6 24,9 24,9 24,9 21,401 0 12,979
759780000 44 F1RLG5;A0A5G2RB75;A0A5G2QKS5;A0A5G2QY56;A0A5G2RDZ0
F1RLG5;A0A5G2RB75;A0A5G2QKS5;A0A5G2QY56737 tr|F1RLG5|F1RLG5_PIG Receptor expression-enhancing protein OS=Sus scrofa OX=9823 GN=REEP5 PE=1 SV=1;tr|A0A5G2RB75|A0A5G2RB75_PIG Receptor expression-enhancing protein OS=Sus scrofa OX=9823 GN=REEP5 PE=1 SV=1;tr|A0A5G2QKS5|A0A5G2QKS5_PIG Receptor expression

0 0 0 0 0 0 0 0 0 0 0 0 2 1 1 4,2 2,4 2,4
42,936 0 3,0412 1234800000 16 F1SGG1;A0A5G2RBD3 F1SGG1;A0A5G2RBD3 738
tr|F1SGG1|F1SGG1_PIG Keratin 18 OS=Sus scrofa OX=9823 GN=KRT18 PE=1 SV=4;tr|A0A5G2RBD3|A0A5G2RBD3_PIG Keratin 18 OS=Sus scrofa OX=9823 GN=KRT18 PE=1 SV=1
42762000 44241000 32735000 38893000 33398000 29407000 69279000 31132000 28624000 46767000
40061000 30537000 20 20 20 33,4 33,4 33,4 72,116 0 158,71
4204000000 169 A0A5G2RBI3;F1S0V3;A0A5G2QF50;A0A5G2R829;A0A5G2QT71
A0A5G2RBI3;F1S0V3;A0A5G2QF50;A0A5G2R829 739 tr|A0A5G2RBI3|A0A5G2RBI3_PIG Annexin OS=Sus scrofa OX=9823 GN=ANXA6 PE=1 SV=1;tr|F1S0V3|F1S0V3_PIG Annexin OS=Sus scrofa OX=9823 GN=ANXA6 PE=1 SV=4;tr|A0A5G2QF50|A0A5G2QF50_PIG Annexin OS=Sus scrofa OX=9823 GN=ANXA6 PE=1 SV=1;tr|A0A5G2R829|A0A5G2R829_PIG A
18265000 29736000 95229000 32480000 69578000 90004000 20873000 39951000 67569000 27822000
72898000 153300000 20 20 20 27,7 27,7 27,7 83,106 0 121,13
6651800000 194 Q29554;A0A5G2RBW4;A0A5G2R3M6
Q29554;A0A5G2RBW4;A0A5G2R3M6 740 sp|Q29554|ECHA_PIG Trifunctional enzyme subunit alpha, mitochondrial OS=Sus scrofa OX=9823 GN=HADHA PE=2 SV=1;tr|A0A5G2RBW4|A0A5G2RBW4_PIG Enoyl-CoA hydratase OS=Sus scrofa OX=9823 GN=HADHA PE=1 SV=1;tr|A0A5G2R3M6|A0A5G2R3M6_PIG Enoyl-CoA hydratase OS=Sus
0 0 0 0 0 0 0 0 0 0 0 0 1 1 1 5,1 5,1 5,1
25,079 0 4,5237 68582000 3 A0A5G2RC36;F1S0B9 A0A5G2RC36;F1S0B9 741
tr|A0A5G2RC36|A0A5G2RC36_PIG Eukaryotic translation initiation factor 4E OS=Sus scrofa OX=9823 GN=EIF4E PE=1 SV=1;tr|F1S0B9|F1S0B9_PIG Eukaryotic translation initiation factor 4E OS=Sus scrofa OX=9823 GN=EIF4E PE=1 SV=2
143560000 115880000 124690000 136130000 138380000 94169000 160270000
134670000 140300000 195240000 162110000 111730000 81 81
81 54,9 54,9 54,9 173,34 0 323,31 36544000000 1127
A0A5G2RCH7;A0A5K1VE56;A0A480KXA1;F1SM75;A0A5G2QUK3
A0A5G2RCH7;A0A5K1VE56;A0A480KXA1;F1SM75;A0A5G2QUK3 742
tr|A0A5G2RCH7|A0A5G2RCH7_PIG Myomesin 1 OS=Sus scrofa OX=9823 GN=MYOM1 PE=1 SV=1;tr|A0A5K1VE56|A0A5K1VE56_PIG Myomesin 1 OS=Sus scrofa OX=9823 GN=MYOM1 PE=1 SV=1;tr|A0A480KXA1|A0A480KXA1_PIG Myomesin 1 OS=Sus scrofa OX=9823 GN=MYOM1 PE=1 SV=1;tr|F1SM75|F1S
0 0 0 0 0 0 0 0 0 0 0 0 2 1 1 9,7 5,6 5,6
29,305 0 5,2539 28528000 7 A0A5G2RD82;F1SHD6;P29412
A0A5G2RD82;F1SHD6;P29412 743 tr|A0A5G2RD82|A0A5G2RD82_PIG Elongation factor 1-beta OS=Sus scrofa OX=9823 GN=EEF1B2 PE=1 SV=1;tr|F1SHD6|F1SHD6_PIG Elongation factor 1-beta OS=Sus scrofa OX=9823 GN=EEF1B2 PE=1 SV=1;sp|P29412|EF1B_PIG Elongation factor 1-beta OS=Sus scrofa OX=9823 GN=EEF
15483000 9303600 28176000 28753000 20193000 24502000 22700000 0 24850000 32410000 29296000
51375000 3 3 3 21,2 21,2 21,2 15,614 0 12,586 512870000 25
A0A5G2REA1;F1SGC6 A0A5G2REA1;F1SGC6 744 tr|A0A5G2REA1|A0A5G2REA1_PIG

Complex I-SGDH OS=Sus scrofa OX=9823 GN=NDUFB5 PE=1 SV=1;tr|F1SGC6|F1SGC6_PIG Complex I-SGDH OS=Sus scrofa OX=9823 GN=NDUFB5 PE=1 SV=1
0 0 3538800 0 0 3121000 0 0 5930400 0 0 0 2 2 2
17,2 17,2 17,2 12,965 0,0022472 2,5083 42125000 4 F1RPU8;A0A5G2REE1
F1RPU8;A0A5G2REE1 745 tr|F1RPU8|F1RPU8_PIG Mitochondrial pyruvate carrier OS=Sus scrofa OX=9823 GN=MPC2 PE=1 SV=4;tr|A0A5G2REE1|A0A5G2REE1_PIG Mitochondrial pyruvate carrier OS=Sus scrofa OX=9823 GN=MPC2 PE=1 SV=1
165270000 74191000 46746000 147030000 78249000 90394000 224430000 43908000 73390000
206830000 71208000 49741000 4 4 4 20,4 20,4 20,4 25,245 0
26,07 1918700000 56
A0A5G2REF2;A0A287B6M7;A0A5G2QM14;F1RT90;A0A287B2T8;I3LIP6;A0A287AI01;A0A287AT82 A0A5G2REF2 746 tr|A0A5G2REF2|A0A5G2REF2_PIG Aspartate beta-hydroxylase OS=Sus scrofa OX=9823 GN=ASPH PE=1 SV=1
0 0 0 0 0 0 0 0 0 0 0 0 2 2 2 3,5 3,5 3,5
62,644 0 3,6473 0 2 F1SCM9;I3LAS2;A0A5G2REI4
F1SCM9;I3LAS2;A0A5G2REI4 747 tr|F1SCM9|F1SCM9_PIG Thyroid hormone receptor interactor 10 OS=Sus scrofa OX=9823 GN=TRIP10 PE=1 SV=2;tr|I3LAS2|I3LAS2_PIG Cdc42-interacting protein 4 isoform 1 OS=Sus scrofa OX=9823 GN=TRIP10 PE=1 SV=3;tr|A0A5G2REI4|A0A5G2REI4_PIG Thyroid hormone receptor
64566000 69782000 114470000 120380000 148260000 140960000 75572000 104720000
130830000 122120000 155470000 208620000 13 13 13 38,2 38,2
38,2 47,346 0 182,75 7165600000 184 A0A5G2REY8;F1RPD2 A0A5G2REY8;F1RPD2
748 tr|A0A5G2REY8|A0A5G2REY8_PIG Ubiquinol-cytochrome c reductase core protein 2 OS=Sus scrofa OX=9823 GN=UQCRC2 PE=1 SV=1;tr|F1RPD2|F1RPD2_PIG Cytochrome b-c1 complex subunit 2, mitochondrial OS=Sus scrofa OX=9823 GN=UQCRC2 PE=1 SV=3
0 0 0 0 0 0 0 0 0 0 0 0 1 1 1 10,8 10,8 10,8
14,714 0,0032017 2,0877 11157000 3 A0A5G2RF87;F1S108 A0A5G2RF87;F1S108
749 tr|A0A5G2RF87|A0A5G2RF87_PIG CDGSH iron sulfur domain 2 OS=Sus scrofa OX=9823 GN=CISD2 PE=1 SV=1;tr|F1S108|F1S108_PIG CDGSH iron sulfur domain 2 OS=Sus scrofa OX=9823 GN=CISD2 PE=1 SV=2
282610000 232420000 519810000 811610000 455610000 504880000 689170000
272980000 502040000 909470000 438360000 720710000 16 16
16 55,3 55,3 55,3 35,484 0 221,79 42626000000 643 P00346;A0A5G2RGL7;I3LP41
P00346;A0A5G2RGL7;I3LP41 750 sp|P00346|MDHM_PIG Malate dehydrogenase, mitochondrial OS=Sus scrofa OX=9823 GN=MDH2 PE=1 SV=2;tr|A0A5G2RGL7|A0A5G2RGL7_PIG Malate dehydrogenase OS=Sus scrofa OX=9823 GN=MDH2 PE=3 SV=1;tr|I3LP41|I3LP41_PIG Malate dehydrogenase OS=Sus scrofa OX=9823 GN=MDH2
0 0 2615100 0 0 5463200 0 0 0 0 0 5134000 3 3 3
3,8 3,8 3,8 79,456 0 2,8726 43682000 7
A0A5G2RH47;F1RU52;I3LGH6;A0A5G2RLJ6 A0A5G2RH47;F1RU52;I3LGH6;A0A5G2RLJ6
751 tr|A0A5G2RH47|A0A5G2RH47_PIG Dipeptidyl peptidase 3 OS=Sus scrofa OX=9823 GN=DPP3 PE=1 SV=1;tr|F1RU52|F1RU52_PIG Dipeptidyl peptidase 3 OS=Sus scrofa OX=9823

GN=DPP3 PE=1 SV=1;tr|I3LGH6|I3LGH6_PIG Dipeptidyl peptidase 3 OS=Sus scrofa OX=9823 GN=DPP3 PE=1 S
19729000 22251000 18396000 0 21177000 14597000 13625000 10921000 24107000 19896000 10498000 14633000 10 10 10 29,4 29,4 29,4 43,266 0 43,516 703110000 47 A0A5G2RHM3;A0A5G2RH83;F1S062;A0A5G2RAQ9 A0A5G2RHM3;A0A5G2RH83;F1S062;A0A5G2RAQ9 752 tr|A0A5G2RHM3|A0A5G2RHM3_PIG Obg-like ATPase 1 OS=Sus scrofa OX=9823 GN=OLA1 PE=1 SV=1;tr|A0A5G2RH83|A0A5G2RH83_PIG Obg-like ATPase 1 OS=Sus scrofa OX=9823 GN=OLA1 PE=1 SV=1;tr|F1S062|F1S062_PIG Obg like ATPase 1 OS=Sus scrofa OX=9823 GN=OLA1 PE=1 SV=4;tr|
9851800 5048900 0 0 0 0 4003000 0 0 0 0 0 3 3 3 3,4 3,4 3,4 126,71 0 3,5122 91146000 5 A0A5G2RIC0;F1RP44 A0A5G2RIC0;F1RP44 753 tr|A0A5G2RIC0|A0A5G2RIC0_PIG Importin 5 OS=Sus scrofa OX=9823 GN=IPO5 PE=1 SV=1;tr|F1RP44|F1RP44_PIG Importin 5 OS=Sus scrofa OX=9823 GN=IPO5 PE=1 SV=3
0 0 0 0 0 0 0 0 0 0 0 0 1 1 1 6,4 6,4 6,4 18,955 0,0031348 1,9393 38146000 1 I3L8J2;A0A5G2RJ56 I3L8J2;A0A5G2RJ56 754 tr|I3L8J2|I3L8J2_PIG Galectin OS=Sus scrofa OX=9823 GN=LGALSL PE=1 SV=1;tr|A0A5G2RJ56|A0A5G2RJ56_PIG Galectin OS=Sus scrofa OX=9823 GN=LGALSL PE=1 SV=1
5863100 0 5076800 0 0 0 5806200 0 4569300 4542200 0 0 2 2 2 6,8 6,8 6,8 40,184 0 4,7092 73387000 8 A0A5G2RKS0;A0A5S6HA01;A7Y521;A0A287AXL6 A0A5G2RKS0;A0A5S6HA01;A7Y521;A0A287AXL6 755 tr|A0A5G2RKS0|A0A5G2RKS0_PIG COP9 signalosome complex subunit 4 OS=Sus scrofa OX=9823 GN=COPS4 PE=1 SV=1;tr|A0A5S6HA01|A0A5S6HA01_PIG COP9 signalosome complex subunit 4 OS=Sus scrofa OX=9823 GN=COPS4 PE=1 SV=1;sp|A7Y521|CSN4_PIG COP9 signalosome complex su
0 0 0 0 0 0 0 0 0 0 0 0 3 3 3 32,1 32,1 32,1 17,936 0 8,8618 694020000 7 A0A5G2RL03;F1S7V6;A0A5G2R2Q9 A0A5G2RL03;F1S7V6;A0A5G2R2Q9 756 tr|A0A5G2RL03|A0A5G2RL03_PIG Protein phosphatase 1 regulatory inhibitor subunit 14C OS=Sus scrofa OX=9823 GN=PPP1R14C PE=1 SV=1;tr|F1S7V6|F1S7V6_PIG Protein phosphatase 1 regulatory inhibitor subunit 14C OS=Sus scrofa OX=9823 GN=PPP1R14C PE=1 SV=2;tr|A0A5G
0 0 0 0 0 0 0 0 0 0 0 0 13 1 1 36,2 2,2 2,2 40,743 0 3,0771 1530200000 26 A0A5K1U1D3 A0A5K1U1D3 757 tr|A0A5K1U1D3|A0A5K1U1D3_PIG Four and a half LIM domains 1 OS=Sus scrofa OX=9823 GN=FHL1 PE=1 SV=1
8182200 15798000 18216000 0 7067800 0 5812300 4573100 3054000 0 3492400 0 8 8 8 8,9 8,9 8,9 113,71 0 39,481 328060000 22 A0A5K1U589;A0A5G2QCS5;A0A287A3T5 A0A5K1U589;A0A5G2QCS5;A0A287A3T5 758 tr|A0A5K1U589|A0A5K1U589_PIG Ubiquitin-activating enzyme E1 OS=Sus scrofa OX=9823 GN=UBA1 PE=1 SV=1;tr|A0A5G2QCS5|A0A5G2QCS5_PIG Ubiquitin-activating enzyme E1 OS=Sus scrofa OX=9823 GN=LOC110257910 PE=1 SV=1;tr|A0A287A3T5|A0A287A3T5_PIG Ubiquitin-activatin

111400000	116500000	83415000 162470000	90351000 98307000 169590000	86066000
114450000	158120000	90015000 83688000	4	4	1	19,3 19,3 4,1
22,226	0	24,129	3779700000	87	A0A5K1U735 A0A5K1U735 759
tr|A0A5K1U735|A0A5K1U735_PIG Reticulon OS=Sus scrofa OX=9823 GN=RTN4 PE=1 SV=1
0	0	0	0	0	0	0	0	0	0	0	0	3	3	3	10,5 10,5 10,5
37,536	0	5,2611	39137000 5	A0A5K1UKA4;F1RV28 A0A5K1UKA4;F1RV28 760
tr|A0A5K1UKA4|A0A5K1UKA4_PIG Mitogen-activated protein kinase kinase 6 OS=Sus scrofa OX=9823 GN=MAP2K6 PE=1 SV=1;tr|F1RV28|F1RV28_PIG Mitogen-activated protein kinase kinase 6 OS=Sus scrofa OX=9823 GN=MAP2K6 PE=1 SV=2
10589000 0	8988200	12062000 0	0	11712000 0	0	15018000 0	8902700
3	3	3	13,1 13,1 13,1 32,117	0	6,9223	144980000	17	B8Y4S5;A0A5K1VFF8
B8Y4S5;A0A5K1VFF8	761	tr|B8Y4S5|B8Y4S5_PIG Sarcoglycan delta OS=Sus scrofa OX=9823 GN=SGCD PE=1 SV=1;tr|A0A5K1VFF8|A0A5K1VFF8_PIG Sarcoglycan delta OS=Sus scrofa OX=9823 GN=SGCD PE=1 SV=1
0	0	0	0	0	0	0	0	0	0	0	0	2	2	2	10,8 10,8 10,8
19,835	0,0022396	2,4795	32441000 9	A0A5K1VIT7;I3LQ80	A0A5K1VIT7;I3LQ80
762	tr|A0A5K1VIT7|A0A5K1VIT7_PIG Vitamin-K-epoxide reductase (warfarin-sensitive) OS=Sus scrofa OX=9823 GN=VKORC1L1 PE=1 SV=1;tr|I3LQ80|I3LQ80_PIG Vitamin-K-epoxide reductase (warfarin-sensitive) OS=Sus scrofa OX=9823 GN=VKORC1L1 PE=1 SV=3
10572000 16216000 10299000 16388000 11692000 9771400	19481000 7543300	10178000 11995000
10876000 10581000	7	7	7	9,2	9,2	9,2	81,738	0	13,614
522190000	45	P35750;A0A5S6GBZ7;A0A5S6H4P7;A0A5G2QYC6
P35750;A0A5S6GBZ7;A0A5S6H4P7;A0A5G2QYC6	763	sp|P35750|CAN1_PIG Calpain-1 catalytic subunit OS=Sus scrofa OX=9823 GN=CAPN1 PE=2 SV=3;tr|A0A5S6GBZ7|A0A5S6GBZ7_PIG Calcium-activated neutral proteinase 1 OS=Sus scrofa OX=9823 GN=CAPN1 PE=1 SV=1;tr|A0A5S6H4P7|A0A5S6H4P7_PIG Calcium-activated neutral prot
35901000 38341000 33256000 0	36507000 13960000 26370000 30967000 35288000 46607000 23458000
7254500	3	3	3	18,1 18,1 18,1 21,155	0	11,891	672520000	43
A0A5S6GER6;Q5PYH3;A0A5S6G7K0;Q52NJ3	A0A5S6GER6;Q5PYH3 764
tr|A0A5S6GER6|A0A5S6GER6_PIG GTP-binding protein SAR1b OS=Sus scrofa OX=9823 GN=SAR1B PE=1 SV=1;sp|Q5PYH3|SAR1B_PIG GTP-binding protein SAR1b OS=Sus scrofa OX=9823 GN=SAR1B PE=2 SV=1
34724000 23862000 13527000 26646000 20530000 16879000 30710000 15049000 13207000 28347000
20003000 20309000	4	4	4	16,9 16,9 16,9 28,068	0	11,572
717860000	29	P04574;A0A5S6GT88;A0A5G2RH51	P04574;A0A5S6GT88	765
sp|P04574|CPNS1_PIG Calpain small subunit 1 OS=Sus scrofa OX=9823 GN=CAPNS1 PE=1 SV=1;tr|A0A5S6GT88|A0A5S6GT88_PIG Calcium-activated neutral proteinase small subunit OS=Sus scrofa OX=9823 GN=CAPNS1 PE=4 SV=1
0	0	0	0	0	0	0	0	0	0	0	0	2	2	2	3	3	3
86,121	0	3,5251	5375500	2	Q8HZV3;A0A5S6GXI4	Q8HZV3;A0A5S6GXI4	766
sp|Q8HZV3|TFR1_PIG Transferrin receptor protein 1 OS=Sus scrofa OX=9823 GN=TFRC PE=2

SV=1;tr|A0A5S6GXI4|A0A5S6GXI4_PIG Transferrin receptor protein 1 OS=Sus scrofa OX=9823 GN=TFRC PE=1 SV=1
36048000 0 71828000 100840000 84735000 66873000 87440000 46662000 60744000 91776000 81479000 128180000 6 6 6 15,5 15,5 15,5 33,02 0 16,947 2219300000 69 A0A5S6HDE4;P00348;A0A5G2QZU9;A0A2C9F3C3 A0A5S6HDE4;P00348;A0A5G2QZU9;A0A2C9F3C3 767 tr|A0A5S6HDE4|A0A5S6HDE4_PIG Hydroxyacyl-coenzyme A dehydrogenase, mitochondrial OS=Sus scrofa OX=9823 GN=HADH PE=1 SV=1;sp|P00348|HCDH_PIG Hydroxyacyl-coenzyme A dehydrogenase, mitochondrial OS=Sus scrofa OX=9823 GN=HADH PE=1 SV=2;tr|A0A5G2QZU9|A0A5G2QZU9
48634000 47646000 384470000 28891000 161260000 342910000 32150000 61610000 136550000 36602000 162600000 556150000 36 24 21 34,8 22,7 19,1 110,59 0 276,94 18759000000 398 A0A5S6HSJ6;A0A5S6HET4;P11607 A0A5S6HSJ6;A0A5S6HET4;P11607 768 tr|A0A5S6HSJ6|A0A5S6HSJ6_PIG Calcium-transporting ATPase OS=Sus scrofa OX=9823 GN=ATP2A2 PE=1 SV=1;tr|A0A5S6HET4|A0A5S6HET4_PIG Calcium-transporting ATPase OS=Sus scrofa OX=9823 GN=ATP2A2 PE=1 SV=1;sp|P11607|AT2A2_PIG Sarcoplasmic/endoplasmic reticulum cal
47175000 9967500 25917000 37124000 8495500 60329000 44031000 7735500 16554000 37503000 13268000 36551000 14 14 14 39,3 39,3 39,3 39,1 0 56,375 1573700000 79 A0A5S6HM44;Q8SPS7;A0A2C9F356 A0A5S6HM44;Q8SPS7;A0A2C9F356 769 tr|A0A5S6HM44|A0A5S6HM44_PIG Haptoglobin OS=Sus scrofa OX=9823 GN=HP PE=3 SV=1;sp|Q8SPS7|HPT_PIG Haptoglobin OS=Sus scrofa OX=9823 GN=HP PE=1 SV=1;tr|A0A2C9F356|A0A2C9F356_PIG Haptoglobin OS=Sus scrofa OX=9823 GN=HP PE=4 SV=2
6254500 9470800 11998000 0 7015600 0 0 0 4165100 0 0 6145200 6 6 6 20 20 20 49,023 0 13,169 130230000 18 P10775;A0A5S6IIV1;A0A5S6HM99;A0A5S6HMU1 P10775;A0A5S6IIV1;A0A5S6HM99;A0A5S6HMU1 770 sp|P10775|RINI_PIG Ribonuclease inhibitor OS=Sus scrofa OX=9823 GN=RNH1 PE=1 SV=1;tr|A0A5S6IIV1|A0A5S6IIV1_PIG Ribonuclease inhibitor OS=Sus scrofa OX=9823 GN=RNH1 PE=4 SV=1;tr|A0A5S6HM99|A0A5S6HM99_PIG Ribonuclease inhibitor OS=Sus scrofa OX=9823 GN=RNH1
13892000 11610000 15840000 14505000 13984000 20627000 14018000 0 16513000 20838000 13339000 20515000 4 4 4 17,8 17,8 17,8 35,158 0 18,847 456890000 41 P05027;A0A5S6HMA0 P05027;A0A5S6HMA0 771 sp|P05027|AT1B1_PIG Sodium/potassium-transporting ATPase subunit beta-1 OS=Sus scrofa OX=9823 GN=ATP1B1 PE=1 SV=2;tr|A0A5S6HMA0|A0A5S6HMA0_PIG Sodium/potassium-transporting ATPase subunit beta OS=Sus scrofa OX=9823 GN=ATP1B1 PE=1 SV=1
16450000 17722000 9945900 13394000 0 6427300 11654000 31105000 22380000 32426000 0 10055000 4 4 4 47,7 47,7 47,7 9,3457 0 38,65 303710000 19 Q0MUU2;A0A5S6HUN0 Q0MUU2;A0A5S6HUN0 772 sp|Q0MUU2|SMPX_PIG Small muscular protein OS=Sus scrofa OX=9823 GN=SMPX PE=2 SV=1;tr|A0A5S6HUN0|A0A5S6HUN0_PIG Small muscular protein OS=Sus scrofa OX=9823 GN=SMPX PE=3 SV=1

0 108610000 59836000 173700000 87355000 61589000 193090000 192240000 105580000 75120000 83486000 70777000 4 4 4 48,3 48,3 48,3 9,8962 0 6,9764 1785500000 20 P12026;A0A5S6I0G9 P12026;A0A5S6I0G9 773 sp|P12026|ACBP_PIG Acyl-CoA-binding protein OS=Sus scrofa OX=9823 GN=DBI PE=1 SV=2;tr|A0A5S6I0G9|A0A5S6I0G9_PIG Acyl-CoA-binding protein OS=Sus scrofa OX=9823 GN=DBI PE=4 SV=1

14106000 10987000 13226000 8852600 10892000 7100200 16051000 6146800 7709400 12249000 7224200 10670000 4 4 2 12,5 12,5 6,6 34,448 0 9,6583 353030000 29 A0A5S8KL32;P61292;A0A5S6I1J4;A0A5G2R6F8;A0A5G2QYQ0;A0A5G2QI00 A0A5S8KL32;P61292;A0A5S6I1J4;A0A5G2R6F8;A0A5G2QYQ0;A0A5G2QI00 774 tr|A0A5S8KL32|A0A5S8KL32_PIG Serine/threonine-protein phosphatase OS=Sus scrofa OX=9823 GN=PPP1CB PE=1 SV=1;sp|P61292|PP1B_PIG Serine/threonine-protein phosphatase PP1-beta catalytic subunit OS=Sus scrofa OX=9823 GN=PPP1CB PE=1 SV=3;tr|A0A5S6I1J4|A0A5S6I1J

85580000 47988000 113420000 125460000 61678000 137780000 110570000 47084000 88625000 130140000 59574000 107700000 3 3 3 36,5 36,5 36,5 9,557 0 17,803 2165600000 34 A0A5S6I2J5;P82460;A0A2C9F3A7;A0A5G2Q9S8;A0A5G2QTF5 A0A5S6I2J5;P82460;A0A2C9F3A7;A0A5G2Q9S8 775 tr|A0A5S6I2J5|A0A5S6I2J5_PIG Thioredoxin OS=Sus scrofa OX=9823 GN=TXN PE=4 SV=1;sp|P82460|THIO_PIG Thioredoxin OS=Sus scrofa OX=9823 GN=TXN PE=1 SV=3;tr|A0A2C9F3A7|A0A2C9F3A7_PIG Thioredoxin OS=Sus scrofa OX=9823 GN=TXN PE=3 SV=2;tr|A0A5G2Q9S8|A0A5G2Q9S8_P

19903000 20470000 13445000 23561000 14821000 17727000 32084000 15287000 16799000 26833000 19218000 19130000 3 3 3 13,4 13,4 13,4 23,197 0 5,7988 367660000 38 A0A5S6I5L2;Q56P28;A0A5G2QS88 A0A5S6I5L2;Q56P28;A0A5G2QS88 776 tr|A0A5S6I5L2|A0A5S6I5L2_PIG PRA1 family protein OS=Sus scrofa OX=9823 GN=ARL6IP5 PE=1 SV=1;sp|Q56P28|PRAF3_PIG PRA1 family protein 3 OS=Sus scrofa OX=9823 GN=ARL6IP5 PE=2 SV=1;tr|A0A5G2QS88|A0A5G2QS88_PIG PRA1 family protein OS=Sus scrofa OX=9823 GN=ARL6I

29709000 36170000 35982000 25546000 35377000 38404000 31159000 25738000 32615000 35108000 31990000 31953000 4 4 4 19,8 19,8 19,8 29,813 0 6,8498 654580000 12 A0A5S6IDI6;Q9GJT2;A0A5G2QPR4 A0A5S6IDI6;Q9GJT2;A0A5G2QPR4 777 tr|A0A5S6IDI6|A0A5S6IDI6_PIG S-formylglutathione hydrolase OS=Sus scrofa OX=9823 GN=ESD PE=1 SV=1;sp|Q9GJT2|ESTD_PIG S-formylglutathione hydrolase OS=Sus scrofa OX=9823 GN=ESD PE=2 SV=1;tr|A0A5G2QPR4|A0A5G2QPR4_PIG S-formylglutathione hydrolase OS=Sus scro

27567000 38834000 62657000 37532000 70104000 58355000 24563000 43773000 41807000 38681000 63521000 83752000 16 16 16 39,1 39,1 39,1 49,97 0 62,512 3917000000 142 A0A5S8M9X9;P10173;I3LPP1;A0A286ZIY0 A0A5S8M9X9;P10173;I3LPP1;A0A286ZIY0 778 tr|A0A5S8M9X9|A0A5S8M9X9_PIG Fumarate hydratase, mitochondrial OS=Sus scrofa OX=9823 GN=FH PE=3 SV=1;sp|P10173|FUMH_PIG Fumarate hydratase, mitochondrial OS=Sus scrofa OX=9823 GN=FH PE=1 SV=2;tr|I3LPP1|I3LPP1_PIG Fumarate hydratase, mitochondrial OS=Sus sc

9049300 7846500 10674000 6707400 8389800 11008000 8899800 0 8001400 0 9997900
19040000 4 4 4 30,5 30,5 30,5 13,338 0 7,5938 262350000 20
F1SBU8;A1XQR7 F1SBU8;A1XQR7 779 tr|F1SBU8|F1SBU8_PIG MICOS complex subunit MIC13 OS=Sus scrofa OX=9823 GN=MICOS13 PE=1 SV=1;sp|A1XQR7|MIC13_PIG MICOS complex subunit MIC13 OS=Sus scrofa OX=9823 GN=MICOS13 PE=3 SV=1
85657000 54939000 149110000 194860000 153960000 154180000 156230000 67557000
122440000 218300000 152170000 261700000 5 5 5 59,2 59,2
59,2 8,8001 0 19,314 6713400000 130 A1XQT2 A1XQT2 780
sp|A1XQT2|COX6C_PIG Cytochrome c oxidase subunit 6C OS=Sus scrofa OX=9823 GN=COX6C PE=3 SV=1
38347000 58149000 28490000 15765000 18187000 27025000 16264000 32797000 45581000 18086000
27932000 28547000 15 15 1 37,4 37,4 2,8 50,098 0 85,928
1774000000 96 A4Z6H0 A4Z6H0 781 sp|A4Z6H0|PURA1_PIG Adenylosuccinate synthetase isozyme 1 OS=Sus scrofa OX=9823 GN=ADSS1 PE=2 SV=1
5804700 8386800 13698000 0 7819700 7714200 0 0 9147700 0 6897800 6890600
4 4 4 4 4 4 107,47 0 4,5971 245380000 14
A5GFU0;A5GFT9;P29797;A5GFU7;A5GFU5;A0A5G2QYC4;Q06AS6;I3LRX8;I3LK93;K7GQA7;A0A5G2QDJ3;F1SB90;Q06AS8;A0A287B6X9;A0A287A9G3;F1S603;A0A287BRP2;A0A5G2R5X2;F1SB91 A5GFU0;A5GFT9;P29797;A5GFU7;A5GFU5;A0A5G2QYC4 782 tr|A5GFU0|A5GFU0_PIG GNAS complex locus OS=Sus scrofa OX=9823 GN=GNAS PE=1 SV=1;tr|A5GFT9|A5GFT9_PIG GNAS complex locus OS=Sus scrofa OX=9823 GN=GNAS PE=1 SV=1;sp|P29797|GNAS_PIG Guanine nucleotide-binding protein G(s) subunit alpha OS=Sus scrofa OX=9823 G
3667300000 4495300000 1460400000 4277300000 3578300000 2008600000 4188200000
3636900000 2231200000 3024200000 3574600000 1099900000 18 18
14 55,7 55,7 43,5 28,677 0 221,08 142860000000 1359 B5KJG2 B5KJG2 783
tr|B5KJG2|B5KJG2_PIG Phosphoglycerate mutase OS=Sus scrofa OX=9823 GN=PGAM2 PE=1 SV=1
8544400 6268000 6344000 7440800 7067600 10437000 8742100 0 6460900 7666000 6533900
6909200 5 5 5 21,2 21,2 21,2 25,206 0 9,9935 300820000 20
B6E241;A0A5G2QN78;A0A5G2QG04 B6E241;A0A5G2QN78 784 tr|B6E241|B6E241_PIG Growth factor receptor bound protein 2 OS=Sus scrofa OX=9823 GN=GRB2 PE=1 SV=1;tr|A0A5G2QN78|A0A5G2QN78_PIG Growth factor receptor bound protein 2 OS=Sus scrofa OX=9823 GN=GRB2 PE=1 SV=1
0 0 0 0 0 0 0 0 0 0 0 0 1 1 1 4,4 4,4 4,4
31,284 0,0087125 1,4249 0 1 C5H0C6 C5H0C6 785 tr|C5H0C6|C5H0C6_PIG Ubiquitin thioesterase OS=Sus scrofa OX=9823 GN=OTUB1 PE=1 SV=1
0 0 13886000 0 0 0 0 0 0 0 0 28597000 5 5 5 13,1
13,1 13,1 42,048 0 7,1344 129410000 9 F6PZX4;D0G0B3;A0A286ZXB9
F6PZX4;D0G0B3;A0A286ZXB9 802 tr|F6PZX4|F6PZX4_PIG Acetyl-CoA acyltransferase 2 OS=Sus scrofa OX=9823 GN=ACAA2 PE=1 SV=2;tr|D0G0B3|D0G0B3_PIG 3-ketoacyl-CoA thiolase, mitochondrial OS=Sus scrofa OX=9823 GN=ACAA2 PE=1 SV=1;tr|A0A286ZXB9|A0A286ZXB9_PIG Acetyl-CoA acyltransferase 2 OS=Sus

11157000 5812500 0 0 6412100 6343600 9894600 0 0 8084700 0 0
4 4 4 23,7 23,7 23,7 22,16 0 39,458 134340000 17 F1REW9 F1REW9 803
tr|F1REW9|F1REW9_PIG Density-regulated protein OS=Sus scrofa OX=9823 GN=DENR PE=1 SV=3
0 0 18984000 33202000 24916000 0 24432000 0 0 31933000 0 0 2
2 2 43,1 43,1 43,1 6,4255 0 42,318 368470000 18 F1RFD4 F1RFD4 804
tr|F1RFD4|F1RFD4_PIG ATP synthase membrane subunit DAPIT OS=Sus scrofa OX=9823 GN=ATP5MK PE=1 SV=1
1688800000 2119100000 1511700000 634940000 1476900000 1897800000 626310000
1524800000 1713200000 858010000 1349500000 924140000 46 46
29 44,5 44,5 28,3 110,1 0 323,31 225370000000 2490
F1RFH9;A0A287APK5;A0A5G2R940;A0A287AL21
F1RFH9;A0A287APK5;A0A5G2R940;A0A287AL21 805 tr|F1RFH9|F1RFH9_PIG Calcium-transporting ATPase OS=Sus scrofa OX=9823 GN=ATP2A1 PE=1 SV=3;tr|A0A287APK5|A0A287APK5_PIG Calcium-transporting ATPase OS=Sus scrofa OX=9823 GN=ATP2A1 PE=1 SV=2;tr|A0A5G2R940|A0A5G2R940_PIG Calcium-transporting ATPase OS=Sus sc
5077500 6159300 23285000 0 0 0 0 0 0 0 0 0 3 3 3
5,8 5,8 5,8 61,398 0 8,1853 67484000 5 F1RFI1 F1RFI1 806
tr|F1RFI1|F1RFI1_PIG Elongation factor Tu OS=Sus scrofa OX=9823 GN=TUFM PE=1 SV=2
25573000 17526000 16827000 32942000 22886000 23658000 38738000 18015000 14822000 22270000
20576000 18905000 3 3 3 12,9 12,9 12,9 25,692 0 6,3962
533380000 23 F1RFV5;A0A5G2R4Y9 F1RFV5 807 tr|F1RFV5|F1RFV5_PIG Proteasome subunit beta OS=Sus scrofa OX=9823 GN=PSMB6 PE=1 SV=2
0 0 0 0 0 0 0 0 0 0 0 0 1 1 1 7,1 7,1 7,1
19,077 0 3,0319 445800000 3 F1RG17 F1RG17 808 tr|F1RG17|F1RG17_PIG Nucleoside diphosphate kinase OS=Sus scrofa OX=9823 GN=NME3 PE=1 SV=3
28616000 15206000 45626000 42014000 35941000 44957000 38131000 24188000 42193000 57363000
41472000 80305000 6 6 6 50,5 50,5 50,5 11,082 0 31,7 1285100000
79 F1RGE3;A0A5G2R7U7;A0A5G2QV68 F1RGE3;A0A5G2R7U7;A0A5G2QV68 809
tr|F1RGE3|F1RGE3_PIG NADH dehydrogenase [ubiquinone] 1 alpha subcomplex subunit 2 OS=Sus scrofa OX=9823 GN=NDUFA2 PE=1 SV=1;tr|A0A5G2R7U7|A0A5G2R7U7_PIG Complex I-B8 OS=Sus scrofa OX=9823 GN=NDUFA2 PE=1 SV=1;tr|A0A5G2QV68|A0A5G2QV68_PIG Complex I-B8 OS=Sus
0 0 0 0 0 0 0 0 0 0 0 0 17 1 1 49,2 5,5 5,5
29,357 0 18,576 12545000 2
F1RGK5;A0A5G2RD45;A0A5G2RA93;A0A5G2QIK0;A0A288CFY3
F1RGK5;A0A5G2RD45;A0A5G2RA93;A0A5G2QIK0;A0A288CFY3 810
tr|F1RGK5|F1RGK5_PIG Tropomyosin alpha-3 chain OS=Sus scrofa OX=9823 GN=TPM3 PE=1 SV=3;tr|A0A5G2RD45|A0A5G2RD45_PIG Tropomyosin alpha-3 chain OS=Sus scrofa OX=9823 GN=TPM3 PE=1 SV=1;tr|A0A5G2RA93|A0A5G2RA93_PIG Tropomyosin alpha-3 chain OS=Sus scrofa OX=98

0 0 0 0 0 0 0 0 0 0 0 0 1 1 1 16,5 16,5 16,5
9,4145 0 9,5202 3306400 2 F1RGW3 F1RGW3 811 tr|F1RGW3|F1RGW3_PIG
Uncharacterized protein OS=Sus scrofa OX=9823 GN=COA6 PE=4 SV=2
18369000 0 44320000 7186300 4588700 51008000 0 16672000 16451000 7047100 12963000
17916000 24 18 18 29,8 22,9 22,9 103,73 0 64,008 1056500000 65
F1RHL9;A0A287AU97;A0A5G2QP94;A0A5G2QN32;I3LCP0;A0A5G2QLU2;A0A287BIH3
F1RHL9;A0A287AU97;A0A5G2QP94;A0A5G2QN32;I3LCP0;A0A5G2QLU2;A0A287BIH3 813
tr|F1RHL9|F1RHL9_PIG Actinin alpha 2 OS=Sus scrofa OX=9823 GN=ACTN2 PE=1 SV=1;tr|A0A287AU97|A0A287AU97_PIG Actinin alpha 2 OS=Sus scrofa OX=9823 GN=ACTN2 PE=1 SV=2;tr|A0A5G2QP94|A0A5G2QP94_PIG Actinin alpha 2 OS=Sus scrofa OX=9823 GN=ACTN2 PE=1 SV=1;tr|A0A
0 3809200 5450600 4134900 9468600 9421400 0 3321200 4461400 4192300 5808000
14548000 2 2 2 12,2 12,2 12,2 9,7672 0 5,4904 142150000 20
F1RI18 F1RI18 814 tr|F1RI18|F1RI18_PIG Complex III subunit 8 OS=Sus scrofa OX=9823 GN=UQCRQ PE=1 SV=1
31103000 35097000 19670000 53901000 23099000 17156000 43582000 22493000 38401000 41277000
17415000 11788000 4 4 4 12,8 12,8 12,8 26,11 0 23,104
749500000 30 F1RIA7;A0A5G2RAI4 F1RIA7;A0A5G2RAI4 815 tr|F1RIA7|F1RIA7_PIG Transmembrane protein 109 OS=Sus scrofa OX=9823 GN=TMEM109 PE=1 SV=1;tr|A0A5G2RAI4|A0A5G2RAI4_PIG Transmembrane protein 109 OS=Sus scrofa OX=9823 GN=TMEM109 PE=1 SV=1
11666000 7909200 11085000 25402000 10131000 11119000 13729000 10374000 14697000 9324300
10706000 11341000 8 8 8 6,9 6,9 6,9 126,84 0 10,104
338180000 26 F1RIE2 F1RIE2 816 tr|F1RIE2|F1RIE2_PIG DNA damage-binding protein 1 OS=Sus scrofa OX=9823 GN=DDB1 PE=1 SV=3
999250000 376470000 1124500000 1300000000 605900000 2005200000 960210000
524480000 806830000 1482400000 714140000 921340000 9 9
9 70,7 70,7 70,7 16,166 0 137,09 56201000000 485
F1RII7;P02067;A0A5G2QRW3;F1RII5 F1RII7;P02067;A0A5G2QRW3 817
tr|F1RII7|F1RII7_PIG Hemoglobin subunit beta OS=Sus scrofa OX=9823 GN=HBB PE=1 SV=1;sp|P02067|HBB_PIG Hemoglobin subunit beta OS=Sus scrofa OX=9823 GN=HBB PE=1 SV=3;tr|A0A5G2QRW3|A0A5G2QRW3_PIG Hemoglobin subunit beta OS=Sus scrofa OX=9823 GN=HBB PE=1 SV=1
5322900 6575200 0 9075000 0 5931700 14724000 0 4102300 11916000 0 5674500
5 5 5 22,8 22,8 22,8 28,745 0 11,207 148640000 14 F1RJJ3 F1RJJ3
818 tr|F1RJJ3|F1RJJ3_PIG Ribosome maturation protein SBDS OS=Sus scrofa OX=9823 GN=SBDS PE=1 SV=1
137200000 187210000 203640000 26810000 131430000 109550000 39211000
117760000 119500000 42716000 128970000 149690000 16 16 16
24 24 24 86,624 0 138,38 7695000000 209 F1RK48;A0A287BAE0
F1RK48;A0A287BAE0 819 tr|F1RK48|F1RK48_PIG Sarcalumenin isoform X1 OS=Sus scrofa OX=9823 GN=SRL PE=1 SV=3;tr|A0A287BAE0|A0A287BAE0_PIG Dynamin-type G domain-containing protein OS=Sus scrofa OX=9823 GN=SRL PE=1 SV=2

607130000 324240000 259400000 842520000 325050000 368240000 894080000
252310000 283180000 814910000 274650000 242410000 10 10
10 75,4 75,4 75,4 20,971 0 89,376 20303000000 193 F1RKG8 F1RKG8 820
tr|F1RKG8|F1RKG8_PIG Uncharacterized protein OS=Sus scrofa OX=9823 GN=PEBP1 PE=1 SV=1
0 0 0 0 0 0 0 0 0 0 0 0 1 1 1 6,3 6,3 6,3
13,725 0,0049407 1,5325 430350000 20 F1RKI3 F1RKI3 821
tr|F1RKI3|F1RKI3_PIG Histidine triad nucleotide binding protein 1 OS=Sus scrofa OX=9823 GN=HINT1 PE=1 SV=1
0 0 0 0 0 0 0 0 0 0 0 0 4 1 1 10,2 2,5 2,5
55,347 0,0011561 2,7785 38942000 1 F1RL74;I3LNG5;A0A287BAW1
F1RL74;I3LNG5 822 tr|F1RL74|F1RL74_PIG Calcium/calmodulin-dependent protein kinase OS=Sus scrofa OX=9823 GN=CAMK2A PE=1 SV=3;tr|I3LNG5|I3LNG5_PIG Calcium/calmodulin-dependent protein kinase OS=Sus scrofa OX=9823 GN=CAMK2A PE=1 SV=2
0 0 0 0 0 0 0 0 0 0 0 0 3 1 1 9,4 3,5 3,5
44,272 0 3,2373 55668000 5 F1RM74 F1RM74 823 tr|F1RM74|F1RM74_PIG
Glyceraldehyde-3-phosphate dehydrogenase OS=Sus scrofa OX=9823 GN=GAPDHS PE=1 SV=3
0 0 0 0 0 0 0 0 0 0 0 0 2 2 2 3,7 3,7 3,7
89,601 0 4,5419 47566000 5 F1RMJ9 F1RMJ9 824 tr|F1RMJ9|F1RMJ9_PIG
Oxysterol-binding protein OS=Sus scrofa OX=9823 GN=OSBP PE=1 SV=2
27257000 35351000 14077000 21487000 20823000 23165000 18008000 24591000 19732000 22311000
21631000 13616000 7 7 7 15,9 15,9 15,9 52,447 0 14,02
915440000 38 F1RN71;A0A5G2R8F5;A0A5G2QTS8 F1RN71;A0A5G2R8F5 825
tr|F1RN71|F1RN71_PIG Bleomycin hydrolase OS=Sus scrofa OX=9823 GN=BLMH PE=1 SV=4;tr|A0A5G2R8F5|A0A5G2R8F5_PIG Uncharacterized protein OS=Sus scrofa OX=9823 GN=BLMH PE=1 SV=1
0 0 0 0 0 0 0 0 0 0 0 0 1 1 1 6,5 6,5 6,5
18,293 0 4,5237 77151000 11 F1RNI5 F1RNI5 826 tr|F1RNI5|F1RNI5_PIG
Complex I-B9 OS=Sus scrofa OX=9823 GN=NDUFA3 PE=1 SV=2
42574000 50432000 78965000 43424000 90026000 84974000 29358000 65188000 74251000 43229000
114680000 156830000 7 7 7 19,3 19,3 19,3 31,779 0 44,358
2788700000 78 F1RNZ1 F1RNZ1 827 tr|F1RNZ1|F1RNZ1_PIG Rieske domain-containing protein OS=Sus scrofa OX=9823 GN=UQCRFS1 PE=1 SV=2
0 0 0 0 0 0 0 0 0 0 0 0 2 2 2 5,6 5,6 5,6
45,703 0 4,7931 35746000 3 F1RP05 F1RP05 828 tr|F1RP05|F1RP05_PIG DnaJ heat shock protein family (Hsp40) member A2 OS=Sus scrofa OX=9823 GN=DNAJA2 PE=1 SV=1
1067500000 1384600000 675660000 1195600000 1045400000 853920000 1288900000
1116700000 1162300000 806410000 986130000 511320000 28 28
23 65 65 51,1 44,558 0 323,31 120670000000 1374
Q7SIB7;F1RPH0;A0A5G2QJC3;K7GKJ8;A0A5G2R4C4
Q7SIB7;F1RPH0;A0A5G2QJC3;K7GKJ8;A0A5G2R4C4 829 sp|Q7SIB7|PGK1_PIG
Phosphoglycerate kinase 1 OS=Sus scrofa OX=9823 GN=PGK1 PE=1 SV=3;tr|F1RPH0|F1RPH0_PIG Phosphoglycerate kinase OS=Sus scrofa OX=9823 GN=PGK1 PE=1

SV=3;tr|A0A5G2QJC3|A0A5G2QJC3_PIG Phosphoglycerate kinase OS=Sus scrofa OX=9823 GN=PGK1 PE=

11794000 71146000 28633000 0 9908800 3758900 5992300 15353000 5084100 0 10398000

7584700 15 15 15 14,2 14,2 14,2 137,25 0 47,105 635260000 40

F1RPM0;I3LVF3;I3L8R9;A0A286ZYC0;A0A287AHM2;A0A287BNP7;A0A5G2R6P7;A0A5G2RAA1;F1SQP0;A0A286ZQP9;A0A287B6P0

F1RPM0;I3LVF3;I3L8R9;A0A286ZYC0;A0A287AHM2;A0A287BNP7 830

tr|F1RPM0|F1RPM0_PIG Phosphorylase b kinase regulatory subunit OS=Sus scrofa OX=9823 GN=PHKA1 PE=1 SV=2;tr|I3LVF3|I3LVF3_PIG Phosphorylase b kinase regulatory subunit OS=Sus scrofa OX=9823 GN=PHKA1 PE=1 SV=2;tr|I3L8R9|I3L8R9_PIG Phosphorylase b kinase regu

25216000 27966000 26401000 0 15428000 0 7565400 15435000 17633000 9736100 9665100

7330800 11 11 11 24,8 24,8 24,8 49,954 0 41,097 598030000 45

F1RPW9;Q29387 F1RPW9;Q29387 831 tr|F1RPW9|F1RPW9_PIG Elongation factor 1-gamma OS=Sus scrofa OX=9823 GN=EEF1G PE=4 SV=3;sp|Q29387|EF1G_PIG Elongation factor 1-gamma (Fragment) OS=Sus scrofa OX=9823 GN=EEF1G PE=2 SV=2

0 0 0 0 0 0 0 0 0 0 0 0 2 2 2 8,4 8,4 8,4

28,141 0,0021858 2,2845 4620200 2 F1RQZ4 F1RQZ4 832

tr|F1RQZ4|F1RQZ4_PIG Coiled-coil domain-containing protein 43 OS=Sus scrofa OX=9823 GN=CCDC43 PE=3 SV=2

0 0 0 0 0 0 0 0 0 0 0 0 1 1 1 1,2 1,2 1,2

77,166 0,0086372 1,3912 191980000 7 F1RSG0 F1RSG0 833

tr|F1RSG0|F1RSG0_PIG Kinesin-like protein OS=Sus scrofa OX=9823 GN=KIF2B PE=3 SV=2

0 0 0 0 0 0 0 0 0 0 0 0 2 2 2 5,1 5,1 5,1

40,75 0,003178 2,0355 21557000 12 F1RTY6 F1RTY6 834 tr|F1RTY6|F1RTY6_PIG Peptidylprolyl isomerase D OS=Sus scrofa OX=9823 GN=PPID PE=1 SV=1

0 0 0 0 0 0 0 0 0 0 0 0 2 2 2 12,1 12,1 12,1

15,184 0,0022321 2,4565 156060000 5 F1RUE0 F1RUE0 835

tr|F1RUE0|F1RUE0_PIG Acyl-CoA thioesterase 13 OS=Sus scrofa OX=9823 GN=ACOT13 PE=1 SV=2

0 0 0 0 0 0 0 0 0 0 0 0 2 2 2 2,5 2,5 2,5

88,265 0,0021598 2,151 12354000 3 Q9GLP0;K7GNY7;K7GQ83;F1RVE7;K7GS94

Q9GLP0;K7GNY7;K7GQ83;F1RVE7;K7GS94 836 sp|Q9GLP0|ITB1_PIG Integrin beta-1 OS=Sus scrofa OX=9823 GN=ITGB1 PE=2 SV=1;tr|K7GNY7|K7GNY7_PIG Integrin beta OS=Sus scrofa OX=9823 GN=ITGB1 PE=1 SV=3;tr|K7GQ83|K7GQ83_PIG Integrin beta OS=Sus scrofa OX=9823 GN=ITGB1 PE=1 SV=2;tr|F1RVE7|F1RVE7_PIG Integri

16654000 15937000 44963000 14459000 31282000 38360000 11283000 17365000 35711000 19769000

32775000 76026000 10 10 10 23,1 23,1 23,1 50,569 0 26,256

1623700000 67 F1RVN1 F1RVN1 837 tr|F1RVN1|F1RVN1_PIG NADH dehydrogenase [ubiquinone] flavoprotein 1, mitochondrial OS=Sus scrofa OX=9823 GN=NDUFV1 PE=1 SV=1

41781000 25780000 17045000 14508000 15996000 20753000 42844000 14827000 24030000 43086000

17158000 9065300 8 8 8 11 11 11 71,203 0 28,063

875700000 47 F1RWK0 F1RWK0 838 tr|F1RWK0|F1RWK0_PIG Junctophilin OS=Sus scrofa OX=9823 GN=JPH1 PE=1 SV=1

17706000 15235000 30498000 24985000 20273000 28938000 19379000 21904000 33516000 42908000
24335000 66226000 6 6 6 44,1 44,1 44,1 21,675 0 23,801
768610000 46 F1RWL7 F1RWL7 839 tr|F1RWL7|F1RWL7_PIG NADH dehydrogenase [ubiquinone] 1 beta subcomplex subunit 8, mitochondrial OS=Sus scrofa OX=9823 GN=NDUFB8 PE=1 SV=1
0 0 0 0 0 0 0 0 0 0 0 0 2 2 2 11,3 11,3 11,3
19,574 0 3,9801 10036000 3 F1RWM1;K7GNQ0;Q6DUB7;A0A5S6HQM6
F1RWM1;K7GNQ0;Q6DUB7;A0A5S6HQM6 840 tr|F1RWM1|F1RWM1_PIG Stathmin OS=Sus scrofa OX=9823 GN=STMN2 PE=1 SV=3;tr|K7GNQ0|K7GNQ0_PIG Stathmin OS=Sus scrofa OX=9823 GN=STMN2 PE=1 SV=3;sp|Q6DUB7|STMN1_PIG Stathmin OS=Sus scrofa OX=9823 GN=STMN1 PE=2 SV=3;tr|A0A5S6HQM6|A0A5S6HQM6_PIG Stathmin OS=Sus
0 0 4845400 3465900 4609600 5057700 0 0 0 0 3885400 10231000
4 4 4 9,6 9,6 9,6 45,369 0 7,126 88030000 15 F1RWZ4 F1RWZ4 841
tr|F1RWZ4|F1RWZ4_PIG Enoyl-CoA delta isomerase 2 OS=Sus scrofa OX=9823 GN=ECI2 PE=1 SV=3
0 0 0 0 0 0 0 0 0 0 0 0 1 1 1 4,3 4,3 4,3
27,172 0,0030488 1,7883 299270000 14 F1RX19;K7GQ32 F1RX19;K7GQ32 842
tr|F1RX19|F1RX19_PIG Chromosome X CXorf38 homolog OS=Sus scrofa OX=9823 GN=CXHXorf38 PE=4 SV=3;tr|K7GQ32|K7GQ32_PIG Chromosome X CXorf38 homolog OS=Sus scrofa OX=9823 GN=CXHXorf38 PE=4 SV=2
0 0 0 0 0 3319700 0 0 4256100 0 0 0 3 3 3 4
4 4 101,09 0 3,6574 27647000 4 F1RX36 F1RX36 843
tr|F1RX36|F1RX36_PIG Fibrinogen alpha chain OS=Sus scrofa OX=9823 GN=FGA PE=1 SV=4
0 0 6807300 0 3540400 0 0 0 0 0 0 0 4 4 4 8,1
8,1 8,1 59,001 0 7,004 52954000 8 I3LL55;F1RXA7 I3LL55;F1RXA7 844
tr|I3LL55|I3LL55_PIG Proteasome 26S subunit, non-ATPase 3 OS=Sus scrofa OX=9823 GN=PSMD3 PE=1 SV=2;tr|F1RXA7|F1RXA7_PIG 26S proteasome non-ATPase regulatory subunit 3 OS=Sus scrofa OX=9823 GN=PSMD3 PE=1 SV=3
0 0 0 0 0 0 0 0 0 0 0 0 1 1 1 1 1 1
88,369 0,0021622 2,1561 6651700000 44 F1RXD6 F1RXD6 845
tr|F1RXD6|F1RXD6_PIG Cyclic nucleotide gated channel subunit beta 3 OS=Sus scrofa OX=9823 GN=CNGB3 PE=4 SV=3
0 0 0 0 0 0 0 0 0 0 0 0 1 1 1 1,6 1,6 1,6
69,965 0,0031024 1,8713 4308500 1 F1RZV8 F1RZV8 846
tr|F1RZV8|F1RZV8_PIG Kelch like family member 31 OS=Sus scrofa OX=9823 GN=KLHL31 PE=1 SV=1
31604000 20867000 47409000 80662000 37022000 49373000 60056000 26392000 40275000 98732000
41577000 88413000 5 5 5 42,3 42,3 42,3 13,329 0 31,441
1314400000 56 F1S031 F1S031 847 tr|F1S031|F1S031_PIG NADH dehydrogenase [ubiquinone] iron-sulfur protein 6, mitochondrial OS=Sus scrofa OX=9823 GN=NDUFS6 PE=1 SV=1
0 0 0 0 0 0 0 0 0 0 0 0 2 1 1 3,5 2 2
50,357 0,003212 2,1052 9177300 1 F1S0K7 F1S0K7 848 tr|F1S0K7|F1S0K7_PIG Keratin 35 OS=Sus scrofa OX=9823 GN=KRT35 PE=1 SV=1

0 6849400 7596100 0 0 0 0 0 0 0 0 0 3 3 3 2,5
2,5 2,5 123,71 0,0021786 2,2646 34458000 3 F1S0N2 F1S0N2 849
tr|F1S0N2|F1S0N2_PIG ATP-citrate synthase OS=Sus scrofa OX=9823 GN=ACLY PE=1 SV=3
0 0 0 6999900 0 0 5341300 0 3198700 0 0 0 3 3 3
24 24 24 16,697 0 10,359 40329000 6 F1S192 F1S192 850
tr|F1S192|F1S192_PIG Prefoldin subunit 2 OS=Sus scrofa OX=9823 GN=PFDN2 PE=1 SV=1
0 0 0 0 0 0 0 0 0 0 0 0 1 1 1 11,3 11,3 11,3
11,041 0 3,09 41303000 8 F1S1L1 F1S1L1 851 tr|F1S1L1|F1S1L1_PIG Mitochondrial import inner membrane translocase subunit OS=Sus scrofa OX=9823 GN=TIMM8A PE=1 SV=1
0 0 0 0 0 0 0 0 0 0 0 0 2 2 2 5,9 5,9 5,9
58,336 0,0030706 1,8182 6933500 4 F1S288;A0A286ZNZ0 F1S288;A0A286ZNZ0
852 tr|F1S288|F1S288_PIG Syntrophin beta 1 OS=Sus scrofa OX=9823 GN=SNTB1 PE=1 SV=3;tr|A0A286ZNZ0|A0A286ZNZ0_PIG Syntrophin beta 1 OS=Sus scrofa OX=9823 GN=SNTB1 PE=1 SV=1
26137000 10576000 10591000 34825000 16517000 14659000 37193000 6893600 10442000 31376000
10185000 10285000 10 10 10 32,5 32,5 32,5 27,859 0 30,746
1032700000 53 F1S2A8 F1S2A8 853 tr|F1S2A8|F1S2A8_PIG B-cell receptor-associated protein OS=Sus scrofa OX=9823 GN=BCAP31 PE=1 SV=2
7101200 9361100 5384600 16450000 8402000 6724000 11203000 10434000 6749300 7152600
9903800 9179000 8 8 8 19,7 19,7 19,7 53,943 0 65,381
393650000 54 F1S2E2;A0A5K1UZZ4;F1RRP6;A0A287A080 F1S2E2;A0A5K1UZZ4 854
tr|F1S2E2|F1S2E2_PIG Annexin OS=Sus scrofa OX=9823 GN=ANXA11 PE=1 SV=2;tr|A0A5K1UZZ4|A0A5K1UZZ4_PIG Annexin OS=Sus scrofa OX=9823 GN=ANXA11 PE=1 SV=1
65629000 65139000 125550000 115330000 108790000 118920000 128450000 63045000
121710000 154030000 98228000 178490000 10 10 10 38,8 38,8 38,8
31,604 0 90,913 3652700000 106 F1S2F6;Q9MZ15;A0A287B452
F1S2F6;Q9MZ15;A0A287B452 855 tr|F1S2F6|F1S2F6_PIG Voltage-dependent anion-selective channel protein 2 OS=Sus scrofa OX=9823 GN=VDAC2 PE=3 SV=3;sp|Q9MZ15|VDAC2_PIG Voltage-dependent anion-selective channel protein 2 OS=Sus scrofa OX=9823 GN=VDAC2 PE=2 SV=1;tr|A0A287B452|A0A287B452_PIG
8717500 0 0 0 0 0 0 0 0 8583800 0 0 3 3 3 25,9
25,9 25,9 12,701 0 9,676 54088000 7 F1S2G3;A0A5G2QMR7 F1S2G3;A0A5G2QMR7
856 tr|F1S2G3|F1S2G3_PIG Tubulin-specific chaperone A OS=Sus scrofa OX=9823 GN=TBCA PE=1 SV=2;tr|A0A5G2QMR7|A0A5G2QMR7_PIG Tubulin-specific chaperone A OS=Sus scrofa OX=9823 GN=TBCA PE=1 SV=1
3857600 0 0 5761200 0 3827600 4460800 0 0 0 0 5600700 3
3 3 18,2 18,2 18,2 18,919 0 4,9212 53343000 5 F1S2I8 F1S2I8 857
tr|F1S2I8|F1S2I8_PIG Tubulin polymerization-promoting protein family member 3 OS=Sus scrofa OX=9823 GN=TPPP3 PE=1 SV=2
60905000 44916000 61263000 125670000 98704000 90188000 127540000 42555000 60653000
139760000 81696000 117170000 6 6 6 67 67 67 10,671 0

92,471 2346500000 78 F1S3W0 F1S3W0 858 tr|F1S3W0|F1S3W0_PIG Cytochrome b-c1 complex subunit 6 OS=Sus scrofa OX=9823 GN=LOC100524873 PE=1 SV=1

15762000 9278200 8907000 18409000 11597000 8514900 20919000 13269000 6055700 14089000

16220000 12183000 8 8 8 18,4 18,4 18,4 65,252 0 28,17

556570000 42 F1S415 F1S415 859 tr|F1S415|F1S415_PIG BAG cochaperone 3 OS=Sus scrofa OX=9823 GN=BAG3 PE=1 SV=2

4476900 4251900 2297000 3712100 2905400 2835300 3818800 0 4404300 4683900 3266200

3580900 4 4 4 14,1 14,1 14,1 38,273 0 9,1883 83960000 20

F1S4Y8 F1S4Y8 860 tr|F1S4Y8|F1S4Y8_PIG Eukaryotic translation initiation factor 2 subunit beta OS=Sus scrofa OX=9823 GN=EIF2S2 PE=1 SV=2

8102600 4347400 5624100 8502900 4570500 0 12340000 0 0 9550200 0 4802400

2 2 2 10,7 10,7 10,7 24,979 0 7,8799 124900000 11 F1S4Z2 F1S4Z2

861 tr|F1S4Z2|F1S4Z2_PIG Uncharacterized protein OS=Sus scrofa OX=9823 GN=CHMP4B PE=1 SV=3

77058000 277100000 79683000 48267000 214440000 107390000 28547000 242520000

149240000 23701000 209360000 64481000 58 58 58 43,9 43,9 43,9

170,98 0 323,31 25243000000 701 F1S557;A0A287AXA8;A0A287B3F4;A0A286ZMH7

F1S557;A0A287AXA8;A0A287B3F4;A0A286ZMH7 862 tr|F1S557|F1S557_PIG 4-alpha-glucanotransferase OS=Sus scrofa OX=9823 GN=AGL PE=1 SV=2;tr|A0A287AXA8|A0A287AXA8_PIG 4-alpha-glucanotransferase OS=Sus scrofa OX=9823 GN=AGL PE=1 SV=2;tr|A0A287B3F4|A0A287B3F4_PIG 4-alpha-glucanotransferase OS=Sus scrofa OX=9

0 0 0 0 0 0 0 0 0 0 0 0 1 1 1 3,1 3,1 3,1

43,712 0 6,342 83802000 9 F1S6B4 F1S6B4 863 tr|F1S6B4|F1S6B4_PIG Prolargin OS=Sus scrofa OX=9823 GN=PRELP PE=1 SV=3

0 0 0 0 0 0 0 0 0 0 0 0 3 3 3 5,3 5,3 5,3

47,708 0,0011261 2,523 76379000 4

F1S734;A0A5G2QB44;A0A287BB80;A0A5G2R7Z3;A0A287A541

F1S734;A0A5G2QB44;A0A287BB80 864 tr|F1S734|F1S734_PIG Protein-tyrosine-phosphatase OS=Sus scrofa OX=9823 GN=FBXO30 PE=4 SV=2;tr|A0A5G2QB44|A0A5G2QB44_PIG Protein-tyrosine-phosphatase OS=Sus scrofa OX=9823 GN=FBXO30 PE=4 SV=1;tr|A0A287BB80|A0A287BB80_PIG Uncharacterized protein OS=Sus scro

0 0 0 6605300 0 0 0 0 0 8126900 0 0 3 3 3 20,6

20,6 20,6 18,13 0 3,2061 79099000 8 F1S765 F1S765 865 tr|F1S765|F1S765_PIG CXXC motif containing zinc binding protein OS=Sus scrofa OX=9823 GN=CZIB PE=1 SV=2

821290000 1740100000 672890000 797430000 1472400000 875880000 615470000

1564500000 1114200000 417830000 1381300000 503470000 26 26

26 46,7 46,7 46,7 61,747 0 213,37 137700000000 1178 F1S814;A0A287A530

F1S814;A0A287A530 866 tr|F1S814|F1S814_PIG Phosphoglucomutase 1 OS=Sus scrofa OX=9823 GN=PGM1 PE=1 SV=4;tr|A0A287A530|A0A287A530_PIG Phosphoglucomutase 1 OS=Sus scrofa OX=9823 GN=PGM1 PE=1 SV=2

8512900 8347500 5764100 3845400 4617700 2182000 12792000 6215300 5037000 11193000

4823000 4779700 6 6 6 21,9 21,9 21,9 40,57 0 20,869

186670000 29 F1S880;A0A5G2R8G1;A0A287AGR0 F1S880;A0A5G2R8G1;A0A287AGR0

867 tr|F1S880|F1S880_PIG NSFL1 cofactor p47 OS=Sus scrofa OX=9823 GN=NSFL1C PE=1 SV=3;tr|A0A5G2R8G1|A0A5G2R8G1_PIG NSFL1 cofactor p47 OS=Sus scrofa OX=9823 GN=NSFL1C PE=1 SV=1;tr|A0A287AGR0|A0A287AGR0_PIG NSFL1 cofactor p47 OS=Sus scrofa OX=9823 GN=NSFL1C PE=1

0 0 12615000 10026000 10634000 11321000 19453000 12972000 9682000 18179000 11923000

11028000 3 3 3 13,9 13,9 13,9 25,627 0 16,995 261030000 28

F1S9H9;A0A286ZW71;P81693 F1S9H9;A0A286ZW71;P81693 868

tr|F1S9H9|F1S9H9_PIG Low molecular weight cytosolic acid phosphatase OS=Sus scrofa OX=9823 GN=ACP1 PE=1 SV=4;tr|A0A286ZW71|A0A286ZW71_PIG Low molecular weight phosphotyrosine protein phosphatase OS=Sus scrofa OX=9823 GN=ACP1 PE=1 SV=1;sp|P81693|PPAC_PIG Lo

11863000 4195500 5171000 0 0 0 4346800 0 0 0 4243700 0 3

3 3 11,6 11,6 11,6 37,865 0 15,831 84988000 13 F1S9T0 F1S9T0 869

tr|F1S9T0|F1S9T0_PIG DnaJ heat shock protein family (Hsp40) member B4 OS=Sus scrofa OX=9823 GN=DNAJB4 PE=1 SV=2

10529000 17682000 23252000 0 19402000 35175000 6240400 10724000 11913000 7100400 13477000

37013000 2 2 2 6 6 6 33,521 0 5,9036 343700000 34

F1S9V9 F1S9V9 870 tr|F1S9V9|F1S9V9_PIG Transmembrane protein 38A OS=Sus scrofa OX=9823 GN=TMEM38A PE=1 SV=3

0 0 0 0 0 0 0 0 0 0 0 0 1 1 1 0,9 0,9 0,9

111,44 0,0086622 1,4041 153730000 5 F1SAC4 F1SAC4 871

tr|F1SAC4|F1SAC4_PIG Uncharacterized protein OS=Sus scrofa OX=9823 GN=SCART1 PE=4 SV=3

0 5749200 0 0 0 0 9711600 0 0 7107700 0 0 4 4 4

6,5 6,5 6,5 72,908 0 17,062 89222000 21 F1SAD9 F1SAD9 872

tr|F1SAD9|F1SAD9_PIG Protein disulfide-isomerase OS=Sus scrofa OX=9823 GN=PDIA4 PE=1 SV=1

0 0 0 13308000 0 6361300 9663100 0 6695700 11399000 5821600 4794500

15 15 15 8,9 8,9 8,9 197,2 0 30,042 374230000 39

F1SAE9;A0A5G2QKZ6;A0A5G2R1S7 F1SAE9;A0A5G2QKZ6 873 tr|F1SAE9|F1SAE9_PIG Laminin subunit beta 1 OS=Sus scrofa OX=9823 GN=LAMB1 PE=1 SV=4;tr|A0A5G2QKZ6|A0A5G2QKZ6_PIG Laminin subunit beta 1 OS=Sus scrofa OX=9823 GN=LAMB1 PE=1 SV=1

0 58398000 129880000 116100000 115160000 103200000 94989000 329450000

215450000 103410000 71476000 117900000 4 4 4 42,7 42,7 42,7

11,317 0 32,12 2023900000 38 F1SAK7;A0A5G2QK91;A0A287A725

F1SAK7;A0A5G2QK91;A0A287A725 874 tr|F1SAK7|F1SAK7_PIG ATP synthase subunit OS=Sus scrofa OX=9823 GN=ATP5MG PE=1 SV=2;tr|A0A5G2QK91|A0A5G2QK91_PIG ATP synthase subunit OS=Sus scrofa OX=9823 GN=ATP5MG PE=1 SV=1;tr|A0A287A725|A0A287A725_PIG ATP synthase subunit OS=Sus scrofa OX=9823 GN=ATP5M

0 0 0 0 0 0 0 0 0 0 0 0 1 1 1 9,1 9,1 9,1

16,617 0,0022222 2,4018 6904500 2 F1SAR5 F1SAR5 875

tr|F1SAR5|F1SAR5_PIG Glutaredoxin 5 OS=Sus scrofa OX=9823 GN=GLRX5 PE=1 SV=1

7643400 11140000 5492500 0 6028000 3933300 0 0 0 0 0 3298400

4 4 4 10,9 10,9 10,9 48,633 0 21,355 104160000 10 F1SB53;A0A287BR45

F1SB53;A0A287BR45 876 tr|F1SB53|F1SB53_PIG 26S proteasome AAA-ATPase subunit RPT1 OS=Sus scrofa OX=9823 GN=PSMC2 PE=1 SV=2;tr|A0A287BR45|A0A287BR45_PIG 26S proteasome AAA-ATPase subunit RPT1 OS=Sus scrofa OX=9823 GN=PSMC2 PE=1 SV=1

6633600 5821600 6566800 4104500 6836800 6159300 0 0 6881100 0 0 4763900

4 4 4 7,7 7,7 7,7 60,652 0 7,1749 123630000 17

F1SB63;A0A287AKV4;A0A287AJY2;A0A287BDF2

F1SB63;A0A287AKV4;A0A287AJY2;A0A287BDF2 877 tr|F1SB63|F1SB63_PIG CCT-alpha OS=Sus scrofa OX=9823 GN=TCP1 PE=1 SV=2;tr|A0A287AKV4|A0A287AKV4_PIG T-complex 1 OS=Sus scrofa OX=9823 GN=TCP1 PE=1 SV=1;tr|A0A287AJY2|A0A287AJY2_PIG CCT-alpha OS=Sus scrofa OX=9823 GN=TCP1 PE=1 SV=2;tr|A0A287BDF2|A0A287BDF2_

66943000 38589000 109140000 49900000 35830000 157730000 62961000 31608000 61072000

57495000 41438000 117820000 11 11 4 26,6 26,6 11,3 46,391 0

89,125 3287400000 111 F1SCC9 F1SCC9 878 tr|F1SCC9|F1SCC9_PIG SERPIN domain-containing protein OS=Sus scrofa OX=9823 GN=LOC106504545 PE=1 SV=2

79469000 88023000 111210000 248740000 79109000 96308000 172640000 0 0 0

86601000 0 6 3 0 11 6 0 46,815 0 6,1636 1220600000

16 F1SCD1 F1SCD1 879 tr|F1SCD1|F1SCD1_PIG SERPIN domain-containing protein OS=Sus scrofa OX=9823 GN=LOC100156325 PE=1 SV=3

7981000 7413900 8538900 6989400 0 10812000 6577700 5325400 4360400 8576900 0

8445500 3 3 3 8,1 8,1 8,1 46,951 0 10,997 152150000 18

F1SCF0;P50447 F1SCF0;P50447 880 tr|F1SCF0|F1SCF0_PIG Alpha-1-antitrypsin OS=Sus scrofa OX=9823 GN=SERPINA1 PE=3 SV=2;sp|P50447|A1AT_PIG Alpha-1-antitrypsin OS=Sus scrofa OX=9823 GN=SERPINA1 PE=2 SV=1

14486000 0 15252000 17192000 16126000 16519000 16728000 0 13004000 26719000 0 0

2 2 2 12,4 12,4 12,4 16,458 0 3,2713 282680000 12 F1SCH1 F1SCH1

881 tr|F1SCH1|F1SCH1_PIG Complex I-B18 OS=Sus scrofa OX=9823 GN=NDUFB7 PE=1 SV=3

0 0 41306000 29038000 34171000 33255000 32022000 0 0 45617000 0 61278000

2 2 2 32,8 32,8 32,8 7,0322 0 8,1512 473770000 17 F1SD73 F1SD73 882

tr|F1SD73|F1SD73_PIG Complex I-MNLL OS=Sus scrofa OX=9823 PE=1 SV=2

22782000 14340000 14419000 9746300 11028000 11198000 20449000 13523000 12849000 19590000

11929000 9467500 5 5 5 15,5 15,5 15,5 44,032 0 28,64

604950000 41 F1SDT0 F1SDT0 883 tr|F1SDT0|F1SDT0_PIG Uncharacterized protein OS=Sus scrofa OX=9823 GN=JPH2 PE=1 SV=2

0 0 0 0 0 0 0 0 0 0 0 0 1 1 1 2,2 2,2 2,2

68,916 0,0011547 2,7717 19313000 1 I3LLF2;Q9GL01;F1SEM2;I3LRG0

I3LLF2;Q9GL01;F1SEM2;I3LRG0 884 tr|I3LLF2|I3LLF2_PIG Dolichyl-diphosphooligosaccharide--protein glycosyltransferase subunit 2 OS=Sus scrofa OX=9823 GN=RPN2

PE=1 SV=3;sp|Q9GL01|RPN2_PIG Dolichyl-diphosphooligosaccharide--protein glycosyltransferase subunit 2 OS=Sus scrofa OX=9823 GN=RPN2
6391900 0 0 6222300 0 0 6029600 0 0 8180200 0 5222000 5
5 5 10,2 10,2 10,2 61,307 0 11,789 83120000 12
P42174;F1SEN2;A0A287ATW3;A0A5G2R3R8 P42174;F1SEN2;A0A287ATW3;A0A5G2R3R8
885 sp|P42174|DHE3_PIG Glutamate dehydrogenase 1, mitochondrial OS=Sus scrofa OX=9823 GN=GLUD1 PE=1 SV=2;tr|F1SEN2|F1SEN2_PIG Glutamate dehydrogenase (NAD(P)(+)) OS=Sus scrofa OX=9823 GN=GLUD1 PE=1 SV=1;tr|A0A287ATW3|A0A287ATW3_PIG Glutamate dehydrogenase (NAD
0 0 8873700 0 8999500 9888100 9633300 0 8667400 12053000 8149800 11856000
2 2 2 6,1 6,1 6,1 37,077 0 5,8793 171990000 4 F1SEQ3 F1SEQ3
886 tr|F1SEQ3|F1SEQ3_PIG Growth hormone-inducible transmembrane protein OS=Sus scrofa OX=9823 GN=GHITM PE=1 SV=1
41788000 58004000 64951000 39990000 45028000 84832000 48822000 42062000 51326000 45181000
47075000 64556000 9 9 9 29,9 29,9 29,9 38,789 0 48,066
1924900000 102 F1SFI7;P29700;A0A287BDE8 F1SFI7;P29700;A0A287BDE8 887
tr|F1SFI7|F1SFI7_PIG Alpha-2-HS-glycoprotein OS=Sus scrofa OX=9823 GN=AHSG PE=1 SV=3;sp|P29700|FETUA_PIG Alpha-2-HS-glycoprotein (Fragment) OS=Sus scrofa OX=9823 GN=AHSG PE=1 SV=1;tr|A0A287BDE8|A0A287BDE8_PIG Alpha-2-HS-glycoprotein OS=Sus scrofa OX=9823 G
0 0 0 0 0 0 0 0 0 0 0 0 1 1 1 3,7 3,7 3,7
46,601 0,0078049 1,4838 2152900 1 F1SGP8 F1SGP8 888
tr|F1SGP8|F1SGP8_PIG Reticulocalbin-1 OS=Sus scrofa OX=9823 GN=RCN1 PE=1 SV=3
8586700 10497000 38813000 11433000 26872000 36611000 9742700 15392000 32624000 17785000
32108000 69916000 11 11 11 26,6 26,6 26,6 40,421 0 50,15
1521300000 65 F1SIS9;A0A5G2R3C0 F1SIS9 889 tr|F1SIS9|F1SIS9_PIG NADH dehydrogenase [ubiquinone] 1 alpha subcomplex subunit 10, mitochondrial OS=Sus scrofa OX=9823 GN=NDUFA10 PE=1 SV=1
6219300 13479000 17705000 45211000 11640000 13853000 24531000 31752000 46326000 51738000
21129000 31903000 2 2 2 17,1 17,1 17,1 16,748 0 22,342
504290000 52 F1SJ34;A0A287B0Q9 F1SJ34;A0A287B0Q9 890 tr|F1SJ34|F1SJ34_PIG Cytochrome c oxidase polypeptide Va OS=Sus scrofa OX=9823 GN=COX5A PE=1 SV=1;tr|A0A287B0Q9|A0A287B0Q9_PIG Cytochrome c oxidase polypeptide Va OS=Sus scrofa OX=9823 GN=COX5A PE=1 SV=1
4571800 0 0 0 0 0 4392000 0 0 5716200 0 4577300 3 3
2 11,1 11,1 7,3 36,669 0 3,7755 73080000 10 F1SJ93 F1SJ93 891
tr|F1SJ93|F1SJ93_PIG Reticulocalbin 2 OS=Sus scrofa OX=9823 GN=RCN2 PE=1 SV=4
12716000 11752000 5735400 7718400 0 5922900 9694800 0 6259400 9548400 5501500 0
6 6 6 25,3 25,3 25,3 33,264 0 28,914 221300000 25
K7GLE1;F1SJB5;P19619 K7GLE1;F1SJB5;P19619 892 tr|K7GLE1|K7GLE1_PIG Annexin OS=Sus scrofa OX=9823 GN=ANXA1 PE=1 SV=2;tr|F1SJB5|F1SJB5_PIG Annexin OS=Sus scrofa OX=9823 GN=ANXA1 PE=1 SV=3;sp|P19619|ANXA1_PIG Annexin A1 OS=Sus scrofa OX=9823 GN=ANXA1 PE=1 SV=3

13743000 9196300 7798800 0 0 0 0 0 0 0 0 0 12 12 12
14 14 14 122,83 0 36,023 192240000 24 F1SJC2 F1SJC2 893
tr|F1SJC2|F1SJC2_PIG Protein phosphatase 1 regulatory subunit 3A OS=Sus scrofa OX=9823 GN=PPP1R3A PE=1 SV=3
0 0 7649500 0 0 7373900 0 0 0 0 0 10617000 7 7 7
12,9 12,9 12,9 62,132 0 15,446 189630000 13 F1SJE6;A0A5G2QE37;A0A5G2QVN5
F1SJE6;A0A5G2QE37;A0A5G2QVN5 894 tr|F1SJE6|F1SJE6_PIG Phosphoglucomutase 5 OS=Sus scrofa OX=9823 GN=PGM5 PE=1 SV=3;tr|A0A5G2QE37|A0A5G2QE37_PIG Phosphoglucomutase 5 OS=Sus scrofa OX=9823 GN=PGM5 PE=1 SV=1;tr|A0A5G2QVN5|A0A5G2QVN5_PIG Phosphoglucomutase 5 OS=Sus scrofa OX=9823 GN=PGM5 PE=1
0 0 0 0 0 0 0 0 0 0 0 0 1 1 1 5,2 5,2 5,2
17,854 0,0049358 1,5325 313730000 20 F1SJP6 F1SJP6 895
tr|F1SJP6|F1SJP6_PIG Complex I-B14 OS=Sus scrofa OX=9823 GN=NDUFA6 PE=1 SV=3
0 0 0 13073000 0 0 8322000 0 0 12871000 0 0 7 7 7
35,7 35,7 35,7 31,191 0 16,879 119690000 17 F1SKY2;A0A5G2RGN4
F1SKY2;A0A5G2RGN4 896 tr|F1SKY2|F1SKY2_PIG Omega-amidase NIT2 OS=Sus scrofa OX=9823 GN=NIT2 PE=1 SV=3;tr|A0A5G2RGN4|A0A5G2RGN4_PIG CN hydrolase domain-containing protein OS=Sus scrofa OX=9823 GN=NIT2 PE=1 SV=1
172140000 184360000 371550000 229490000 300860000 313450000 169880000
274500000 394560000 351770000 367620000 576640000 15 15
15 42,4 42,4 42,4 48,643 0 139,65 22362000000 482
F1SLA0;K7GLT8;A0A287BET3;A0A287ANH8 F1SLA0;K7GLT8;A0A287BET3 897
tr|F1SLA0|F1SLA0_PIG ATP synthase subunit beta OS=Sus scrofa OX=9823 GN=ATP5F1B PE=1 SV=3;tr|K7GLT8|K7GLT8_PIG ATP synthase subunit beta OS=Sus scrofa OX=9823 GN=ATP5F1B PE=1 SV=2;tr|A0A287BET3|A0A287BET3_PIG ATP synthase F1 subunit beta OS=Sus scrofa OX=9
27291000 34932000 25549000 29608000 33709000 41784000 20187000 33503000 26871000 25154000
29107000 36373000 10 10 1 43,8 43,8 2,6 28,563 0 25,234
1617100000 109 F1SLF0 F1SLF0 898 tr|F1SLF0|F1SLF0_PIG Sepiapterin reductase OS=Sus scrofa OX=9823 GN=SPR PE=1 SV=3
23359000 14780000 38666000 23113000 26998000 29796000 18355000 19419000 29822000 24331000
29594000 69777000 5 5 5 25,7 25,7 25,7 27,715 0 11,79
1008700000 32 F1SLR1;A0A5G2QVE8 F1SLR1;A0A5G2QVE8 899
tr|F1SLR1|F1SLR1_PIG Complex I-19kD OS=Sus scrofa OX=9823 GN=NDUFA8 PE=1 SV=3;tr|A0A5G2QVE8|A0A5G2QVE8_PIG Complex I-19kD OS=Sus scrofa OX=9823 GN=NDUFA8 PE=1 SV=1
0 21577000 20226000 71756000 43431000 30511000 51870000 43305000 31953000 44783000 37623000
42162000 7 7 7 66,7 66,7 66,7 10,96 0 11,407 1029900000 38
F1SMZ6;A0A287A1U5;A0A5G2R8I0;A0A5G2QEY3
F1SMZ6;A0A287A1U5;A0A5G2R8I0;A0A5G2QEY3 900 tr|F1SMZ6|F1SMZ6_PIG 10 kDa heat shock protein, mitochondrial OS=Sus scrofa OX=9823 GN=HSPE1 PE=1 SV=1;tr|A0A287A1U5|A0A287A1U5_PIG 10 kDa heat shock protein, mitochondrial OS=Sus scrofa OX=9823 GN=HSPE1 PE=1 SV=1;tr|A0A5G2R8I0|A0A5G2R8I0_PIG 10 kDa heat s

19166000 6503600 73612000 15674000 4231600 88125000 20860000 3787800 37728000 19601000
5348500 72124000 8 8 8 30 30 30 23,045 0 36,033
2503900000 85 F1SN68;A0A5G2R0I4 F1SN68;A0A5G2R0I4 901
tr|F1SN68|F1SN68_PIG Alpha-1-acid glycoprotein OS=Sus scrofa OX=9823 GN=ORM1 PE=1 SV=1;tr|A0A5G2R0I4|A0A5G2R0I4_PIG Lipocln_cytosolic_FA-bd_dom domain-containing protein OS=Sus scrofa OX=9823 GN=ORM1 PE=1 SV=1
98522000 25101000 204700000 231260000 121710000 317600000 126220000 95119000
97357000 73487000 80172000 140800000 9 7 6 45,9 37,8 32,1 21,76
0 86,604 4817200000 133 F1SNW4;A0A5G2QI13 F1SNW4;A0A5G2QI13 902
tr|F1SNW4|F1SNW4_PIG Myosin light chain 3 OS=Sus scrofa OX=9823 GN=MYL3 PE=1 SV=3;tr|A0A5G2QI13|A0A5G2QI13_PIG Myosin light chain 3 OS=Sus scrofa OX=9823 GN=MYL3 PE=1 SV=1
21147000 11168000 11344000 16777000 14870000 10686000 26811000 9882500 9944800 18636000
8795400 9907100 5 5 5 19,9 19,9 19,9 34,975 0 31,343
475500000 20 F1SP32 F1SP32 903 tr|F1SP32|F1SP32_PIG UV excision repair protein RAD23 OS=Sus scrofa OX=9823 GN=RAD23B PE=1 SV=2
5765100 7822000 6785400 7524700 6899500 7078500 10127000 7534200 7119000 6921900
6766300 8157700 6 6 6 14,1 14,1 14,1 58,056 0 19,125
265340000 10 F1SQN1;A0A5G2QN54 F1SQN1;A0A5G2QN54 906
tr|F1SQN1|F1SQN1_PIG T-complex protein 1 subunit delta OS=Sus scrofa OX=9823 GN=CCT4 PE=1 SV=1;tr|A0A5G2QN54|A0A5G2QN54_PIG T-complex protein 1 subunit delta OS=Sus scrofa OX=9823 GN=CCT4 PE=1 SV=1
11580000 12762000 33621000 17667000 17688000 29421000 14160000 11028000 23840000 22071000
26619000 69187000 5 5 5 40 40 40 17,136 0 12,194
657880000 58 F1SQP4;A0A5G2QQP8;A0A286ZTC8;A0A5G2QB28;A0A5G2QM44
F1SQP4;A0A5G2QQP8;A0A286ZTC8;A0A5G2QB28 907 tr|F1SQP4|F1SQP4_PIG NADH dehydrogenase [ubiquinone] 1 alpha subcomplex subunit 12 OS=Sus scrofa OX=9823 GN=NDUFA12 PE=1 SV=2;tr|A0A5G2QQP8|A0A5G2QQP8_PIG Uncharacterized protein OS=Sus scrofa OX=9823 GN=NDUFA12 PE=1 SV=1;tr|A0A286ZTC8|A0A286ZTC8_PIG NADH
6745500 13009000 8872500 7362400 7872100 12525000 7582900 9169800 8449700 5940600
9300300 7898000 8 8 8 14 14 14 68,623 0 9,6888
275580000 27 F1SQR8 F1SQR8 908 tr|F1SQR8|F1SQR8_PIG Leukotriene A(4) hydrolase OS=Sus scrofa OX=9823 GN=LTA4H PE=1 SV=4
34331000 55447000 180330000 74452000 200110000 120090000 54602000 93977000 94246000
44810000 164610000 281810000 9 8 8 31,7 28,1 28,1 36,612 0
102,07 6062200000 151 P00336;F1SR05;A0A287AQS1;A0A5G2QHL7
P00336;F1SR05;A0A287AQS1;A0A5G2QHL7 909 sp|P00336|LDHB_PIG L-lactate dehydrogenase B chain OS=Sus scrofa OX=9823 GN=LDHB PE=1 SV=3;tr|F1SR05|F1SR05_PIG L-lactate dehydrogenase OS=Sus scrofa OX=9823 GN=LDHB PE=3 SV=3;tr|A0A287AQS1|A0A287AQS1_PIG L-lactate dehydrogenase B chain OS=Sus scrofa OX=98
0 0 0 0 0 0 0 0 0 0 0 0 3 1 0 21 5,1 0
17,015 0,005 1,6457 259630000 9 F1SR48 F1SR48 910 tr|F1SR48|F1SR48_PIG

Protein phosphatase 1 regulatory inhibitor subunit 1A OS=Sus scrofa OX=9823 GN=PPP1R1A PE=1 SV=4
0 0 0 0 0 0 0 0 0 0 0 0 27 1 1 40,1 1 1
85,447 0 3,3856 398020000 6 F1SRC5 F1SRC5 911 tr|F1SRC5|F1SRC5_PIG Aconitate hydratase, mitochondrial OS=Sus scrofa OX=9823 GN=ACO2 PE=1 SV=3
7226400 9862300 11567000 0 6554900 0 0 0 0 0 0 0 4 4
4 6,9 6,9 6,9 69,594 0 7,2193 80218000 8 F1SRF1 F1SRF1 913
tr|F1SRF1|F1SRF1_PIG Kelch like family member 40 OS=Sus scrofa OX=9823 GN=KLHL40 PE=3 SV=3
13527000 39196000 59876000 0 33830000 22980000 0 6072900 35458000 4294300 34114000
73175000 79 16 3 40,7 7,9 2,1 223,17 0 56,235 1799700000 84
Q9TV61;F1SS65 Q9TV61;F1SS65 914 sp|Q9TV61|MYH1_PIG Myosin-1 OS=Sus scrofa OX=9823 GN=MYH1 PE=2 SV=1;tr|F1SS65|F1SS65_PIG Myosin-4 OS=Sus scrofa OX=9823 GN=MYH4 PE=3 SV=4
0 0 0 0 0 0 0 0 0 0 0 0 2 2 2 6,8 6,8 6,8
38,094 0,0078355 1,497 50719000 3 F1SSI2;A0A5G2QGL0 F1SSI2;A0A5G2QGL0
916 tr|F1SSI2|F1SSI2_PIG Dehydrogenase/reductase 7 OS=Sus scrofa OX=9823 GN=DHRS7 PE=1 SV=1;tr|A0A5G2QGL0|A0A5G2QGL0_PIG Dehydrogenase/reductase 7 OS=Sus scrofa OX=9823 GN=DHRS7 PE=1 SV=1
0 0 0 8507000 0 0 10613000 0 0 0 0 0 3 3 3 27,3
27,3 27,3 14,51 0 7,9832 60156000 6 F1SSK4 F1SSK4 917 tr|F1SSK4|F1SSK4_PIG zf-Tim10_DDP domain-containing protein OS=Sus scrofa OX=9823 PE=1 SV=2
26225000 15897000 20088000 42099000 16776000 26894000 49031000 17537000 17497000 40145000
12545000 14529000 6 6 6 17,7 17,7 17,7 35,824 0 11,473
654350000 29 F1ST73;A0A287A1W5 F1ST73;A0A287A1W5 918 tr|F1ST73|F1ST73_PIG Glyoxylate and hydroxypyruvate reductase OS=Sus scrofa OX=9823 GN=GRHPR PE=1 SV=3;tr|A0A287A1W5|A0A287A1W5_PIG Glyoxylate and hydroxypyruvate reductase OS=Sus scrofa OX=9823 GN=GRHPR PE=1 SV=1
21579000 24943000 16936000 37946000 19034000 21411000 43475000 10742000 10763000 33239000
20903000 15866000 10 10 10 24,3 24,3 24,3 52,285 0 48,284
1117900000 69 I3LEY2;F1SU59;A0A5G2R850 I3LEY2;F1SU59;A0A5G2R850 919
tr|I3LEY2|I3LEY2_PIG Annexin OS=Sus scrofa OX=9823 GN=ANXA7 PE=1 SV=3;tr|F1SU59|F1SU59_PIG Annexin OS=Sus scrofa OX=9823 GN=ANXA7 PE=1 SV=4;tr|A0A5G2R850|A0A5G2R850_PIG Annexin OS=Sus scrofa OX=9823 GN=ANXA7 PE=1 SV=1
0 0 0 0 0 0 0 0 0 0 0 0 5 5 5 11,1 11,1 11,1
42,446 0 8,5866 47115000 6 F1SUE4;A0A5G2R455 F1SUE4 920
tr|F1SUE4|F1SUE4_PIG Asporin OS=Sus scrofa OX=9823 GN=ASPN PE=1 SV=1
25629000 12473000 20783000 0 9713600 0 7373300 0 9434300 5284100 0 5821900
5 5 5 42,4 42,4 42,4 13,17 0 12,745 368200000 13
F1SUG4;A0A5G2R2Y3;A0A5G2R1M4 F1SUG4;A0A5G2R2Y3 921 tr|F1SUG4|F1SUG4_PIG Ubiquitin-like domain-containing protein OS=Sus scrofa OX=9823 GN=ELOB PE=1 SV=1;tr|A0A5G2R2Y3|A0A5G2R2Y3_PIG Ubiquitin-like domain-containing protein OS=Sus scrofa OX=9823 GN=ELOB PE=1 SV=1

18870000 13086000 24586000 34903000 22878000 25613000 23633000 20171000 38470000 46202000
23066000 62326000 4 4 4 39,6 39,6 39,6 12,48 0 9,5814
756340000 11 F1SV23 F1SV23 922 tr|F1SV23|F1SV23_PIG Complex I-15 kDa OS=Sus scrofa OX=9823 GN=NDUFS5 PE=1 SV=1
38165000 17192000 21591000 0 0 0 0 0 0 0 0 7089200 4 4
4 15 15 15 31,114 0 14,807 213520000 9 F1SV39;F1SV27
F1SV39;F1SV27 923 tr|F1SV39|F1SV39_PIG Four and a half LIM domains 3 OS=Sus scrofa OX=9823 GN=FHL3 PE=1 SV=1;tr|F1SV27|F1SV27_PIG Four and a half LIM domains 3 OS=Sus scrofa OX=9823 GN=FHL3 PE=1 SV=2
9222500 0 11140000 0 7203700 0 6972000 0 5388000 7782100 0 11702000
10 5 5 39,4 22 22 28,211 0 31,444 147300000 19 F2Z4Y1 F2Z4Y1 924
tr|F2Z4Y1|F2Z4Y1_PIG Tyrosine 3-monooxygenase/tryptophan 5-monooxygenase activation protein eta OS=Sus scrofa OX=9823 GN=YWHAH PE=1 SV=1
227270000 202550000 143330000 28070000 111120000 30627000 55186000 33068000
33731000 31655000 29364000 54191000 13 13 7 54,3 54,3 33,2 28,302 0
90,118 5486000000 181 F2Z4Z1 F2Z4Z1 925 tr|F2Z4Z1|F2Z4Z1_PIG 14_3_3 domain-containing protein OS=Sus scrofa OX=9823 GN=YWHAG PE=1 SV=1
6440300 0 0 7986600 0 0 8564600 0 0 9978100 0 0 2 2
2 6,1 6,1 6,1 42,512 0 5,6243 61551000 7 P80229;F2Z5B1 P80229;F2Z5B1
926 sp|P80229|ILEU_PIG Leukocyte elastase inhibitor OS=Sus scrofa OX=9823 GN=SERPINB1 PE=1 SV=1;tr|F2Z5B1|F2Z5B1_PIG Leukocyte elastase inhibitor OS=Sus scrofa OX=9823 GN=SERPINB1 PE=1 SV=2
43697000 27496000 15166000 37771000 15511000 14197000 42868000 13016000 18940000 40488000
19180000 16378000 8 8 7 22,7 22,7 20,6 36,134 0 64,015
1285800000 70 F2Z5C1;A0A287A531;A0A5G2R3B5 F2Z5C1;A0A287A531;A0A5G2R3B5
927 tr|F2Z5C1|F2Z5C1_PIG Annexin OS=Sus scrofa OX=9823 GN=ANXA5 PE=1 SV=4;tr|A0A287A531|A0A287A531_PIG Annexin OS=Sus scrofa OX=9823 GN=ANXA5 PE=1 SV=2;tr|A0A5G2R3B5|A0A5G2R3B5_PIG Annexin OS=Sus scrofa OX=9823 GN=ANXA5 PE=1 SV=1
0 0 0 0 0 0 0 0 0 0 0 0 1 1 1 5,3 5,3 5,3
24,205 0,0032154 2,1076 25636000 2 F2Z5F5 F2Z5F5 928 tr|F2Z5F5|F2Z5F5_PIG 40S ribosomal protein S8 OS=Sus scrofa OX=9823 GN=RPS8 PE=1 SV=3
11821000 0 8812000 13393000 8455800 13345000 10663000 0 8581600 14353000 0 7249400
2 2 2 15,2 15,2 15,2 13,742 0 3,6641 185140000 17 F2Z5G8
F2Z5G8 929 tr|F2Z5G8|F2Z5G8_PIG 40S ribosomal protein S25 OS=Sus scrofa OX=9823 GN=RPS25 PE=1 SV=3
6132400 0 9741100 0 0 0 0 0 0 0 0 0 3 3 3 9,5
9,5 9,5 49,184 0,0011325 2,5943 45968000 7 F2Z5J1 F2Z5J1 930
tr|F2Z5J1|F2Z5J1_PIG 26S protease regulatory subunit 4 OS=Sus scrofa OX=9823 GN=PSMC1 PE=1 SV=1
17560000 16343000 13787000 27296000 16697000 16867000 24631000 16177000 13552000 17156000
17526000 16910000 5 5 5 25,4 25,4 25,4 25,242 0 24,147
676770000 45 F2Z5K2 F2Z5K2 931 tr|F2Z5K2|F2Z5K2_PIG Proteasome subunit alpha type-5 OS=Sus scrofa OX=9823 GN=PSMA5 PE=1 SV=2

12589000 11189000 14471000 27349000 14194000 18644000 20590000 9152600 15356000 19865000
13260000 13828000 3 3 3 19,6 19,6 19,6 20,987 0 6,2659
375130000 23
F2Z5K3;A0A5G2QN26;A0A5G2QA99;A0A287APX6;A0A287B931;A0A5G2RFP5;A0A286ZVJ2;A0A5G2QZL2
F2Z5K3;A0A5G2QN26;A0A5G2QA99;A0A287APX6;A0A287B931;A0A5G2RFP5;A0A286ZVJ2
932 tr|F2Z5K3|F2Z5K3_PIG RAP1A, member of RAS oncogene family OS=Sus scrofa OX=9823 GN=RAP1A PE=1 SV=3;tr|A0A5G2QN26|A0A5G2QN26_PIG Uncharacterized protein OS=Sus scrofa OX=9823 GN=RAP1B PE=1 SV=1;tr|A0A5G2QA99|A0A5G2QA99_PIG Uncharacterized protein OS=Sus scr
0 0 0 0 0 0 0 0 0 0 0 0 1 1 1 17,7 17,7 17,7
11,075 0,0030457 1,7823 33094000 3 P04163;F2Z5M2 P04163;F2Z5M2 933
sp|P04163|S10AA_PIG Protein S100-A10 OS=Sus scrofa OX=9823 GN=S100A10 PE=1 SV=2;tr|F2Z5M2|F2Z5M2_PIG Calpactin I light chain OS=Sus scrofa OX=9823 GN=S100A10 PE=2 SV=1
5418300 5325000 6453000 0 4618300 9900800 5107500 0 6293000 0 4308000 6070700
4 4 4 16,9 16,9 16,9 28,68 0 7,037 123300000 12
F2Z5Q6;A0A287BPA1;F1SML8;A0A287BCW5 F2Z5Q6 934 tr|F2Z5Q6|F2Z5Q6_PIG 40S ribosomal protein S6 OS=Sus scrofa OX=9823 GN=RPS6 PE=1 SV=4
0 0 0 0 0 0 0 0 0 0 0 0 3 3 3 6,3 6,3 6,3
55,238 0 3,9309 12694000 6 Q19PY3;F2Z5V0 Q19PY3;F2Z5V0 935
sp|Q19PY3|RTCB_PIG RNA-splicing ligase RtcB homolog OS=Sus scrofa OX=9823 GN=RTCB PE=2 SV=1;tr|F2Z5V0|F2Z5V0_PIG RNA-splicing ligase RtcB homolog OS=Sus scrofa OX=9823 GN=RTCB PE=3 SV=1
0 0 0 0 0 0 0 0 0 0 0 0 1 1 1 9,9 9,9 9,9
12,698 0,0011403 2,6693 16693000 2 F2Z5V7 F2Z5V7 936
tr|F2Z5V7|F2Z5V7_PIG Mitochondrial import inner membrane translocase subunit OS=Sus scrofa OX=9823 GN=TIMM8B PE=1 SV=2
5350400 9672300 8633400 5864800 8578100 8238400 5617200 5742000 6029500 6569400
4726700 9097600 4 4 4 15,2 15,2 15,2 31,62 0 8,1715
249590000 25 F6Q2A5;Q0MVN8;Q19QT8;A0A287BRT8 F6Q2A5;Q0MVN8;Q19QT8 937
tr|F6Q2A5|F6Q2A5_PIG Quinone oxidoreductase OS=Sus scrofa OX=9823 GN=CRYZ PE=3 SV=1;sp|Q0MVN8|QOR_PIG Quinone oxidoreductase OS=Sus scrofa OX=9823 GN=CRYZ PE=2 SV=1;tr|Q19QT8|Q19QT8_PIG Quinone oxidoreductase OS=Sus scrofa OX=9823 GN=CRYZ PE=2 SV=1
0 0 11452000 0 0 21258000 0 0 7898600 0 0 11062000 5 2
2 9,4 4,4 4,4 46,301 0 3,6256 98828000 6 F6Q469 F6Q469 938
tr|F6Q469|F6Q469_PIG SERPIN domain-containing protein OS=Sus scrofa OX=9823 GN=LOC100156325 PE=1 SV=1
8203000 0 0 7208300 0 8856400 0 0 7076100 0 7684700 7984500
3 3 3 12,2 12,2 12,2 24,217 0 5,397 114320000 11
F6Q5P0;P62279;A0A287ARH8 F6Q5P0;P62279 939 tr|F6Q5P0|F6Q5P0_PIG 40S

ribosomal protein S13 OS=Sus scrofa OX=9823 GN=RPS13 PE=1 SV=4;sp|P62279|RS13_PIG 40S ribosomal protein S13 (Fragment) OS=Sus scrofa OX=9823 GN=RPS13 PE=2 SV=2
36431000 10610000 70335000 78312000 79048000 152490000 19134000 44310000 44434000 14843000 86498000 325760000 6 6 6 24 24 24 21,565 0 47,747 1777300000 37 F6Q6A7 F6Q6A7 940 tr|F6Q6A7|F6Q6A7_PIG Cysteine and glycine rich protein 3 OS=Sus scrofa OX=9823 GN=CSRP3 PE=1 SV=2
7292000 7681300 0 7054500 7142500 8008500 8968200 0 0 6103800 0 6934800 7 7 7 14,4 14,4 14,4 57,33 0 9,5927 237640000 19 F6Q936;A0A5G2QMG1 F6Q936;A0A5G2QMG1 941 tr|F6Q936|F6Q936_PIG CCT-beta OS=Sus scrofa OX=9823 GN=CCT2 PE=1 SV=1;tr|A0A5G2QMG1|A0A5G2QMG1_PIG CCT-beta OS=Sus scrofa OX=9823 GN=CCT2 PE=1 SV=1
15210000 13802000 14782000 5855400 9449600 11356000 10731000 7961500 13640000 8333700 6286400 9331100 9 9 9 20,6 20,6 20,6 56,729 0 16,864 445200000 43 F6QA08 F6QA08 942 tr|F6QA08|F6QA08_PIG Protein disulfide-isomerase OS=Sus scrofa OX=9823 GN=PDIA3 PE=1 SV=1
21963000 0 0 0 15652000 26749000 26278000 0 16234000 20986000 13009000 43694000 2 2 2 12,7 12,7 12,7 20,029 0 11,62 381740000 19 I3L660;A0A5G2QQU9 I3L660;A0A5G2QQU9 943 tr|I3L660|I3L660_PIG Profilin OS=Sus scrofa OX=9823 GN=PFN2 PE=1 SV=3;tr|A0A5G2QQU9|A0A5G2QQU9_PIG Profilin OS=Sus scrofa OX=9823 GN=PFN2 PE=1 SV=1
0 0 2649100 0 0 3213200 0 0 0 0 0 0 4 4 4 6,6 6,6 6,6 59,055 0 3,7779 33425000 7 I3L804 I3L804 944 tr|I3L804|I3L804_PIG Tyrosine--tRNA ligase OS=Sus scrofa OX=9823 GN=YARS1 PE=1 SV=2
0 0 0 0 0 0 0 0 0 0 0 0 4 4 4 4,3 4,3 4,3 110,65 0,0011468 2,6845 76167000 4 I3LPP4;I3L918;A0A287BD56 I3LPP4;I3L918;A0A287BD56 945 tr|I3LPP4|I3LPP4_PIG Chromosome segregation 1-like protein OS=Sus scrofa OX=9823 GN=CSE1L PE=1 SV=2;tr|I3L918|I3L918_PIG Chromosome segregation 1-like protein OS=Sus scrofa OX=9823 GN=CSE1L PE=1 SV=3;tr|A0A287BD56|A0A287BD56_PIG Chromosome segregation 1 li
0 0 0 0 0 0 0 0 0 0 0 0 2 2 2 11 11 11 30,722 0 8,4872 58395000 2 I3L9N1 I3L9N1 946 tr|I3L9N1|I3L9N1_PIG Uncharacterized protein OS=Sus scrofa OX=9823 GN=ADPRHL1 PE=1 SV=2
0 0 0 0 0 0 0 0 0 0 0 0 1 1 1 5,7 5,7 5,7 19,676 0 26,548 2267500000 38 I3L9U0 I3L9U0 947 tr|I3L9U0|I3L9U0_PIG Myosin light chain 5 OS=Sus scrofa OX=9823 GN=MYL5 PE=4 SV=3
0 0 0 0 0 0 0 0 0 0 0 0 1 1 1 7,3 7,3 7,3 23,521 0,0031414 1,9601 56473000 10 I3LAJ6 I3LAJ6 948 tr|I3LAJ6|I3LAJ6_PIG Small monomeric GTPase OS=Sus scrofa OX=9823 GN=RALA PE=1 SV=1
63653000 43236000 35948000 98056000 65924000 66454000 109470000 45878000 32968000 83391000 48375000 49449000 7 7 7 60,6 60,6 60,6 11,206 0 65,142 2792500000 101 P00819;I3LC10;A0A5G2RBE9;A0A5G2R9Q8;A0A5G2RKL2 P00819;I3LC10;A0A5G2RBE9;A0A5G2R9Q8;A0A5G2RKL2 949 sp|P00819|ACYP2_PIG Acylphosphatase-2 OS=Sus scrofa OX=9823 GN=ACYP2 PE=1 SV=2;tr|I3LC10|I3LC10_PIG

Acylphosphatase OS=Sus scrofa OX=9823 GN=ACYP2 PE=3 SV=2;tr|A0A5G2RBE9|A0A5G2RBE9_PIG Acylphosphatase OS=Sus scrofa OX=9823 GN=ACYP2 PE=3 SV=1;tr|A0A5G2R9Q8
0 7892800 13641000 0 0 0 0 0 0 0 0 0 7 7 7 6,5
6,5 6,5 153,67 0 18,527 99537000 10 I3LC73 I3LC73 950 tr|I3LC73|I3LC73_PIG
Beta-ketoacyl-[acyl-carrier-protein] synthase I OS=Sus scrofa OX=9823 GN=FASN PE=1 SV=3
9408200 9538200 7075300 13972000 9549500 9267600 16212000 7002400 9106400 8827600
9191200 8207800 12 12 12 23 23 23 59,975 0 29,287
620520000 58 I3LCA2 I3LCA2 951 tr|I3LCA2|I3LCA2_PIG CCT-theta OS=Sus scrofa OX=9823 GN=CCT8 PE=1 SV=2
0 0 0 0 0 0 0 0 0 0 0 0 3 3 3 6,2 6,2 6,2
64,39 0 7,0331 36098000 4 I3LCW1 I3LCW1 952 tr|I3LCW1|I3LCW1_PIG Fatty acid synthase OS=Sus scrofa OX=9823 GN=FASN PE=1 SV=2
6981600 9016300 24277000 0 7754400 7143500 3268000 3531800 5865600 3267500 0
9684700 5 5 4 9 9 7,4 61,229 0 6,5328 236810000 15
I3LD72 I3LD72 953 tr|I3LD72|I3LD72_PIG EH domain containing 2 OS=Sus scrofa OX=9823 GN=EHD2 PE=1 SV=1
40419000 18176000 46097000 67270000 40806000 44936000 56693000 22337000 46813000 91903000
36570000 75579000 6 6 6 31,3 31,3 31,3 23,52 0 51,191
1717400000 56 I3LDC3 I3LDC3 954 tr|I3LDC3|I3LDC3_PIG Complex I-PDSW OS=Sus scrofa OX=9823 GN=NDUFB10 PE=1 SV=2
204970000 92630000 56868000 120700000 66821000 52229000 234710000 80764000 72953000
192100000 50888000 36299000 5 5 5 17,4 17,4 17,4 20,73 0
9,0788 3490400000 68 I3LDM7;A0A5G2QBC0 I3LDM7 955 tr|I3LDM7|I3LDM7_PIG
Lactoylglutathione lyase OS=Sus scrofa OX=9823 GN=GLO1 PE=1 SV=1
15559000 18629000 19252000 6249300 16850000 0 16420000 11750000 15577000 11581000 14238000
10670000 5 5 4 17,1 17,1 14 37,501 0 17,367 500140000 37
I3LEC2 I3LEC2 956 tr|I3LEC2|I3LEC2_PIG Uncharacterized protein OS=Sus scrofa OX=9823 GN=PCBP1 PE=1 SV=1
0 0 0 0 0 0 0 0 0 0 0 0 1 1 1 2,1 2,1 2,1
48,597 0,0095511 1,3631 0 1 I3LEF2 I3LEF2 957 tr|I3LEF2|I3LEF2_PIG
Nucleoredoxin OS=Sus scrofa OX=9823 GN=NXN PE=1 SV=2
0 0 0 8370700 7651600 0 12229000 0 7257200 8697200 8310900 13488000
5 5 5 10,5 10,5 10,5 56,775 0 8,7213 187120000 16
K7GKH7;I3LEH4;Q6PLK3;A0A287A039;A0A5G2R723;A0A5G2QUP3;A0A5G2QTH6;Q6Q2J0;A0A5G2QF04;A0A5G2R0E5;F1RX00 K7GKH7;I3LEH4;Q6PLK3;A0A287A039 958
tr|K7GKH7|K7GKH7_PIG Amine oxidase OS=Sus scrofa OX=9823 GN=MAOB PE=1 SV=2;tr|I3LEH4|I3LEH4_PIG Amine oxidase OS=Sus scrofa OX=9823 GN=MAOB PE=1 SV=3;sp|Q6PLK3|AOFB_PIG Amine oxidase [flavin-containing] B OS=Sus scrofa OX=9823 GN=MAOB PE=2 SV=3;tr|A0A287A0
13229000 0 0 17677000 0 0 23051000 0 0 0 0 0 3 3 3
18,4 18,4 18,4 21,577 0 6,9274 146170000 12 I3LEI8 I3LEI8 959

tr|I3LEI8|I3LEI8_PIG 1,2-dihydroxy-3-keto-5-methylthiopentene dioxygenase OS=Sus scrofa OX=9823 GN=ADI1 PE=1 SV=1

0 0 835620 0 0 0 0 0 0 0 0 1142000 2 2 1 4,8 4,8 2,7 60,572 0 3,1299 4720000 6 I3LHS9;I3LF61 I3LHS9;I3LF61 960 tr|I3LHS9|I3LHS9_PIG Uncharacterized protein OS=Sus scrofa OX=9823 GN=CYP4F8 PE=3 SV=3;tr|I3LF61|I3LF61_PIG Uncharacterized protein OS=Sus scrofa OX=9823 GN=CYP4F8 PE=3 SV=3

0 0 7497300 6703500 0 0 7135500 0 14916000 10964000 7092600 12169000 2 2 2 18,4 18,4 18,4 14,672 0 7,6145 139300000 8 I3LGM4 I3LGM4 961 tr|I3LGM4|I3LGM4_PIG Complex I-B14.7 OS=Sus scrofa OX=9823 GN=NDUFA11 PE=1 SV=1

42028000 6288000 0 10118000 9236600 0 16510000 68961000 9144600 10185000 25291000 0 8 8 8 27,4 27,4 27,4 55,318 0 72,297 596040000 16 I3LIE7 I3LIE7 962 tr|I3LIE7|I3LIE7_PIG Myosin-binding protein H OS=Sus scrofa OX=9823 GN=MYBPH PE=4 SV=2

56376000 56342000 41623000 16251000 43400000 21993000 34420000 20395000 23480000 17930000 18653000 25286000 22 22 22 24,2 24,2 24,2 95,835 0 54,545 3548100000 131 I3LII3;A0A287A5P3;A0A5G2R2F7;F1RQZ9 I3LII3 963 tr|I3LII3|I3LII3_PIG Eukaryotic translation elongation factor 2 OS=Sus scrofa OX=9823 GN=EEF2 PE=1 SV=3

17942000 0 64419000 8145400 29958000 49484000 0 24932000 31789000 12948000 40250000 106260000 4 4 4 19,7 19,7 19,7 15,368 0 8,2659 730020000 25 I3LJI1 I3LJI1 964 tr|I3LJI1|I3LJI1_PIG Cytochrome c oxidase subunit NDUFA4 OS=Sus scrofa OX=9823 GN=NDUFA4 PE=1 SV=1

0 0 8983500 10481000 8312100 17171000 18319000 0 6275700 11652000 0 28839000 3 3 3 9,2 9,2 9,2 32,4 0 11,084 191330000 12 I3LJJ4;A0A287AE42 I3LJJ4;A0A287AE42 965 tr|I3LJJ4|I3LJJ4_PIG Enoyl-CoA hydratase 1 OS=Sus scrofa OX=9823 GN=ECH1 PE=1 SV=2;tr|A0A287AE42|A0A287AE42_PIG Enoyl-CoA hydratase 1 OS=Sus scrofa OX=9823 GN=ECH1 PE=1 SV=1

0 0 0 0 0 0 0 0 0 0 0 0 1 1 1 6,6 6,6 6,6 21,608 0 3,0683 56806000 2 I3LNC7 I3LNC7 966 tr|I3LNC7|I3LNC7_PIG Protein Hikeshi OS=Sus scrofa OX=9823 GN=HIKESHI PE=1 SV=2

0 0 33631000 0 16191000 0 0 0 0 12981000 10667000 13839000 3 3 3 18,9 18,9 18,9 22,213 0 9,4114 247000000 10 I3LQH7;A0A5G2R5C4 I3LQH7;A0A5G2R5C4 967 tr|I3LQH7|I3LQH7_PIG Biliverdin reductase B OS=Sus scrofa OX=9823 GN=BLVRB PE=1 SV=2;tr|A0A5G2R5C4|A0A5G2R5C4_PIG Biliverdin reductase B OS=Sus scrofa OX=9823 GN=BLVRB PE=1 SV=1

44735000 37700000 32098000 57473000 27535000 35421000 64492000 32455000 35669000 81468000 29983000 34978000 6 6 6 24,2 24,2 24,2 29,048 0 29,561 2022700000 95 I3LR51;A0A287B1N7 I3LR51;A0A287B1N7 968 tr|I3LR51|I3LR51_PIG Peptidylprolyl isomerase OS=Sus scrofa OX=9823 GN=FKBP3 PE=1 SV=2;tr|A0A287B1N7|A0A287B1N7_PIG Peptidylprolyl isomerase OS=Sus scrofa OX=9823 GN=FKBP3 PE=1 SV=1

43457000 10298000 39890000 7787900 11485000 13425000 11882000 7701200 23019000 14404000
9723300 15689000 21 21 21 20,2 20,2 20,2 137,01 0 86,553
1796400000 120 I3LUI1 I3LUI1 969 tr|I3LUI1|I3LUI1_PIG Immunoglobulin like and fibronectin type III domain containing 1 OS=Sus scrofa OX=9823 GN=IGFN1 PE=4 SV=3
0 0 0 0 0 0 0 0 0 0 0 0 4 4 4 10,4 10,4 10,4
51,065 0 4,6046 38969000 5 I3LUM9;A0A5G2RIT4;F1SPY4;A0A287APW7
I3LUM9;A0A5G2RIT4;F1SPY4;A0A287APW7 970 tr|I3LUM9|I3LUM9_PIG Protein-synthesizing GTPase OS=Sus scrofa OX=9823 GN=EIF2S3 PE=1 SV=1;tr|A0A5G2RIT4|A0A5G2RIT4_PIG Protein-synthesizing GTPase OS=Sus scrofa OX=9823 GN=LOC100624149 PE=4 SV=1;tr|F1SPY4|F1SPY4_PIG Protein-synthesizing GTPase OS=Sus scro
67238000 0 53658000 54962000 81972000 60514000 61907000 78168000 56969000 47205000 75424000
45996000 4 4 4 24,9 24,9 24,9 21,768 0 7,3068 1420900000 22
I3LVS7;A0A5G2QN15;F2Z5K4;Q06AT8 I3LVS7;A0A5G2QN15;F2Z5K4 971
tr|I3LVS7|I3LVS7_PIG Ras homolog family member A OS=Sus scrofa OX=9823 GN=RHOA PE=1 SV=1;tr|A0A5G2QN15|A0A5G2QN15_PIG Ras homolog family member C OS=Sus scrofa OX=9823 GN=RHOC PE=1 SV=1;tr|F2Z5K4|F2Z5K4_PIG Ras homolog family member C OS=Sus scrofa OX=9823
39298000 42360000 41935000 36636000 22688000 39114000 36701000 10815000 23182000 21754000
19463000 29862000 14 14 14 43,4 43,4 43,4 38,422 0 94,48
1932800000 95
P19620;K7GKR6;F1S073;A0A287BI04;A0A286ZJV6;A0A5G2QCF9;A0A287AC54
P19620;K7GKR6;F1S073;A0A287BI04;A0A286ZJV6;A0A5G2QCF9 972
sp|P19620|ANXA2_PIG Annexin A2 OS=Sus scrofa OX=9823 GN=ANXA2 PE=1 SV=4;tr|K7GKR6|K7GKR6_PIG Annexin OS=Sus scrofa OX=9823 GN=ANXA2 PE=1 SV=1;tr|F1S073|F1S073_PIG Annexin OS=Sus scrofa OX=9823 GN=ANXA2 PE=1 SV=3;tr|A0A287BI04|A0A287BI04_PIG Annexin OS=Sus
0 0 0 0 0 2678600 0 0 2621500 0 0 3390000 2 2 2
2 2 2 105,3 0,0021668 2,2034 22091000 4 K7GLU6 K7GLU6 973
tr|K7GLU6|K7GLU6_PIG Aminopeptidase OS=Sus scrofa OX=9823 GN=ERAP1 PE=1 SV=3
64089000 21713000 29501000 34296000 14613000 67319000 45616000 16675000 23183000 39044000
16035000 41066000 14 14 14 47,2 47,2 47,2 30,33 0 38,773
1986100000 85 K7GM40;P18648;A0A286ZQC7;CON__P15497
K7GM40;P18648;A0A286ZQC7 974 tr|K7GM40|K7GM40_PIG Apolipoprotein A-I OS=Sus scrofa OX=9823 GN=APOA1 PE=3 SV=2;sp|P18648|APOA1_PIG Apolipoprotein A-I OS=Sus scrofa OX=9823 GN=APOA1 PE=1 SV=4;tr|A0A286ZQC7|A0A286ZQC7_PIG Apolipoprotein A-I OS=Sus scrofa OX=9823 GN=APOA1 PE=3 SV=2
0 0 10509000 0 6791100 7125600 0 0 0 0 0 12940000 3 3
3 13 13 13 30,787 0 6,3312 102630000 11 K7GP28;A0A5G2QSR2
K7GP28;A0A5G2QSR2 975 tr|K7GP28|K7GP28_PIG Hydroxysteroid 17-beta dehydrogenase 10 OS=Sus scrofa OX=9823 GN=HSD17B10 PE=1 SV=1;tr|A0A5G2QSR2|A0A5G2QSR2_PIG Hydroxysteroid 17-beta dehydrogenase 10 OS=Sus scrofa OX=9823 GN=HSD17B10 PE=1 SV=1
55588000 0 94458000 117580000 0 0 107310000 0 0 139640000 0
143700000 2 2 2 11 11 11 14,752 0,0021834 2,2703

1729900000 24 K7GS22 K7GS22 976 tr|K7GS22|K7GS22_PIG Uncharacterized protein OS=Sus scrofa OX=9823 GN=ATP5F1E PE=1 SV=1
15845000 9624400 20372000 22576000 14209000 23386000 13151000 15570000 23588000 28187000 23359000 46268000 4 4 4 19,5 19,5 19,5 26,301 0 15,512 448820000 37 K7GSE5;A0A5G2QSA7;A0A5G2R954 K7GSE5;A0A5G2QSA7;A0A5G2R954 977 tr|K7GSE5|K7GSE5_PIG Complex I-B22 OS=Sus scrofa OX=9823 GN=NDUFB9 PE=1 SV=2;tr|A0A5G2QSA7|A0A5G2QSA7_PIG Complex I-B22 OS=Sus scrofa OX=9823 GN=NDUFB9 PE=1 SV=1;tr|A0A5G2R954|A0A5G2R954_PIG Complex I-B22 OS=Sus scrofa OX=9823 GN=NDUFB9 PE=1 SV=1
0 0 0 27140000 0 0 29024000 0 17783000 26085000 0 0 3 3 3 30,5 30,5 30,5 12,895 0 23,238 257260000 24 K9IVW4;A0A286ZSZ3 K9IVW4;A0A286ZSZ3 978 tr|K9IVW4|K9IVW4_PIG Myotrophin OS=Sus scrofa OX=9823 GN=MTPN PE=1 SV=1;tr|A0A286ZSZ3|A0A286ZSZ3_PIG Myotrophin OS=Sus scrofa OX=9823 GN=MTPN PE=1 SV=1
0 0 5560600 0 0 7423200 0 0 6309000 0 6696600 10825000 3 3 3 8,8 8,8 8,8 54,247 0 13,578 93226000 10 M3UZ54;A0A287AM51;F1SBP8 M3UZ54 979 tr|M3UZ54|M3UZ54_PIG Monocarboxylate transporter 1 OS=Sus scrofa OX=9823 GN=SLC16A1 PE=1 SV=1
206730000 105350000 461700000 479110000 343630000 395020000 446370000 143670000 218440000 540510000 268700000 721660000 8 8 8 48,9 48,9 48,9 14,749 0 29,379 14578000000 126 O02772;A0A287B9U4;F1STV2;I3LTW5 O02772 980 sp|O02772|FABPH_PIG Fatty acid-binding protein, heart OS=Sus scrofa OX=9823 GN=FABP3 PE=2 SV=3
0 0 0 0 0 0 0 0 0 0 0 0 1 1 1 8,9 8,9 8,9 13,79 0 8,7236 50746000 10 O62680 O62680 981 sp|O62680|CD59_PIG CD59 glycoprotein OS=Sus scrofa OX=9823 GN=CD59 PE=1 SV=1
18510000 7935800 22456000 12078000 7567400 17329000 14412000 0 8839000 25660000 0 15367000 9 9 9 20,1 20,1 20,1 59,771 0 20,015 495550000 45 O62839;F1SGS9;A0A287A2C3 O62839;F1SGS9;A0A287A2C3 982 sp|O62839|CATA_PIG Catalase OS=Sus scrofa OX=9823 GN=CAT PE=1 SV=4;tr|F1SGS9|F1SGS9_PIG Catalase OS=Sus scrofa OX=9823 GN=CAT PE=1 SV=3;tr|A0A287A2C3|A0A287A2C3_PIG Catalase OS=Sus scrofa OX=9823 GN=CAT PE=1 SV=1
0 0 5188300 3441700 0 0 3691100 0 0 6771600 0 14944000 1 1 1 13 13 13 12,993 0,0022297 2,4467 90726000 5 O79880 O79880 983 sp|O79880|NU3M_PIG NADH-ubiquinone oxidoreductase chain 3 OS=Sus scrofa OX=9823 GN=MT-ND3 PE=1 SV=1
0 0 0 0 8058200 11984000 0 0 0 0 0 0 2 2 2 3,1 3,1 3,1 51,824 0,0031153 1,9016 69662000 2 O79881 O79881 984 sp|O79881|NU4M_PIG NADH-ubiquinone oxidoreductase chain 4 OS=Sus scrofa OX=9823 GN=MT-ND4 PE=1 SV=2
39770000 39416000 40768000 8614600 8295600 23591000 22476000 6890500 21102000 19098000 7924100 27945000 7 7 6 44,7 44,7 35,6 14,676 0 29,631

1196300000 62 O97788 O97788 985 sp|O97788|FABP4_PIG Fatty acid-binding protein, adipocyte OS=Sus scrofa OX=9823 GN=FABP4 PE=1 SV=3
3829500000 12116000000 5243700000 4996300000 10150000000 5632200000 5184100000
15817000000 6705700000 3374600000 11208000000 3693800000 16 16
14 53,5 53,5 46,2 35,836 0 323,31 341230000000 2284
P00355;A0A287BG23;A0A286ZVK5;A0A5S8K8F1;A0A286ZQE4
P00355;A0A287BG23;A0A286ZVK5;A0A5S8K8F1;A0A286ZQE4 986 sp|P00355|G3P_PIG Glyceraldehyde-3-phosphate dehydrogenase OS=Sus scrofa OX=9823 GN=GAPDH PE=1 SV=4;tr|A0A287BG23|A0A287BG23_PIG Glyceraldehyde-3-phosphate dehydrogenase OS=Sus scrofa OX=9823 GN=GAPDH PE=3 SV=1;tr|A0A286ZVK5|A0A286ZVK5_PIG Glyceraldehyde-3
59538000 97450000 320370000 116070000 228510000 229300000 73590000 138960000
151500000 107070000 252030000 399660000 21 21 21 59,8 59,8
59,8 46,474 0 158,67 16166000000 261 P00503 P00503 987 sp|P00503|AATC_PIG Aspartate aminotransferase, cytoplasmic OS=Sus scrofa OX=9823 GN=GOT1 PE=1 SV=3
42968000 70924000 132460000 99586000 133050000 107890000 67527000 84872000
119350000 107510000 126790000 184430000 14 14 14 27,4 27,4
27,4 51,629 0 49,746 7493400000 173 P00889;A0A286ZKX2 P00889;A0A286ZKX2
988 sp|P00889|CISY_PIG Citrate synthase, mitochondrial OS=Sus scrofa OX=9823 GN=CS PE=1 SV=2;tr|A0A286ZKX2|A0A286ZKX2_PIG Citrate synthase OS=Sus scrofa OX=9823 GN=CS PE=1 SV=2
2238900000 1332600000 5032100000 6082200000 3052600000 5189700000 4587900000
2366000000 2593100000 7130300000 3528900000 9329500000 11 11
11 61 61 61 17,084 0 174,36 155980000000 2282 P02189 P02189 989
sp|P02189|MYG_PIG Myoglobin OS=Sus scrofa OX=9823 GN=MB PE=1 SV=2
0 0 10276000 0 0 0 0 0 0 0 15394000 0 4 4 4 9,3
9,3 9,3 53,628 0 12,911 81204000 8 P02540;A0A287BSR0 P02540;A0A287BSR0
990 sp|P02540|DESM_PIG Desmin OS=Sus scrofa OX=9823 GN=DES PE=1 SV=4;tr|A0A287BSR0|A0A287BSR0_PIG Desmin OS=Sus scrofa OX=9823 GN=DES PE=1 SV=1
0 2918100 0 0 0 0 0 91711000 6091700 0 12103000 1969500 10
1 0 56 7,5 0 18,025 0 6,6917 288670000 22 P02587 P02587 991
sp|P02587|TNNC2_PIG Troponin C, skeletal muscle OS=Sus scrofa OX=9823 GN=TNNC2 PE=1 SV=2
124760000 80971000 127590000 250940000 111160000 169770000 251270000
100500000 99358000 215250000 117610000 165550000 5 5 5
37,9 37,9 37,9 15,892 0 14,094 5037800000 98 P04178;A0A2C9F3F0
P04178;A0A2C9F3F0 992 sp|P04178|SODC_PIG Superoxide dismutase [Cu-Zn] OS=Sus scrofa OX=9823 GN=SOD1 PE=1 SV=2;tr|A0A2C9F3F0|A0A2C9F3F0_PIG Superoxide dismutase [Cu-Zn] OS=Sus scrofa OX=9823 GN=SOD1 PE=3 SV=2
138210000 131680000 119320000 134590000 108650000 189250000 138450000
88445000 70460000 141270000 124040000 123590000 41 41 35 57
57 47,3 76,967 0 323,31 19159000000 583
P09571;A0A288CFV5;A0A5G2R5N9;A0A5G2RBB4;A0A5G2QUQ4;Q6YT39;P14632;CON__Q2HJF0 P09571;A0A288CFV5;A0A5G2R5N9;A0A5G2RBB4 994 sp|P09571|TRFE_PIG

Serotransferrin OS=Sus scrofa OX=9823 GN=TF PE=1 SV=2;tr|A0A288CFV5|A0A288CFV5_PIG Beta-1 metal-binding globulin OS=Sus scrofa OX=9823 GN=TF PE=3 SV=2;tr|A0A5G2R5N9|A0A5G2R5N9_PIG Beta-1 metal-binding globulin OS=Sus scrofa OX=9823 GN=TF

7790200 7300700 9059800 19947000 6626200 8524100 14693000 12972000 13860000 18503000 7434400 9491000 3 3 3 24,5 24,5 24,5 11,828 0 13,263 320480000 34 P12309 P12309 995 sp|P12309|GLRX1_PIG Glutaredoxin-1 OS=Sus scrofa OX=9823 GN=GLRX PE=1 SV=2

55621000 32648000 46020000 113130000 50903000 47356000 107450000 37022000 45863000 155440000 53870000 104020000 8 8 8 75 75 75 8,9299 0 81,883 2510700000 85 P13618;A0A287ACM8;A0A287B441 P13618;A0A287ACM8;A0A287B441 996 sp|P13618|ATP5J_PIG ATP synthase-coupling factor 6, mitochondrial OS=Sus scrofa OX=9823 GN=ATP5PF PE=1 SV=1;tr|A0A287ACM8|A0A287ACM8_PIG ATP synthase-coupling factor 6, mitochondrial OS=Sus scrofa OX=9823 GN=ATP5PF PE=3 SV=1;tr|A0A287B441|A0A287B441_PIG AT

0 0 0 0 0 0 0 0 0 0 0 0 2 2 2 7,1 7,1 7,1 28,054 0,0031088 1,8846 0 2 P15980;A0A287BMG0;A0A287B797;A0A286ZN51;Q7YQ94;P15981 P15980;A0A287BMG0;A0A287B797;A0A286ZN51;Q7YQ94;P15981 997 sp|P15980|HA2C_PIG SLA class II histocompatibility antigen, DQ haplotype C alpha chain OS=Sus scrofa OX=9823 PE=2 SV=1;tr|A0A287BMG0|A0A287BMG0_PIG Ig-like domain-containing protein OS=Sus scrofa OX=9823 GN=SLA-DQA1 PE=4 SV=1;tr|A0A287B797|A0A287B797_PIG I

0 0 0 8405100 0 0 9100700 0 0 9611600 0 0 2 2 2 7,4 7,4 7,4 29,262 0,0050251 1,6755 51877000 4 P15983;F6PX38;Q8MGP9;P15982;A0A5G2QCN4 P15983;F6PX38;Q8MGP9;P15982;A0A5G2QCN4 998 sp|P15983|HB2D_PIG SLA class II histocompatibility antigen, DQ haplotype D beta chain OS=Sus scrofa OX=9823 PE=2 SV=1;tr|F6PX38|F6PX38_PIG Ig-like domain-containing protein OS=Sus scrofa OX=9823 GN=SLA-DQB1 PE=1 SV=1;tr|Q8MGP9|Q8MGP9_PIG HLA class II histo

52695000 108410000 195180000 119770000 159900000 170090000 89141000 106450000 175150000 95165000 169360000 308550000 27 27 1 41 41 1,9 85,76 0 233,83 19984000000 457 P16276 P16276 999 sp|P16276|ACON_PIG Aconitate hydratase, mitochondrial OS=Sus scrofa OX=9823 GN=ACO2 PE=1 SV=1

10441000 40360000 35091000 508910 23311000 37869000 0 31438000 40460000 1817500 28281000 25674000 63 63 62 14,7 14,7 14,5 565,33 0 270,15 5013300000 389 P16960 P16960 1000 sp|P16960|RYR1_PIG Ryanodine receptor 1 OS=Sus scrofa OX=9823 GN=RYR1 PE=2 SV=2

9489600 0 5586800 9579200 0 0 10998000 0 0 8774600 0 4573400 2 1 1 50 33,3 33,3 4,8234 0 5,7124 111650000 12 P21753 P21753 1001 sp|P21753|TYB10_PIG Thymosin beta-10 OS=Sus scrofa OX=9823 GN=TMSB10 PE=1 SV=2

0 0 0 0 0 0 0 0 0 0 0 0 1 1 1 2,4 2,4 2,4 42,811 0,0022198 2,3866 95864000 12 Q1HBG9;P24964 Q1HBG9;P24964 1002

tr|Q1HBG9|Q1HBG9_PIG Cytochrome b OS=Sus scrofa OX=9823 GN=cytb PE=1 SV=1;sp|P24964|CYB_PIG Cytochrome b OS=Sus scrofa OX=9823 GN=MT-CYB PE=3 SV=2
31965000 19562000 19743000 27541000 18412000 28162000 32538000 14778000 20541000 26114000 17048000 20277000 32 32 32 32,1 32,1 32,1 123,94 0 138,46 3005700000 182 P26234;A0A5G2RBX6;A0A2C9F393;A0A287BSL4 P26234;A0A5G2RBX6;A0A2C9F393;A0A287BSL4 1003 sp|P26234|VINC_PIG Vinculin OS=Sus scrofa OX=9823 GN=VCL PE=1 SV=4;tr|A0A5G2RBX6|A0A5G2RBX6_PIG Metavinculin OS=Sus scrofa OX=9823 GN=VCL PE=1 SV=1;tr|A0A2C9F393|A0A2C9F393_PIG Metavinculin OS=Sus scrofa OX=9823 GN=VCL PE=1 SV=1;tr|A0A287BSL4|A0A287BSL4_PI
25165000 10323000 16006000 5419200 11099000 10996000 30105000 6410000 11536000 25265000 8093600 7351400 9 9 9 23,3 23,3 23,3 48,287 0 43,3 903540000 33 P28491;A0A287B0P6;A0A287AG29 P28491;A0A287B0P6;A0A287AG29 1004 sp|P28491|CALR_PIG Calreticulin OS=Sus scrofa OX=9823 GN=CALR PE=1 SV=3;tr|A0A287B0P6|A0A287B0P6_PIG Calreticulin OS=Sus scrofa OX=9823 GN=CALR PE=1 SV=1;tr|A0A287AG29|A0A287AG29_PIG Calreticulin OS=Sus scrofa OX=9823 GN=CALR PE=1 SV=2
7068400 5792400 7156700 6925400 5699600 0 6271600 5038200 7483500 0 6412600 6498600 2 2 2 13,6 13,6 13,6 14,515 0,0050201 1,6607 144910000 13 P46405 P46405 1005 sp|P46405|RS12_PIG 40S ribosomal protein S12 OS=Sus scrofa OX=9823 GN=RPS12 PE=1 SV=2
0 0 0 0 0 0 0 0 0 0 0 0 1 1 1 2,7 2,7 2,7 42,03 0 2,9988 40064000 3 P46410 P46410 1006 sp|P46410|GLNA_PIG Glutamine synthetase OS=Sus scrofa OX=9823 GN=GLUL PE=2 SV=3
42204000 96723000 198180000 115100000 240410000 206930000 67534000 142630000 232020000 79839000 204740000 308900000 5 5 5 21,9 21,9 21,9 26,184 0 18,821 4238900000 105 P50667;Q69GF7 P50667;Q69GF7 1007 sp|P50667|COX2_PIG Cytochrome c oxidase subunit 2 OS=Sus scrofa OX=9823 GN=MT-CO2 PE=3 SV=1;tr|Q69GF7|Q69GF7_PIG Cytochrome c oxidase subunit 2 OS=Sus scrofa OX=9823 GN=MT-CO2 PE=1 SV=1
50004000 25102000 57089000 40497000 20716000 79574000 49681000 56908000 30060000 45823000 51108000 48258000 8 8 8 22,4 22,4 22,4 51,305 0 23,341 1368900000 56 P50828;A0A5G2QGR8;F1RMN7;A0A5G2QRQ8;A0A5G2QSB7 P50828;A0A5G2QGR8;F1RMN7;A0A5G2QRQ8;A0A5G2QSB7 1008 sp|P50828|HEMO_PIG Hemopexin OS=Sus scrofa OX=9823 GN=HPX PE=1 SV=1;tr|A0A5G2QGR8|A0A5G2QGR8_PIG Hemopexin OS=Sus scrofa OX=9823 GN=HPX PE=3 SV=1;tr|F1RMN7|F1RMN7_PIG Hemopexin OS=Sus scrofa OX=9823 GN=HPX PE=3 SV=3;tr|A0A5G2QRQ8|A0A5G2QRQ8_PIG Hemopexin O
39445000 38349000 46882000 0 39330000 11656000 10667000 13133000 13562000 0 11922000 37955000 4 4 4 36,5 36,5 36,5 13,931 0 8,2631 481150000 31 P59083 P59083 1009 sp|P59083|PHP14_PIG 14 kDa phosphohistidine phosphatase OS=Sus scrofa OX=9823 GN=PHPT1 PE=1 SV=2
16304000 0 57122000 50880000 53586000 70608000 48816000 16812000 34081000 39209000 35552000 86210000 1 1 1 21,2 21,2 21,2 6,0804 0 6,7773 881900000 56

P61013 P61013 1010 sp|P61013|PPLA_PIG Cardiac phospholamban OS=Sus scrofa OX=9823 GN=PLN PE=1 SV=1
56053000 0 10946000 16766000 3256800 18180000 8499100 5329900 7485200 30457000 6633400 9110100 6 6 6 53,4 53,4 53,4 11,367 0 15,554 628570000 38
P62802 P62802 1011 sp|P62802|H4_PIG Histone H4 OS=Sus scrofa OX=9823 PE=1 SV=2
108040000 105790000 32827000 14297000 49775000 16240000 62348000 71241000 55991000 41985000 50013000 24344000 6 6 5 36,6 36,6 31,1 17,869 0 17,672 1525600000 72
P62936;A0A5S6GHG5;A0A5G2QWG7;A0A5G2QIR3;A0A287AH51;A0A287A343
P62936;A0A5S6GHG5;A0A5G2QWG7;A0A5G2QIR3 1012 sp|P62936|PPIA_PIG Peptidyl-prolyl cis-trans isomerase A OS=Sus scrofa OX=9823 GN=PPIA PE=1 SV=2;tr|A0A5S6GHG5|A0A5S6GHG5_PIG Peptidyl-prolyl cis-trans isomerase OS=Sus scrofa OX=9823 GN=PPIA PE=3 SV=1;tr|A0A5G2QWG7|A0A5G2QWG7_PIG Peptidyl-prolyl cis-trans
0 0 0 0 0 5648400 0 0 0 6607600 0 0 1 1 1 12 12 12 9,1113 0,0031056 1,8713 70087000 6 P63221 P63221 1013
sp|P63221|RS21_PIG 40S ribosomal protein S21 OS=Sus scrofa OX=9823 GN=RPS21 PE=1 SV=1
0 0 9373600 0 0 11322000 0 0 9374000 0 7638700 0 3 3 3 10,7 10,7 10,7 35,076 0 15,936 99804000 12 P63246;A0A286ZLL3
P63246;A0A286ZLL3 1014 sp|P63246|RACK1_PIG Receptor of activated protein C kinase 1 OS=Sus scrofa OX=9823 GN=RACK1 PE=1 SV=3;tr|A0A286ZLL3|A0A286ZLL3_PIG Receptor of-activated protein C kinase 1 OS=Sus scrofa OX=9823 GN=RACK1 PE=1 SV=2
103130000 12976000 255910000 123830000 74272000 145280000 64880000 46558000 74323000 54985000 75393000 249990000 5 5 5 31,1 31,1 31,1 18,416 0 83,618 2551700000 65 P63317;A0A287AIU7 P63317;A0A287AIU7 1015
sp|P63317|TNNC1_PIG Troponin C, slow skeletal and cardiac muscles OS=Sus scrofa OX=9823 GN=TNNC1 PE=1 SV=1;tr|A0A287AIU7|A0A287AIU7_PIG Troponin C, slow skeletal and cardiac muscles OS=Sus scrofa OX=9823 GN=TNNC1 PE=1 SV=1
0 0 0 0 0 0 0 0 0 0 0 0 12 1 0 35,1 5,6 0 28,521 0 5,6721 25825000 3 P67937;A0A5G2QY33 P67937;A0A5G2QY33 1016
sp|P67937|TPM4_PIG Tropomyosin alpha-4 chain OS=Sus scrofa OX=9823 GN=TPM4 PE=2 SV=3;tr|A0A5G2QY33|A0A5G2QY33_PIG Tropomyosin alpha-4 chain OS=Sus scrofa OX=9823 GN=TPM4 PE=1 SV=1
902310000 2213700000 607520000 1748100000 853630000 2026800000 696370000 1215800000 632640000 558470000 814000000 492440000 16 16 8 58,4 58,4 36,3 42,051 0 159,18 76893000000 634
P68137;A0A5S6G831;A0A287BF33;A0A5S6IE01;A0A5G2QKZ4;F6Q364;B6VNT8;C7AI81;A0A287AXB6;A0A287AVS8;A0A5G2R0X5;A0A480K2S5;A0A287A6R7;A0A5G2QX85;F1SLG5;A0A286ZWJ1;A0A287BGY0;A0A5G2QZ37;A0A5G2QIZ5;A0A5G2QY90;A0A287B0H6;A0A5G2QID5
P68137;A0A5S6G831;A0A287BF33;A0A5S6IE01;A0A5G2QKZ4;F6Q364;B6VNT8;C7AI81;A0A287AXB6;A0A287AVS8;A0A5G2R0X5;A0A480K2S5;A0A287A6R7;A0A5G2QX85;F1SLG5;A0A286ZWJ1;A0A287BGY0;A0A5G2QZ37;A0A5G2QIZ5;A0A5G2QY90;A0A287B0H6;A0A5G2QID5 1017
sp|P68137|ACTS_PIG Actin, alpha skeletal muscle OS=Sus scrofa OX=9823 GN=ACTA1 PE=1

SV=1;tr|A0A5S6G831|A0A5S6G831_PIG Actin, alpha skeletal muscle OS=Sus scrofa OX=9823 GN=ACTA1 PE=3 SV=1;tr|A0A287BF33|A0A287BF33_PIG Actin, alpha skeletal muscle OS=Sus scr
12548000 13459000 23053000 17361000 28803000 17504000 12478000 20783000 23309000 21741000
28350000 47092000 10 10 10 25,3 25,3 25,3 47,956 0 86,026
959590000 52 P79274 P79274 1018 sp|P79274|ACADL_PIG Long-chain specific acyl-CoA dehydrogenase, mitochondrial OS=Sus scrofa OX=9823 GN=ACADL PE=2 SV=1
48396000 37134000 87002000 51200000 58290000 98531000 42911000 42645000 56270000 53691000
51954000 104770000 12 12 12 38,3 38,3 38,3 35,868 0 43,096
4382400000 92 P80276;A0A287A5M7;A0A5G2R274;A0A5S6HCI3;A0A286ZQQ7;P82125
P80276;A0A287A5M7;A0A5G2R274 1019 sp|P80276|ALDR_PIG Aldo-keto reductase family 1 member B1 OS=Sus scrofa OX=9823 GN=AKR1B1 PE=1 SV=2;tr|A0A287A5M7|A0A287A5M7_PIG Aldo-keto reductase family 1 member B1 OS=Sus scrofa OX=9823 GN=AKR1B1 PE=3 SV=2;tr|A0A5G2R274|A0A5G2R274_PIG Aldo-keto reducta
29692000 45023000 0 39577000 0 34389000 41406000 0 0 49974000 0 34953000
7 7 7 24,6 24,6 24,6 30,83 0 13,828 553490000 30
P83686;A0A5G2QPB6;A0A5G2R3P5;F1SJQ5 P83686;A0A5G2QPB6;A0A5G2R3P5;F1SJQ5
1020 sp|P83686|NB5R3_PIG NADH-cytochrome b5 reductase 3 (Fragment) OS=Sus scrofa OX=9823 GN=CYB5R3 PE=1 SV=1;tr|A0A5G2QPB6|A0A5G2QPB6_PIG NADH-cytochrome b5 reductase OS=Sus scrofa OX=9823 GN=CYB5R3 PE=1 SV=1;tr|A0A5G2R3P5|A0A5G2R3P5_PIG Cytochrome-b5 reductase
0 0 0 0 0 0 0 0 0 0 0 0 2 1 1 11 5,2 5,2
21,258 0 3,2823 199100000 11 Q007T2 Q007T2 1021 sp|Q007T2|CDC42_PIG Cell division control protein 42 homolog OS=Sus scrofa OX=9823 GN=CDC42 PE=2 SV=2
0 0 0 0 0 0 0 0 0 0 0 0 1 1 1 6,4 6,4 6,4
22,724 0,0040241 1,7138 9123900 1 Q03472 Q03472 1022 sp|Q03472|APOR_PIG Apolipoprotein R OS=Sus scrofa OX=9823 GN=APOR PE=1 SV=1
336790000 237490000 240950000 576180000 225340000 253750000 588500000
226390000 223920000 561280000 261320000 245010000 14 14
14 63,5 63,5 63,5 19,936 0 58,571 21992000000 353 Q0R678;A0A5G2QIK1
Q0R678;A0A5G2QIK1 1023 tr|Q0R678|Q0R678_PIG Maillard deglycase OS=Sus scrofa OX=9823 GN=PARK7 PE=1 SV=1;tr|A0A5G2QIK1|A0A5G2QIK1_PIG Parkinsonism associated deglycase OS=Sus scrofa OX=9823 GN=PARK7 PE=1 SV=1
3604800 0 28985000 0 12027000 8842300 0 6171600 9823800 0 7101100 47606000
8 8 8 34,5 34,5 34,5 29,937 0 51,182 427430000 35 Q1AG08 Q1AG08
1024 tr|Q1AG08|Q1AG08_PIG Calsarcin 1 OS=Sus scrofa OX=9823 GN=MYOZ2 PE=2 SV=1
6991300000 8858000000 2948500000 11813000000 6854800000 3795900000 12416000000
9554700000 5456700000 8115800000 6750000000 2324000000 28 28
19 56,2 56,2 36,9 47,13 0 323,31 472320000000 2080
Q1KYT0;A0A5G2R7R6;F1RFY2;A0A286ZSQ1;A0A287AZR0;A0A5G2RCS9;A0A5G2R1M2
Q1KYT0;A0A5G2R7R6;F1RFY2;A0A286ZSQ1;A0A287AZR0;A0A5G2RCS9;A0A5G2R1M2 1025
sp|Q1KYT0|ENOB_PIG Beta-enolase OS=Sus scrofa OX=9823 GN=ENO3 PE=2 SV=1;tr|A0A5G2R7R6|A0A5G2R7R6_PIG 2-phospho-D-glycerate hydro-lyase OS=Sus scrofa

OX=9823 GN=ENO3 PE=1 SV=1;tr|F1RFY2|F1RFY2_PIG 2-phospho-D-glycerate hydro-lyase OS=Sus scrofa OX=9823 GN=E
51342000 0 80777000 85103000 107210000 108720000 70304000 0 61267000 116280000
97139000 173090000 2 2 2 28,6 28,6 28,6 7,3466 0 9,1753
1672000000 31 Q1W0Y2 Q1W0Y2 1026 sp|Q1W0Y2|COX7C_PIG Cytochrome c oxidase subunit 7C, mitochondrial OS=Sus scrofa OX=9823 GN=COX7C PE=1 SV=1
5037600 0 4950600 0 0 0 5841200 0 0 6378300 0 0 3 3
3 28,3 28,3 28,3 12,511 0 3,6526 60012000 3 Q29036;A0A5G2QH37;A0A5S6FUV7
Q29036;A0A5G2QH37 1027 sp|Q29036|DAD1_PIG Dolichyl-diphosphooligosaccharide--protein glycosyltransferase subunit DAD1 OS=Sus scrofa OX=9823 GN=DAD1 PE=3 SV=3;tr|A0A5G2QH37|A0A5G2QH37_PIG Dolichyl-diphosphooligosaccharide--protein glycosyltransferase subunit DAD1 OS=Sus scrofa OX
0 0 6237500 9285700 0 9030700 0 0 0 4684900 0 4068400 3
3 3 18,5 18,5 18,5 16,445 0 6,2933 77688000 23 Q29201;K7GKC0;A0A5S6GW77
Q29201;K7GKC0;A0A5S6GW77 1028 sp|Q29201|RS16_PIG 40S ribosomal protein S16 OS=Sus scrofa OX=9823 GN=RPS16 PE=1 SV=4;tr|K7GKC0|K7GKC0_PIG 40S ribosomal protein S16 OS=Sus scrofa OX=9823 GN=RPS16 PE=3 SV=2;tr|A0A5S6GW77|A0A5S6GW77_PIG 40S ribosomal protein S16 OS=Sus scrofa OX=9823 GN=RP
0 0 14603000 30928000 0 31232000 30744000 0 0 27564000 30224000 0
2 2 1 11,8 11,8 7,9 20,212 0 6,4568 314810000 34 Q29205 Q29205 1029
sp|Q29205|RL11_PIG 60S ribosomal protein L11 OS=Sus scrofa OX=9823 GN=RPL11 PE=1 SV=3
46869000 30304000 28336000 76837000 25301000 35841000 78725000 23303000 24198000 67355000
26175000 34071000 10 10 10 25,1 25,1 25,1 53,941 0 39,766
2377000000 99 Q29228;F1S232 Q29228;F1S232 1030 sp|Q29228|AL9A1_PIG 4-trimethylaminobutyraldehyde dehydrogenase OS=Sus scrofa OX=9823 GN=ALDH9A1 PE=2 SV=2;tr|F1S232|F1S232_PIG 4-trimethylaminobutyraldehyde dehydrogenase OS=Sus scrofa OX=9823 GN=ALDH9A1 PE=1 SV=3
19073000 0 0 0 0 0 0 0 0 23498000 0 0 2 2 2 19,4
19,4 19,4 11,13 0 3,6754 135900000 5 Q29290 Q29290 1031
sp|Q29290|CYTB_PIG Cystatin-B OS=Sus scrofa OX=9823 GN=CSTB PE=1 SV=1
0 0 0 0 0 0 0 0 0 0 0 0 0 1 1 1 8,1 8,1 8,1
14,549 0,0011312 2,5695 53248000 2 Q29361 Q29361 1032 sp|Q29361|RL35_PIG 60S ribosomal protein L35 OS=Sus scrofa OX=9823 GN=RPL35 PE=1 SV=3
8523500 9900800 21510000 8266900 9563900 21949000 10619000 0 15368000 13558000 12343000
35123000 6 6 6 9,4 9,4 9,4 56,337 0 11,92 388380000 21
Q29551;A0A5G2Q8L3;A0A5G2QI86;A0A5G2RD73;A0A5G2RCZ1;A0A5S6IB20;M9MMN4
Q29551;A0A5G2Q8L3;A0A5G2QI86;A0A5G2RD73;A0A5G2RCZ1;A0A5S6IB20 1033
sp|Q29551|SCOT1_PIG Succinyl-CoA:3-ketoacid coenzyme A transferase 1, mitochondrial OS=Sus scrofa OX=9823 GN=OXCT1 PE=1 SV=2;tr|A0A5G2Q8L3|A0A5G2Q8L3_PIG Succinyl-CoA:3-ketoacid-coenzyme A transferase OS=Sus scrofa OX=9823 GN=OXCT1 PE=1 SV=1;tr|A0A5G2QI86|
0 58338000 0 128500000 0 0 101910000 0 89994000 166480000 85996000 0
2 2 2 39,1 39,1 39,1 7,4004 0 5,1559 1291300000 19 Q2EN79

Q2EN79 1034 tr|Q2EN79|Q2EN79_PIG Complex III subunit 9 OS=Sus scrofa OX=9823 GN=UQCR10 PE=1 SV=1
298490000 561140000 367340000 763850000 709910000 365660000 676980000 706880000 667860000 700370000 752620000 775960000 13 13 13 32,7 32,7 32,7 47,478 0 183,37 21734000000 610
Q2HYU1;F6PU58;A0A287AKB5;A0A286ZQI3;A0A287BBV9;A0A286ZI31;Q29577;F1SI77
Q2HYU1;F6PU58;A0A287AKB5;A0A286ZQI3 1035 tr|Q2HYU1|Q2HYU1_PIG Creatine kinase OS=Sus scrofa OX=9823 GN=CKMT2 PE=1 SV=1;tr|F6PU58|F6PU58_PIG Creatine kinase OS=Sus scrofa OX=9823 GN=CKMT2 PE=1 SV=1;tr|A0A287AKB5|A0A287AKB5_PIG Creatine kinase OS=Sus scrofa OX=9823 GN=CKMT2 PE=1 SV=1;tr|A0A286ZQI3|
0 0 0 0 0 0 0 0 0 0 0 0 1 1 1 4,9 4,9 4,9 25,093 0,0011455 2,6841 110720000 11 Q2MJQ7 Q2MJQ7 1036
sp|Q2MJQ7|CCG1_PIG Voltage-dependent calcium channel gamma-1 subunit OS=Sus scrofa OX=9823 GN=CACNG1 PE=2 SV=1
0 0 0 0 0 0 0 0 0 0 0 0 2 2 2 35,8 35,8 35,8 7,9474 0 4,7584 204530000 10 Q35914 Q35914 1037 sp|Q35914|ATP8_PIG ATP synthase protein 8 OS=Sus scrofa OX=9823 GN=MT-ATP8 PE=1 SV=2
0 5511200 5572800 0 0 0 0 0 3836300 0 5325000 10324000 1 1 1 4 4 4 25,039 0 4,571 97019000 9 Q35915 Q35915 1038
sp|Q35915|ATP6_PIG ATP synthase subunit a OS=Sus scrofa OX=9823 GN=MT-ATP6 PE=1 SV=2
0 0 36334000 0 0 37078000 0 0 31017000 0 0 62677000 1 1 1 5,4 5,4 5,4 29,728 0 30,536 473910000 18 Q35916 Q35916 1039
sp|Q35916|COX3_PIG Cytochrome c oxidase subunit 3 OS=Sus scrofa OX=9823 GN=MT-CO3 PE=3 SV=2
0 0 0 0 0 0 0 0 0 0 0 0 4 4 4 6,2 6,2 6,2 74,218 0 5,9611 27111000 8
Q3ZD69;F1RLQ2;A0A287AE06;A0A480NRZ3;A0A287B1C7;A0A287BCB7
Q3ZD69;F1RLQ2;A0A287AE06;A0A480NRZ3;A0A287B1C7;A0A287BCB7 1040
sp|Q3ZD69|LMNA_PIG Prelamin-A/C OS=Sus scrofa OX=9823 GN=LMNA PE=3 SV=1;tr|F1RLQ2|F1RLQ2_PIG Prelamin-A/C OS=Sus scrofa OX=9823 GN=LMNA PE=1 SV=2;tr|A0A287AE06|A0A287AE06_PIG Lamin isoform A OS=Sus scrofa OX=9823 GN=LMNA PE=1 SV=1;tr|A0A480NRZ3|A0A480NRZ3_
33289000 30080000 27479000 23004000 17836000 32351000 26934000 0 23530000 33110000 16474000 23249000 3 3 1 12,6 12,6 6 17,367 0 4,0371 515760000 17
Q3ZDQ5;A0A286ZW86 Q3ZDQ5;A0A286ZW86 1041 sp|Q3ZDQ5|CAV3_PIG Caveolin-3 OS=Sus scrofa OX=9823 GN=CAV3 PE=2 SV=1;tr|A0A286ZW86|A0A286ZW86_PIG Caveolin OS=Sus scrofa OX=9823 GN=CAV1 PE=3 SV=2
39918000 14941000 44578000 6780600 22494000 18409000 21870000 70464000 13810000 13072000 48212000 13358000 6 6 6 27,9 27,9 27,9 31,667 0 113,55 810440000 36 Q4PS85;A0A2C9F381 Q4PS85;A0A2C9F381 1042
sp|Q4PS85|MYOZ1_PIG Myozenin-1 OS=Sus scrofa OX=9823 GN=MYOZ1 PE=2

SV=1;tr|A0A2C9F381|A0A2C9F381_PIG Myozenin-1 OS=Sus scrofa OX=9823 GN=MYOZ1 PE=3 SV=2
28271000 0 7320500 26569000 8271500 11360000 27030000 0 0 21146000 0 11361000
3 3 3 16,3 16,3 16,3 23,927 0 6,7128 245080000 14
Q52NJ6;A0A287BPB8 Q52NJ6;A0A287BPB8 1043 sp|Q52NJ6|RAB14_PIG Ras-related protein Rab-14 OS=Sus scrofa OX=9823 GN=RAB14 PE=2 SV=3;tr|A0A287BPB8|A0A287BPB8_PIG Ras-related protein Rab-14 OS=Sus scrofa OX=9823 GN=RAB14 PE=4 SV=1
100240000 73695000 120880000 41028000 111910000 95543000 62685000 83708000 84328000
71799000 90415000 126750000 7 7 5 44 44 33,1 18,736 0
128,53 3258400000 70 Q5G6V9 Q5G6V9 1044 sp|Q5G6V9|COF2_PIG Cofilin-2 OS=Sus scrofa OX=9823 GN=CFL2 PE=2 SV=4
19073000 9585400 44799000 0 24070000 25437000 0 5440700 13275000 0 16926000 57419000
13 13 13 45,5 45,5 45,5 40,659 0 47,723 1508200000 69
Q5PXT2;A0A5G2Q8A7;A0A5G2QFD7;A0A5G2QVX1;A0A5G2R4Z6;A0A5G2QVD0;A0A5G2QTN0
Q5PXT2;A0A5G2Q8A7;A0A5G2QFD7;A0A5G2QVX1;A0A5G2R4Z6;A0A5G2QVD0;A0A5G2QTN0 1045 sp|Q5PXT2|LMCD1_PIG LIM and cysteine-rich domains protein 1 OS=Sus scrofa OX=9823 GN=LMCD1 PE=2 SV=1;tr|A0A5G2Q8A7|A0A5G2Q8A7_PIG LIM and cysteine-rich domains protein 1 OS=Sus scrofa OX=9823 GN=LMCD1 PE=1 SV=1;tr|A0A5G2QFD7|A0A5G2QFD7_PIG LIM and cysteine
1657300000 3217600000 1151300000 1505800000 5355600000 2016200000 1524300000
4446700000 2973100000 1631500000 4365600000 1363700000 14 14
14 62,3 62,3 62,3 29,411 0 323,31 141540000000 1203
Q5S1S4;A0A5S6G0V8;A0A5S6I8A2 Q5S1S4;A0A5S6G0V8;A0A5S6I8A2 1046
sp|Q5S1S4|CAH3_PIG Carbonic anhydrase 3 OS=Sus scrofa OX=9823 GN=CA3 PE=2 SV=3;tr|A0A5S6G0V8|A0A5S6G0V8_PIG Carbonic anhydrase OS=Sus scrofa OX=9823 GN=CA3 PE=3 SV=1;tr|A0A5S6I8A2|A0A5S6I8A2_PIG Carbonic anhydrase OS=Sus scrofa OX=9823 GN=CA3 PE=3 SV=1
75786000 48247000 109370000 149160000 126270000 127620000 107860000 63162000
98989000 172390000 112220000 189550000 6 6 6 37,2 37,2 37,2
13,783 0 43,076 5412900000 138 Q5S3G4 Q5S3G4 1047 sp|Q5S3G4|COX5B_PIG Cytochrome c oxidase subunit 5B, mitochondrial OS=Sus scrofa OX=9823 GN=COX5B PE=2 SV=1
0 0 0 0 0 0 0 0 0 0 0 0 2 2 2 14,8 14,8 14,8
15,524 0 3,7541 54514000 6 Q6QAP7;A0A286ZLG7 Q6QAP7;A0A286ZLG7 1048
sp|Q6QAP7|RS17_PIG 40S ribosomal protein S17 OS=Sus scrofa OX=9823 GN=RPS17 PE=1 SV=3;tr|A0A286ZLG7|A0A286ZLG7_PIG 40S ribosomal protein S17 OS=Sus scrofa OX=9823 PE=3 SV=1
10385000 7319200 7454500 15334000 8087300 8373800 15284000 8675800 6312400 11289000
7686600 7513300 3 3 3 46,4 46,4 46,4 7,8409 0 11,449
235210000 35 Q6QAT1 Q6QAT1 1049 sp|Q6QAT1|RS28_PIG 40S ribosomal protein S28 OS=Sus scrofa OX=9823 GN=RPS28 PE=1 SV=2
189180000 91181000 94117000 62148000 72367000 107600000 86788000 50480000 74430000
128270000 70755000 100140000 12 12 12 47,9 47,9 47,9 39,545 0

121,12 5541900000 201 Q6QGC0;D3GGC9;A0A480QG05;I3L9B6

Q6QGC0;D3GGC9;A0A480QG05 1050 sp|Q6QGC0|PDLI3_PIG PDZ and LIM domain protein 3 OS=Sus scrofa OX=9823 GN=PDLIM3 PE=2 SV=1;tr|D3GGC9|D3GGC9_PIG Actinin-associated LIM protein 3 OS=Sus scrofa OX=9823 GN=PDLIM3 PE=1 SV=1;tr|A0A480QG05|A0A480QG05_PIG PDZ and LIM domain protein 3 OS=Sus scro

11425000 0 0 14674000 0 0 17923000 0 0 16540000 0 0 3 3

2 33 33 23,2 12,323 0 5,2962 153940000 5 Q712U6 Q712U6 1052

sp|Q712U6|ARP19_PIG cAMP-regulated phosphoprotein 19 OS=Sus scrofa OX=9823 GN=ARPP19 PE=2 SV=3

0 0 0 0 0 0 0 0 0 0 0 0 1 1 1 4 4 4

28,399 0,0049751 1,615 22119000 4 Q85ZW4 Q85ZW4 1053

tr|Q85ZW4|Q85ZW4_PIG MHC class II antigen OS=Sus scrofa OX=9823 GN=SLA-DRA1 PE=1 SV=1

18851000 7688600 11762000 22254000 12451000 18353000 24282000 0 14567000 24296000 13702000

17679000 5 5 5 17,5 17,5 17,5 20,571 0 9,067 483810000 26

Q864V5;A0A5G2QLL4 Q864V5;A0A5G2QLL4 1054 sp|Q864V5|FUND2_PIG FUN14 domain-containing protein 2 OS=Sus scrofa OX=9823 GN=FUNDC2 PE=2 SV=1;tr|A0A5G2QLL4|A0A5G2QLL4_PIG FUN14 domain-containing protein 2 OS=Sus scrofa OX=9823 GN=FUNDC2 PE=1 SV=1

0 0 0 0 0 0 0 0 0 0 0 0 1 1 1 3,7 3,7 3,7

27,517 0,0031949 2,0833 14791000 6 Q8HXL4 Q8HXL4 1055

tr|Q8HXL4|Q8HXL4_PIG Cytoskeleton-associated protein 1 OS=Sus scrofa OX=9823 GN=TBCB PE=1 SV=1

455670000 56364000 682700000 467630000 281190000 748650000 231200000

187470000 278480000 316990000 313120000 596850000 8 8

8 42,8 42,8 42,8 18,891 0 43,084 21867000000 244 Q8MHY0;F1RKG0 Q8MHY0

1056 tr|Q8MHY0|Q8MHY0_PIG MYL2 OS=Sus scrofa OX=9823 GN=MYL2 PE=1 SV=1

28527000 0 0 78813000 0 156070000 42511000 107340000 167040000 0

136810000 185510000 2 2 2 28,8 28,8 28,8 9,0144 0 38,549

1943900000 36 Q8SPJ9 Q8SPJ9 1057 sp|Q8SPJ9|CX7A1_PIG Cytochrome c oxidase subunit 7A1, mitochondrial OS=Sus scrofa OX=9823 GN=COX7A1 PE=3 SV=1

0 7924200 0 7381700 0 0 7951500 7784100 0 9758500 8601700 0

2 2 2 24,1 24,1 24,1 9,3447 0,0011494 2,732 171370000 6

Q95KL4;A0A287AMJ4 Q95KL4;A0A287AMJ4 1058 sp|Q95KL4|SELW_PIG Selenoprotein W OS=Sus scrofa OX=9823 GN=SELENOW PE=3 SV=4;tr|A0A287AMJ4|A0A287AMJ4_PIG Selenoprotein W OS=Sus scrofa OX=9823 GN=SELENOW PE=4 SV=2

104760000 47889000 117710000 145190000 108910000 106190000 128580000

56715000 78324000 209850000 102350000 193930000 2 2 2 32,4

32,4 32,4 8,2314 0 23,015 2132800000 85 Q9MYT8;A0A5S6GNK8

Q9MYT8;A0A5S6GNK8 1059 sp|Q9MYT8|ATP5I_PIG ATP synthase subunit e, mitochondrial OS=Sus scrofa OX=9823 GN=ATP5ME PE=1 SV=4;tr|A0A5S6GNK8|A0A5S6GNK8_PIG ATP synthase membrane subunit e OS=Sus scrofa OX=9823 GN=ATP5ME PE=3 SV=1

87980000 105330000 129360000 184670000 145940000 130580000 177870000 115590000 146800000 234470000 166640000 178450000 9 9 9 40,6 40,6 40,6 30,726 0 59,28 5339200000 98 Q9MZ16;A0A287AVV2;A0A2C9F3B2;A0A5S6HVC8 Q9MZ16;A0A287AVV2;A0A2C9F3B2;A0A5S6HVC8 1060 sp|Q9MZ16|VDAC1_PIG Voltage-dependent anion-selective channel protein 1 OS=Sus scrofa OX=9823 GN=VDAC1 PE=2 SV=3;tr|A0A287AVV2|A0A287AVV2_PIG Voltage-dependent anion-selective channel protein 1 OS=Sus scrofa OX=9823 GN=VDAC1 PE=1 SV=2;tr|A0A2C9F3B2|A0A2C9F

0 0 12993000 0 10761000 12129000 0 7337600 9920200 0 9885400 21666000 3 3 3 5 5 5 68,649 0,0011481 2,7164 220910000 14 Q9TDR1 Q9TDR1 1061 sp|Q9TDR1|NU5M_PIG NADH-ubiquinone oxidoreductase chain 5 OS=Sus scrofa OX=9823 GN=MT-ND5 PE=1 SV=1

53694000 71673000 66924000 114690000 96956000 72846000 118830000 63131000 55116000 111050000 86165000 89390000 10 10 10 38,4 38,4 38,4 25,037 0 23,989 4236100000 110 Q9TSX9 Q9TSX9 1062 sp|Q9TSX9|PRDX6_PIG Peroxiredoxin-6 OS=Sus scrofa OX=9823 GN=PRDX6 PE=2 SV=3

252490000 455450000 168730000 44672000 171470000 77955000 45279000 124380000 132670000 54135000 154700000 133930000 83 83 0 43,8 43,8 0 223,23 0 323,31 16610000000 605 Q9TV62 Q9TV62 1063 sp|Q9TV62|MYH4_PIG Myosin-4 OS=Sus scrofa OX=9823 GN=MYH4 PE=2 SV=1

74069000 43415000 114840000 24016000 18998000 67793000 28707000 11489000 28185000 33294000 20828000 27552000 10 10 10 27,5 27,5 27,5 39,899 0 41,204 1817700000 114 Q9XSD9;A0A5G2RB02;F1SQ10;A0A5G2QKZ7;A0A5G2QHJ3;A0A5G2Q800;A0A5G2QIH8 Q9XSD9;A0A5G2RB02;F1SQ10;A0A5G2QKZ7 1064 sp|Q9XSD9|PGS2_PIG Decorin OS=Sus scrofa OX=9823 GN=DCN PE=2 SV=1;tr|A0A5G2RB02|A0A5G2RB02_PIG Decorin OS=Sus scrofa OX=9823 GN=DCN PE=3 SV=1;tr|F1SQ10|F1SQ10_PIG Decorin OS=Sus scrofa OX=9823 GN=DCN PE=3 SV=2;tr|A0A5G2QKZ7|A0A5G2QKZ7_PIG Decorin OS=Sus sc
